# LncRNA MACC1-AS1 induces gemcitabine resistance in pancreatic cancer cells through suppressing ferroptosis

**DOI:** 10.1038/s41420-024-01866-y

**Published:** 2024-02-27

**Authors:** Jiyun Zhu, Zehao Yu, Xuguang Wang, Jinghui Zhang, Yi Chen, Kaibo Chen, Bin Zhang, Jianhong Sun, Jianshuai Jiang, Siming Zheng

**Affiliations:** 1 Hepatopancreatobiliary Surgery Department, The First Affiliated Hospital of Ningbo University, Ningbo, China; 2 FUJIAN Medical University, Fuzhou, China; 3 Health Science Center, Ningbo University, Ningbo, China; 4 Emergency Department, The First Affiliated Hospital of Ningbo University, Ningbo, China; 5 Department of Surgery, The Second Affiliated Hospital, Zhejiang University School of Medicine, Hangzhou, China; 6 Department of Traditional Chinese Medicine, The First Affiliated Hospital of Ningbo University, Ningbo, China

**Keywords:** Pancreatic cancer, Oncogenes

## Abstract

Pancreatic ductal adenocarcinoma (PDA) mortality is primarily attributed to metastasis and chemotherapy resistance. In this research, the long non-coding RNA MACC1-AS1 was studied, playing a significant role in regulating lipid oxidation processes. This regulation could further lead to the inhibition of ferroptosis induced by chemotherapeutic drugs, making it a contributing factor to gemcitabine resistance in PDA. In both gemcitabine-resistant PDA patients and mouse models, the elevated expression level of MACC1-AS1 in the tumors was noted. Additionally, overexpression of MACC1-AS1 in pancreatic cancer cells was found to enhance tolerance to gemcitabine and suppress ferroptosis. Proteomic analysis of drug-resistant pancreatic cells revealed that overexpressed MACC1-AS1 inhibited the ubiquitination degradation of residues in the protein kinase STK33 by MDM4. Furthermore, its accumulation in the cytoplasm activated STK33, further activating the ferroptosis-suppressing proteins GPX4, thereby counteracting gemcitabine-induced cellular oxidative damage. These findings suggested that the long non-coding RNA MACC1-AS1 could play a significant role in the ability of pancreatic cancer cells to evade iron-mediated ferroptosis induced by gemcitabine. This discovery holds promise for developing clinical therapeutic strategies to combat chemotherapy resistance in pancreatic cancer.

## Introduction

Pancreatic cancer is an infrequent malignant tumor, comprising only 2%–3% of all cancer cases [1]. However, it is associated with a significantly high mortality rate of 7% [2]. At present, gemcitabine is the most commonly used chemotherapeutic drug for pancreatic cancer. Regrettably, the overall response rate to gemcitabine in treating pancreatic cancer is below 20%, and a staggering 80% of patients experience recurrence and succumb within 1 year [3]. Despite its effectiveness in advanced metastatic patients, the outcomes of gemcitabine are unsatisfactory, which can be mainly attributed to the development of resistance.

Ferroptosis is an iron-dependent, lipid peroxide accumulation-driven regulated cell death process [4]. Studies have shown that pancreatic cancer cells utilize antioxidant systems associated with ferroptosis to eliminate reactive oxygen species (ROS) as a strategy to evade cell death [5]. The Nrf2-ARE pathway plays a significant role in cellular antioxidant stress response [6]. Additionally, the Keap1-Nrf2 pathway inhibits ferroptosis by regulating the expression level of the downstream target gene SLC7A11 [7]. A great number of studies have highlighted the involvement of non-coding RNAs, such as microRNAs (miRNAs) and long non-coding RNAs (lncRNAs), in the regulation of ferroptosis [8]. Among them, MACC1-AS1 has been identified as an antisense RNA of MACC1 mRNA. Studies have demonstrated that MACC1-AS1 plays a role in stabilizing the expression level of MACC1 through the adenosine-activated protein kinase/Lin28 pathway, promoting cellular behaviors, including proliferation and invasion of gastric cancer cells [9]. Although several lncRNAs have been associated with the resistance of pancreatic cancer cells, no research has concentrated on the relationship between MACC1-AS1 and the resistance of pancreatic cancer cells. Previous in vitro and in vivo studies have demonstrated that overexpression of STK33 promotes the malignant biological behaviors of pancreatic ductal adenocarcinoma (PDAC) cells, while its inhibition suppresses tumor cell malignancy [10]. This phenomenon can be attributed to the direct interaction between hypoxia-inducible factor-1α (HIF-1α) and the hypoxia response element located on the STK33 promoter [11]. This interaction leads to the activation of STK33 promoter activity, thereby promoting the malignant progression of PDAC.

In the present study, the role of the lncRNA MACC1-AS1 in pancreatic cancer tissue and cell line resistance was investigated and validated. The findings revealed that the MACC1-AS1/STK33 axis plays a crucial role in promoting resistance to gemcitabine by inhibiting iron-mediated ferroptosis. Moreover, the therapeutic effects of inhibiting MACC1-AS1 and STK33 were independently evaluated in a clinical setting. Additionally, clinical prediction DCA models were developed for both targets, presenting novel avenues for future research on the clinical treatment of pancreatic cancer.

## Results

### LncRNA MACC1-AS1 was highly expressed in pancreatic cancer cells both in vitro and in vivo, and it was associated with poor prognosis in the context of gemcitabine resistance

**Fig. 1:**
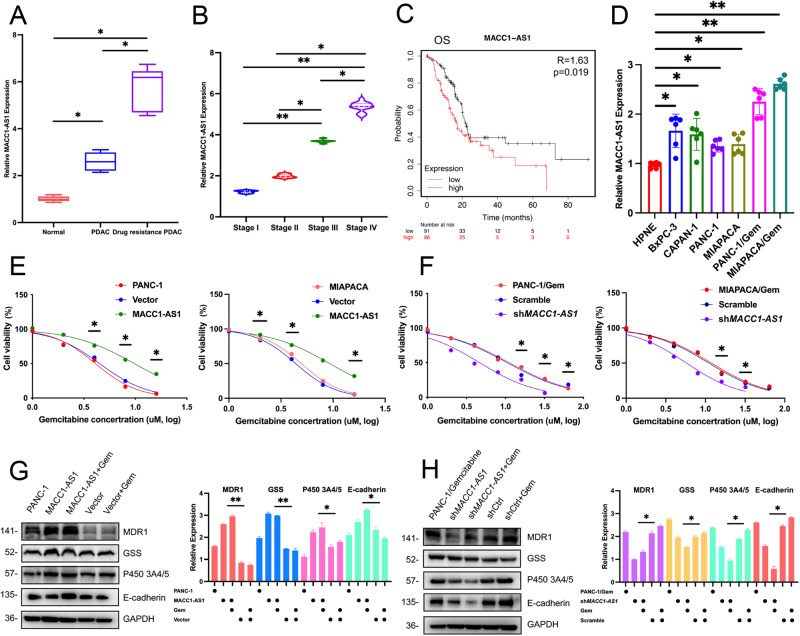
lncRNA MACC1-AS1 is highly expressed in pancreatic cancer both in vitro and in vivo, and is associated with poor prognosis in gemcitabine resistance. **A**, **B** The expression of MACC1-AS1 in PDAC, drug-resistant PDAC, normal patients and in four different clinical stages. **C** Overall survival (OS) of MACC1-AS1 in PDAC patients from TCGA data analysis. **D** RNA expression of MACC1-AS1 in six different pancreatic cancer cells and one normal pancreatic cell. **E**, **F** Cell viability of downregulation and upregulation of MACC1-AS1 gene in PANC-1/Gem, MIAPACA/Gem and PANC-1, MIAPACA respectively under the treatment of Gemcitabine (20 uM). G-H Protein level of four tumor drug resistance-related gene (MDR1, GSS, P450 3A4/5 E-cadherin) under conditions of up and down expression of MACC1-AS1 in PANC-1 and PANC-1/Gem cells, accompanied by statistical results of the expression intensities on the right (values represented mean ± SD. ★*P* < 0.05, ★★*P* < 0.01 versus control). COX analysis was conducted for (**A**, **B**). Student *t*-test was used for (**E**–**H**).

In order to indicate whether MACC1-AS1 plays a role in the development and drug resistance of pancreatic cancer, pancreatic cancer tissues and cell lines obtained from our center and public databases were examined. MACC1-AS1 expression level in pancreatic cancer samples obtained from our center was determined, and it was indicated that it was highly expressed in tumor tissues of PDAC patients compared with normal pancreatic tissues (Fig. 1A). In addition, MACC1-AS1 was found to be highly expressed in stage II, III, and IV pancreatic cancer patients, especially in advanced stage (stage IV) patients, with statistically significant differences in pairwise comparisons (*P* < 0.05) (Fig. 1B). The analysis of data from TCGA database revealed that MACC1-AS1 was associated with unfavorable prognostic outcomes in terms of overall survival (OS) (*P* = 0.019) (Fig. 1C). Furthermore, MACC1-AS1 expression level was significantly elevated in tissues from patients who developed resistance to gemcitabine after initial chemotherapy (*P* < 0.05). Additionally, it was noted that MACC1-AS1 was highly expressed in the most of pancreatic cancer cell lines, and patients from whom the high-expressing cell lines originated exhibited varying degrees of drug resistance (Supplementary Fig. 1A, B). These findings were further validated in pancreatic cancer cell lines and corresponding gemcitabine-resistant cell lines (Fig. 1D). After confirming the significantly higher drug resistance of gemcitabine-resistant cell lines compared with regular pancreatic cancer cell lines, MACC1-AS1 was stably overexpressed in PANC-1 and MIAPACA cell lines, and MACC1-AS1 was stably knocked down in gemcitabine-resistant PANC-1/Gem and MIAPACA/Gem cell lines (Fig. 1E, F). The results indicated that stable knockdown of MACC1-AS1 significantly reduced the resistance of gemcitabine-resistant cell lines (half maximal inhibitory concentration (IC50) = 4.643, 5.781), while stable overexpression of MACC1-AS1 exhibited a higher resistance to gemcitabine (IC50 = 9.749, 9.295). In pairwise comparisons between the control and experimental groups of the four cell lines, statistically significant differences were found at 2–3 different gemcitabine concentrations (*P* < 0.05). After investigating the drug resistance ability of MACC1-AS1 at the cellular level, the RNA expression of MACC1-AS1 and the expression levels of drug-resistant marker proteins were subsequently determined under different treatment conditions at the RNA and protein levels. As illustrated in Supplementary Fig. 1C, regular pancreatic cancer cell lines exhibited decreased RNA expression of MACC1-AS1 when exposed to moderate doses of gemcitabine. However, gemcitabine-resistant cell lines showed a higher expression level of MACC1-AS1. Furthermore, in gemcitabine-resistant cell lines with the stable knockdown of MACC1-AS1, there was a sustained decrease in RNA expression of MACC1-AS1 under similar gemcitabine concentrations. At the protein level, when pancreatic cancer cell lines were exposed to low concentrations of gemcitabine, it was noted that the expression levels of drug resistance-related proteins increased. Notably, the stable overexpression of MACC1-AS1 resulted in a significant upregulation of drug-resistant marker proteins, particularly MDR1 and E-cadherin proteins (Fig. 1G, H). In gemcitabine-resistant cell lines with stable knockdown of MACC1-AS1, the expression levels of drug resistance-related proteins were significantly suppressed, especially under gemcitabine stimulation. In summary, the analysis of the subcellular localization of MACC1-AS1 revealed that its expression was predominantly in the cytoplasmic fractions, with lesser presence in nuclear fractions (Supplementary Fig. 1D).

### MACC1-AS1 directly interacted with STK33 to induce gemcitabine resistance

**Fig. 2:**
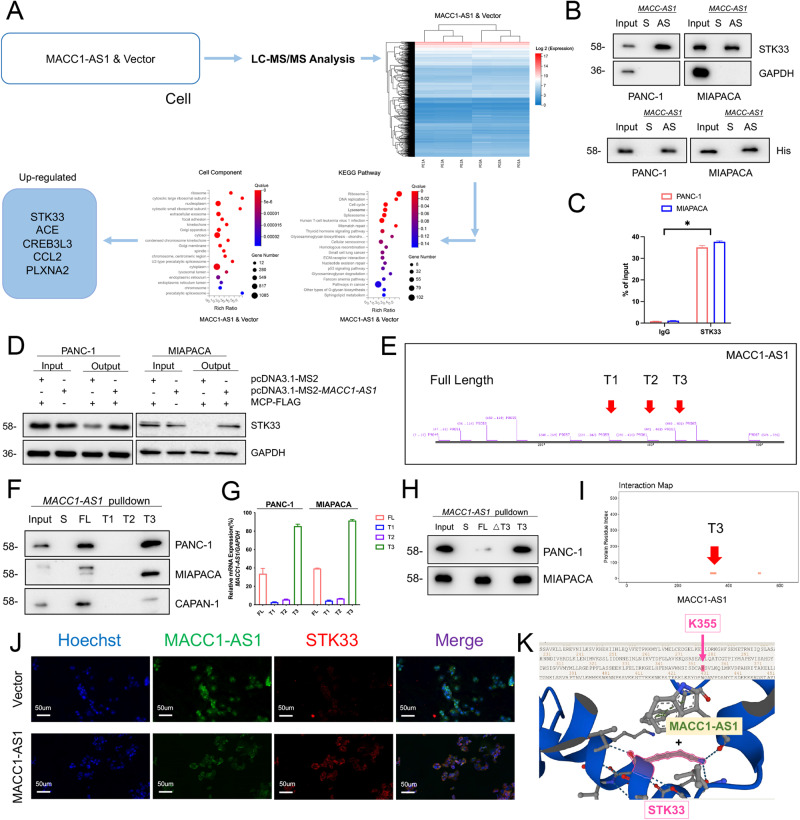
MACC1-AS1 directly interacts with STK33 to induce gemcitabine resistance. **A** LC-MS/MS analysis was used to evaluate the downstream target via MACC1-AS1 overexpression PANC-1 cell, accompanied by vector cells. At the same time, KEGG pathway and cell components were put into work to analyze the results and to select the target. **B** Cell lysates of STK33 or purified His-tagged STK33 was pulled down by biotin-labeled MACC1-AS1 but not by *MACC1-AS1* sense RNA (S, sense. AS, antisense). **C** RIP assays of MACC1-AS1, precipitated with STK33 in whole-cell lysates, and the RNA levels of *MACC1-AS1* and β-actin were measured by qPCR analysis. **D** *MACC1-AS1*-binding proteins were tested by Western blot analysis, using anti-FLAG antibody affinity agarose beads and specific antibodies to identify the targets. **E**, **F** The full length of MACC1-AS1 and the T1, T2, T3 fragments were selected for further research from the predictive website, and RNA pull-down and Western blot assays were performed using synthesized FL and specific fragment of MACC1-AS1, which were incubated with cell lysates or purified His-tagged recombinant STK33. **G** CLIP-qPCR research of T1/T2/T3 fragment of MACC1-AS1 for STK33 binding was conducted. **H** RNA pull-down assays was used to confirm the MACC1-AS1 T3 fragment, interacting with STK33. **I**–**K** The interaction map, IF and Alphafold database were conducted to show the interaction fraction, cell co-location and specific part (K355) of STK33, which binds with MACC1-AS1 (T3 fragment).

After confirming that MACC1-AS1 may play a key role in inducing resistance of pancreatic cancer cells to gemcitabine, it was attempted to explore the specific mechanisms involved. The proteomic analysis was conducted to identify and analyze the targets of MACC1-AS1. Notably, differential analysis was undertaken by comparing cells overexpressing MACC1-AS1 with cells infected with an empty virus, followed by their combination with data from the KEGG signaling pathway (Fig. 2A). It was found that the antisense MACC1, rather than the sense MACC1, could directly interact with the protein kinase STK33 (Fig. 2B). The result was further validated by purifying the antisense MACC1 tagged with His (Fig. 2B). Additionally, RNA immunoprecipitation (RIP) experiment confirmed the potential interaction between MACC1-AS1 and STK33 (Fig. 2C). To further investigate the in vivo interaction between MACC1-AS1 and STK33, MS2-labeled RNA affinity purification was carried out using magnetic beads and immunoblotting. Co-expression of MS2-MACC1-AS1 and MCP-3FLAG plasmids showed significant enrichment of STK33 compared with negative control expression, suggesting that STK33 could specifically bind to MACC1-AS1 (Fig. 2D).

Furthermore, secondary structure analysis performed by software indicated that MACC1-AS1 mainly contains three major binding segments (Fig. 2E). In order to determine the specific regions of MACC1-AS1 that could interact with STK33, RNA pull-down assay was conducted using in vitro synthesized full-length MACC1-AS1 (FL) and T1 (390–410), T2 (441–461), and T3 (480–501) fragments, followed by Western blot analysis of the products. The results revealed that the T3 fragment could bind to STK33, while the other fragments or control groups yielded negative results (Fig. 2F). Besides, cross-linking immunoprecipitation and quantitative polymerase chain reaction (CLIP-qPCR) and pulldown experiments were undertaken, which further confirmed that the T3 fragment was identified as the main binding region for STK33 (Fig. 2G, H). Thereafter, immunofluorescence (IF) co-localization analysis showed that MACC1-AS1 and STK33 were mainly co-localized in the cytoplasm, suggesting that their complex could primarily function in the cytoplasm (Fig. 2J). Combining the abovementioned experimental results with predictions from the PhosphoSitePlus database [12], it was found that amino acid residue 355 of STK33 could serve as the potential binding site for MACC1-AS1 (Fig. 2K).

### MACC1-AS1 enhanced the activity of STK33 by preventing its ubiquitination and degradation

**Fig. 3:**
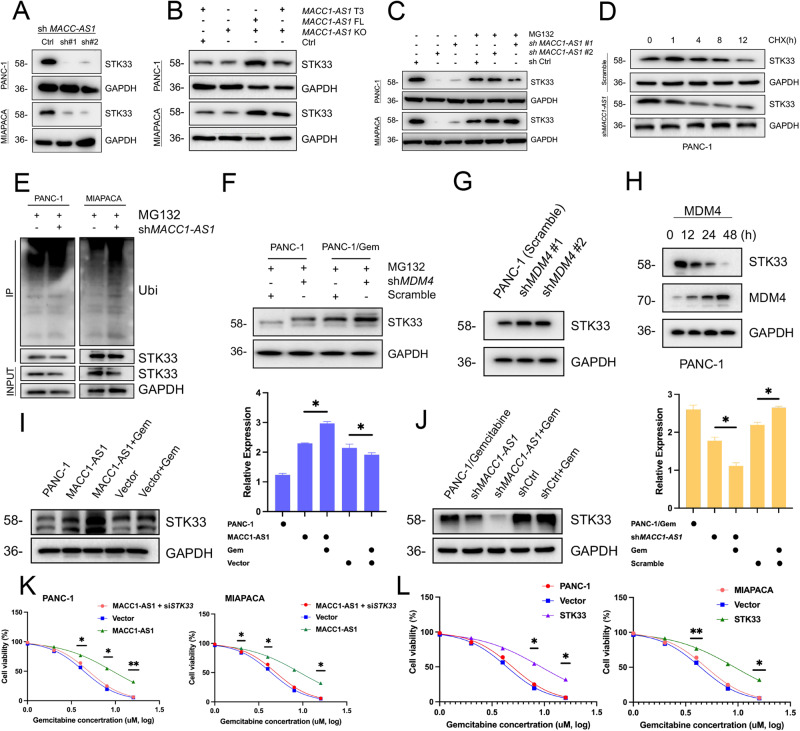
MACC1-AS1 enhances the activity of STK33 by preventing its ubiquitination and degradation. **A** The expression of STK33 was measured under the condition of downregulation of MACC1-AS1 in PANC-1 and PANC-1/Gem cells. **B** STK33 level was tested when treated with MACC1-AS1 CRISPR KO tool to study and verify the MACC1-AS1 FL or T3 fragment. **C** STK33 stability was explored by downregulation of MACC1-AS1 and treatment of MG-132 (10 μM, 12 h). **D** Western blotting detection of STK3 expression with the condition of scramble or sh*MACC1-AS1*, following treatment with CHX (100 μg/ml) for the indicated times. **E** WB assays were used to evaluate STK33 ubiquitination levels under MACC1-AS1 knockdown condition. **F**–**H** WB analysis was conducted to assess the relationship between MDM4 and STK33 with the condition of sh*MDM4* + MG132, sh*MDM4* and the time accumulation of MDM4. **I**, **J** The STK33 expression was explore in overexpression of MACC1-AS1 cell or treatment of Gemcitabine, while down-expression of MACC1-AS1 in drug resistance cell or treatment with Gemcitabine to evaluate the level of STK33, accompanied by statistical results of the expression intensities on the right. **K**, **L** The rescue assay of overexpression of MACC1-AS1 with downregulation of STK33 in PANC-1 and MIAPACA cells, while cell viability of upregulation of STK33 in both above cells was conducted. (values represented mean ± SD. ★*P* < 0.05, ★★*P* < 0.01 versus control).

Next, the functional influence of the interaction between MACC1-AS1 and STK33 was assessed. The results revealed that knockdown of MACC1-AS1 under shRNA conditions significantly downregulated the expression level of STK33 in MACC1-AS1 KO cells using knockdown techniques (Fig. 3A). Furthermore, overexpression of the T3 fragment of MACC1-AS1 segment, rather than the MACC1-AS1 full-length fragment, rescued the decrease in STK33 protein level caused by MACC1-AS1 knockout. These results suggested that MACC1-AS1 could regulate STK33 protein level through the T3 segment (Fig. 3B).

Previous studies have demonstrated that STK33 can be degraded by proteasomes through multiple ubiquitination mechanisms [13]. Therefore, the proteasome inhibitor MG-132 was utilized, and it was indicated that MG-132 restored the reduced STK33 expression level (Fig. 3C). Additionally, knockdown of MACC1-AS1 shortened the half-life of STK33, highlighting a significant influence on the stability of STK33 in pancreatic cancer cells (Fig. 3D). Furthermore, IP and Western blotting were carried out to analyze the ubiquitination level of STK33 in cells with MACC-AS1 knockdown (Fig. 3E). It was noted that knockdown of MACC1-AS1 significantly increased the ubiquitination level of STK33. These results demonstrated that MACC1-AS1 could be involved in stabilizing STK33. Regarding the mRNA level of STK33, MACC1-AS1 knockdown slightly affected STK33 RNA expression (Supplementary Fig. 2A). Additionally, the influence of MACC1-AS1 on the ubiquitination of STK33 by MDM4 was identified through ubiquitination prediction results and correlation analysis of mRNA data from PDAC patients (Supplementary Fig. 2B, D). Attempts were made to elevate STK33 protein level by conducting UBA52 knockdown assay. In the downregulation analysis, it was found that the knockdown of MDM4 resulted in increased expression level of STK33 in both PANC-1 and PANC-1/Gem cells, demonstrating a time-dependent pattern, while it did not lead to an increase in the ubiquitination of STK33 (Fig. 2F–H and Supplementary Fig. 2C), suggesting that MACC1-AS1 could stabilize STK33 by preventing ubiquitination. Therefore, MACC1-AS1 could stabilize STK33 by suppressing MDM4-mediated ubiquitination of STK33.

Thus, MACC1-AS1 could enhance the stability of STK33 by inhibiting its ubiquitination, leading to the increased accumulation of STK33 in cancer cells. Furthermore, it was revealed that stable overexpression of STK33 enhanced the resistance of pancreatic cancer cells to gemcitabine (Fig. 3L). Additionally, in both gemcitabine-treated normal pancreatic cancer cells and drug-resistant cells at low concentrations, STK33 protein expression was upregulated, with its higher expression level was found in drug-resistant cells (Fig. 3I, J). In contrast, stable knockdown of MACC1-AS1 in drug-resistant cells exhibited a significant reduction in STK33 expression level (Fig. 3J). Thereafter, the influence of MACC1-AS1 on STK33 in cells was examined (Fig. 3K). In cell lines with stable overexpression of MACC1-AS1, a striking impact on the resistance to gemcitabine was found when plasmids were transfected aimed at knocking down STK33. These results demonstrated that MACC1-AS1 could primarily rely on the function of STK33 in the resistance to gemcitabine.

### MACC1-AS1/STK33 induced resistance to gemcitabine by inhibiting ferroptosis

**Fig. 4:**
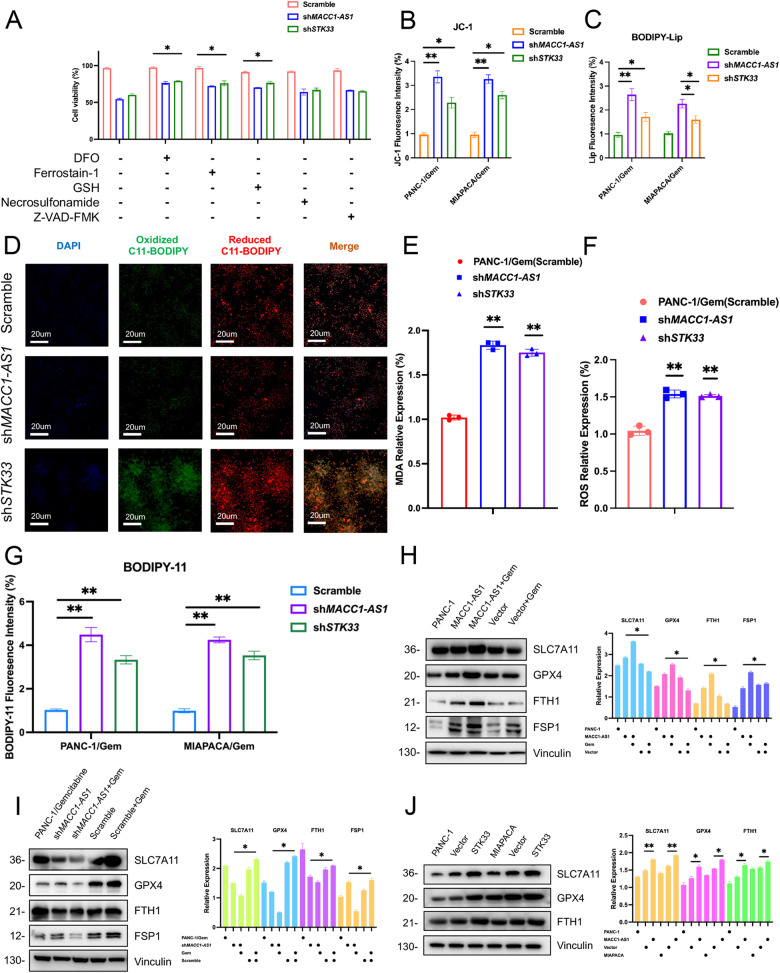
MACC1-AS1/STK33 generates gemcitabine resistance by inhibiting ferroptosis. **A** Cell viability was detected by CCK8 assay under condition of scramble, sh*MACC1-AS1* and sh*STK33* in the treatment or absence of different cell death inhibitors. **B**–**G** Representative images of JC-1, lipid staining, BODIPY C-11, MDA and ROS assay were captured by confocal laser microscope with the above condition (Scale bars: 20 μm). **H**–**J** WB assays of ferroptosis inhibiting proteins (SLC7A11, GPX4, FTH1, FSP1) were conducted under the condition of overexpression of MACC1-AS1, knockdown of MACC1-AS1, or upregulation of STK33, accompanied by statistical results of the expression intensities on the right (values represented mean ± SD. ★*P* < 0.05, ★★*P* < 0.01 versus control).

To further investigate the mechanism of gemcitabine resistance and inhibition of cell death in pancreatic cancer cells, pancreatic cancer cells that had a reduction in MACC1-AS1 or STK33 expression were utilized. These cells were treated with or without different cell death inhibitors. These inhibitors included deferoxamine (DFO, an iron chelator), ferrostatin-1 (fer-1, a ferroptosis inhibitor), Z-VAD-FMK (a necroptosis inhibitor), necrostatin-1 (a necroptosis inhibitor), and glutathione. The results showed that the iron chelator, ferroptosis inhibitor, and glutathione significantly alleviated the inhibitory effects of reduced MACC1-AS1 or STK33 expression on the viability of pancreatic cancer cells (Fig. 4A). Lipid peroxidation is one of the main indicators of ferroptosis. Therefore, the degree of oxidative stress in pancreatic cancer cells was evaluated by downregulating MACC1-AS1 or STK33 expression. The following methods were employed to assess oxidative stress level [1]: measurement of total ROS production using BODIPY C11 probe (Fig. 4D, G) [2], measurement of lipid ROS using JC-1 and Lip BODIPY fluorescence probes (Fig. 4B, C), and [3] detection of malondialdehyde (MDA) as a lipid peroxidation product (Fig. 4E, F). These results indicated that inhibition of MACC1-AS1 or STK33 expression could lead to the increased cellular ROS and lipid peroxidation, particularly causing changes in lipid probes and mitochondrial membrane potential probes. In terms of specific mechanisms, recent studies have demonstrated that the increased ROS production may induce cell death by inhibiting the PI3K/Akt, NF-Κb, NRF2, and MAPK signaling pathways. Pancreatic cancer cells were subsequently treated with downregulated MACC1-AS1 or STK33 using AKT-IN-9 (an Akt inhibitor), Nrf2-IN-1 (an NRF2 inhibitor), JSH-23 (an NF-κB inhibitor), SB203580 (a p38 inhibitor), SP600125 (a JNK inhibitor), and SCH772984 (an ERK inhibitor). The results revealed that suppression of Akt, NF-κB, JAK2, and ERK further enhanced cytotoxicity. These findings suggested that the cytotoxic effects induced by knockdown of MACC1-AS1 or STK33 might not be mediated through these pathways, while they could be regulated through ferroptosis or other signaling pathways (Supplementary Fig. 3A, B). To further validate the association with ferroptosis, the expression levels of ferroptosis-related marker proteins were examined. The results indicated an unchanged in the RNA (Supplementary Fig. 3C) and increased protein levels of ferroptosis-related proteins, particularly GPX4 and SLC7A11 (Fig. 4H–J).

### MACC1-AS1/STK33 inhibited the degradation of GPX4 in ferroptosis

**Fig. 5:**
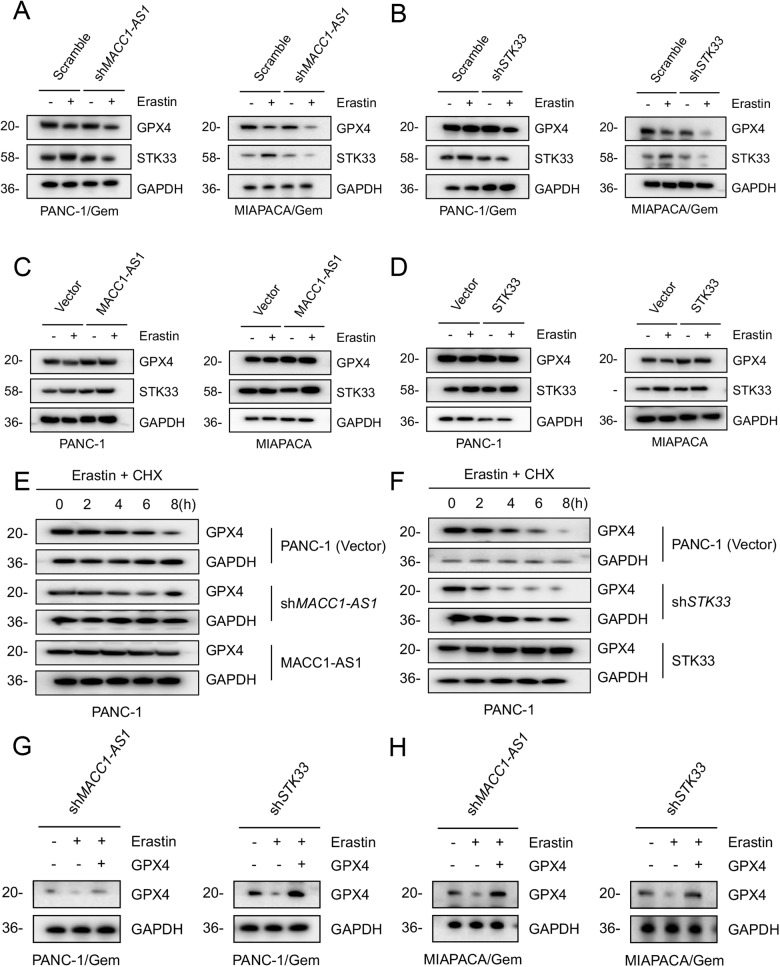
MACC1-AS1/STK33 inhibits GPX4 degradation in ferroptosis. **A**, **B** The effects of knockdown of MACC1-AS1 or STK33 on the expression of STK33 and GPX4 at protein levels in indicated PDAC drug resistance cells following treatment with erastin (30 μM) for 24 h. **C**, **D** The effects of overexpression of MACC1-AS1 or STK33 by gene transfection on the expression of GPX4 protein as well as STK33 in PANC-1 and MIAPACA cells following treatment with erastin (30 μM) for 24 h. **E**, **F** MACC1-AS1 or STK33 knockdown or overexpression PANC1 cells were treated with 25 μM erastin and 25 μg/ml cycloheximide (CHX) for designed time points and Western blot analysis was conducted to evaluate the results. **G**, **H** Overexpression of GPX4 restored GPX4 expression in MACC1-AS1 or STK33 knockdown PDAC drug resistance cells following treatment with erastin (30 μM) for 24 h.

GPX4 is a key enzyme that inhibits lipid peroxidation in ferroptosis and based on the finding that MACC1-AS1/STK33 could contribute to drug resistance by inhibiting ferroptosis, it was attempted to investigate whether MACC1-AS1/STK33 could regulate lipid peroxidation and inhibit ferroptosis induced by gemcitabine through influencing the expression level of GPX4. Erastin did not significantly affect the mRNA expression of GPX4, and knockdown of MACC1-AS1 or STK33 did not significantly influence GPX4 mRNA levels in PANC1/Gem and MIAPACA/Gem cells (Supplementary Fig. 3D, E). However, knockdown of MACC1-AS1 or STK33 promoted the degradation of GPX4 protein induced by erastin in both drug-resistant cells (Fig. 5A, B), while overexpression of MACC1-AS1 or STK33 inhibited the erastin-induced degradation of GPX4 protein (Fig. 5C, D). As expected, the expression level of GPX4 was suppressed upon knockdown of MACC1-AS1 or STK33. In contrast, overexpression of STK33 increased GPX4 protein level in erastin-induced ferroptosis. These findings suggested a potential role of MACC1-AS1/STK33 in regulating GPX4 protein level. To further validate whether MACC1-AS1/STK33 could affect the degradation of GPX4 protein, it was found that knockdown of MACC1-AS1 or STK33 decreased the stability of GPX4 protein in PANC1 cells after erastin treatment, while overexpression of MACC1-AS1 or STK33 extended the stability of GPX4 protein, indicating that MACC1-AS1/STK33 could prevent the degradation of GPX4 protein induced by erastin (Fig. 5E, F).

Additionally, it was attempted to assess whether the degradation of GPX4 upon loss of the MACC1-AS1/STK33 pathway could lead to ferroptosis. Overexpression of GPX4 through gene transfection restored the expression level of GPX4 in drug-resistant cells with knockdown of MACC1-AS1 or STK33 (Fig. 5G, H). Collectively, these findings demonstrated that MACC1-AS1/STK33 inhibited ferroptosis by protecting the degradation of GPX4 protein.

### Kras regulated MACC1-AS1 to maintain gemcitabine resistance in pancreatic cancer cells

**Fig. 6:**
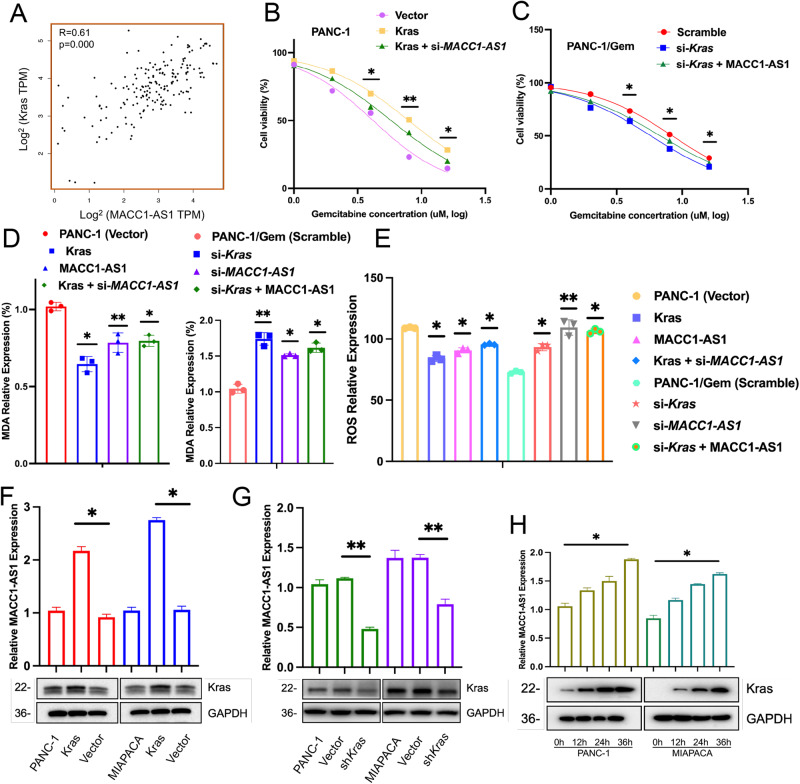
Kras regulates MACC1-AS1 to maintain gemcitabine resistance in pancreatic cancer cells. **A** Correlation analysis of MACC1-AS1 and Kras expression was conducted in a cohort of PDAC patients from TCGA data. **B**, **C** The cell viability was used to evaluate the function of MACC1-AS1 and Kras on the condition of overexpression of Kras, accompanied by si*MACC1-AS1* in PANC-1 cell, and knockdown of Kras, accompanied by upregulation of MACC1-AS1 in PANC-1/Gem cell. **D**, **E** MAD and ROS assays following the above conditions were measured. **F**, **G** qPCR detection of MACC1-AS1 expression in overexpression of Kras and knockdown of Kras cells. **H** qPCR assay of MACC1-AS1 expression under the treatment of 30 ng per/ml Doxycycline to induce ectopic Kras expression for 0, 12, 24, 36 h. The *P*-value was determined by a two-tailed unpaired Student’s *t*-test.

Considering the well-established oncogenic functions of MACC1-AS1/STK33 in the previous findings, the present study attempted to unravel the upstream mechanism through which MACC1-AS1/STK33 contributes to gemcitabine resistance in pancreatic cancer cells. Given that Kras mutations are prevalent in ~80% of pancreatic cancer patients, the potential correlation between Kras and MACC1-AS1 was assessed [14]. The analysis of data from TCGA database indicated a significantly positive correlation between MACC1-AS1 expression and Kras (Fig. 6A). In pancreatic cancer cell lines and resistant cell lines, overexpression of Kras and simultaneous knockdown of MACC1-AS1 or knockdown of Kras and simultaneous overexpression of MACC1-AS1 resulted in different levels of drug resistance. Specifically, a high expression level of Kras significantly enhanced the drug resistance of pancreatic cancer cell lines, while simultaneous knockdown of MACC1-AS1 remarkably reversed the drug resistance (Fig. 6B, C). In resistant cell lines, suppression of Kras significantly reduced their resistance to gemcitabine, which could be reversed by simultaneous overexpression of MACC1-AS1. Furthermore, the correlation between Kras/MACC1-AS1 and ferroptosis-related oxidative reactions was explored (Fig. 6D, E). In the MDA and ROS detection experiments, it was found that the upregulation of Kras or MACC1-AS1 led to a notable reduction in gemcitabine-induced oxidative reactions associated with ferroptosis. However, when Kras was highly expressed and MACC1-AS1 was knocked down simultaneously, the extent of oxidative damage resulting from gemcitabine treatment was significantly reduced. Moreover, in resistant cell lines, the oxidative damage caused by gemcitabine could be resisted, while inhibition of Kras or MACC1-AS1 significantly increased the level of oxidative damage, and when MACC1-AS1 was simultaneously upregulated in pancreatic cancer cell lines, it enhanced their resistance to oxidative reactions.

Thereafter, we examined the upregulation and inhibition of Kras gene expression. Compared with control cells, pancreatic cancer cells with a high expression level of Kras showed a higher expression level of MACC1-AS1. On the other hand, pancreatic cancer cells with a low expression level of Kras exhibited a lower expression level of MACC1-AS1 (Fig. 6F, G). Additionally, the time-dependent correlation between Kras and MACC1-AS1 under the influence of Kras was investigated. The results indicated that as the concentration and duration of Kras increased, the expression level of MACC1-AS1 was also upregulated (Fig. 6H). The abovementioned findings suggested that under the influence of gemcitabine, upregulation of MACC1-AS1 induced by Kras could potentially contribute to drug resistance in pancreatic cancer cell lines.

### Potential application of the MACC1-AS1/STK33 axis in pancreatic cancer animal and cellular models

**Fig. 7:**
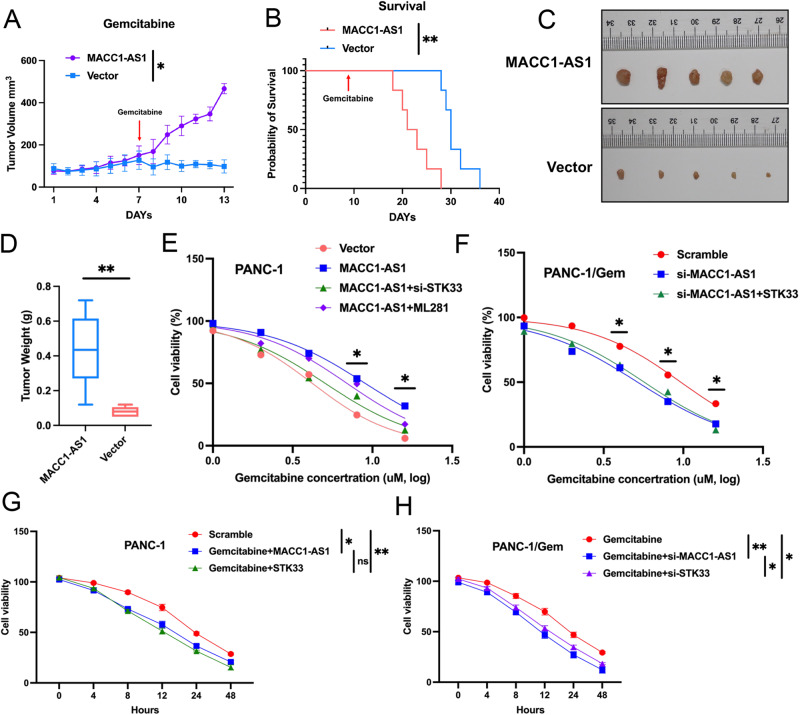
Potential application of the MACC1-AS1/STK33 signaling axis in pancreatic cancer animal and cell models. **A**–**D** The tumor volume, survival time, tumor photo and tumor weight were measured from the animal model injected with overexpression of MACC1-AS1 and control PANC-1 cells, with the treatment of Gemcitabine on the 7th day. **E**, **F** PANC-1 and PANC-1/Gem cells were co-transfected with the indicated vectors/MACC1-AS1/si-STKSS/ML281(treatment) and scramble/si-MACC1-AS1/STK33, by treated with Gemcitabine at different concentrations, after which cell viability was measured by CCK8 assay. **G**, **H** PANC-1 and PANC-1/Gem cells were co-transfected with the indicated scramble/MACC1-AS1/STKSS and scramble/si-MACC1-AS1/si-STK33, by treated with Gemcitabine at different hours, after which cell viability was measured by CCK8 assay. Data were represented as mean ± SD, *P < 0.05; **P < 0.01.

To investigate the therapeutic effects of the MACC1-AS1/STK33 axis on pancreatic cancer resistance, the chemotherapeutic response of resistant animals to gemcitabine after stable overexpression of MACC1-AS1 was examined (Fig. 7A–D). The tumor growth curve, mouse survival curve, and tumor size experiment results revealed that animals with the high expression level of MACC1-AS1 exhibited a significant drug resistance. Despite receiving gemcitabine treatment, their tumors continued to grow and enlarge, without significant inhibition in their growth trend. In contrast, the control group treated with gemcitabine exhibited a noticeable inhibition. Furthermore, the results of survival analysis demonstrated that the group with the high expression level of MACC1-AS1 had a significantly shorter survival time compared with the control group (Fig. 7B). Besides, the viability of pancreatic cancer cell lines treated with different concentrations of gemcitabine and for different durations was assessed (Fig. 7E–H). The results revealed that compared with cells with MACC1-AS1 overexpression alone, siSTK33 co-transfection and STK33 inhibitors reduced the gemcitabine IC50 value in pancreatic cancer cell lines exhibiting a high expression level of MACC1-AS1 to some extent. Similarly, in resistant cell lines with stable knockdown of MACC1-AS1, co-transfection of STK33 overexpression plasmid enhanced their resistance to gemcitabine compared with knocked down cell lines alone. In addition, compared with cells with MACC1-AS1 overexpression alone, co-transfection of STK33 siRNA and MACC1-AS1 overexpression plasmid in pancreatic cancer cell lines reduced cell viability and exhibited time-dependent effects. Moreover, it was found that knockdown of MACC1-AS1 significantly reduced cell viability and gemcitabine IC50 value, while co-expression with STK33 restored cell viability and gemcitabine resistance.

### Clinical prognostic potential of the MACC1-AS1/STK33 axis in pancreatic cancer patients

**Fig. 8:**
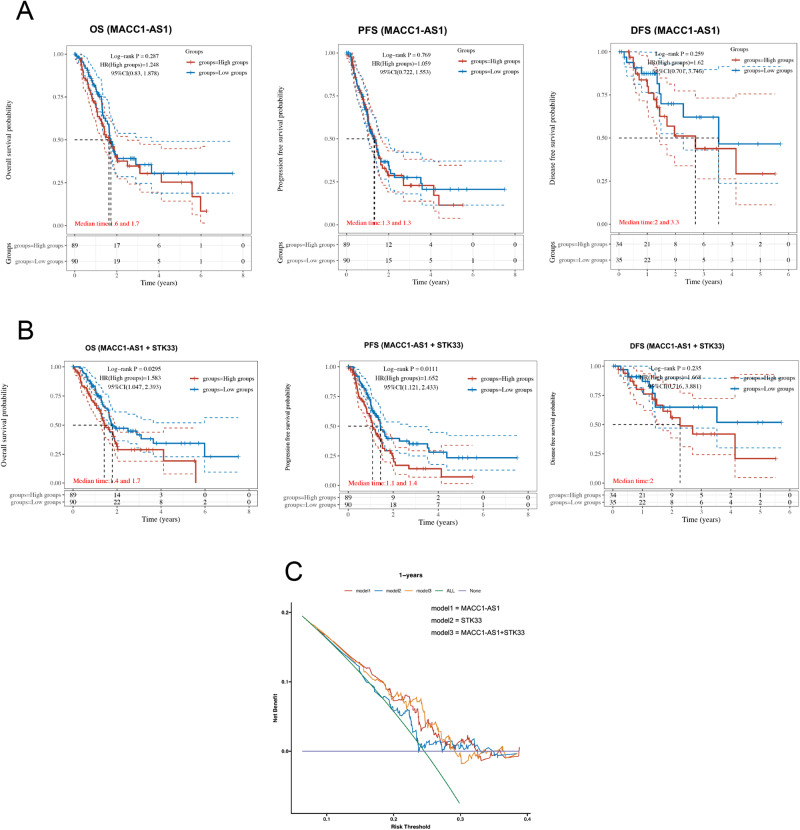
Clinical prognostic potential of the MACC1-AS1/STK33 signaling axis in pancreatic cancer patients. **A**, **B** The OS, PFS and DFS of MACC1-AS1, MACC1-AS1 + STK33 were analyzed from TCGA PADC patients respectively. **C** The combination of MACC1-AS1 and STK33, MACC1-AS1, STK33 were used to establish the DCA module and analyzed 1 year OS time for PDAC patients.

In terms of clinical application, it was attempted to analyze the expression level of MACC1-AS1, STK33 and both genes in pancreatic cancer patients and their prognostic significance (Fig. 8A–C). Patients were firstly divided into high-risk and low-risk groups. Then, the survival status of the two groups was illustrated, and the heat map was displayed, representing the gene expression levels of MACC1-AS1 and MACC1-AS1 + STK33. The KM survival curve showed that patients in the high-risk group had different OS, PFS and DFS rates compared with those in the low-risk group, with different median survival time. The results indicated that high expression of MACC1-AS1 indicated poor survival time for OS, PFS and DFS in pancreatic cancer patients, while exhibiting no statistical significance. However, it was found that the combination of MACC1-AS1 and STK33 showed a significant impact on both OS and PFS rates. The results demonstrated that the combined analysis of MACC1-AS1 and STK33 demonstrated statistically significant results (*P* < 0.05) in terms of OS and PFS, while still with no significance for DFS. Moreover, this combined approach exhibited a superior performance compared with using either gene expression alone. Furthermore, a DCA (Decision Curve Analysis) analysis was established for MACC1-AS1, STK33 and both, the risk scores for each patient were calculated, and different DCA modules was obtained using the “ggDCA” R package [15]. Patients were divided into five modules (Fig. 8C). According to the results of the DCA analysis, the combined prognostic efficacy of MACC1-AS1 and STK33 exhibited a higher DCA value, indicating that the multi-gene model had a greater predictive ability for 1 year OS. These data demonstrated that MACC1-AS1 and STK33 could be utilized as combined targets for clinical prognosis in pancreatic cancer patients.

## Discussion

Pancreatic cancer is one of the most serious malignant tumors worldwide, ranking seventh in incidence and sixth in mortality among all malignancies [16]. Although immunotherapy has recently made some progress in the treatment of pancreatic cancer, chemotherapy remains the mainstay of treatment for pancreatic cancer. Gemcitabine is a first-line chemotherapeutic drug for the treatment of pancreatic cancer. However, long-term utilization of gemcitabine can lead to drug resistance and poor treatment outcomes [17]. Therefore, it is of great significance to investigate the causes and mechanisms of gemcitabine resistance in pancreatic cancer.

In the present study, it was found that the long non-coding RNA MACC1-AS1 plays an important role in resistance to gemcitabine in pancreatic cancer cell lines, and it is a potential therapeutic target. While we initially reported the involvement of MACC1-AS1 in gemcitabine resistance specifically in pancreatic cancer, subsequent studies have highlighted its role in promoting drug resistance in other types of cancer, including gastric cancer and esophageal squamous cell carcinoma [18, 19]. Furthermore, it was revealed that MACC1-AS1 directly interacts with the protein kinase STK33 at the 355 amino acid site, acting as a stabilizing factor. This interaction prevents the ubiquitination and subsequent degradation of STK33, leading to its accumulation in the cytoplasm. Consequently, the upregulation of downstream signaling pathways occurs, ultimately contributing to the development of drug resistance. Consistent with our findings, Zhang et al. found that MACC1-AS1 acts as a sponge to bind multiple miRNAs and RNA-binding protein PTBP1, thereby exerting oncogenic effects [20]. Wang et al. reported that MACC1-AS1 directly interacts with UPF1 to promote tumor stemness in non-small cell lung cancer [21]. Additionally, Zhao et al. demonstrated that MACC1-AS1 interacts with and stabilizes its complementary RNA strand, MACC1 [9]. This interaction has been found to play a role in the regulation of metabolic reprogramming and the promotion of carcinogenesis in gastric cancer cell lines. In addition to uncovering the roles of MACC1-AS1 in metabolic reprogramming and carcinogenesis, the present study explored its inhibitory effects on ferroptosis-associated lipid metabolism. This inhibition was found to counteract the cytotoxic effect of gemcitabine. It is noteworthy that no similar study has yet concentrated on MACC1-AS1 in other types of tumors.

Furthermore, the present study on MACC1-AS1 revealed that it plays a crucial role in promoting the cytoplasmic accumulation of STK33, consequently leading to the inhibition of ferroptosis. Notably, previous research has reported a higher protein expression of STK33 in various histological types of lung cancer tissues compared with benign lung tissues [22]. Additionally, the remarkable expression level of STK33 has been linked to lung cancer patients’ poor survival outcomes. Through Western blotting, it has been noted that the expression level of the STK33 protein was positively associated with the phosphorylation level of extracellular regulated protein kinases (ERK). This suggests that the downregulation of the ERK/STK33 signaling pathway may play a role in promoting the inhibitory process [13]. In addition, dephosphorylation of ERK has been found to induce apoptosis of non-small cell lung cancer (NSCLC) cells and decrease the resistance of NSCLC to gefitinib [23]. In summary, our research findings align with previous studies and aim to investigate and validate the underlying mechanisms of STK33 in tumor drug resistance.

In conclusion, this study has identified a novel intracellular signaling pathway that plays a role in gemcitabine resistance in pancreatic cancer. Through a series of explorations and validations, this finding may provide new insights and theoretical basis for future clinical applications.

### Limitations of the study

To fully harness the therapeutic potential of our research findings, it is essential to undertake additional efforts to validate the clinical implications of the MACC1-AS1/STK33 axis. Conducting clinical studies with well-designed drug treatment regimens is crucial to explore the clinical efficacy in gemcitabine-resistant pancreatic cancer patients. Such studies will shed light on the outcomes associated with reversing chemotherapeutic drug resistance mediated by the MACC1-AS1/STK33 axis. In conclusion, it is imperative to conduct additional research aimed at delving deeper into the specific mechanisms through which MACC1-AS1/STK33 regulates ferroptosis. To unravel these underlying natural phenomena, it is crucial to employ precise and appropriate experimental protocols and techniques.

## Materials and methods

### Patients’ samples

The human pancreatic cancer tissue samples were obtained from the First Affiliated Hospital of Medical School of Ningbo University (Ningbo, China). These tissue samples from 30 patients with pancreatic cancer were collected by means of drawing lots for evaluating MACC1-AS1 expression level. It is noteworthy that research consent was written and collected from all patients involved in the study. The research protocol was in advance approved by the Institutional Review Board of the First Affiliated Hospital of Medical School of Ningbo University (Approval No. 2022-118A-01).

### Cell and cell culture

The human PDAC cell lines PANC-1 and MIAPACA were purchased from the Cell Bank of Chinese Academy of Sciences (Shanghai, China). PANC-1/Gem and MIAPACA/Gem were purchased from the IMMOCELL (Xiamen, China). All cell lines were confirmed using short tandem repeat (STR) markers. All of these cell lines cannot be viewed in the International Cell Line Authentication Committee Database of Cross- contaminated or Misidentified Cell Lines (https://iclac.org/databases/cross-contaminations/). All cells were cultivated with DMEM medium with 10% fetal bovine serum, penicillin (100 mg/mL) and streptomycin (100 U/mL) and all of these were previously purchased from Gibco (Grand Island, NY, USA). Cells were maintained in a designed atmosphere of 5% carbon dioxide at 37 ◦C. All cell lines were regularly detected for mycoplasma and chlamydia contamination and were confirmed to be contamination-free.

### Lentiviral and retroviral production and infection

Gene downregulation by short hairpin RNAs (shRNA) targeting human MACC1-AS1, STK33, Kras were loaded into the lentiviral vector pLKD-CMV-GFP-U6-shRNA. Small interfering RNA (siRNA) targeting MACC1-AS1, STK33 and Kras were constructed followed by protocol. MACC1-AS1, STK33, GPX4 and Kras overexpression genes were produced by PCR amplification and then inserted into the lentiviral vector pLOV-CMV-eGFP. Lentiviruses encoding targeted genes shRNA plasmids, the scrambled shRNA plasmid, the siRNA, the human above overexpression genes and the vector were all manufactured by GENE Chem (Shanghai, China).

### CRISPR/Cas9-mediated gene deletion

MACC1-AS1 KO cell lines were conducted using a CRISPR/Cas9-based tool. MACC1-AS1-specific guide RNA (gRNA) expression vectors were purchased from GENE Chem (Shanghai, China).

### Cell proliferation assay

Cell proliferation was accessed with CCK8 (HY-K0301) (MCE, Shanghai, China) according to the manufacturer’s instructions.

### MDA and ROS analysis

MDA and ROS assay measured by Lipid Peroxidation MDA Assay Kit and Reactive Oxygen Species Assay Kit, respectively. (Beyotime Biotech. Inc., Shanghai, China).

### C11-BODIPY, JC-1 and Lip- BODIPY analysis

C11-BODIPY 581/591, JC-1 and Lip-BODIPY 493/503 analysis were conducted following to the manufacturer’s instructions (HY-D1301, HY-K0601, HY-W090090, MCE, Shanghai, China).

### RIP and RNA pulldown analysis

RIP assays were conducted using a Magna RNA-binding protein immunoprecipitation kit (Millipore, Bedford, MA) and carried out following the manufacturer’s instructions. RNA pull-down assays were also performed with a Pierce Magnetic RNA–Protein Pull-Down Kit (Thermo Fisher Scientific, USA).

### CLIP-qPCR

CLIP-qPCR assay was performed in this experiment using a crosslinking immunoprecipitation and qPCR kit (Bes3014) (BersinBio, Guangzhou, China). The protocol was conducted according to the manufacturer’s instructions to clarify the specific sequence of MACC1-AS1 binding to STK33. During the process, 10 μg of normal IgG (Abcam, ab172730) or STK33 antibody (Abcam, ab206296) were used.

### Subcellular fractionation

A Cytoplasmic & Nuclear RNA Purification Kit (Norgen Biotek Corp, Canada) was adopted to evaluate MACC1-AS1 level in cytoplasmic and nuclear fractions following to the manufacturer’s instructions.

### Fluorescence in situ hybridization (FISH) assay and immunofluorescence staining

FISH assays were carried out with a lncRNA FISH Kit (RiboBio, Guangzhou, China). In brief, cells and FISH probes were prepared. Then hybridization was conducted according to the protocol. All images were obtained with an Olympus FV1000 confocal microscope (Tokyo, Japan). Besides, immunofluorescence staining was proceed following to the manufacturer’s instructions, and an anti-STK33 antibody (1:150, Abcam, ab206296) was used in this study.

### RT-qPCR and western blot analysis

RNA levels were measured by qPCR analysis following to the manufacturer’s instructions. Western blot analysis was also performed according to the manufacturer’s protocol. Briefly, cells or tissues were lysed in RIPA buffer. The protein concentrations were then normalized using a BCA assay kit (Solarbio, Beijing, China). Anti-GAPDH (1:1000, ABclonal, Wuhan, China, AC001), anti-STK33 (1:1000, Abcam, Cambridge, MA, USA, ab206296), anti-MDR1 (1:1000, Affinity, Jiangshu, China, AF5185), anti-GSS (1:1000, Affinity, DF6214), anti-MDM4 (1:1000, Affinity, DF8676), anti-P450 3A4/5 (1:1000, Affinity, AF5312), anti-E-cadherin (1:1000, Affinity, AF0131), anti-SLC7A11 (1:1000, Affinity, DF12509), anti-GPX4 (1:1000, Affinity, DF6701), anti-FTH1 (1:1000, Affinity, DF6278), anti-FSP1 (1:1000, Affinity, DF6516), anti-KRas (1:1000, Affinity, DF6324), and anti-ubiquitin antibodies (1:1000, Affinity, AF0289) were used in this study.

### Animal and tumor models

The Balb/c nude mice (male, 6-week-old) were provided by the Model Animal Research Center of Nanjing University. These mice were maintained in specific-pathogen-free (SPF) conditions at the Experimental Animal Center of Medical School of Ningbo University. Mice were randomly assigned to different groups for the experiment by means of drawing lots. PANC-1 cells, which were stable clones expressing RNA targeting MACC1-AS1 and a control plasmid, were injected subcutaneously into the right flanks of nude mice. Each mouse received 1 × 10^6^ cells. Additionally, mice were treated with Gem (4 μM) for 24 h, twice every week, starting from the 7th day after the beginning of this research. The tumor size was measured using calipers and documented. After 3 weeks, mice were euthanized, and the tumors were extracted for subsequent analysis. After euthanizing the nude mice, the tumors were dissected and removed, weighed, and photographed. The tumor volume was calculated using the formula *V* = 1/2 × *L* × *W*^2^ (*V*: volume, *L*: length of tumor, *W*: width of tumor). The growth curve of the tumors was plotted, and the differences in average tumor volume and weight among the groups were compared.

To assess survival time, PANC-1 parental cells and PANC-1 cells with MACC1-AS1 were mixed with 25 μL of medium and 12.5 μL of Matrigel, and they were then injected into the right flanks of mice. Each mouse received 1 × 10^6^ cells. The drug was administered following the above protocol. The time of death for each mouse was recorded. Animal experiments were complied with all relevant ethical regulations and approved by Ethics Committee of Ningbo University.

### Mass spectrometry

Two groups of cells were prepared for the experiment. One group underwent MACC1-AS1 overexpression, while the other group received a vector in PANC-1 cells. The cells were prepared in a gel using 50 mm ammonium bicarbonate buffer with RapiGest (Waters Corp., Milford, MA, USA) overnight at 37 °C with 200 ng of sequencing-grade modified trypsin (Promega, Madison, WI, USA). The resulting digest was tested by LC-MS/MS using an Orbitrap-Elite mass spectrometer. Protein identification was carried out by searching fragment spectra in the Swiss-Prot protein database (EBI) with the Mascot search engine (ver. 2.3; Matrix Science, London, UK) and SEQUEST (ver. 1.27; University of Washington, Seattle, WA, USA) through the Proteome Discoverer software (ver. 1.4; Thermo Fisher Scientific). Phosphopeptide matches were performed using the phosphoRS algorithm implemented in Proteome Discoverer and manually curated.

### Bioinformatic analysis

The data for MACC1-AS1 and STK33 gene expression were obtained from two databases: The Cancer Genome Atlas (TCGA) and the Genotype-Tissue Expression (GTEx). These databases provided a collection of pancreatic tumor samples and normal samples for analysis. The analysis process was preformed using R software.

### Kaplan–Meier survival analysis

Kaplan–Meier survival analysis was conducted to appraise diversities in the OS and RFS between the high-risk and low-risk groups. The R packages survMiner and survival were used to enable this evaluation.

### Establishing a predictive model

The predictive DCA (Decision Curve Analysis) model (MACC1-AS1 and STK33) for the 1-year OS was set up using the TCGA PDAC data. We analyze the DCA curve of different models (MACC1-AS1, STK33, MACC1-AS1 + STK33, All and None) using the ggDCA package in R software.

### Statistical analysis

The statistical results were presented as mean ± standard deviation (SD) of at least three independent biological replicates. To determine differences between the two groups, Student’s t-test was utilized, while differences among multiple groups were determined using one-way analysis of variance (ANOVA). Overall survival curves were analyzed using the Kaplan–Meier (KM) method, and the log-rank test was employed to compare the curves. Spearman’s rank correlation was used to assess the correlation between two variables. SPSS (ver. 20; IBM Corp., Armonk, NY, USA) and GraphPad Prism (ver. 8.0; GraphPad Software Inc., San Diego, CA, USA) software were utilized to carry out statistical analysis. *P* < 0.05 was considered statistically significant. In the figures, *P < 0.05, **P < 0.01, ***P < 0.001, and ****P < 0.0001.

### Supplementary information

Supplementary Figure Legends

supplementary figure 1

supplementary figure 2

supplementary figure 3

Clinical information of PDAC patients

WB Original data

## Data Availability

The datasets used in this study are available from the corresponding author on reasonable request.

## Code availability

The R code used in “Bioinformatic analysis”, “Kaplan–Meier survival analysis”, and “Establishing a predictive model” of the methodology can be obtained by searching the specific code and workflow on the official R website.
