# Supplementary material for: LncRNA MACC1-AS1 induces gemcitabine resistance in pancreatic cancer cells through suppressing ferroptosis
Source: Cell Death Discov. 2024 Feb 27;10:101. doi: 10.1038/s41420-024-01866-y (PMC10899202; doi:10.1038/s41420-024-01866-y)
Supplement: Supplementary file 1 — Supplementary Figure Legends [file 41420_2024_1866_MOESM1_ESM.docx]

**Supplementary Figure Legends**

**Supplementary Figure 1** **lncRNA MACC1-AS1 is highly expressed in pancreatic cancer both in vitro and in vivo, and is associated with poor prognosis in gemcitabine resistance.** **A** The RNA expression of MACC1-AS1 was analysis in pan-cancer cells. **B** Different PDAC cells data was collected to access the expression level of MACC1-AS1. **C** The expression of MACC1-AS1 was evaluated in cells treated with gemcitabine, including PANC-1&PANC-1/Gem, overexpression of MACC1-AS1 in PANC-1, and sh*MACC1-AS1* in PANC-1/Gem cells. **D** The level of MACC1-AS1 was tested in different cell locations in PANC-1 and MIAPACA cells.

**Supplementary Figure 2 MACC1-AS1 enhances the activity of STK33 by preventing its ubiquitination and degradation. A** The mRNA expression of STK33 was analyzed in drug resistance PDAC cells with sh*MACC1-AS1* via RT-qPCR tool. **B** The ubiquitination of STK33 by MDM4 was predicted using UbiBrowser 2.0. **C** The WB result of downregulation of MDM4 and the treatment of MG132 (10 μM, 12 h) was displayed in the PANC-1 cell. **D** The relation of MACC1-AS1 and STK33 was calculated from PDAC patients of TCGA database (P = 8e-04, R = 0.25).

**Supplementary Figure 3 MACC1-AS1/STK33 generates gemcitabine resistance by inhibiting ferroptosis and inhibits the degradation of GPX4 in ferroptosis. A-B** The cell viability of PANC-1 was evaluated with sh*MACC1-AS1* or sh*STK33*, along with various inhibitors: AKT-IN-9, Nrf-IN-1, JSH-23, SB203580, SP600125 or SCH772984. **C** The mRNA expression of ferroptosis relative protein (SCL7A11, GPX4, FTH1, FSP1) was accessed with gemcitabine treatment in PANC-1, PANC-1/Gem, overexpression of MACC1-AS1 and vector cells. **D-E** The mRNA expression of GPX4 was analyzed under the treatment of erastin (25 μM) and in or not in the condition of erastin (25 μM) or sh*MACC1-AS1*, sh*STK33*.
