# Supplementary figures and images for: LncRNA MACC1-AS1 induces gemcitabine resistance in pancreatic cancer cells through suppressing ferroptosis
Source: Cell Death Discov. 2024 Feb 27;10:101. doi: 10.1038/s41420-024-01866-y (PMC10899202; doi:10.1038/s41420-024-01866-y)

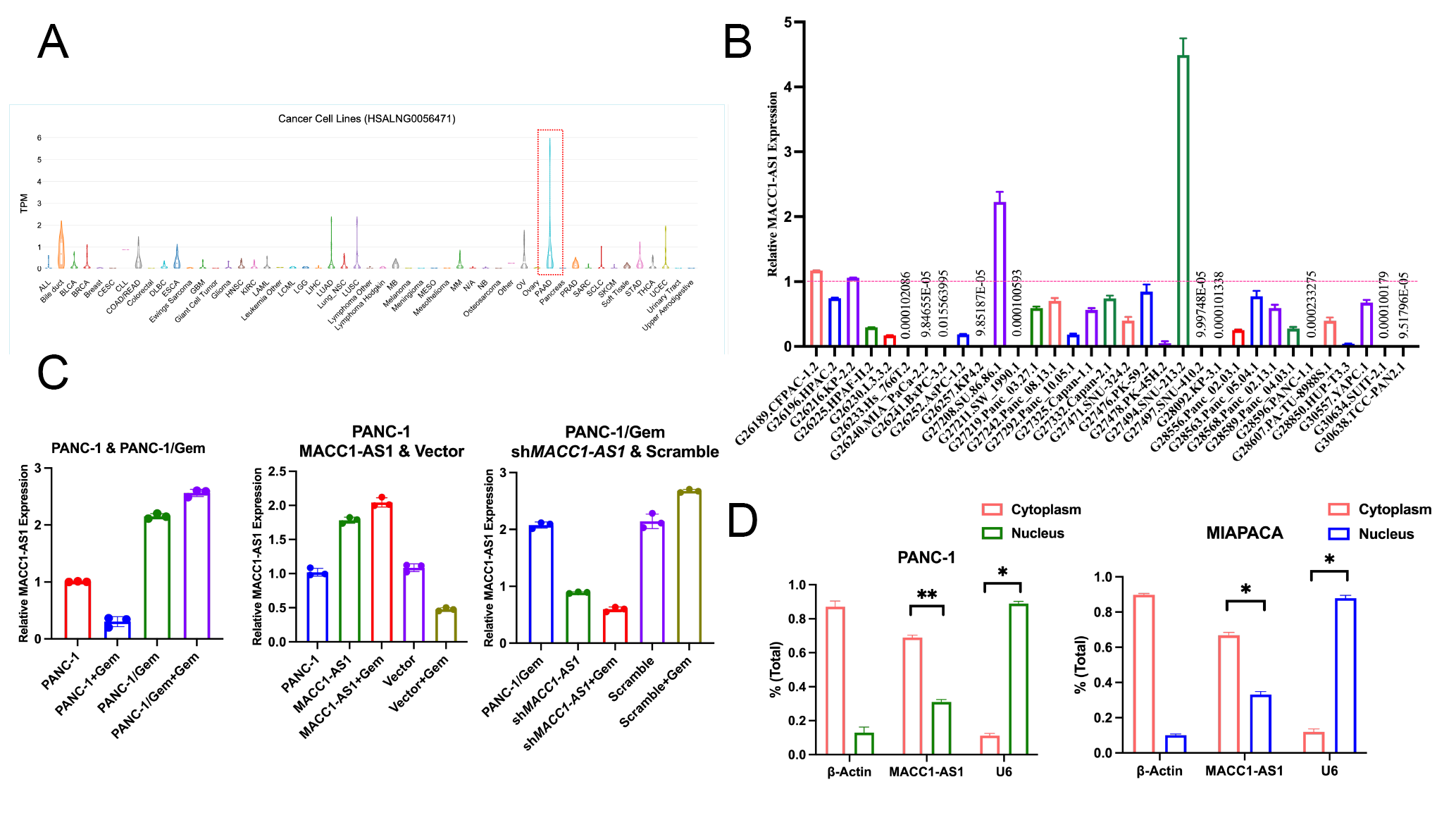

Supplement: Supplementary file 2 — supplementary figure 1 [file 41420_2024_1866_MOESM2_ESM.tif]

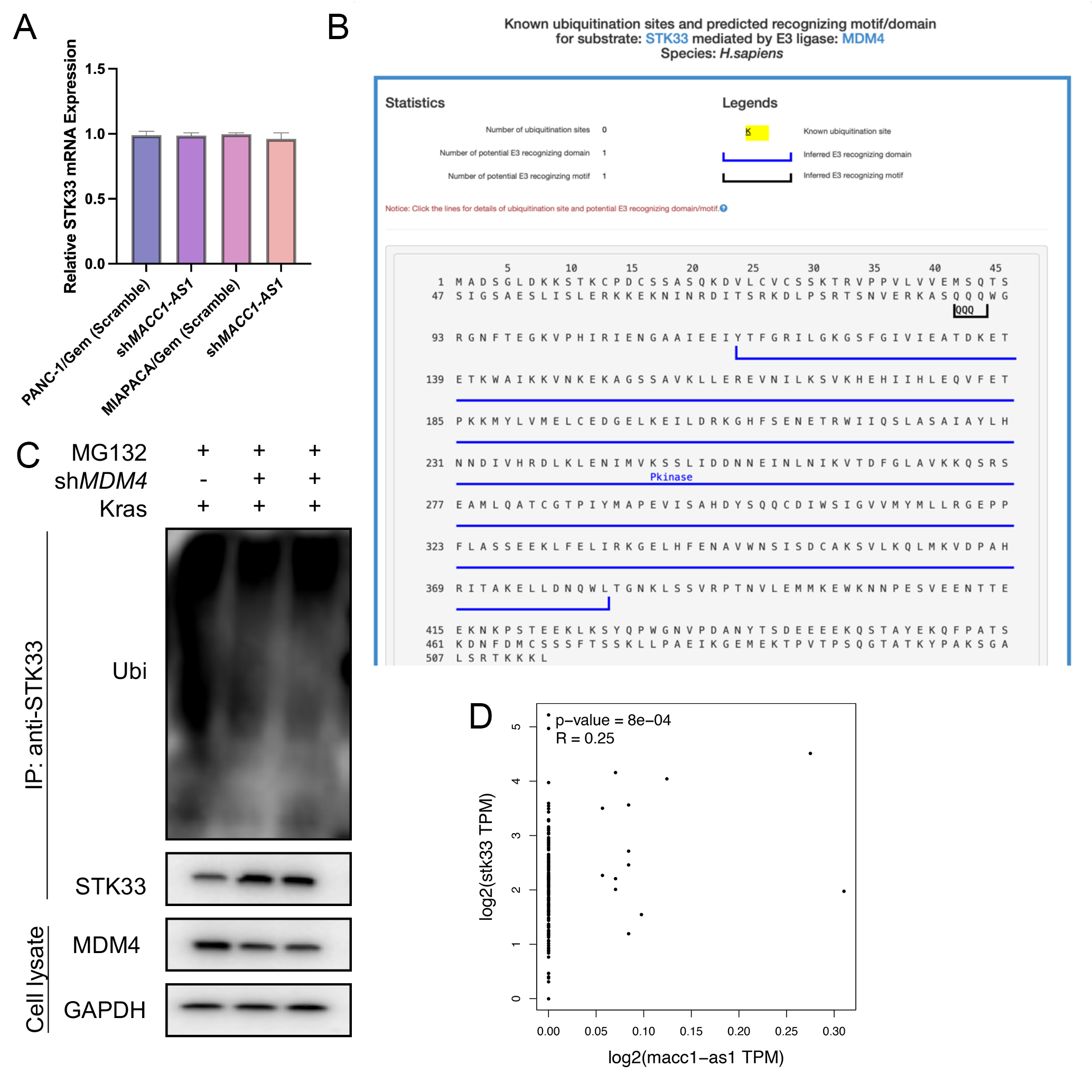

Supplement: Supplementary file 3 — supplementary figure 2 [file 41420_2024_1866_MOESM3_ESM.tif]

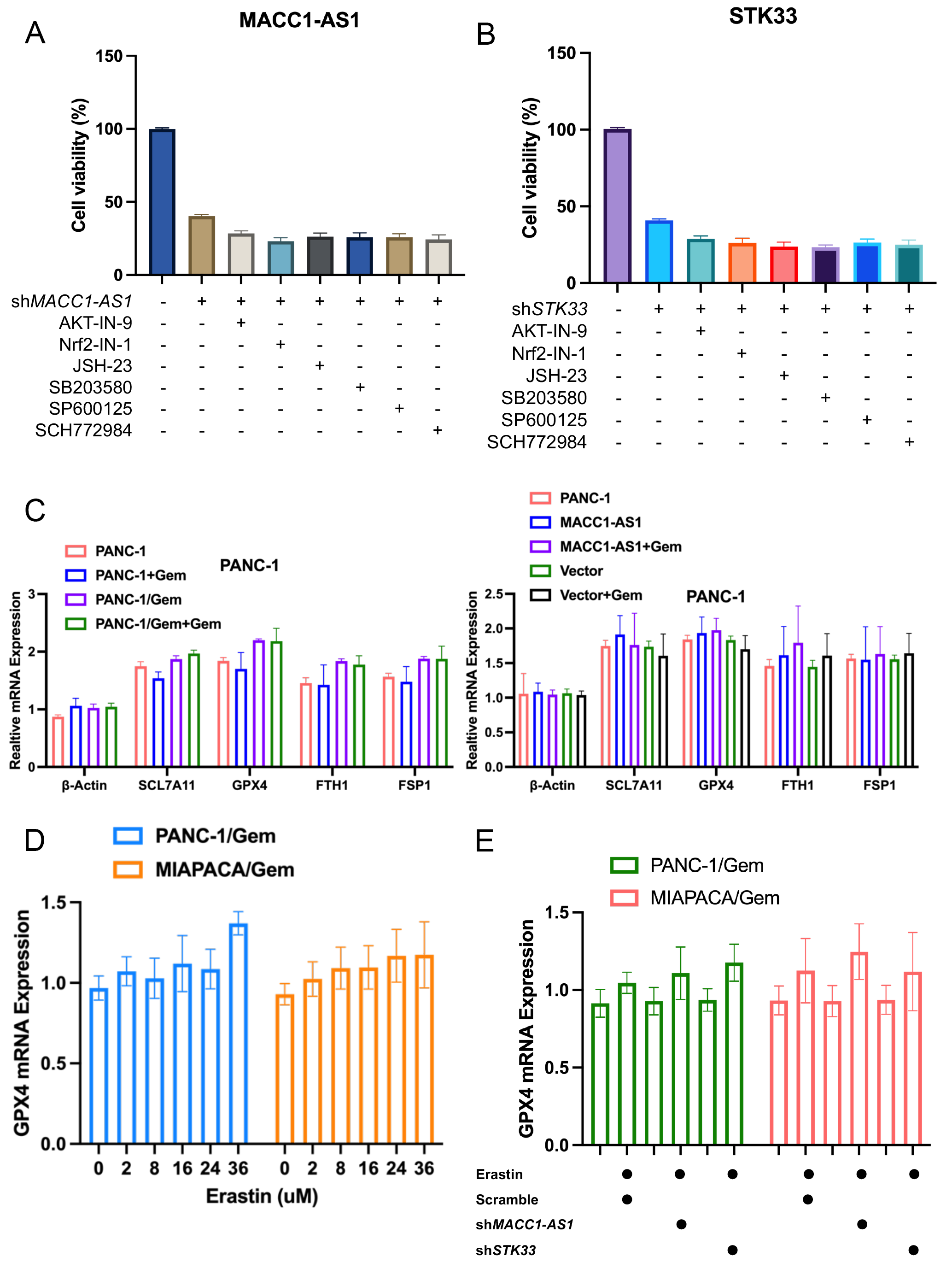

Supplement: Supplementary file 4 — supplementary figure 3 [file 41420_2024_1866_MOESM4_ESM.tif]
