# Supplementary material for: LncRNA MACC1-AS1 induces gemcitabine resistance in pancreatic cancer cells through suppressing ferroptosis
Source: Cell Death Discov. 2024 Feb 27;10:101. doi: 10.1038/s41420-024-01866-y (PMC10899202; doi:10.1038/s41420-024-01866-y)
Supplement: Supplementary file 5 — Clinical information of PDAC patients [file 41420_2024_1866_MOESM5_ESM.docx]

**Clinical information of PDAC patients**

**Study Population**

We retrospectively reviewed clinical data and collected 30 samples from patients who underwent operation at The First Affiliated Hospital of Ningbo University from December 2018 to December 2020.

The following inclusion criteria were applied: (a) a histologically confirmed pancreatic ductal adenocarcinoma; (b) no evidence of tumors invading adjacent organs, paraaortic lymph node enlargement or distant metastasis demonstrated by abdominal computed tomography and/or abdominal ultrasound and posteroanterior chest radiography; and (c) a lymphadenectomy with a curative R0 resection.

Definition of resistance: resistance is defined as the continued progression of the tumor in patients who have received neoadjuvant or adjuvant chemotherapy before or after surgery. It is mainly characterized by persistent elevation of serum tumor markers, evident evidence of tumor size or peripheral infiltration on imaging examinations, and the presence of significant clinical symptoms in patients.

The case exclusion criteria were as follows: (a) patients with T4b tumors, (b) metastatic disease, (c) other pathological types of pancreatic tumors, (d) patients with no available computed tomography (CT) imaging or with a preoperative CT image older than 30 days, and (e) patients with incomplete or inaccurate medical records.

All surgical procedures, including lymphadenectomy, and staging were performed according to the corresponding eighth edition of the American Joint Committee on Cancer (AJCC) Staging Manual [1]. Neoadjuvant chemotherapy or adjuvant chemotherapy regiments were followed by AJCC and were recommended for the patients with PDAC [2-3].

Sample collection method: After identifying 30 pancreatic cancer tumor samples that met the inclusion criteria in the sample repository of the First Affiliated Hospital of Ningbo University, we collected their slides, which were fixed with formalin after surgery. These slides were then collected, and the RNA component was extracted using chloroform, and the content of our target RNA was detected using RT-qPCR. The collection process followed relevant regulations for clinical tissue collection in the People's Republic of China. The samples were transported using liquid nitrogen.

Specimen collection criteria: After the specimens were obtained, they were divided into the following sections: ① Approximately 1.5 cm of tumor tissue, ② Within 1 cm of the tumor adjacent tissue, and ③ Approximately 1.5 cm of normal tissue adjacent to the specimen (at least 5 cm away from the tumor). If the tumor size was small, appropriate-sized specimens were cut according to the actual size of the tumor for storage.

Table 1. The clinicopathological parameters

**Reference:**

1. Siegel RL, Miller KD, Fuchs HE, Jemal A. Cancer statistics, 2022. CA Cancer J Clin. 2022;72(1):7-33. doi:10.3322/caac.21708

2. Collisson EA, Bailey P, Chang DK, Biankin A V. Molecular subtypes of pancreatic cancer. Nat Rev Gastroenterol Hepatol. 2019;16(4):207-220. doi:10.1038/s41575-019-0109-y

3. Catenacci DVT, Junttila MR, Karrison T, et al. Randomized Phase Ib/II Study of Gemcitabine Plus Placebo or Vismodegib, a Hedgehog Pathway Inhibitor, in Patients With Metastatic Pancreatic Cancer. J Clin Oncol. 2015;33(36):4284-4292. doi:10.1200/JCO.2015.62.8719
