# Supplementary material for: LncRNA MACC1-AS1 induces gemcitabine resistance in pancreatic cancer cells through suppressing ferroptosis
Source: Cell Death Discov. 2024 Feb 27;10:101. doi: 10.1038/s41420-024-01866-y (PMC10899202; doi:10.1038/s41420-024-01866-y)
Supplement: Supplementary file 6 — WB Original data [file 41420_2024_1866_MOESM6_ESM.pdf]

# Western Blots Original Data

**LncRNA MACC1-AS1 induces gemcitabine resistance in pancreatic cancer cells through suppressing ferroptosis**

# Figure 1

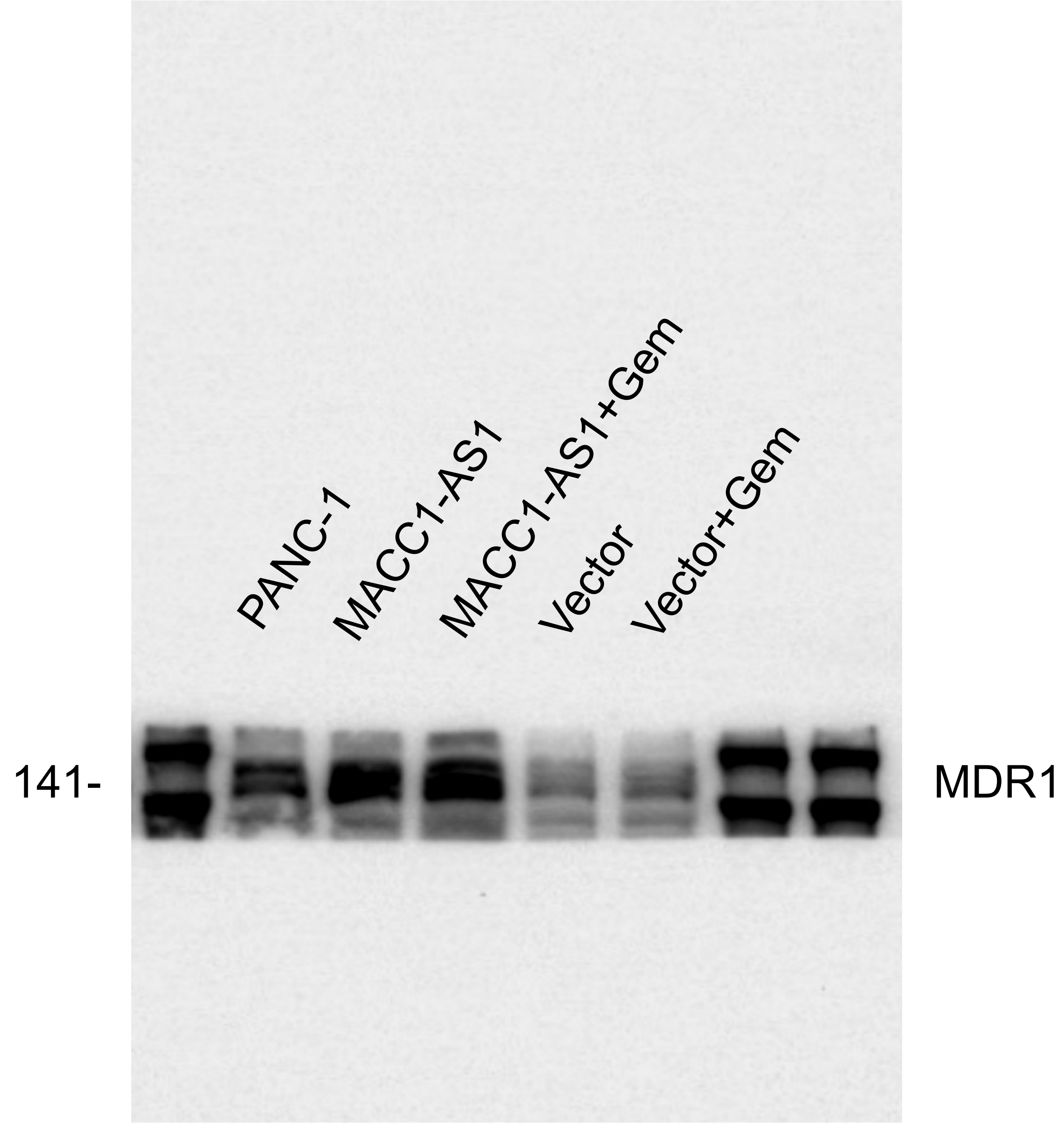
PANC-1
MACC1-AS1
MACC1-AS1+Gem
Vector
Vector+Gem
141-
MDR1

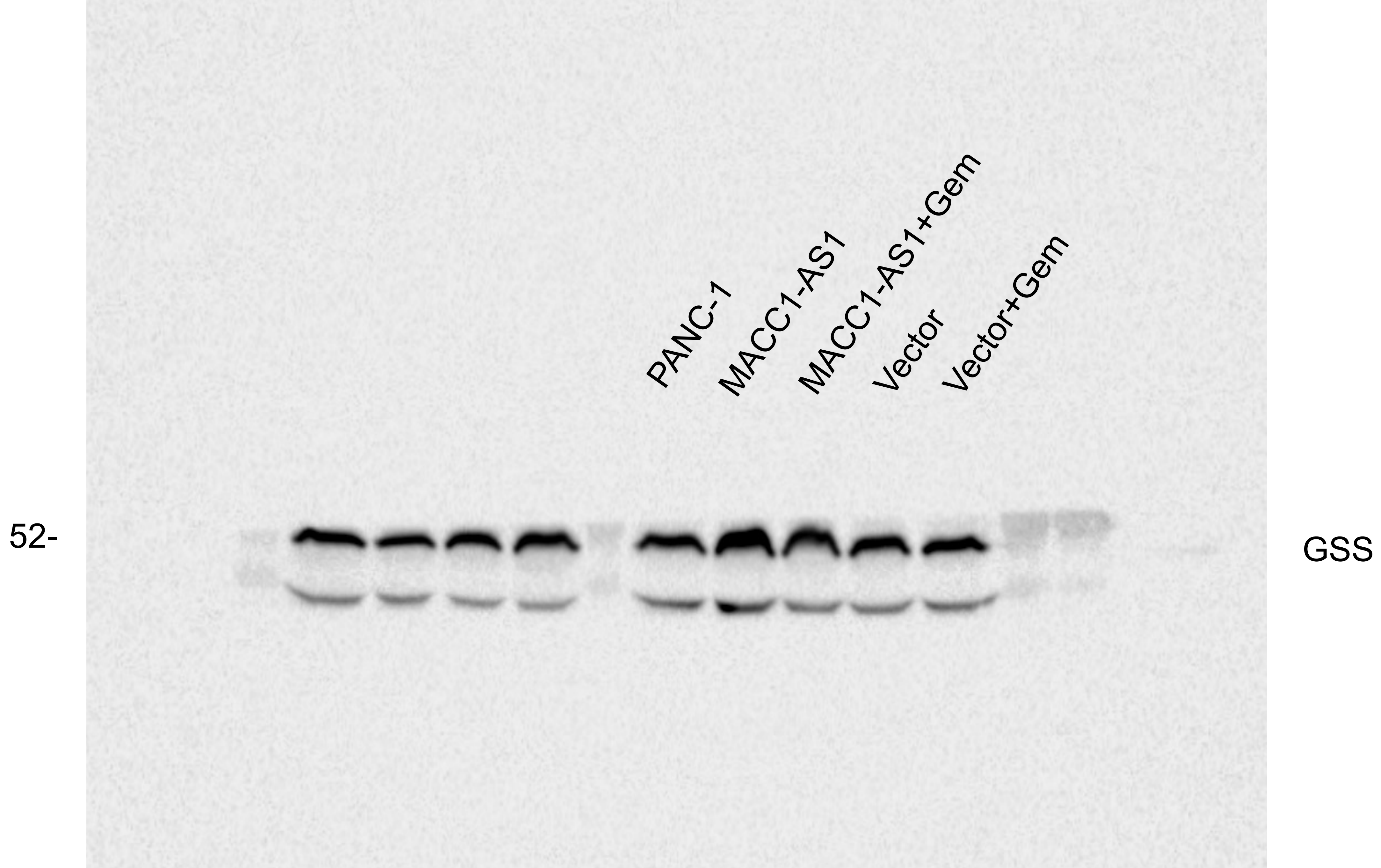
52-
PANC-1
MACC1-AS1
MACC1-AS1+Gem
Vector
Vector+Gem
GSS

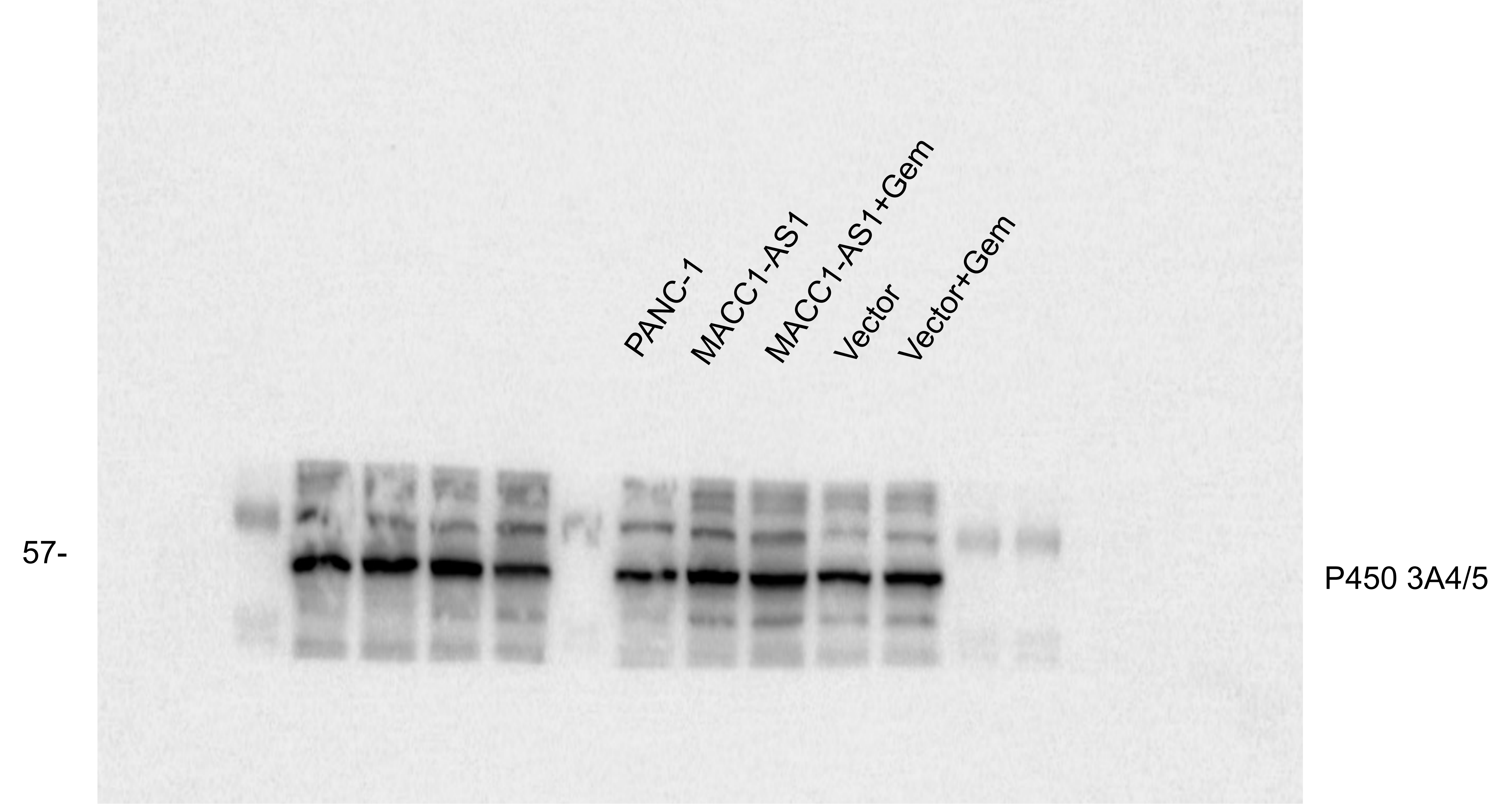
PANC-1
MACC1-AS1
MACC1-AS1+Gem
Vector
Vector+Gem
57-
P450 3A4/5

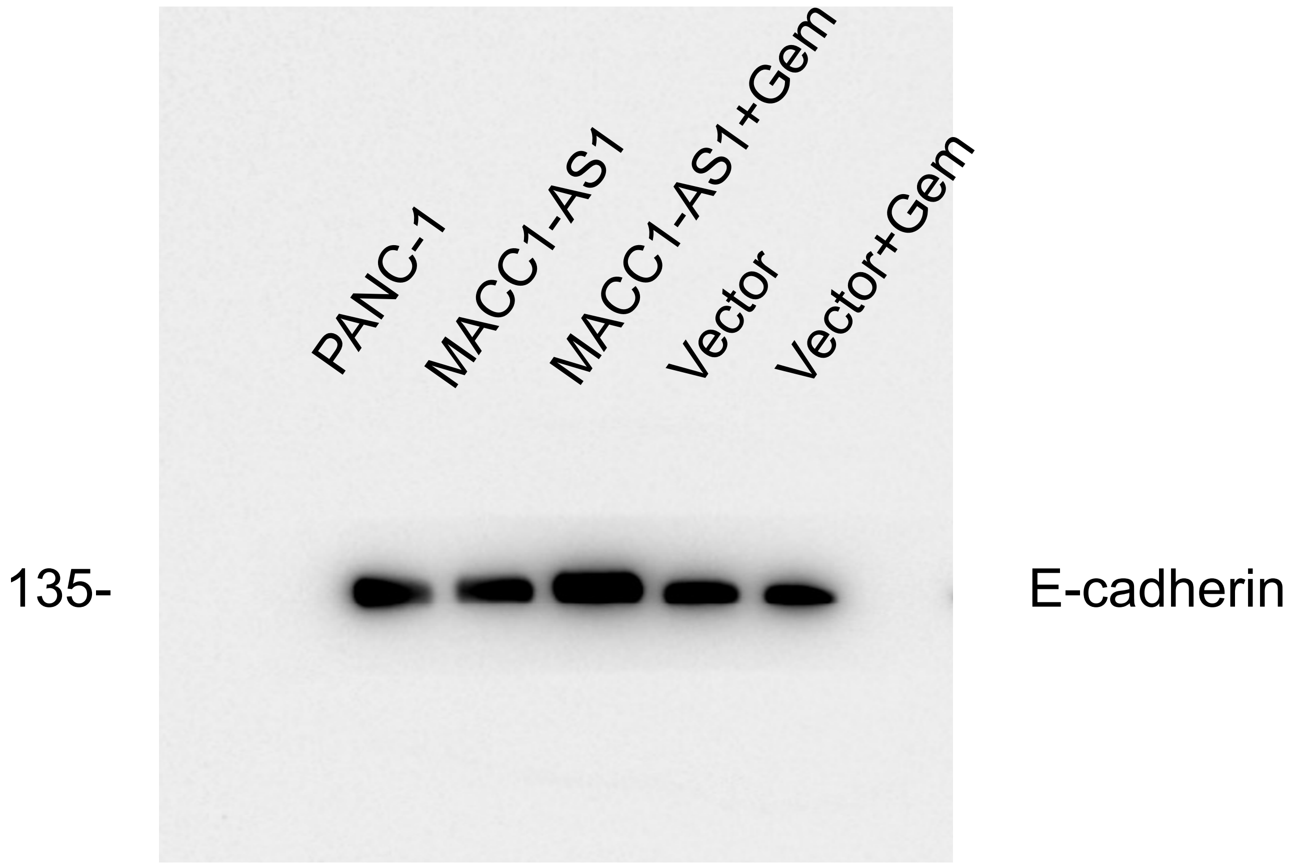
PANC-1
MACC1-AS1
MACC1-AS1+Gem
Vector
Vector+Gem
135-
E-cadherin

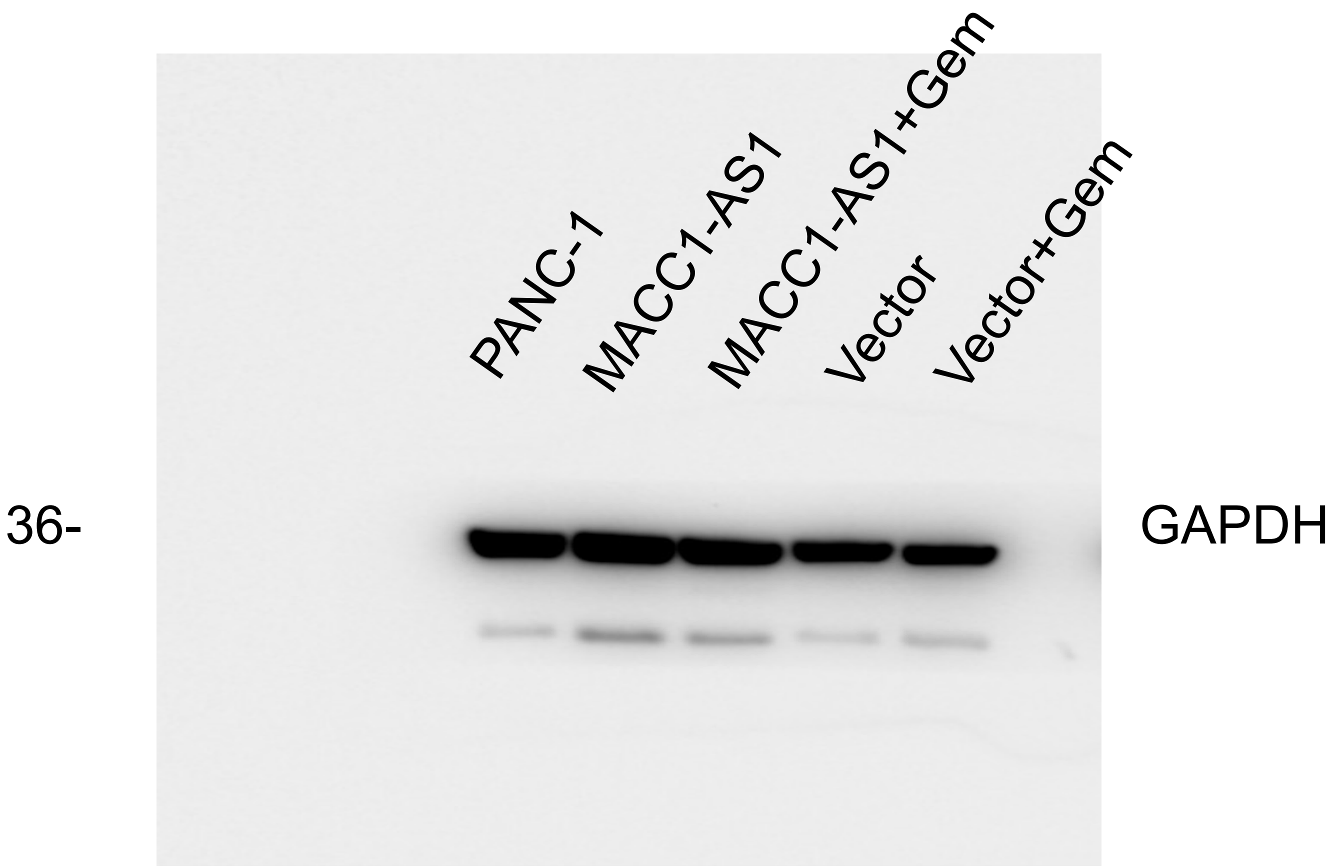
PANC-1
MACC1-AS1
MACC1-AS1+Gem
Vector
Vector+Gem
36-
GAPDH

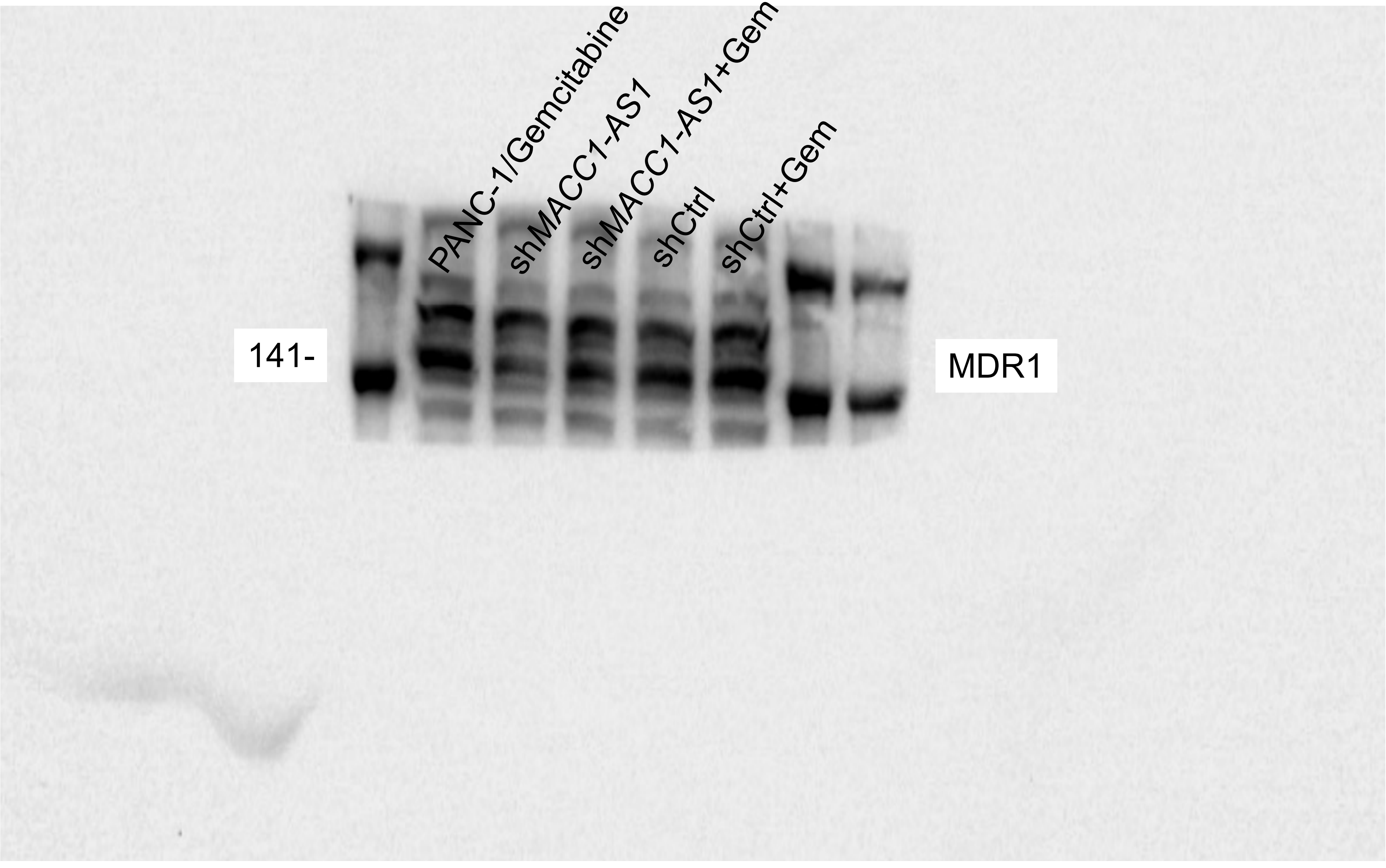
PANC-1/Gemcitabine
shMACC1-AS1
shMACC1-AS1+Gem
shCtrl
shCtrl+Gem
141-
MDR1

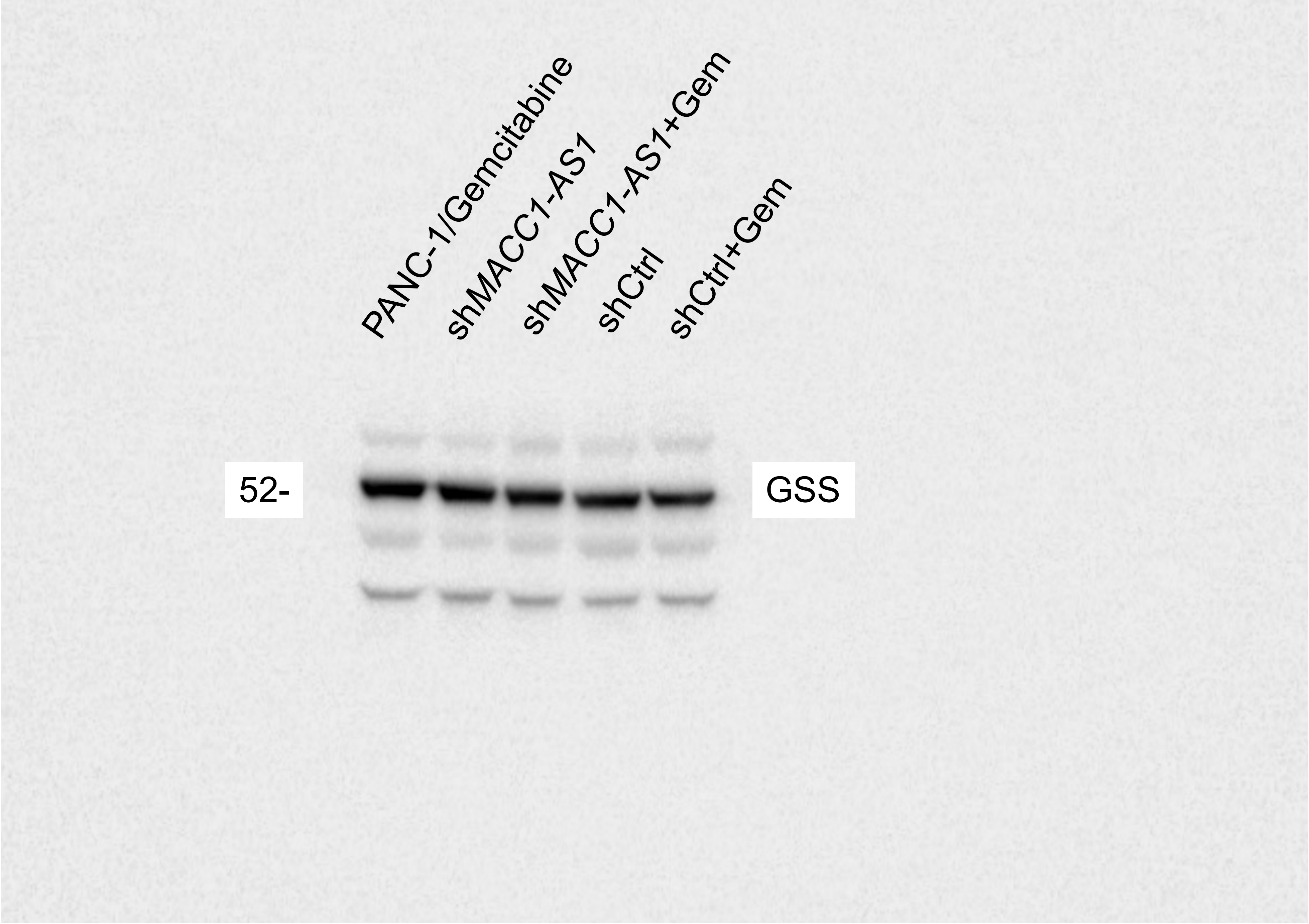
PANC-1/Gemcitabine
shMACC1-AS1
shMACC1-AS1+Gem
shCtrl
shCtrl+Gem
52-
GSS

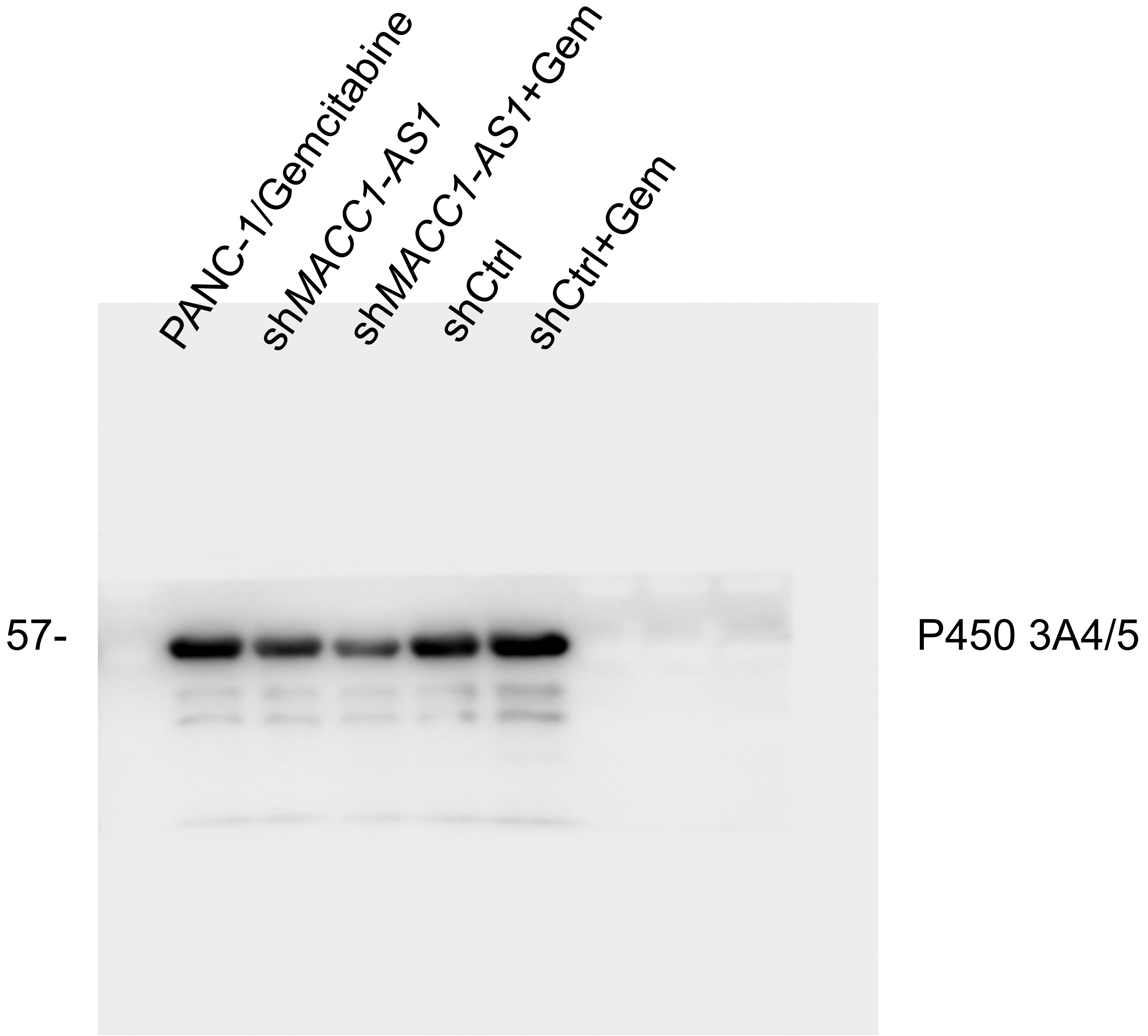

PANC-1/Gemcitabine
shMACC1-AS1
shMACC1-AS1+Gem
shCtrl
shCtrl+Gem
57-
P450 3A4/5

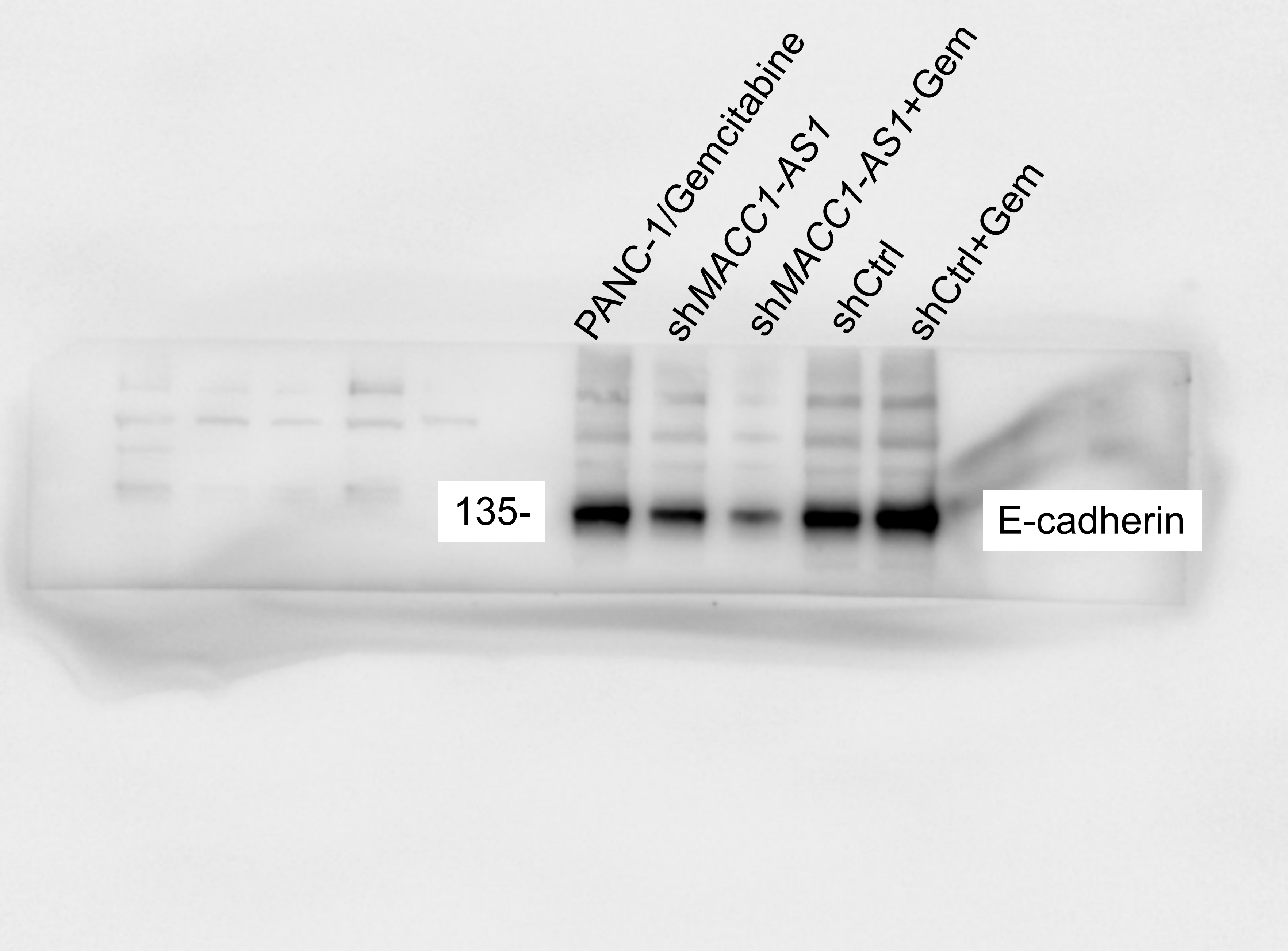
PANC-1/Gemcitabine
shMACC1-AS1
shMACC1-AS1+Gem
shCtrl
shCtrl+Gem
135-
E-cadherin

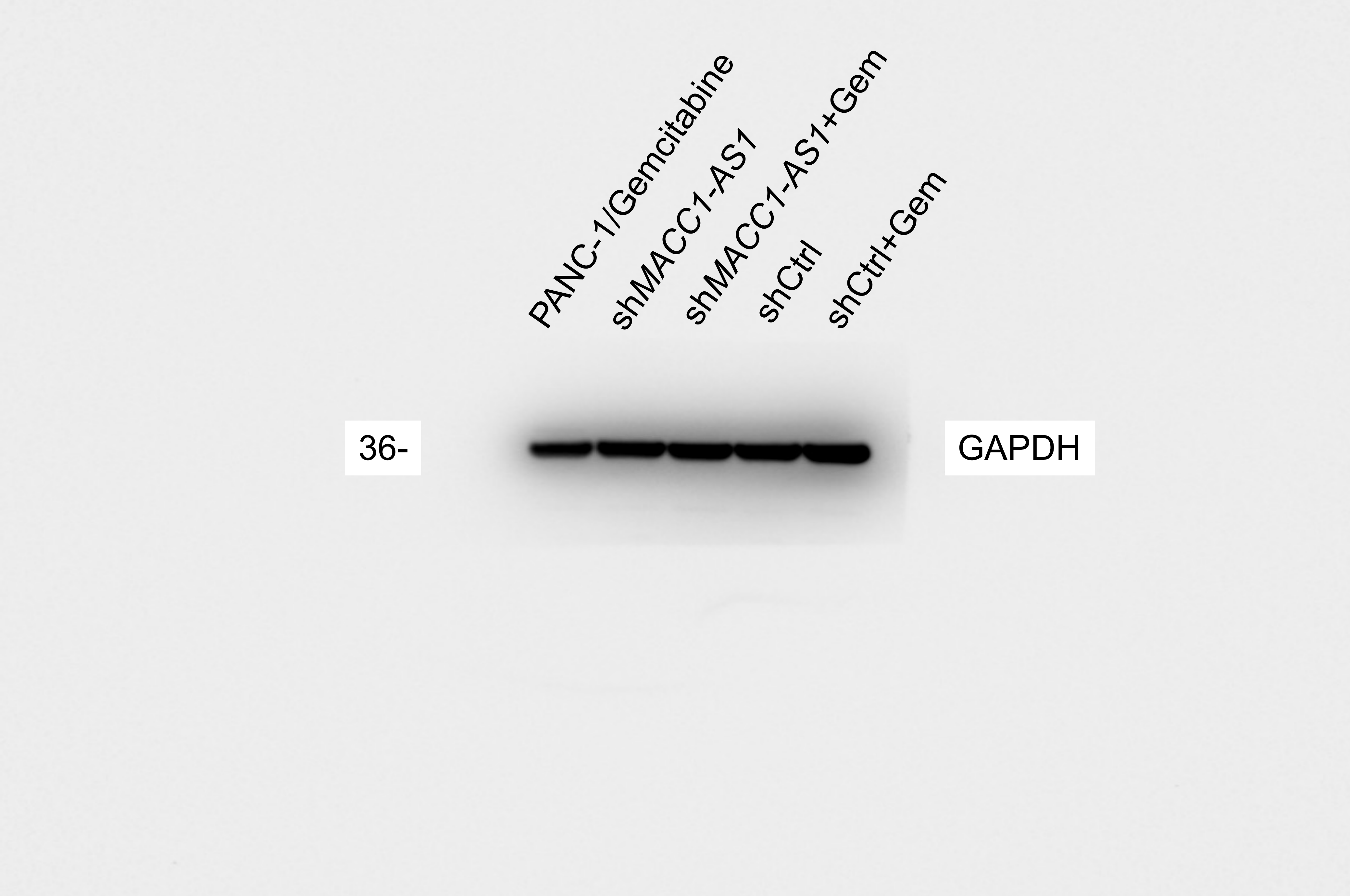
PANC-1/Gemcitabine
shMACC1-AS1
shMACC1-AS1+Gem
shCtrl
shCtrl+Gem
36-
GAPDH

# Figure 2

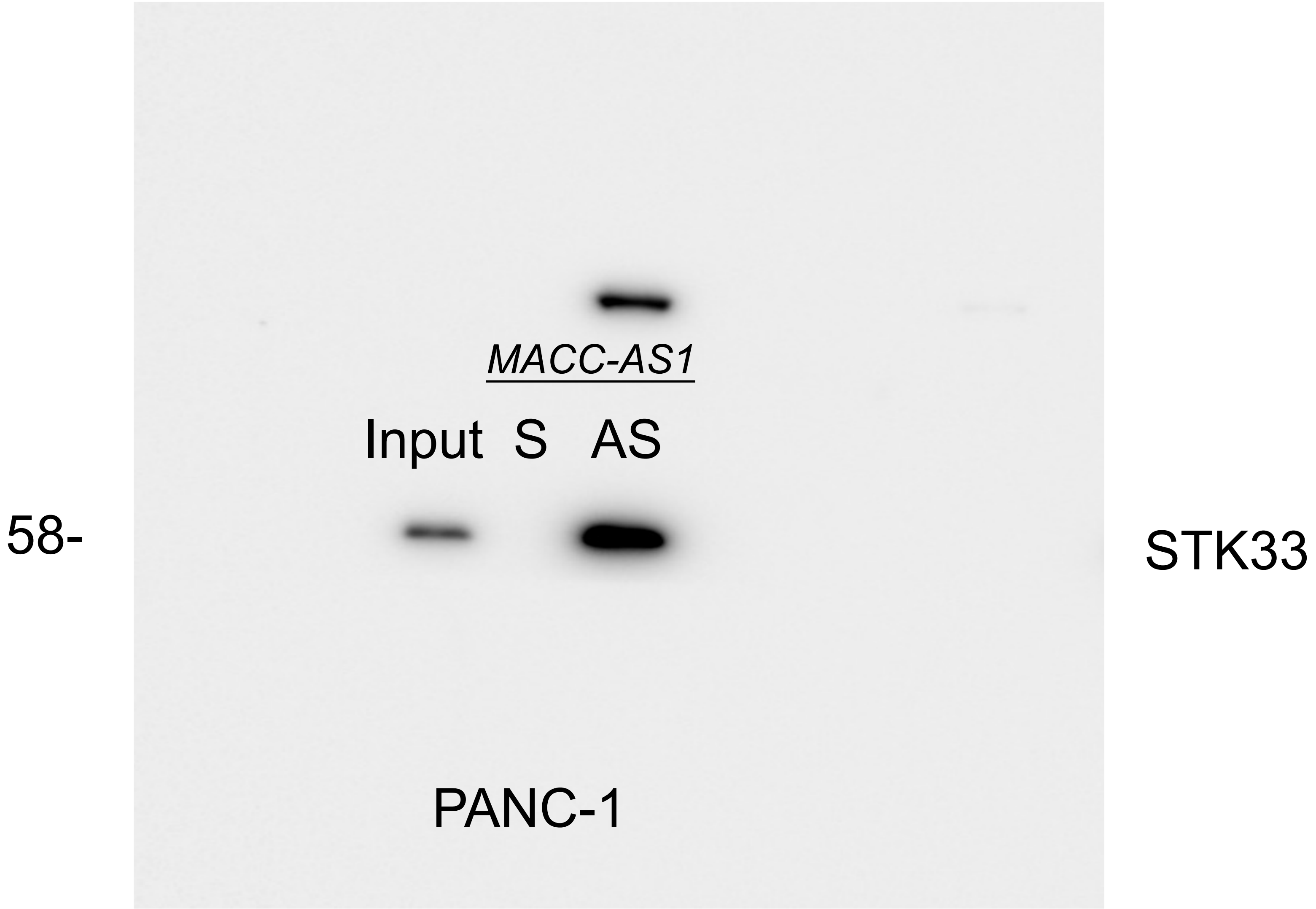
MACC-AS1
Input
S
AS
58-
STK33
PANC-1

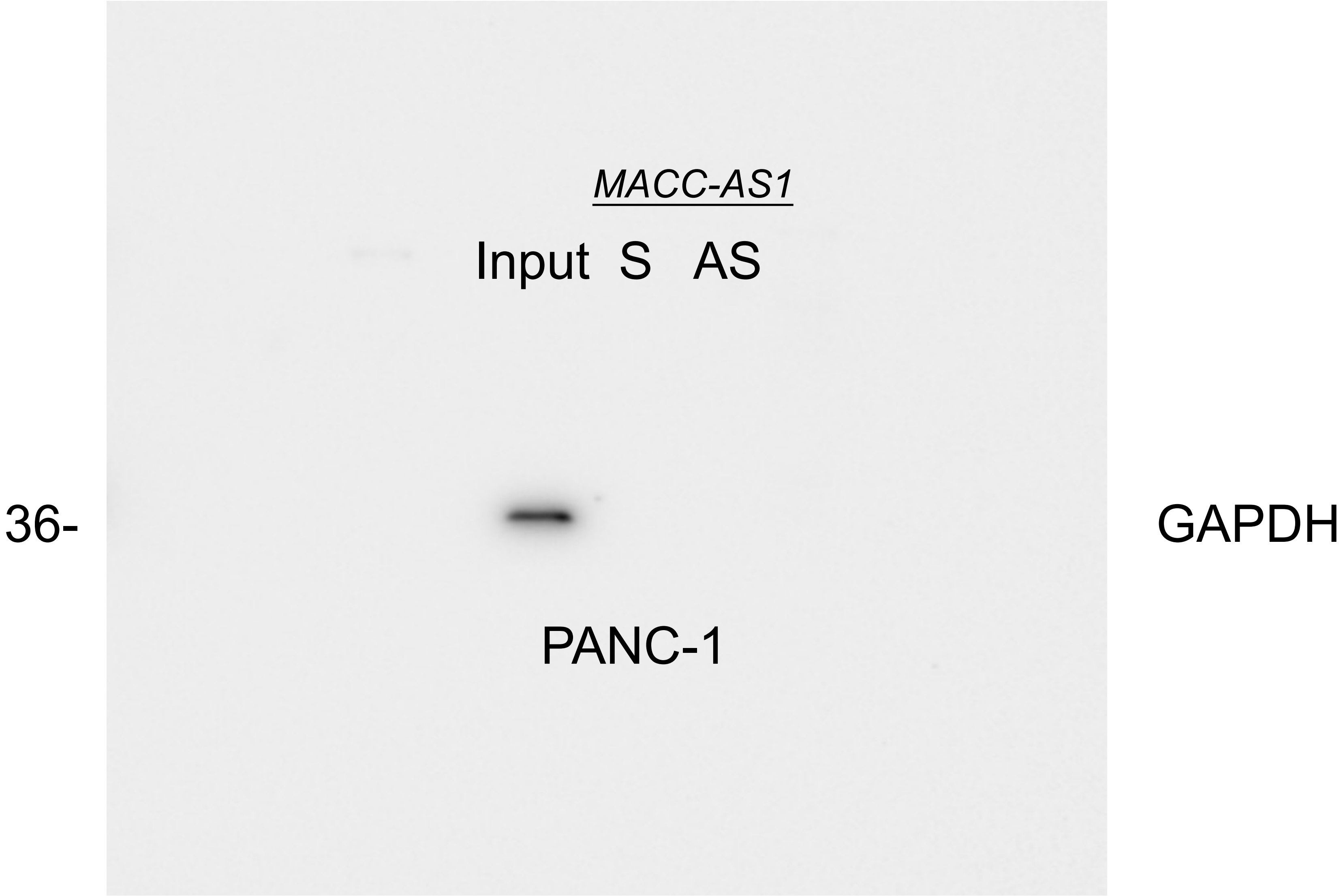
MACC-AS1
Input S AS
36-
GAPDH
PANC-1

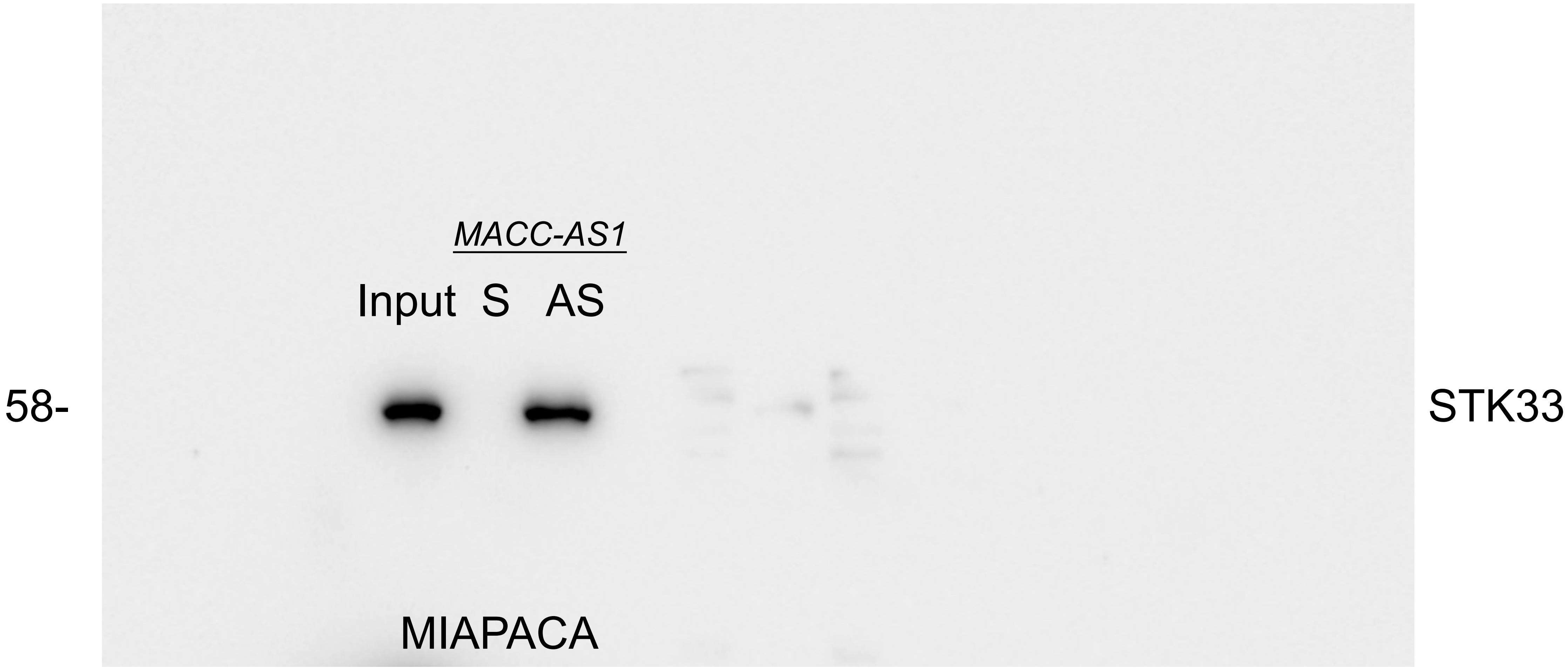

MACC-AS1
Input
S
AS
58-
STK33
MIAPACA

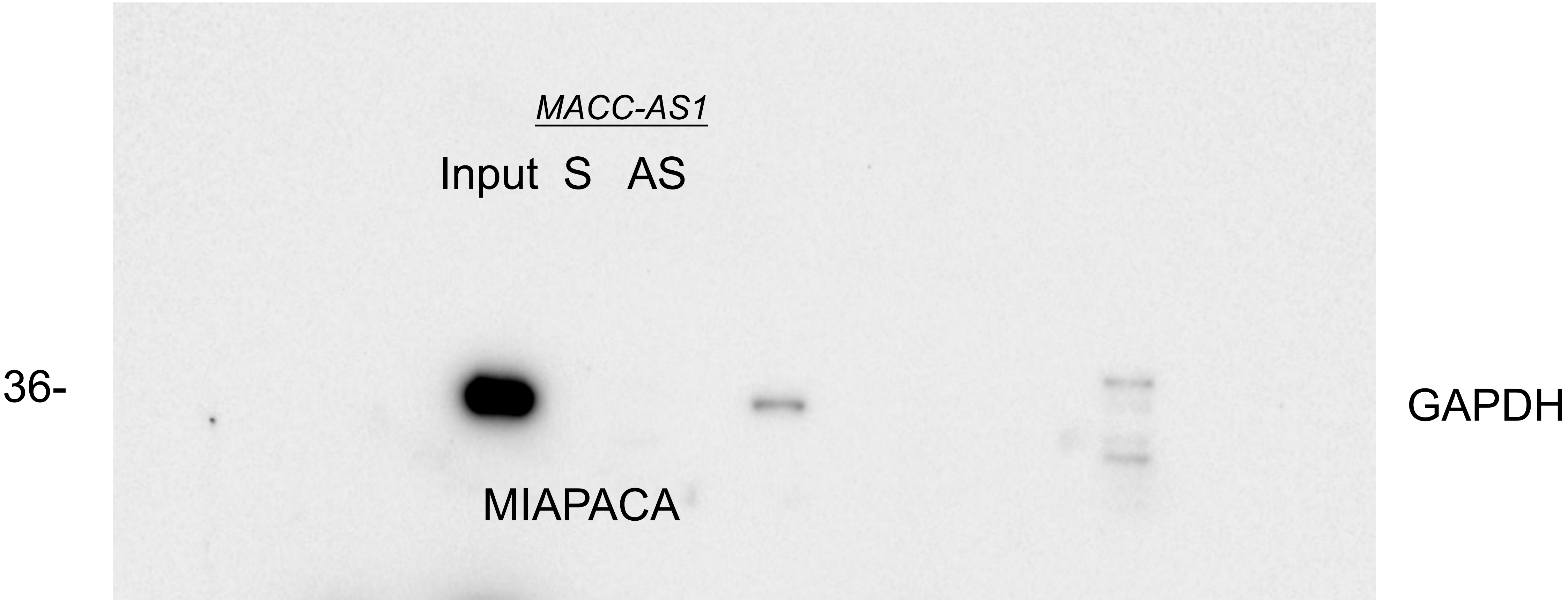
MACC-AS1
Input S AS
36-
GAPDH
MIAPACA

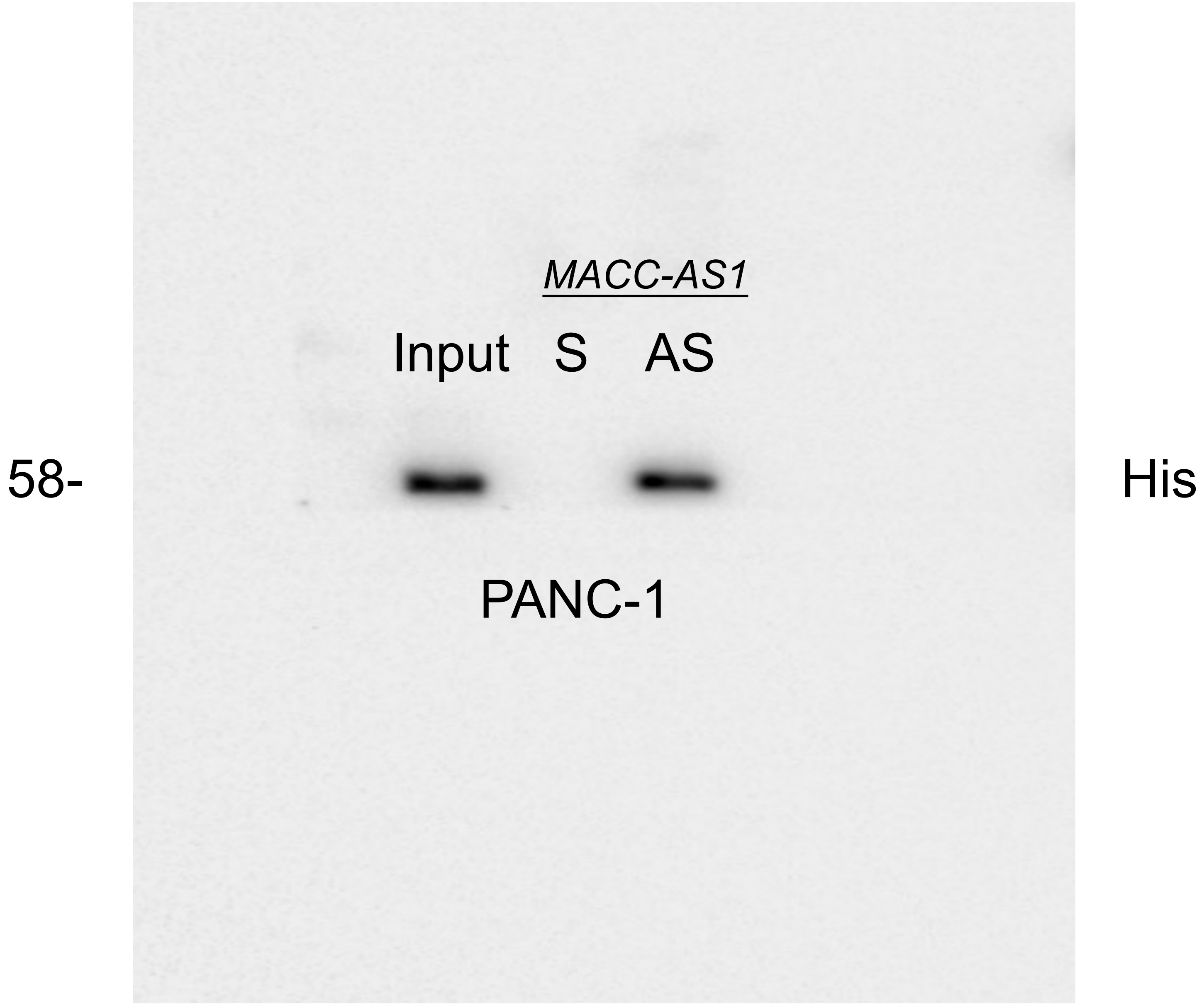
MACC-AS1
Input
S
AS
58-
His
PANC-1

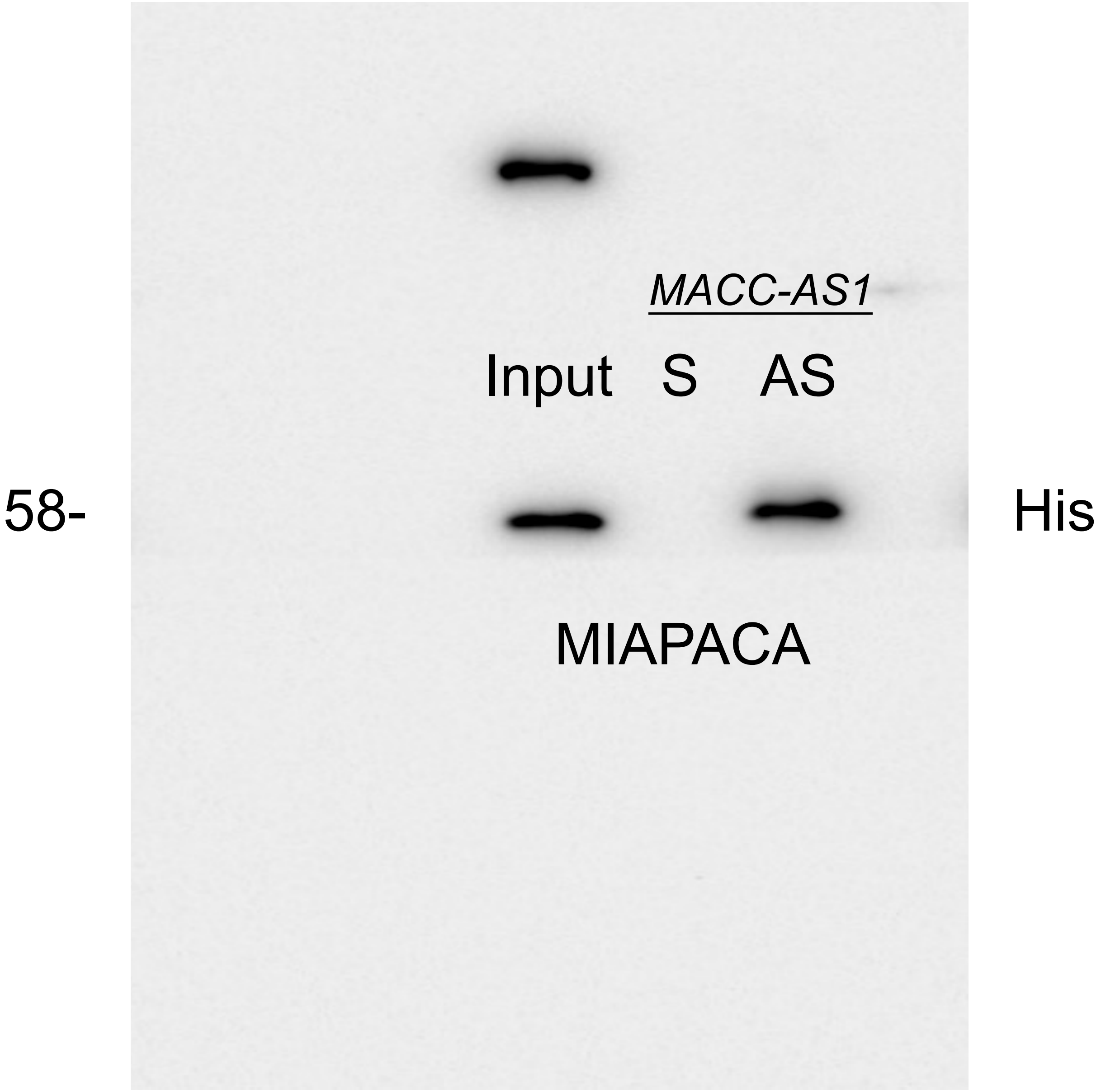

MACC-AS1
Input
S
AS
58-
His
MIAPACA

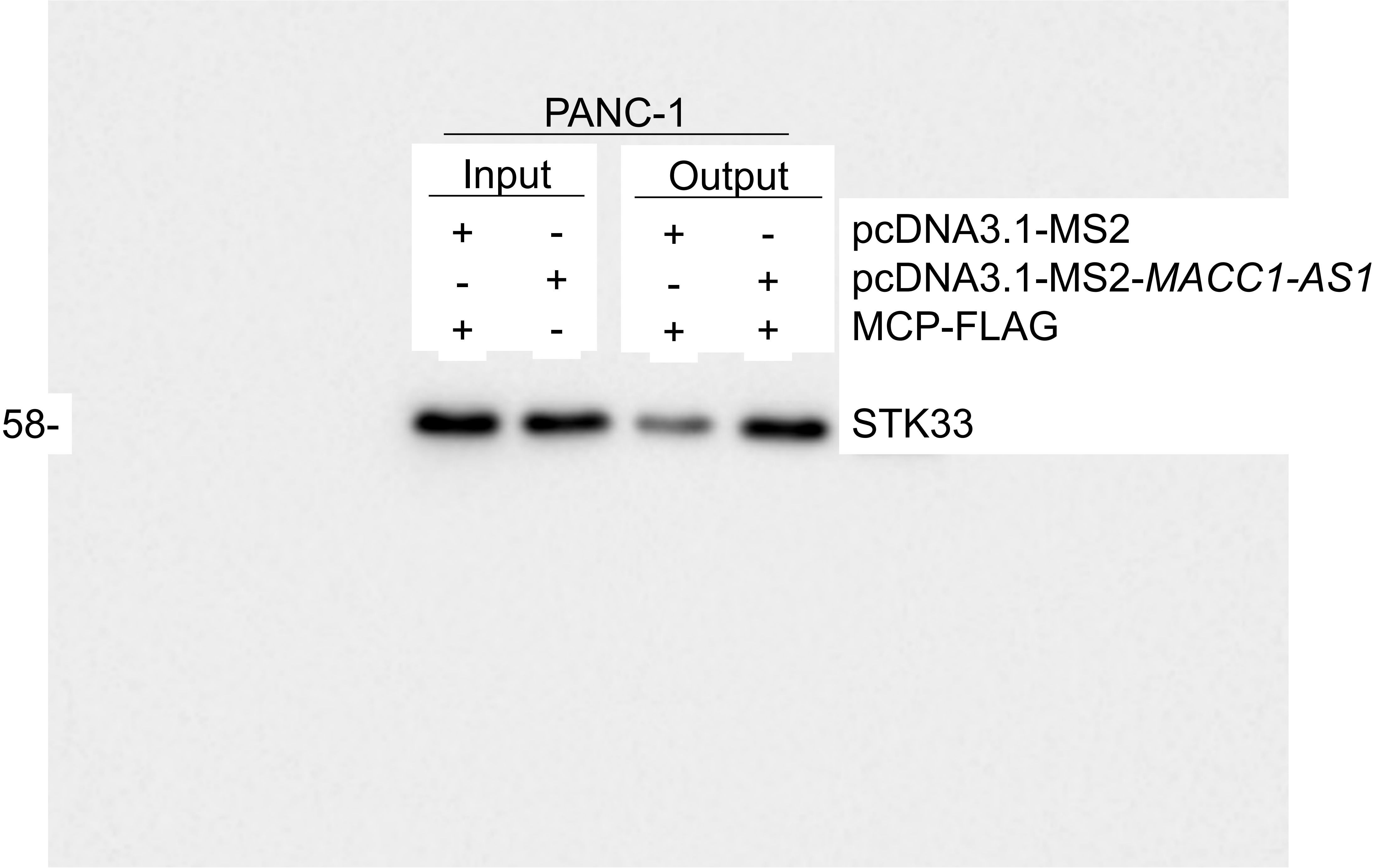
PANC-1
Input
Output
pcDNA3.1-MS2 + - + -
pcDNA3.1-MS2-*MACC1-AS1* - + - +
MCP-FLAG + - + +
58-
STK33

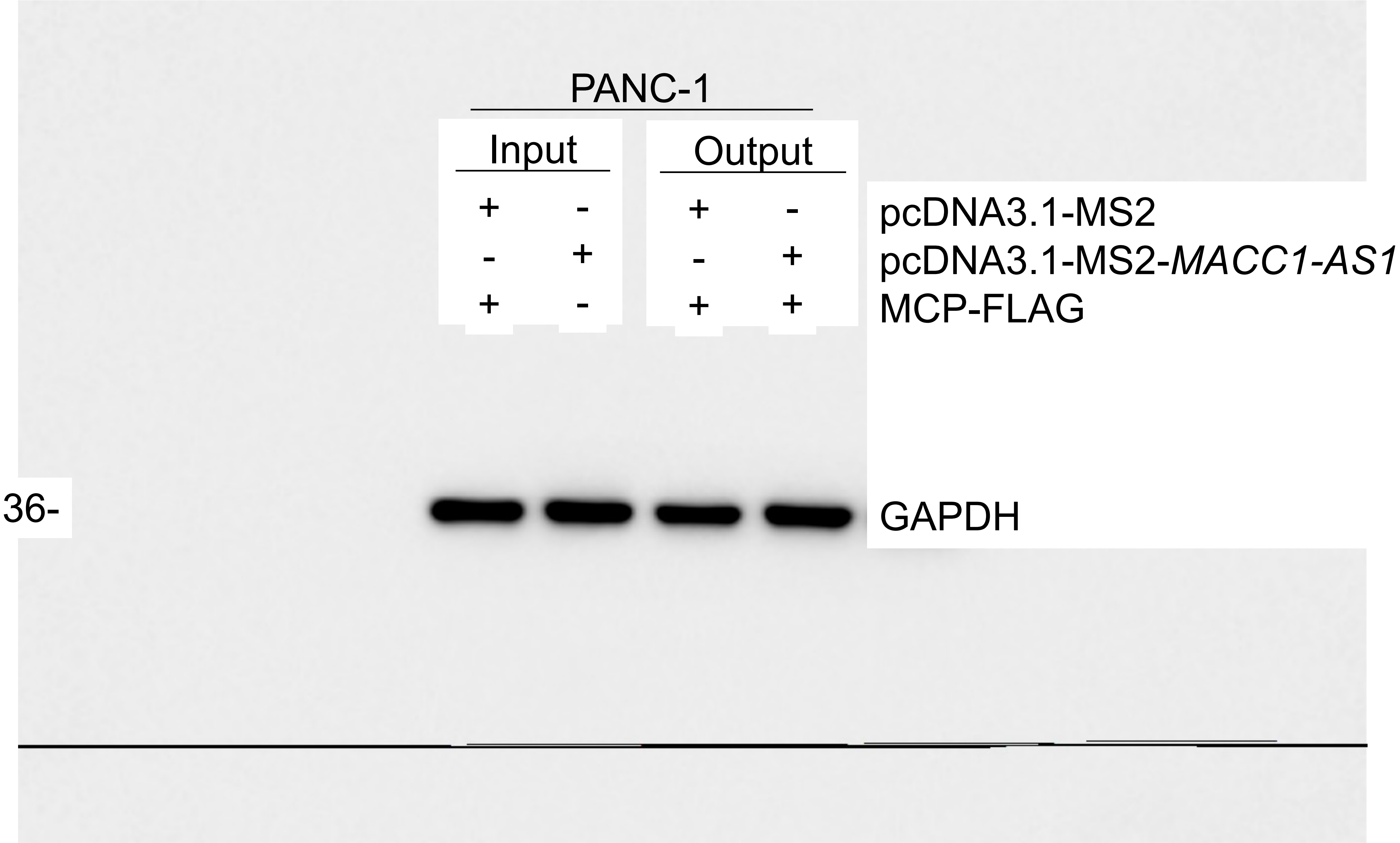
PANC-1
Input
Output
+ - + -
- + - +
+ - + +
pcDNA3.1-MS2
pcDNA3.1-MS2-*MACC1-AS1*
MCP-FLAG
36-
GAPDH

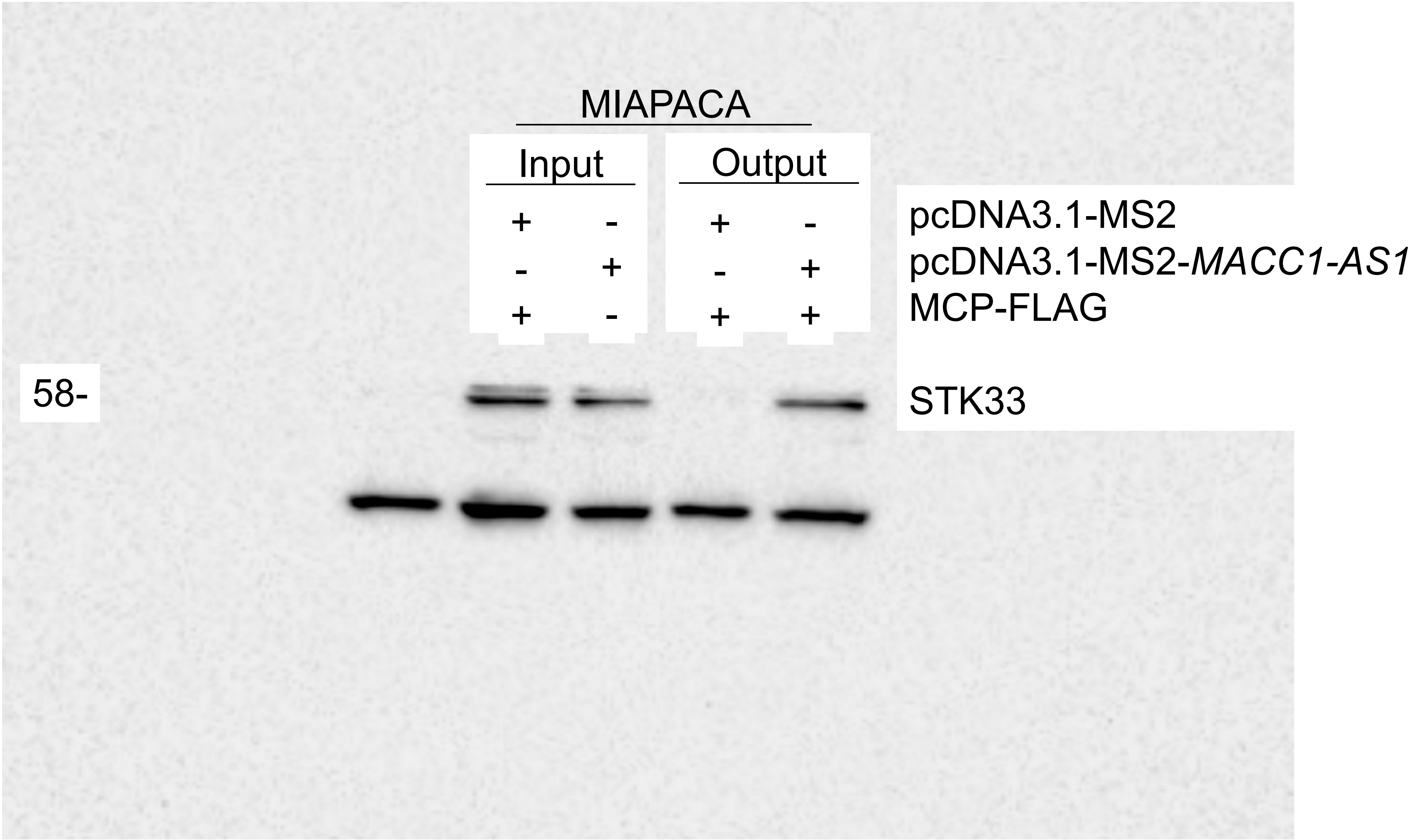
MIAPACA
Input
Output
pcDNA3.1-MS2
pcDNA3.1-MS2-*MACC1-AS1*
MCP-FLAG
+ - + -
- + - +
+ - + +
58-
STK33

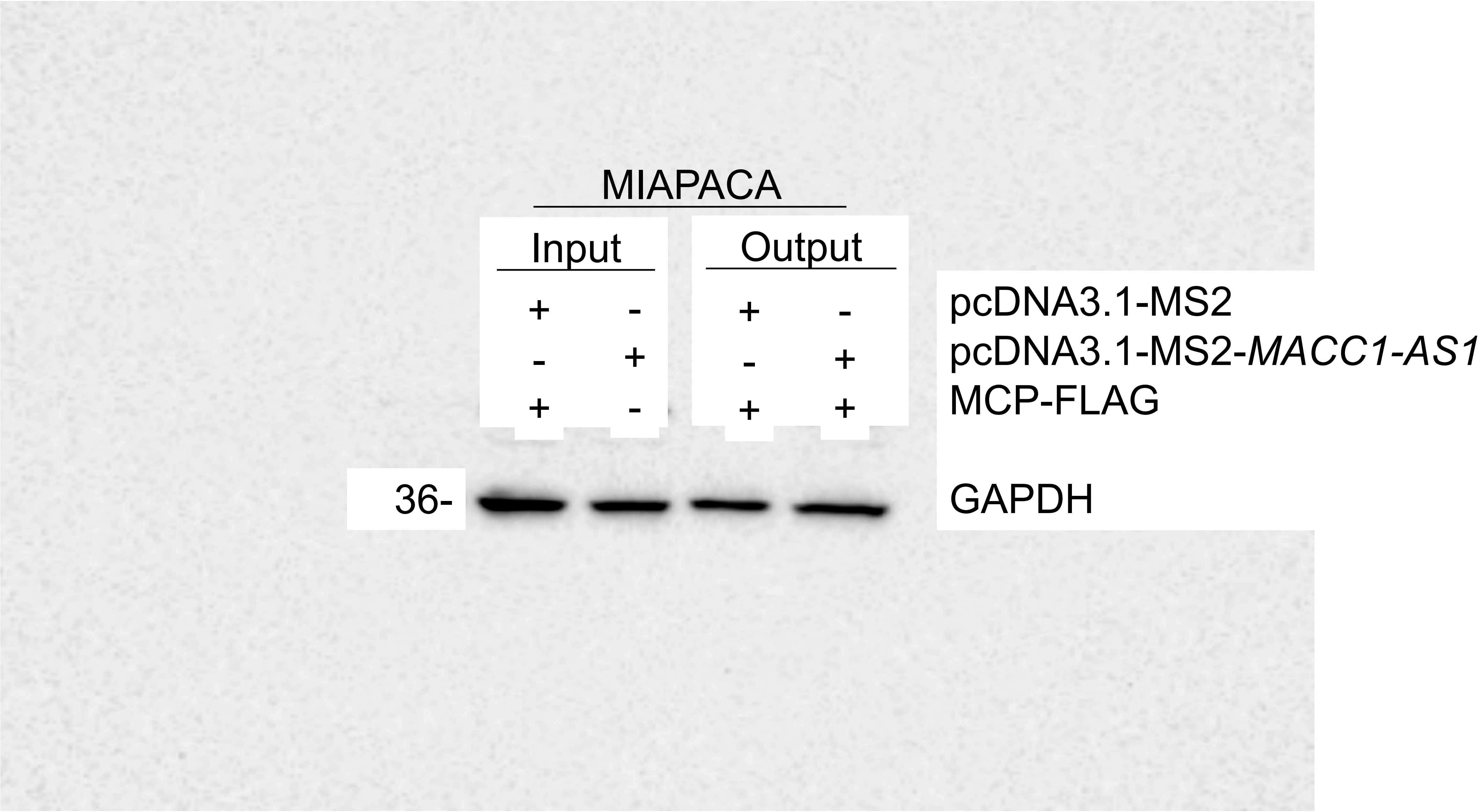
MIAPACA
Input
Output
pcDNA3.1-MS2
pcDNA3.1-MS2-*MACC1-AS1*
MCP-FLAG
+ - + -
- + - +
+ - + +
36-
GAPDH

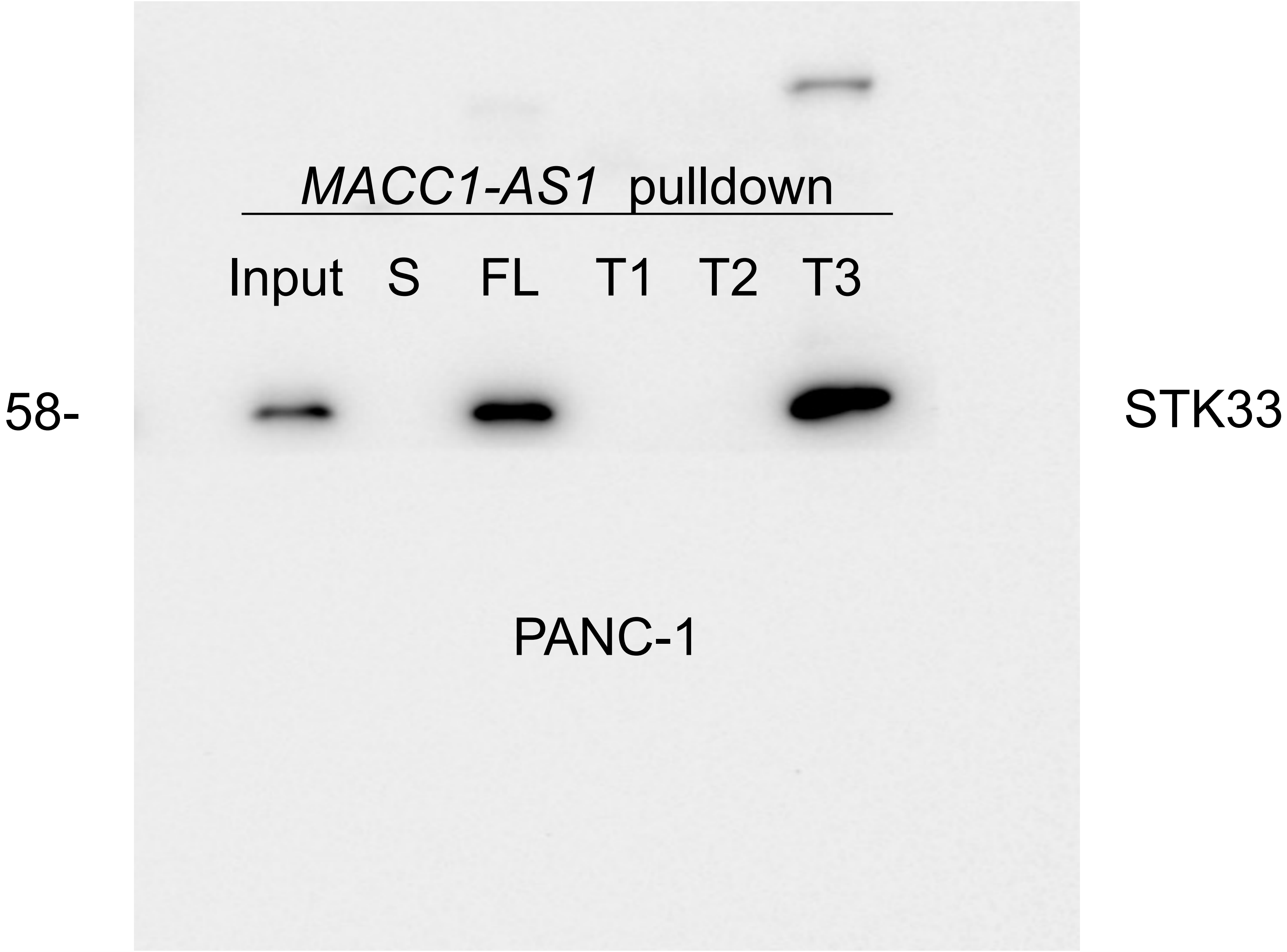
*MACC1-AS1* pulldown
Input
S
FL
T1
T2
T3
58-
STK33
PANC-1

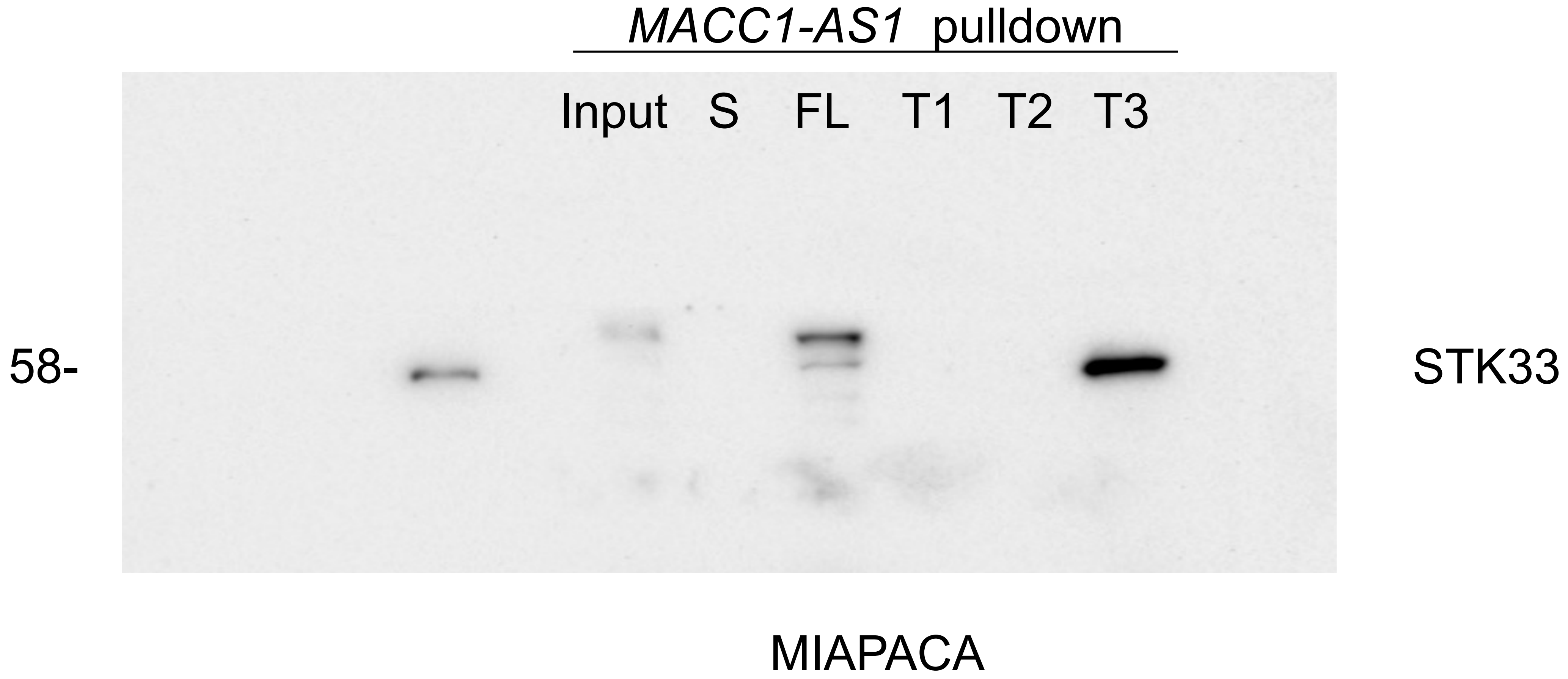
MACC1-AS1 pulldown
Input
S
FL
T1
T2
T3
58-
STK33
MIAPACA

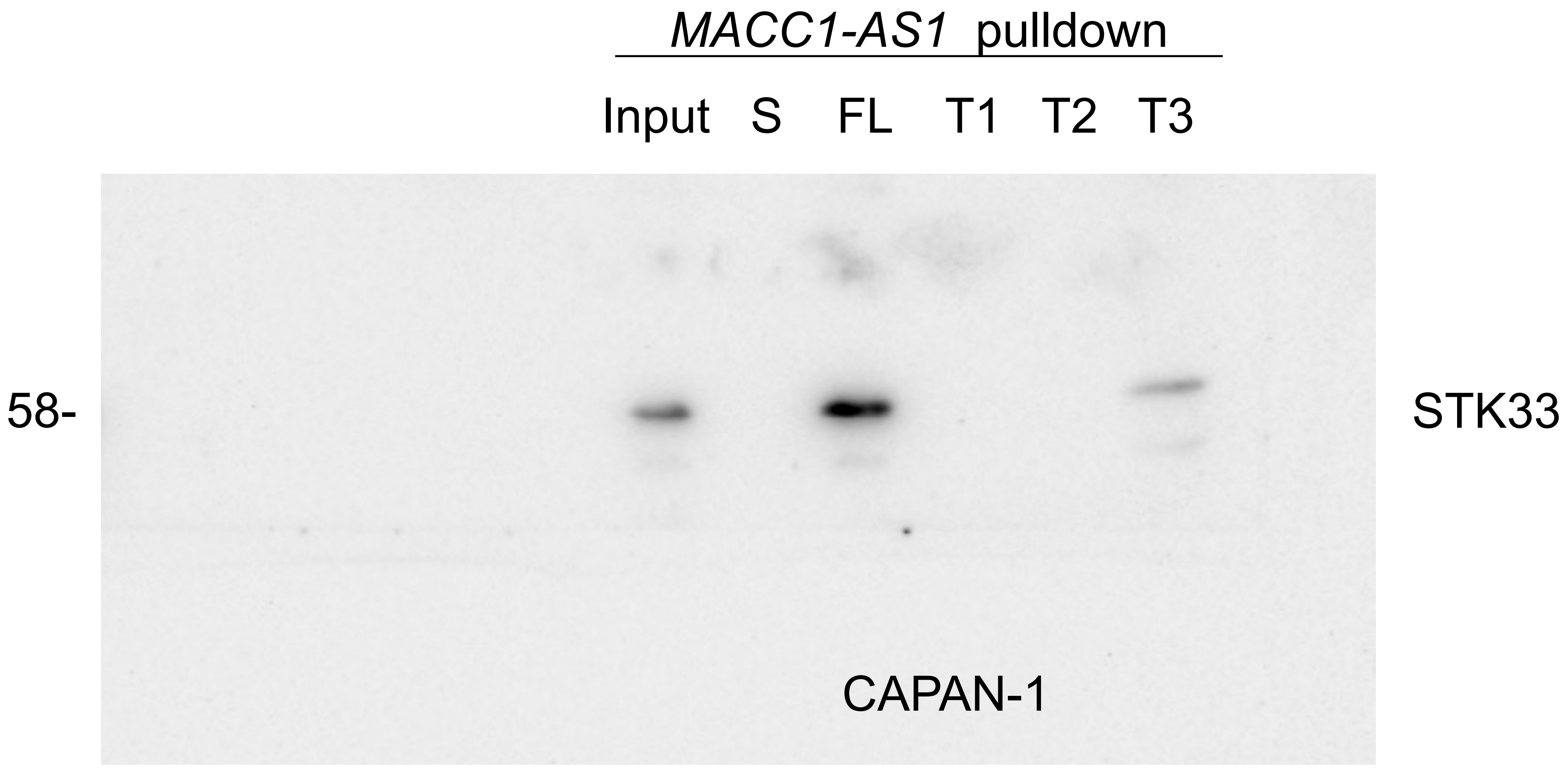
MACC1-AS1 pulldown
Input
S
FL
T1
T2
T3
58-
STK33
CAPAN-1

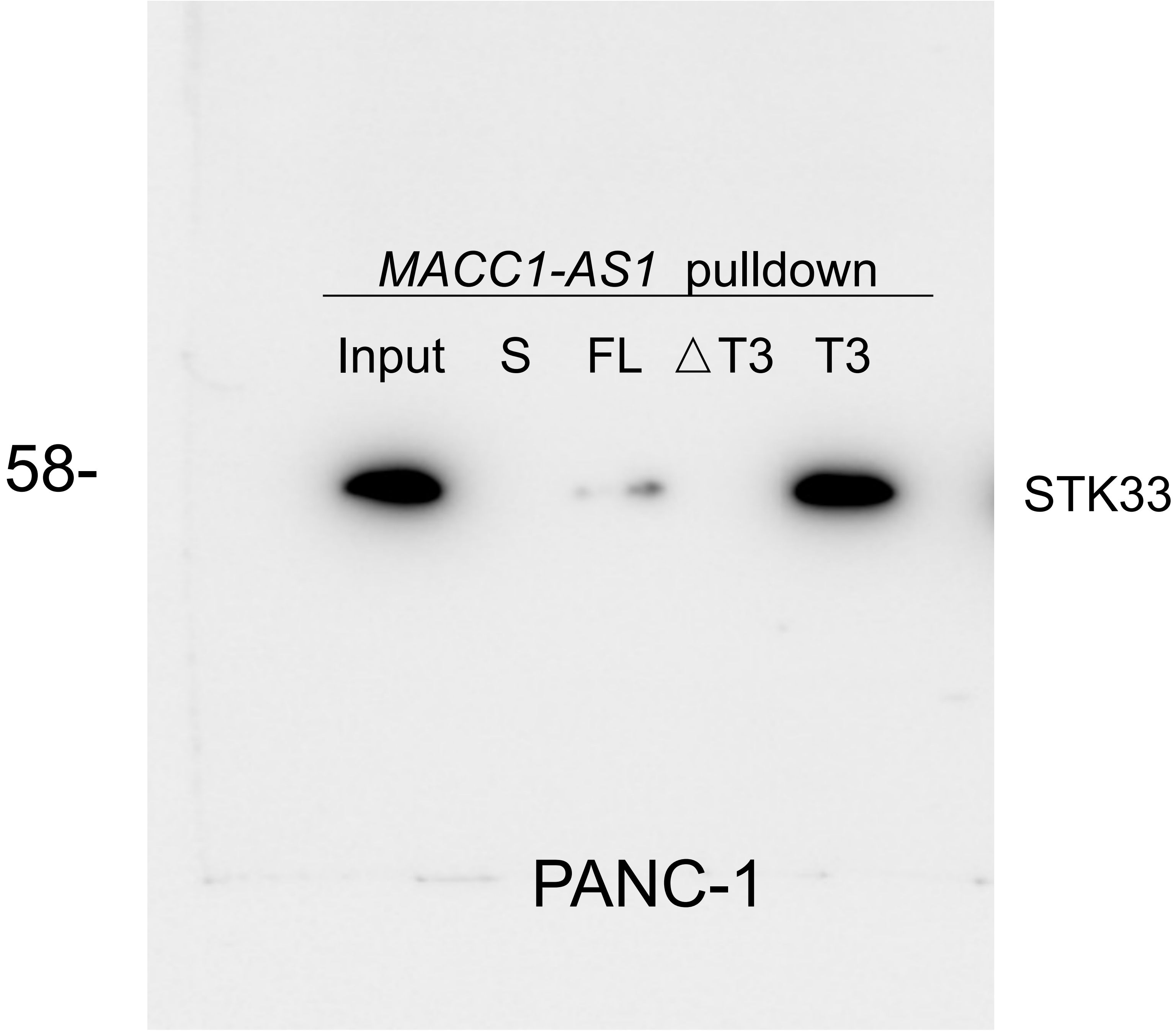
MACC1-AS1 pulldown
Input
S
FL
△T3
T3
58-
STK33
PANC-1

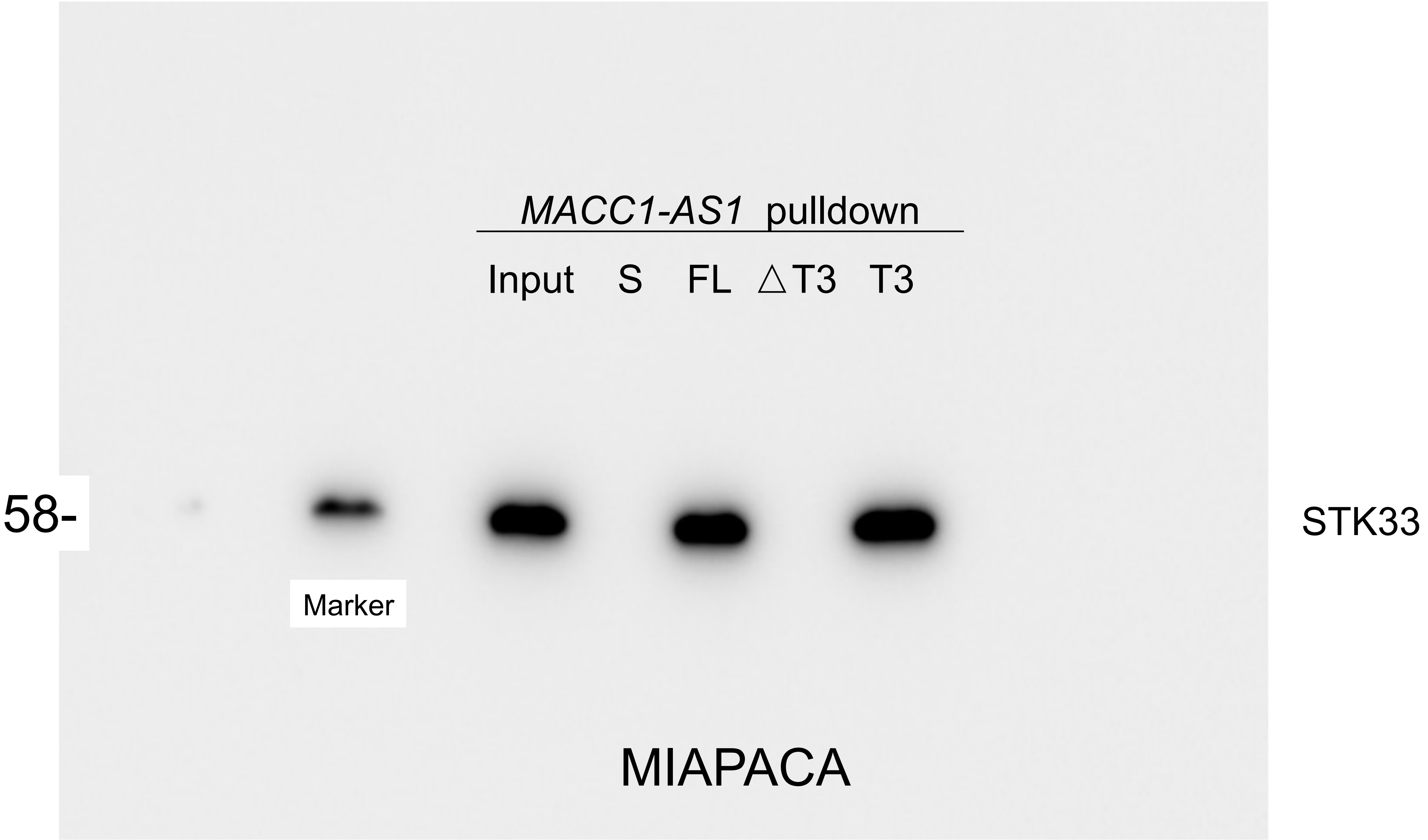

*MACC1-AS1* pulldown
Input
S
FL
△T3
T3
58-
Marker
STK33
MIAPACA

# Figure 3

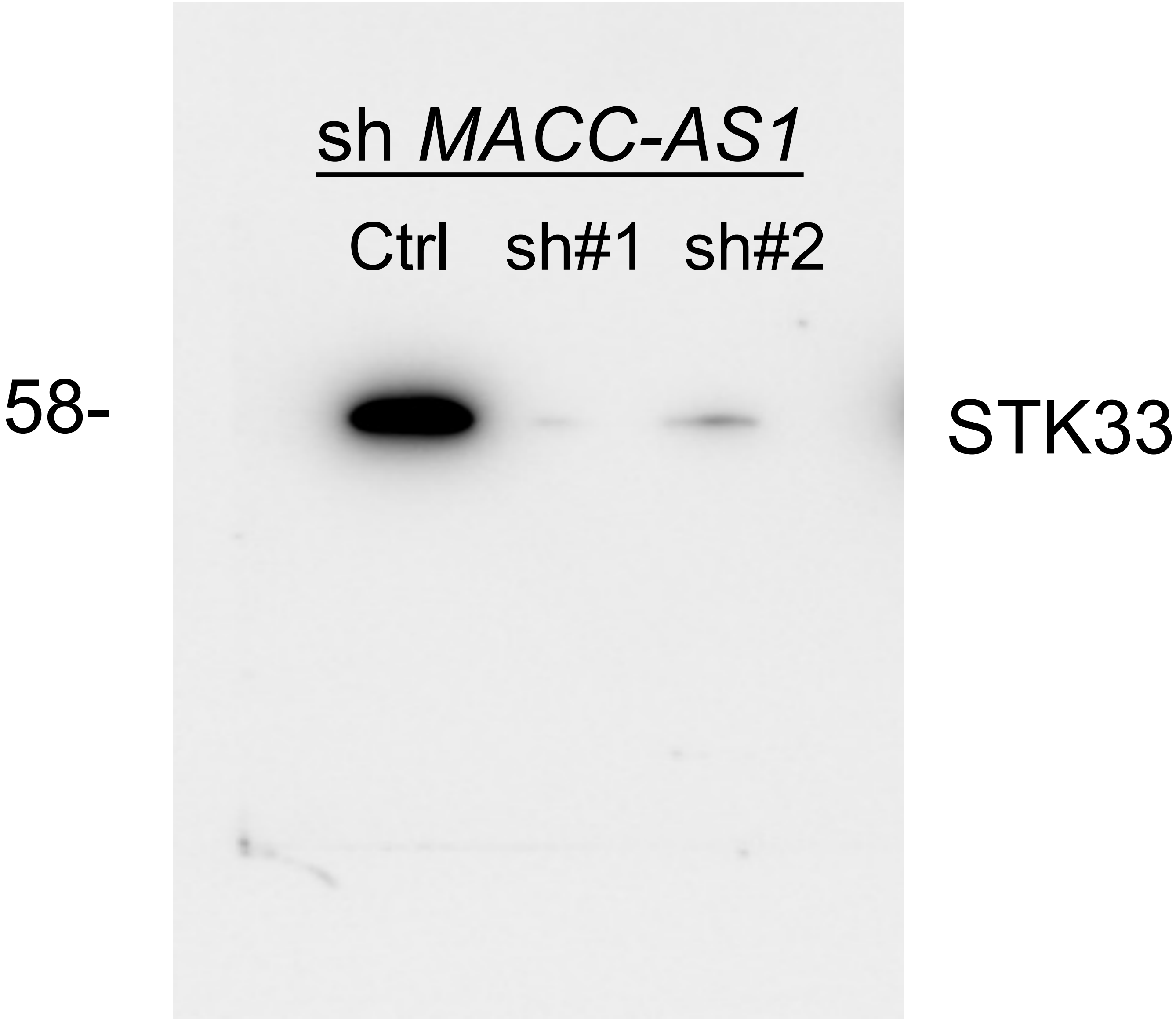
sh *MACC-AS1*
Ctrl
sh#1
sh#2
58-
STK33

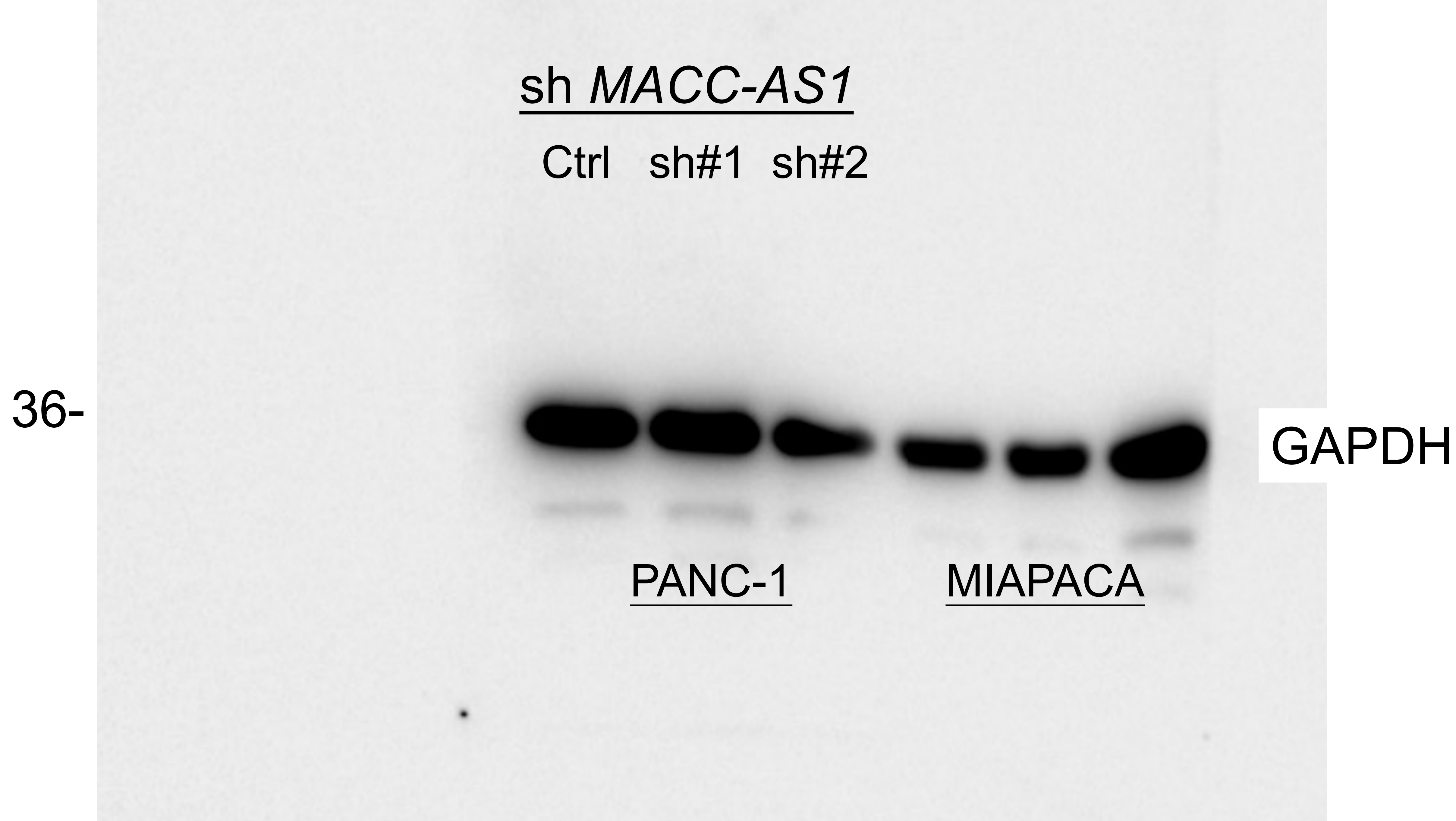
sh *MACC-AS1*
Ctrl sh#1 sh#2
36-
GAPDH
PANC-1
MIAPACA

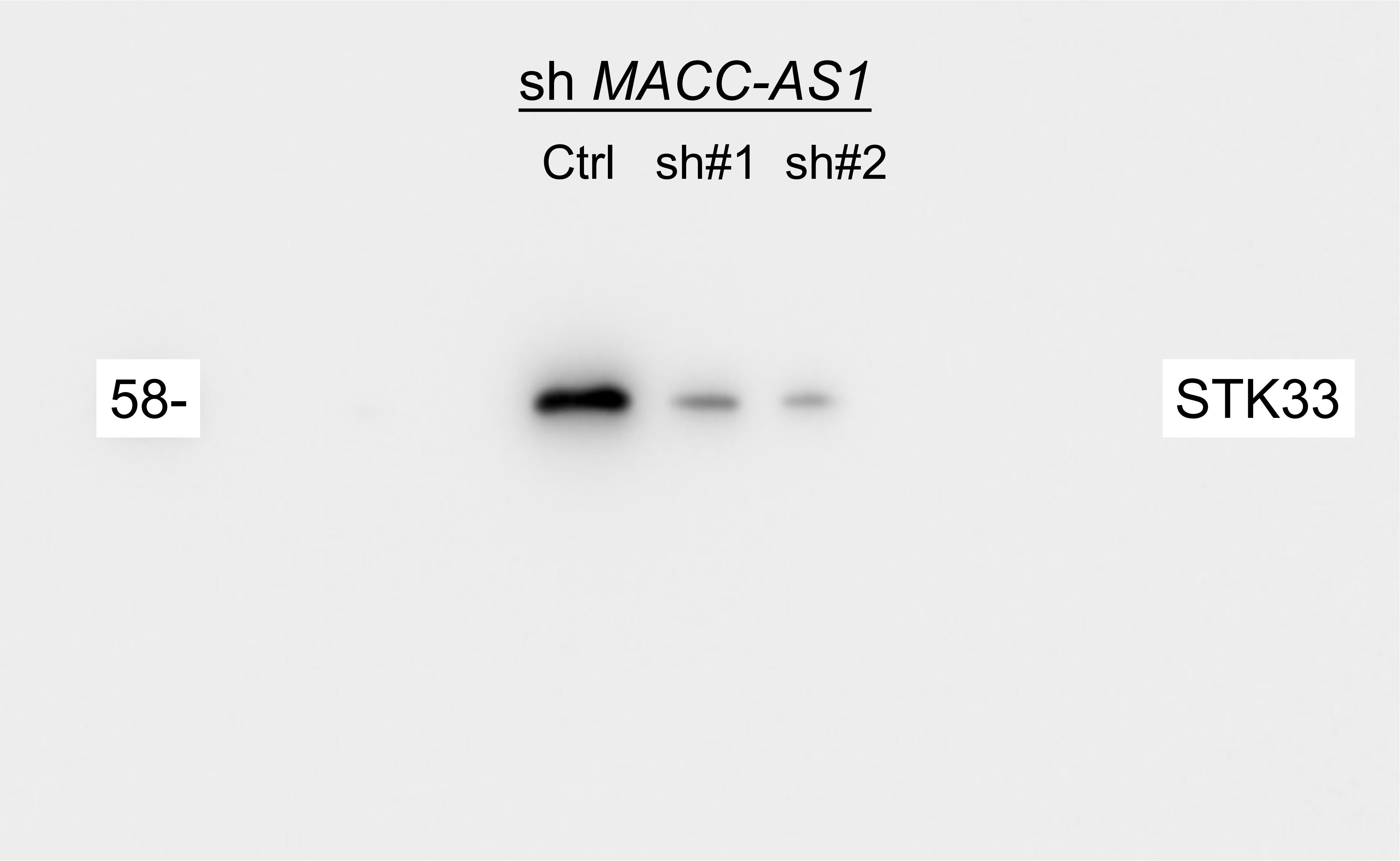
sh *MACC-AS1*
Ctrl
sh#1
sh#2
58-
STK33

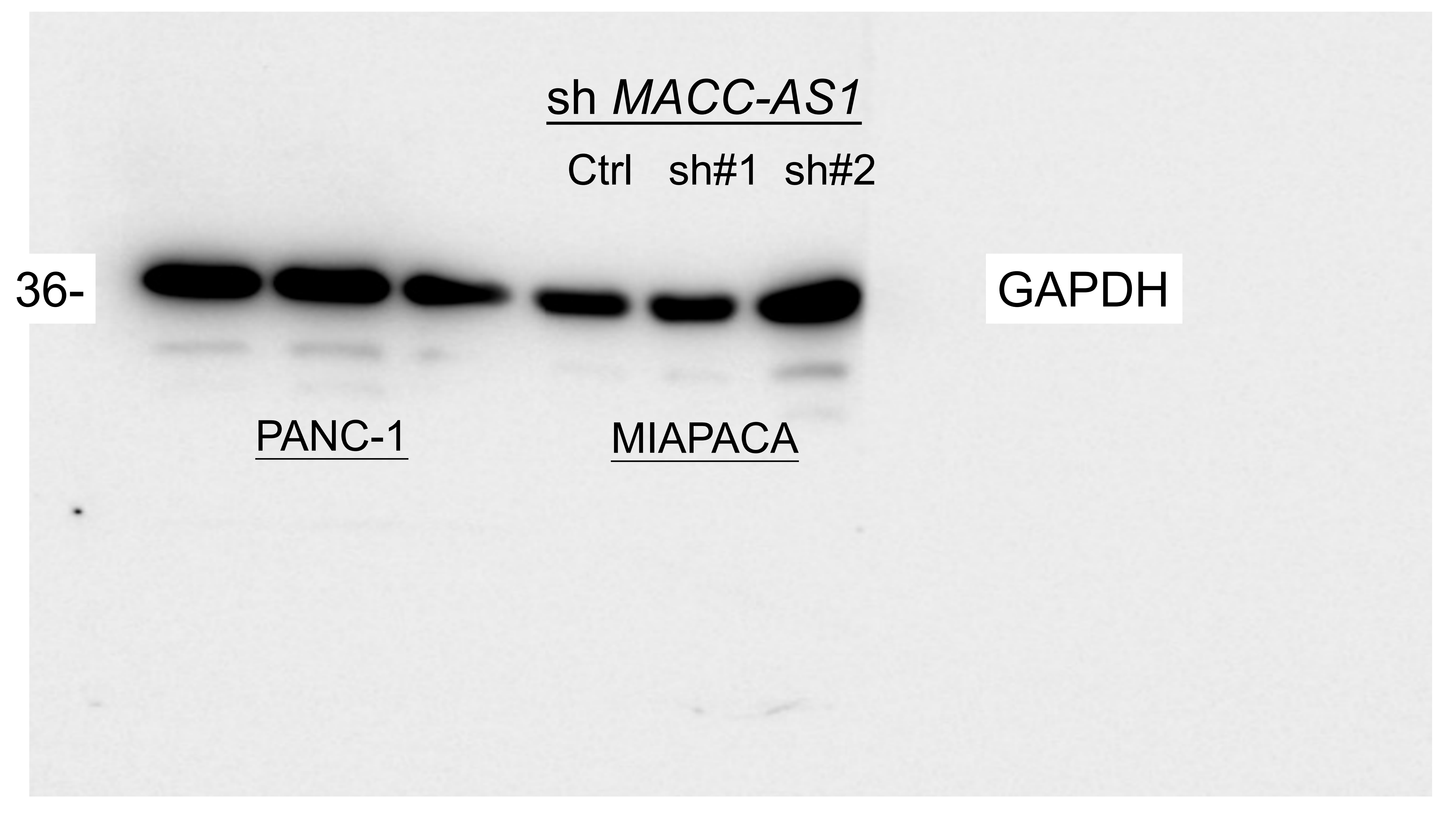
sh MACC-AS1
Ctrl
sh#1
sh#2
36-
GAPDH
PANC-1
MIAPACA

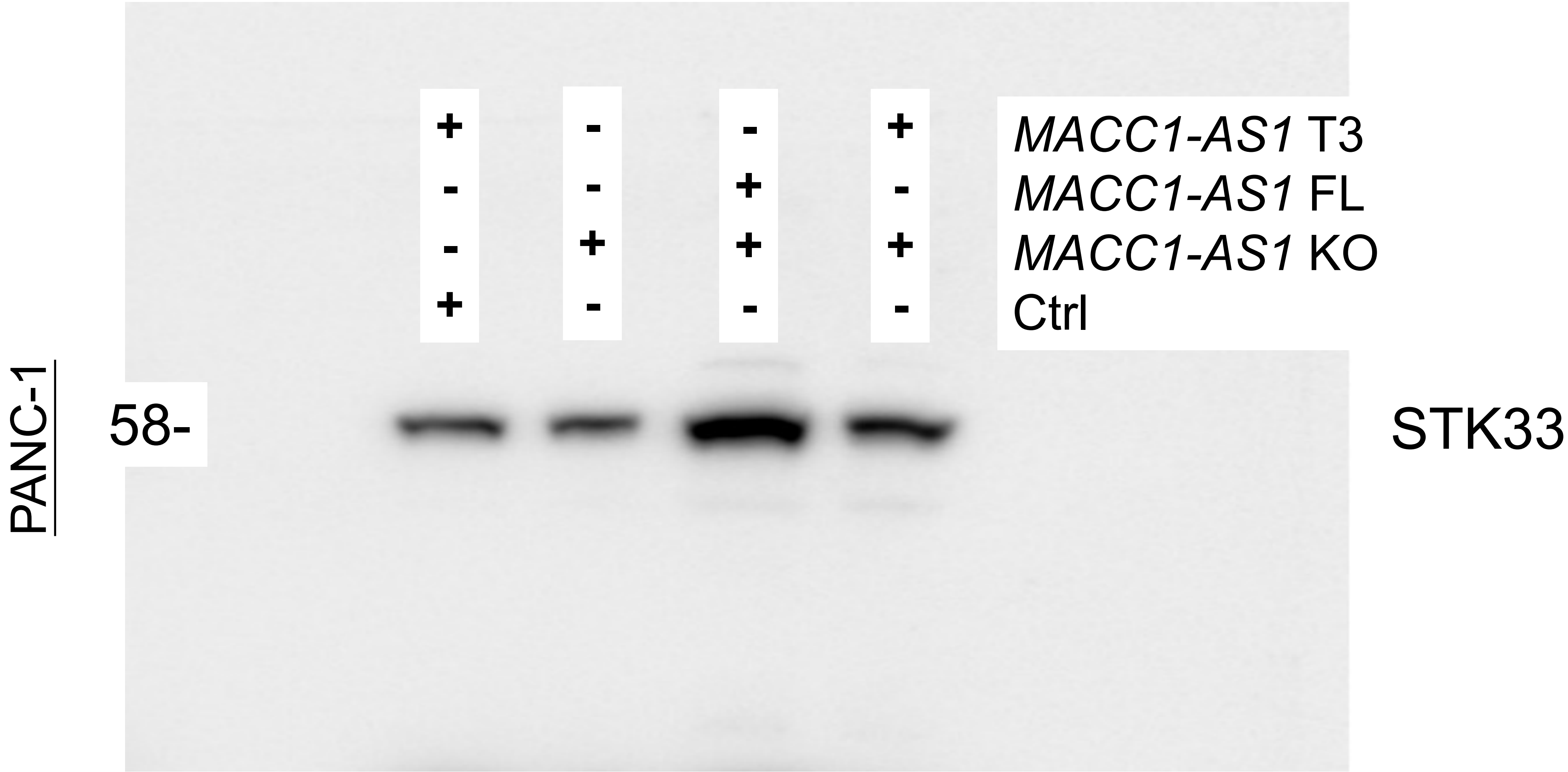

+ - - + *MACC1-AS1* T3
- - + - *MACC1-AS1* FL
- + + + *MACC1-AS1* KO
+ - - - Ctrl
PANC-1
58-
STK33

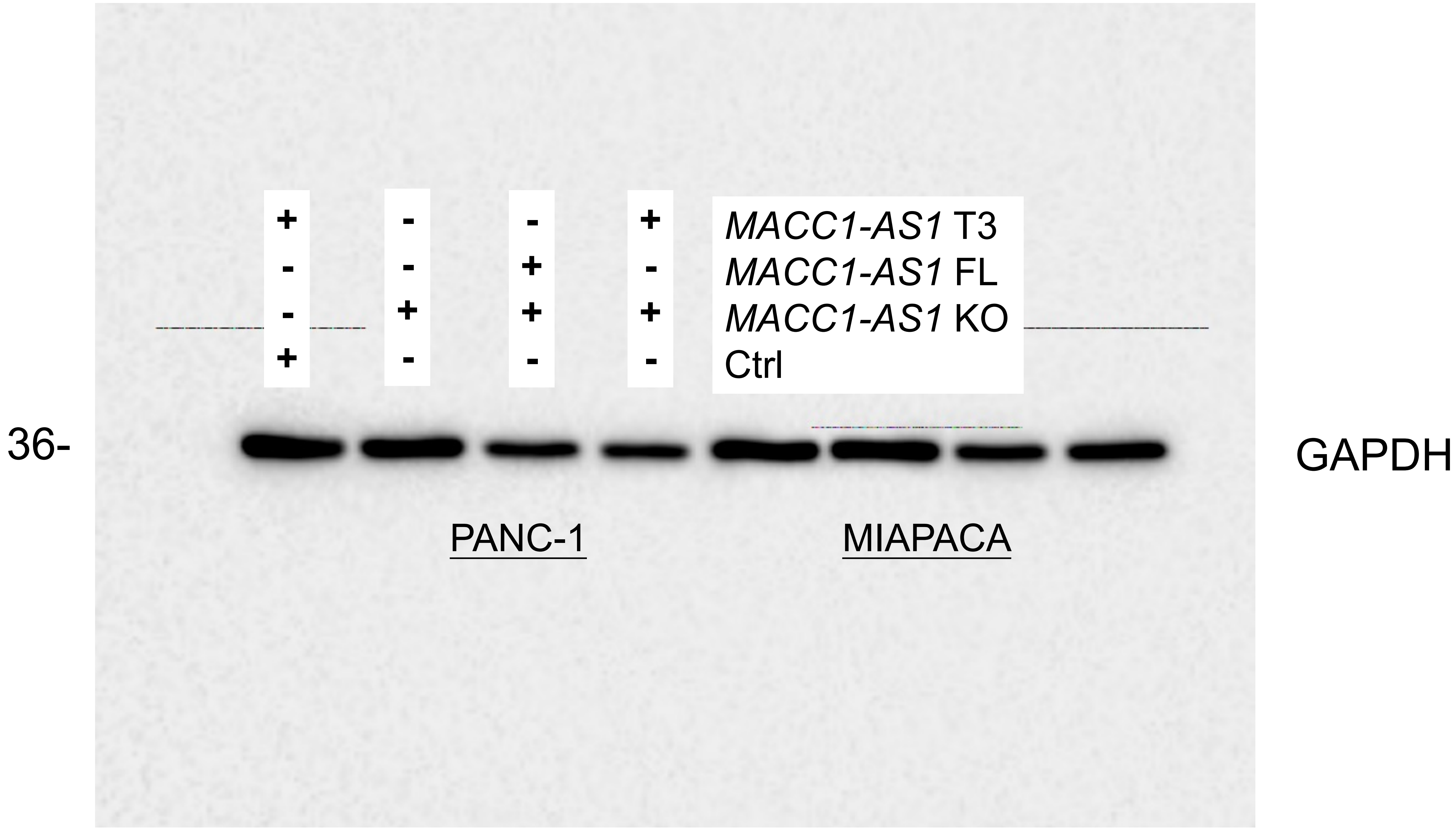

+ - + -
- - + +
- + + -
+ - - -
MACC1-AS1 T3
MACC1-AS1 FL
MACC1-AS1 KO
Ctrl
36-
GAPDH
PANC-1
MIAPACA

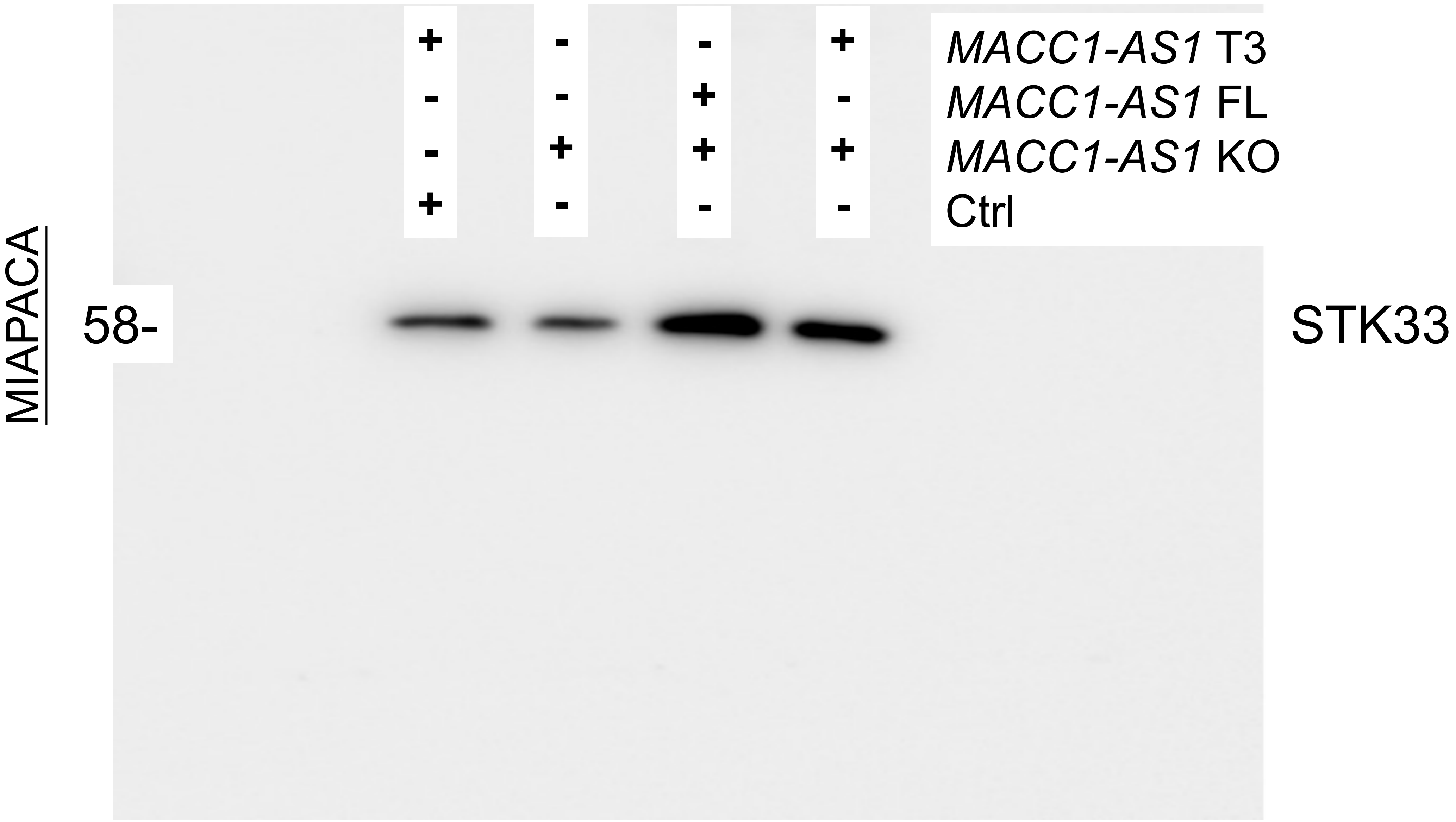
MIAPACA
58-
+ - - +
- - + -
- + + +
+ - - -
*MACC1-AS1* T3
*MACC1-AS1* FL
*MACC1-AS1* KO
Ctrl
STK33

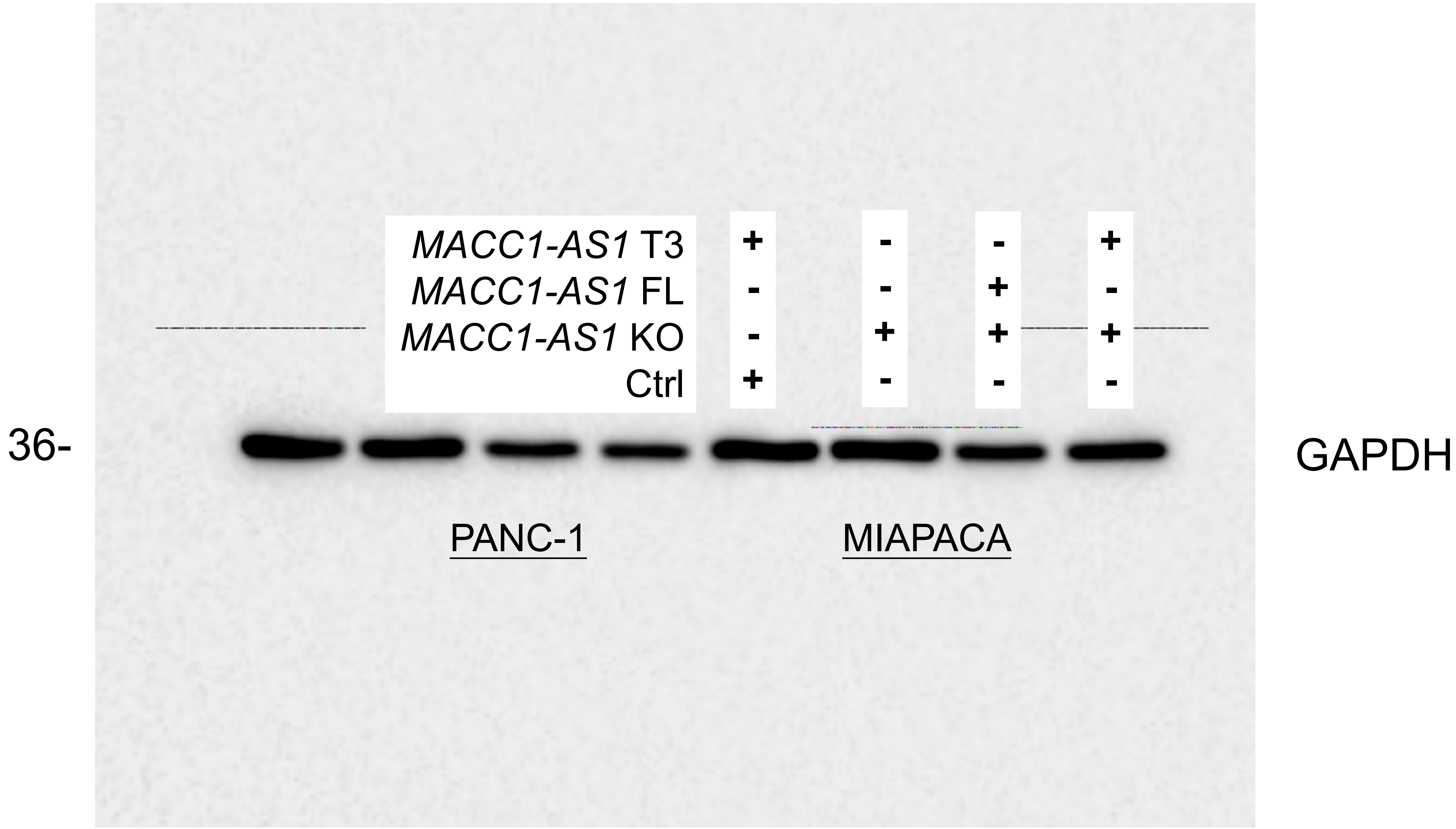
MACC1-AS1 T3 + - - +
MACC1-AS1 FL - - + -
MACC1-AS1 KO - + + +
Ctrl + - - -
36-
GAPDH
PANC-1
MIAPACA

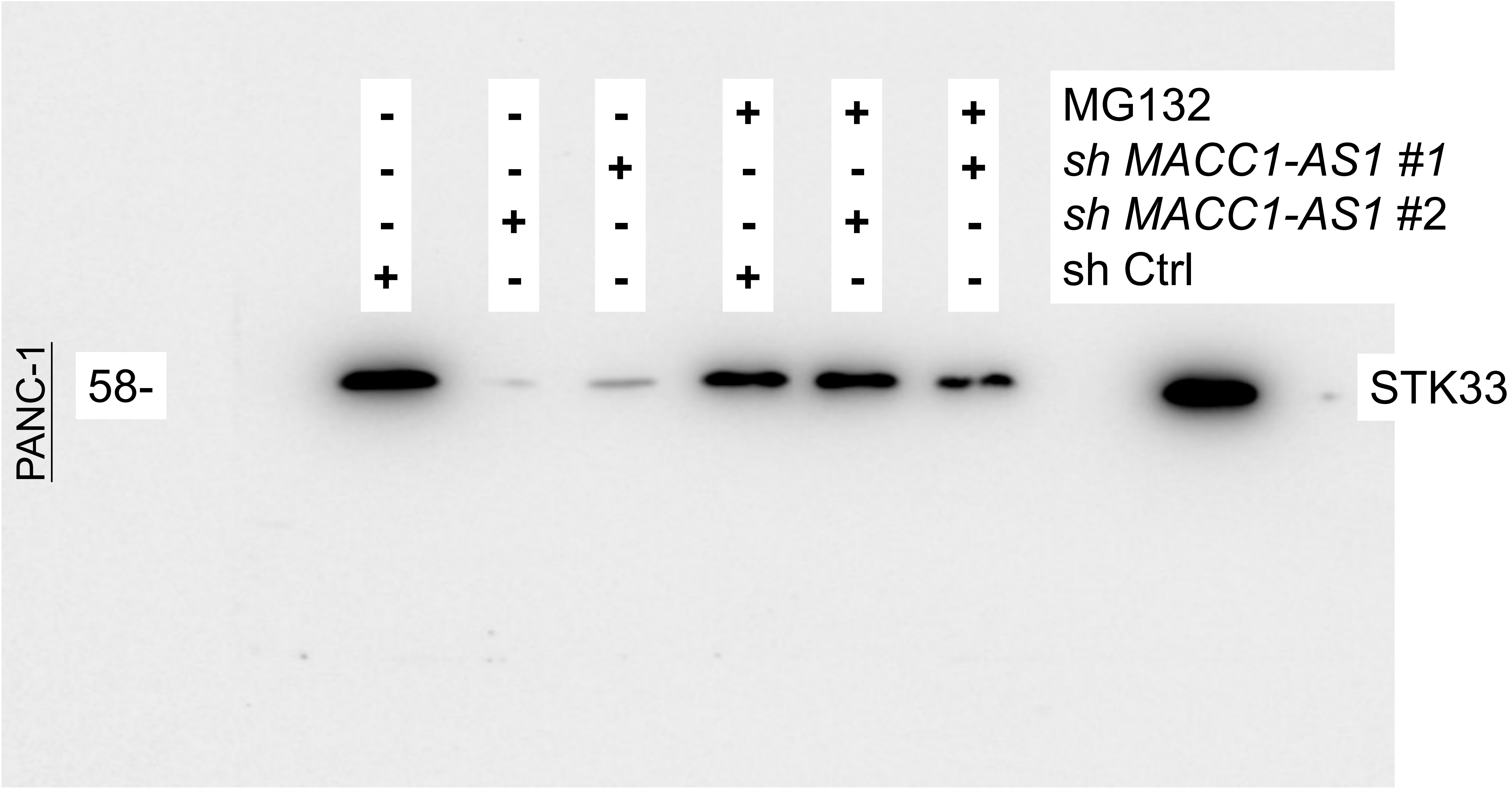
MG132
sh MACC1-AS1 #1
sh MACC1-AS1 #2
sh Ctrl
PANC-1
58-
STK33

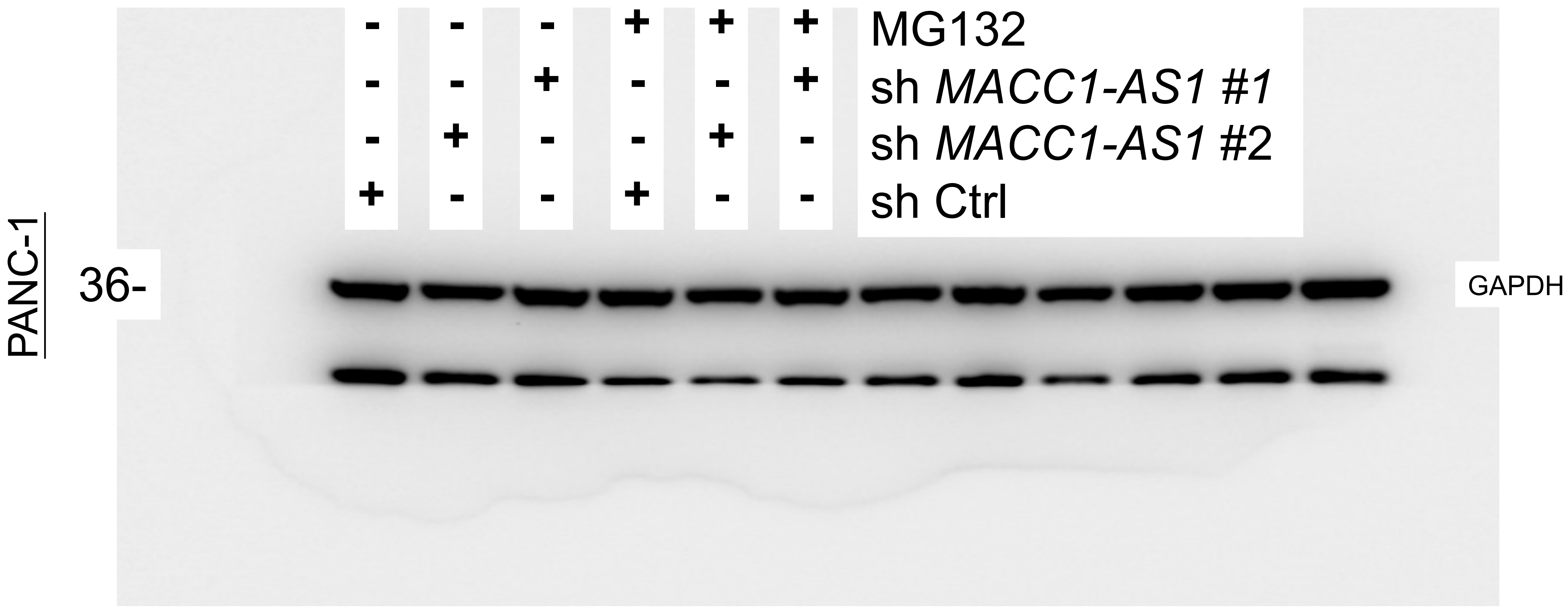

MG132 - - - + + +
sh *MACC1-AS1 #1* - - + - - +
sh *MACC1-AS1 #2* - + - - + -
sh Ctrl + - - + - -
PANC-1
36-
GAPDH

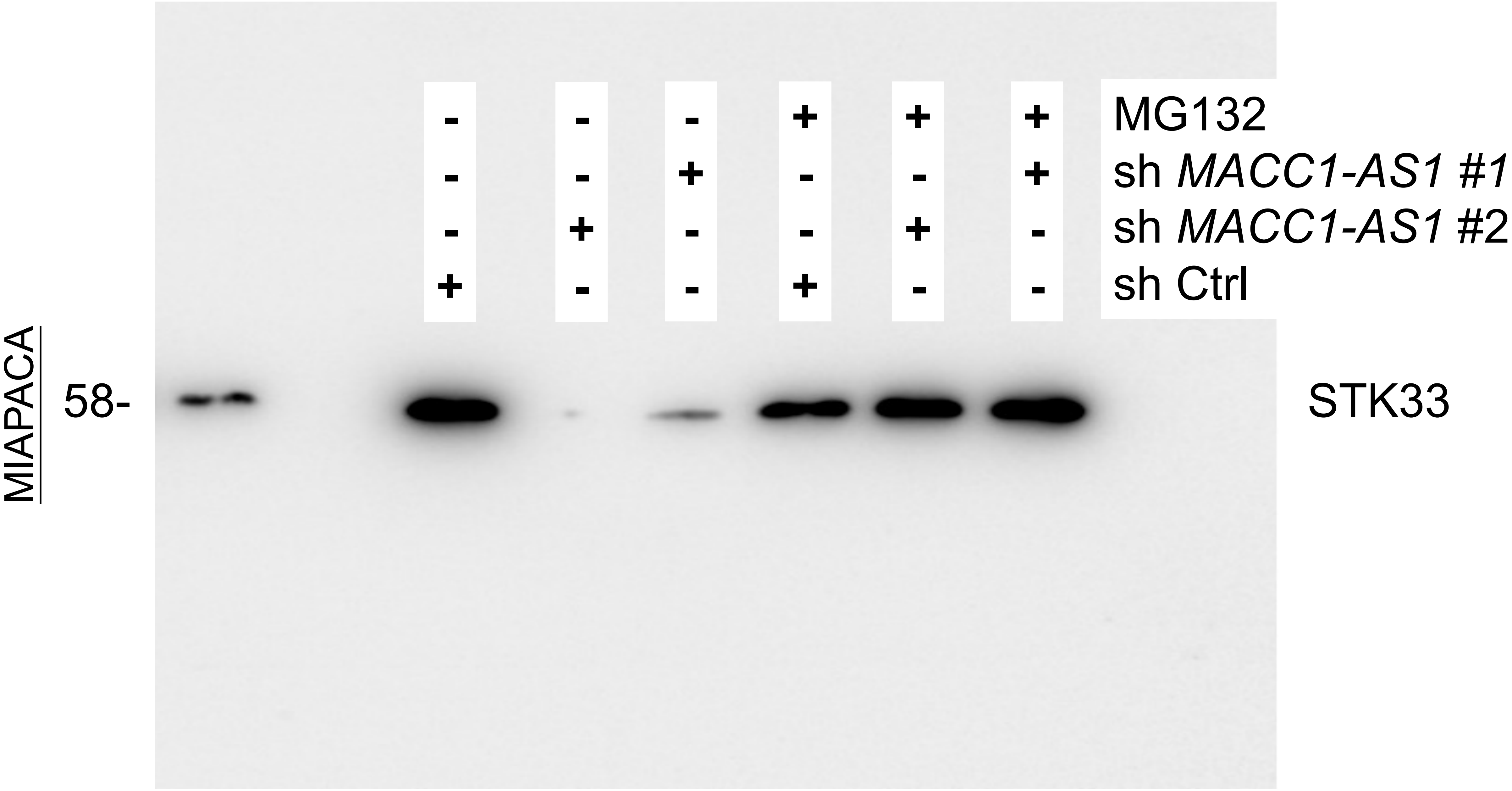
MG132 - - - + + +
sh *MACC1-AS1 #1* - - + - - +
sh *MACC1-AS1 #2* - + - - + -
sh Ctrl + - - + - -
MIAPACA
58-
STK33

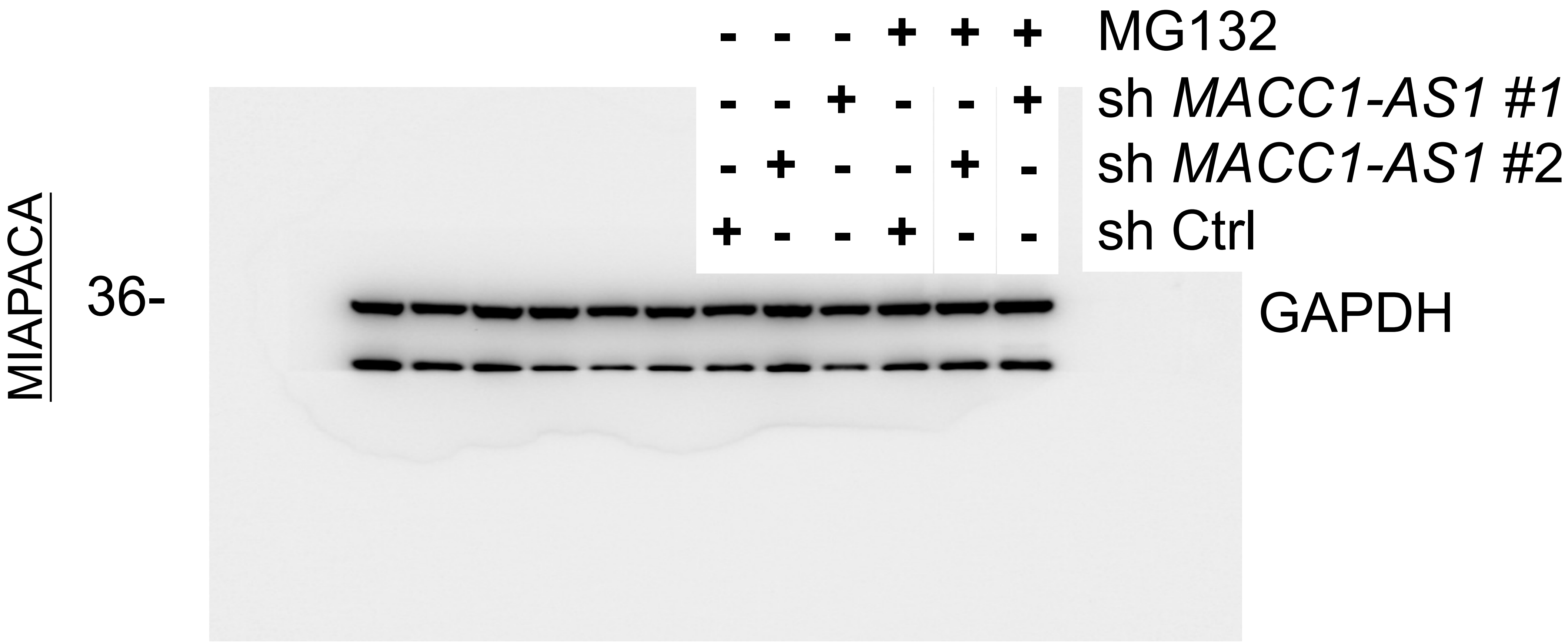

MG132
- - - + + +
sh *MACC1-AS1 #1*
- - + - - +
sh *MACC1-AS1* #2
- + - - + -
sh Ctrl
+ - - + - -
MIAPACA
36-
GAPDH

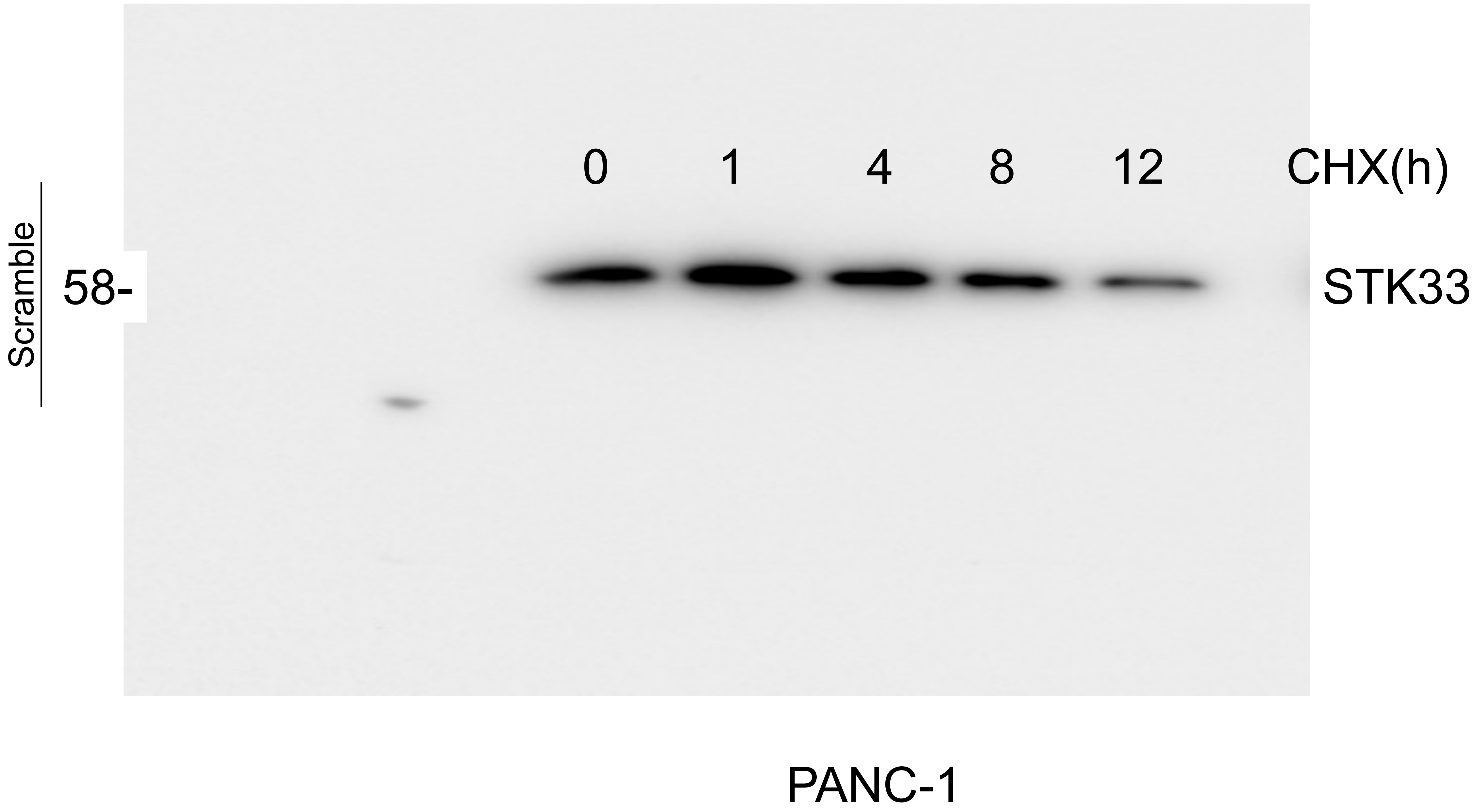
Scramble
58-
0
1
4
8
12
CHX(h)
STK33
PANC-1

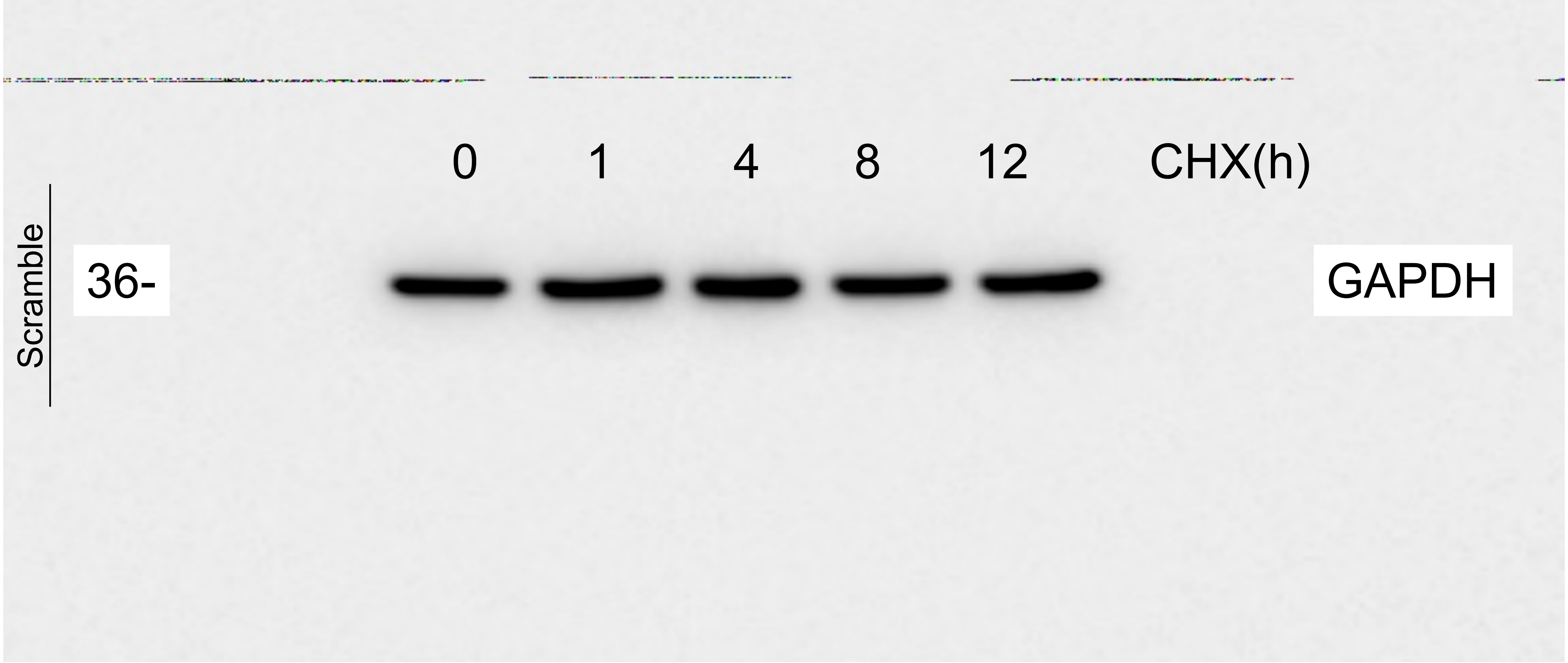


PANC-1

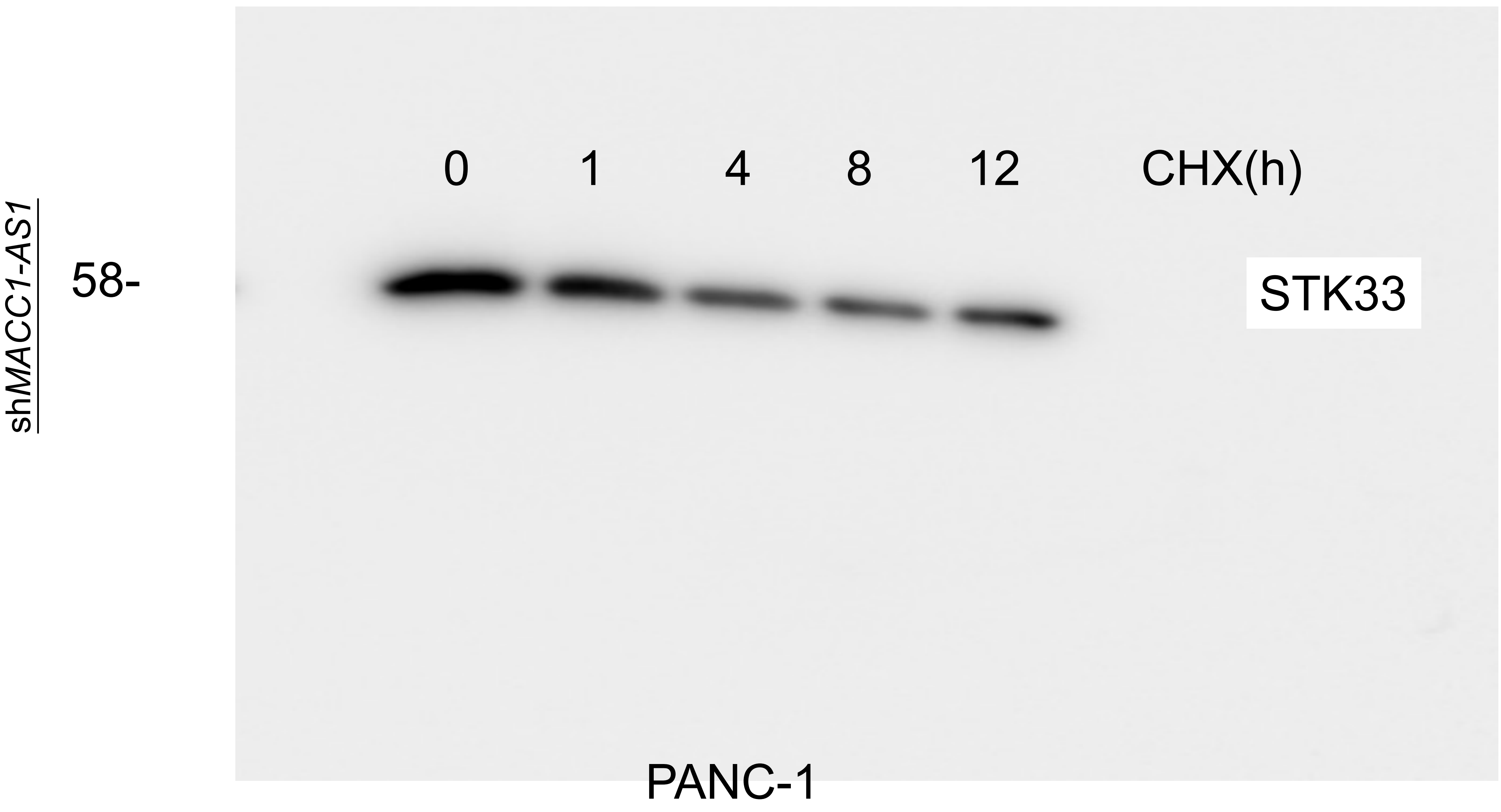
shMACC1-AS1
58-
0 1 4 8 12 CHX(h)
STK33
PANC-1

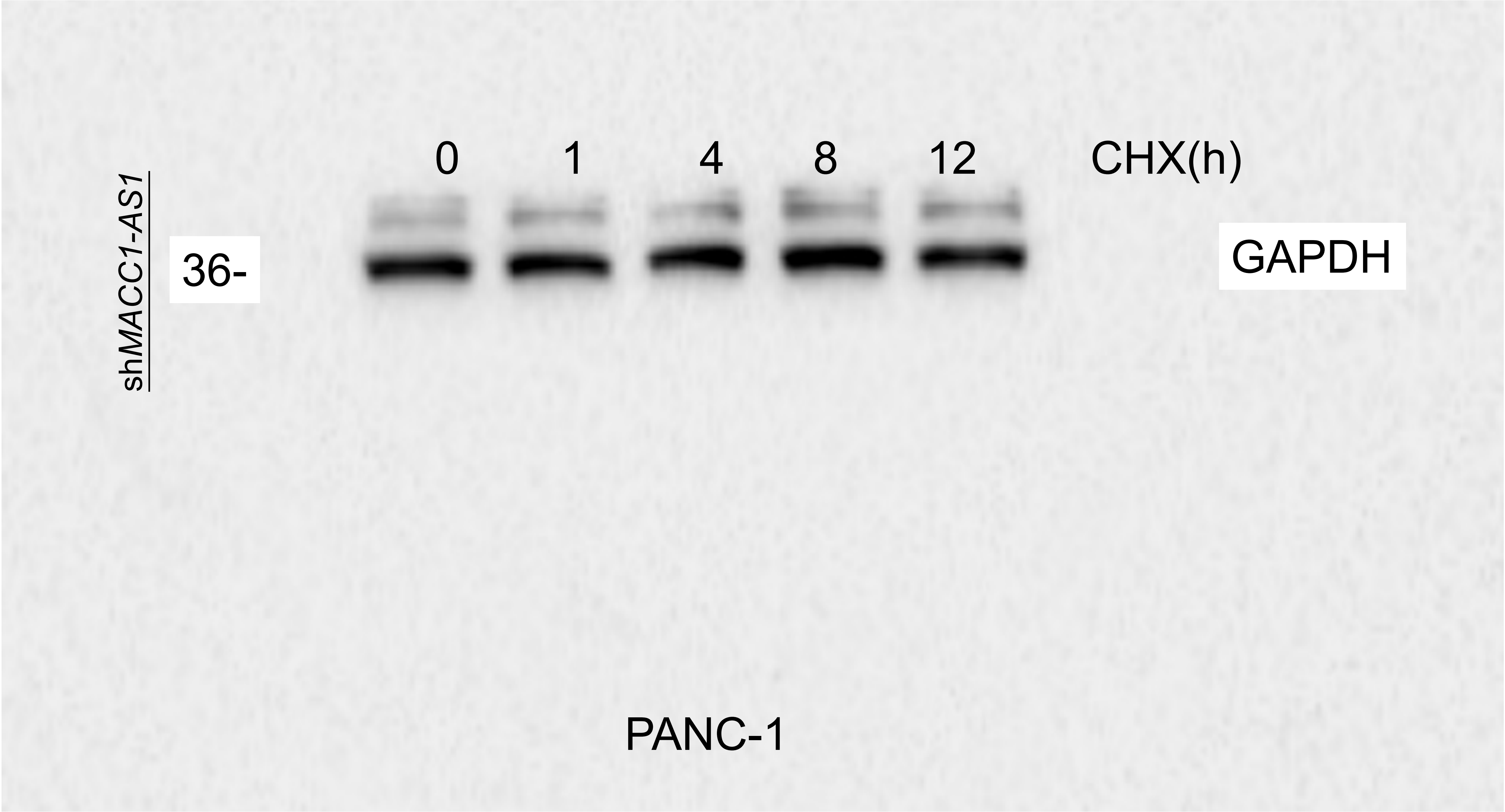
0
1
4
8
12
CHX(h)
shMACC1-AS1
36-
GAPDH
PANC-1

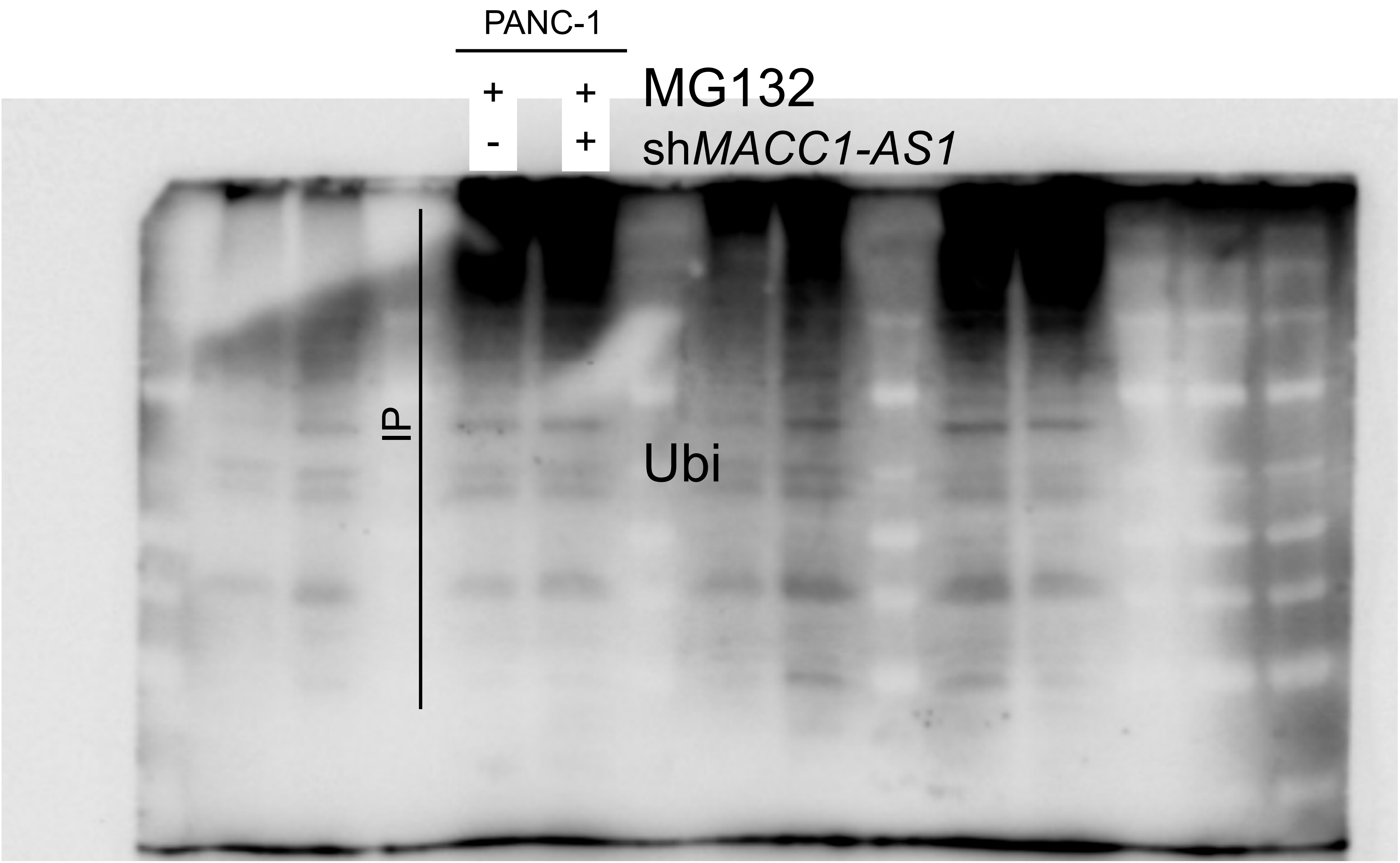
PANC-1
MG132
+ +
- +
shMACC1-AS1
IP
Ubi

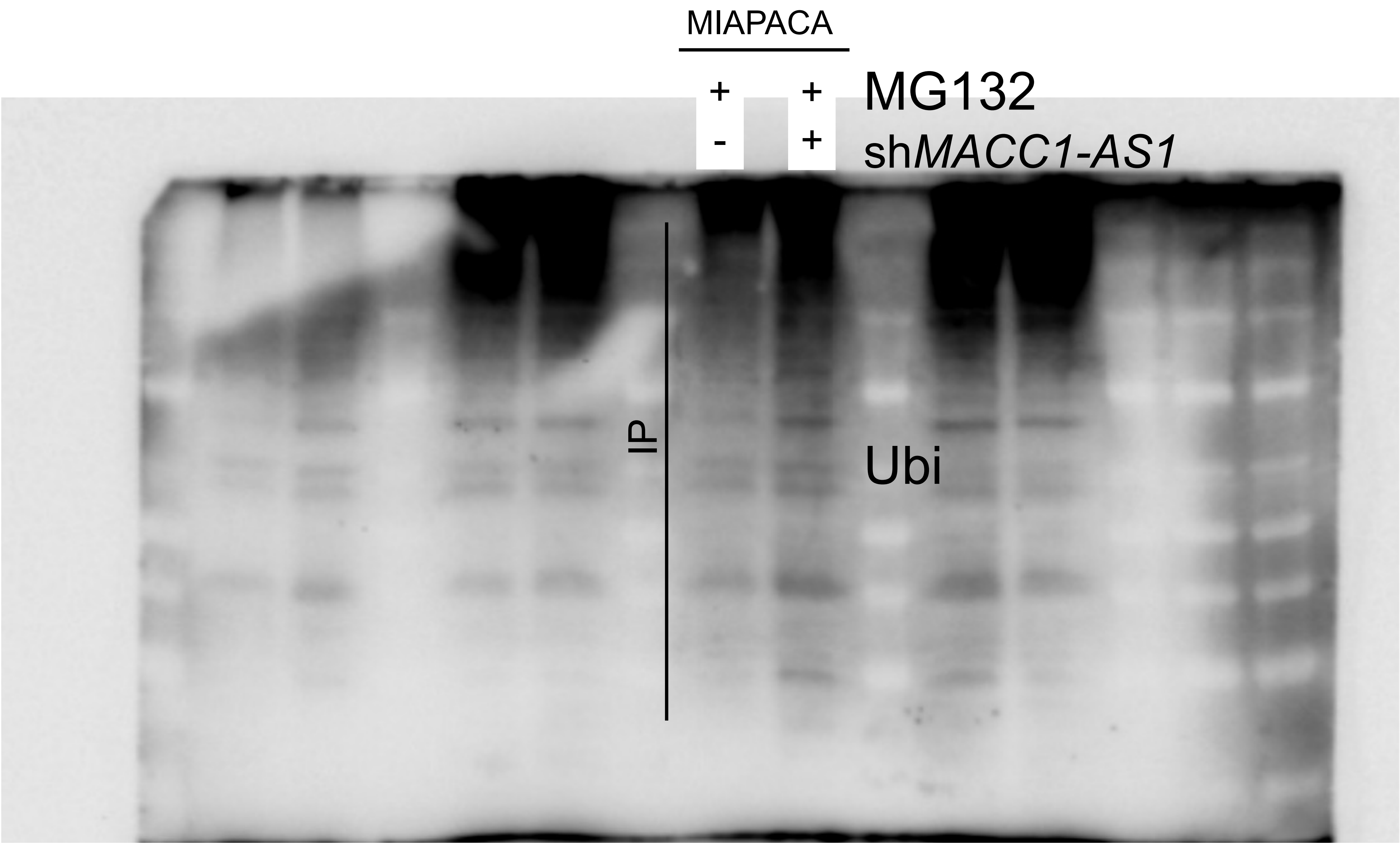
MIAPACA
+ +
MG132
- +
shMACC1-AS1
IP
Ubi

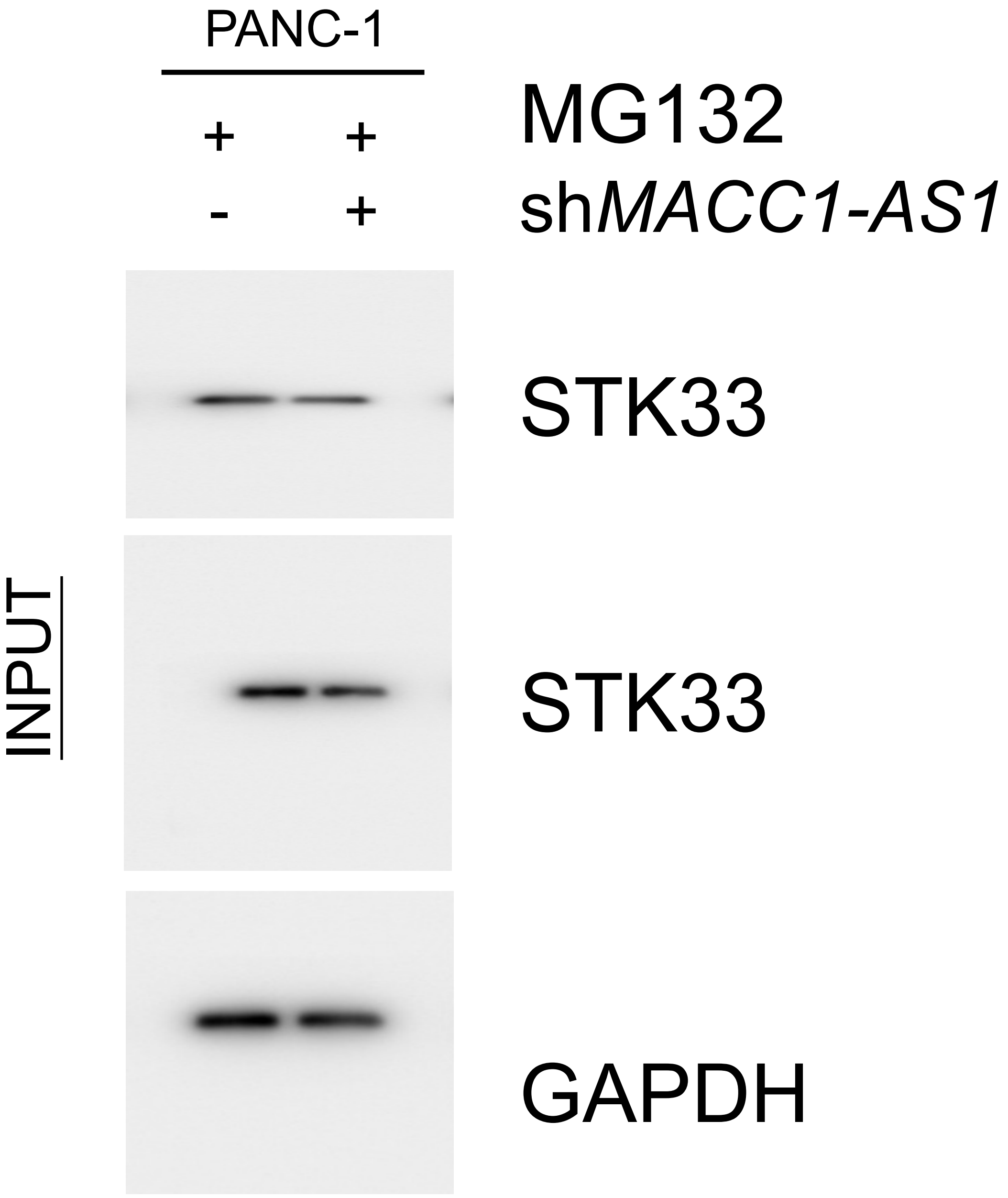
PANC-1
MG132
+ +
sh*MACC1-AS1*
- +
STK33
STK33
INPUT
GAPDH

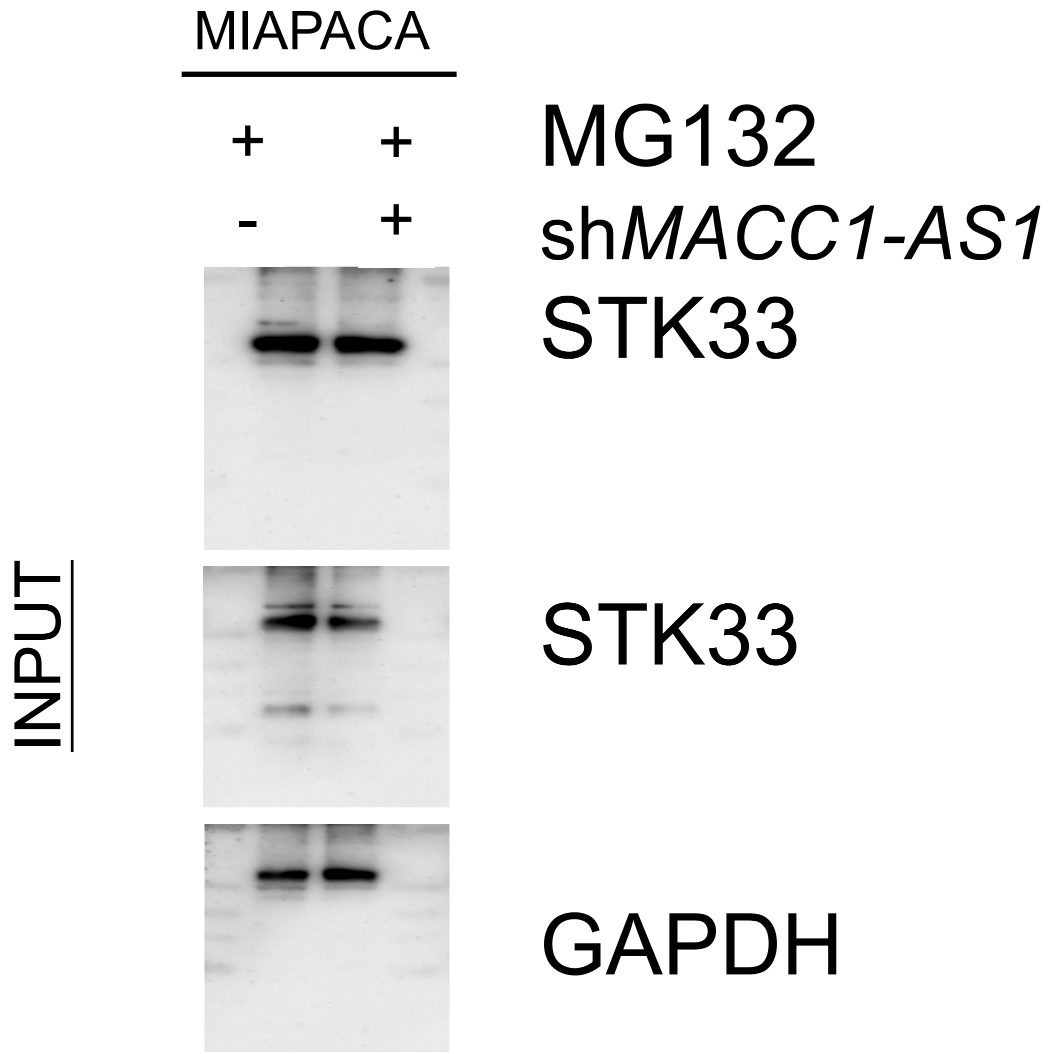
MIAPACA
+ +
MG132
- +
sh*MACC1-AS1*
STK33
INPUT
STK33
GAPDH

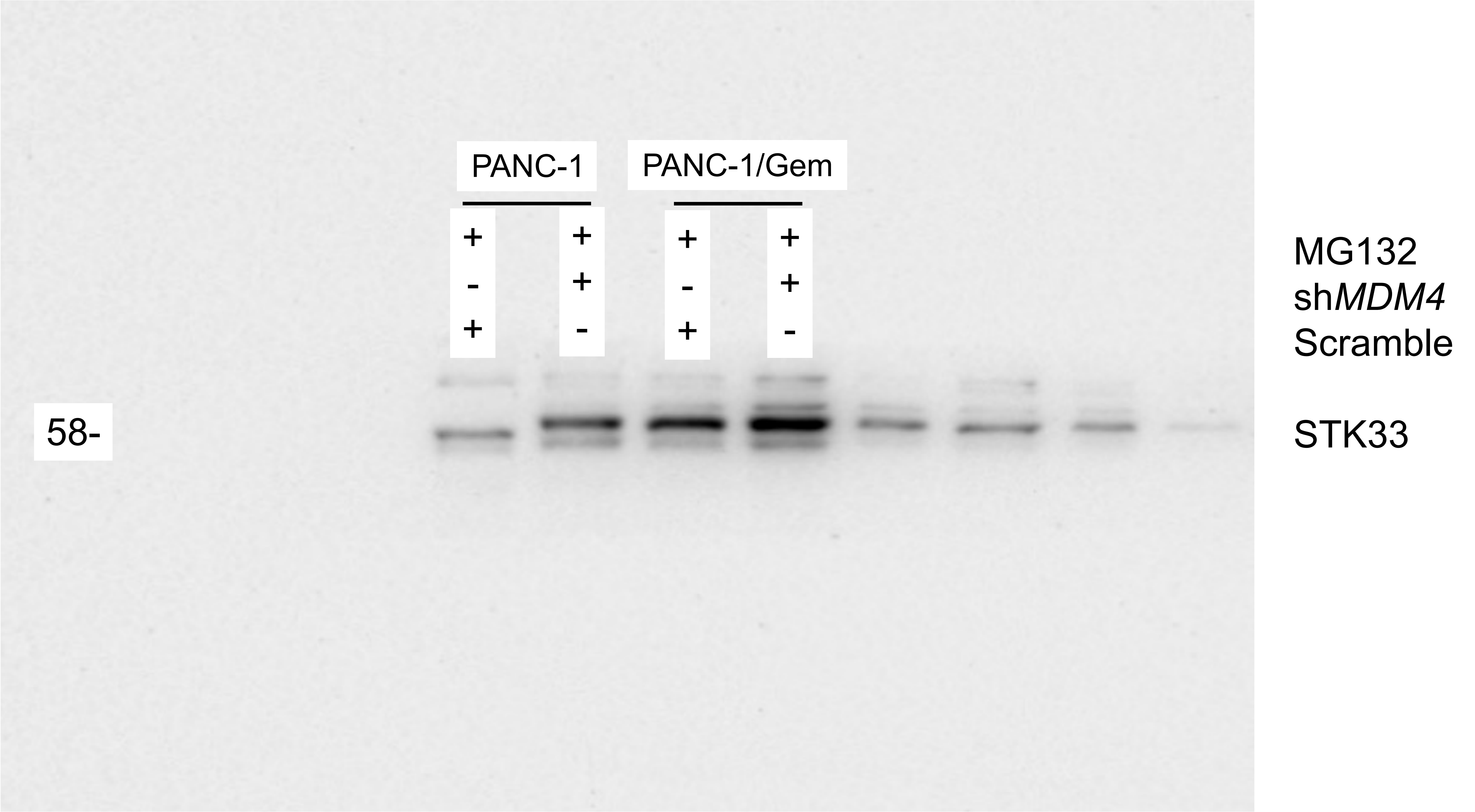
PANC-1
PANC-1/Gem
+ + + +
- + - +
+ - + -
MG132
shMDM4
Scramble
58-
STK33

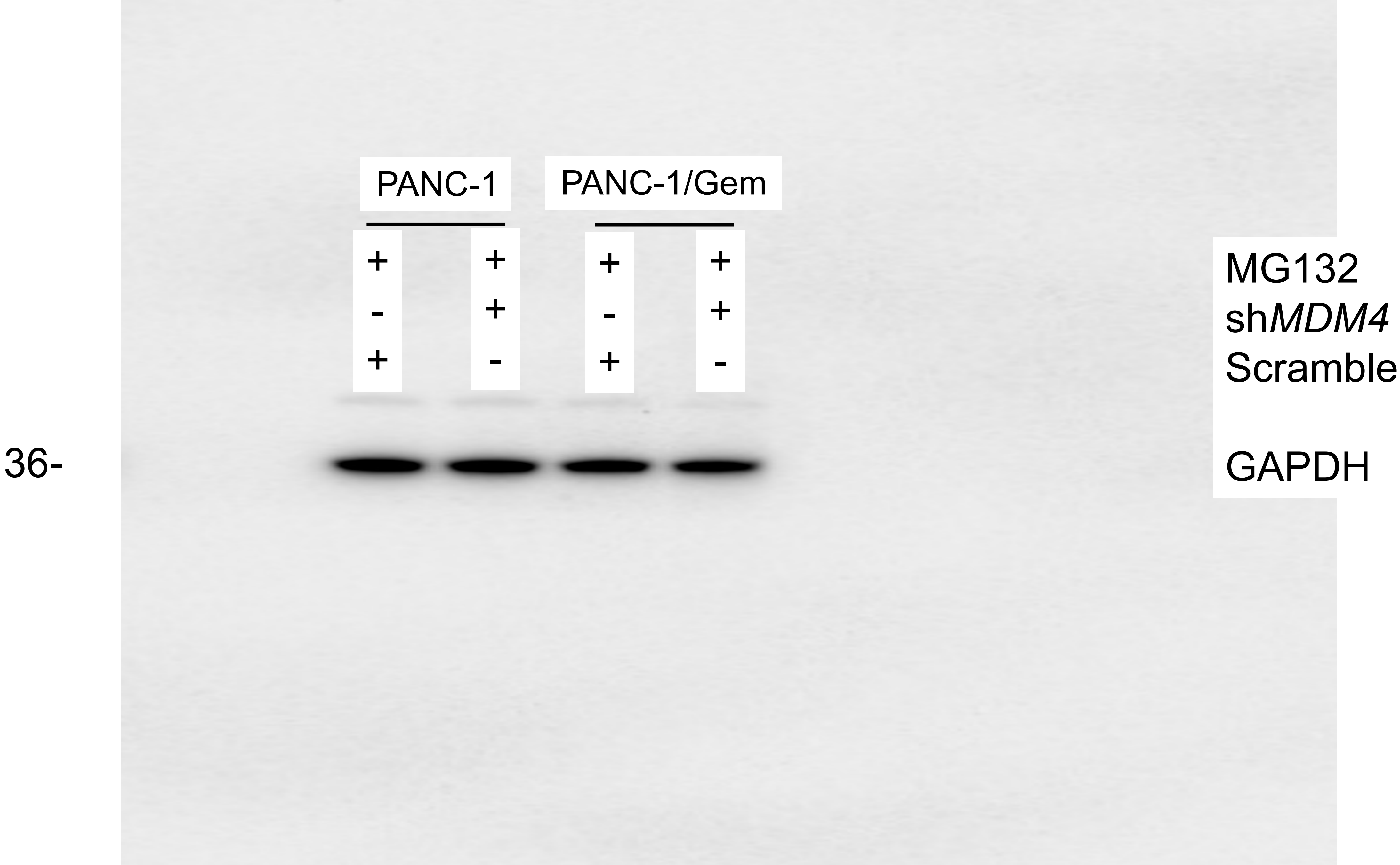

PANC-1
PANC-1/Gem
+ + + +
- + - +
+ - + -
MG132
shMDM4
Scramble
36-
GAPDH

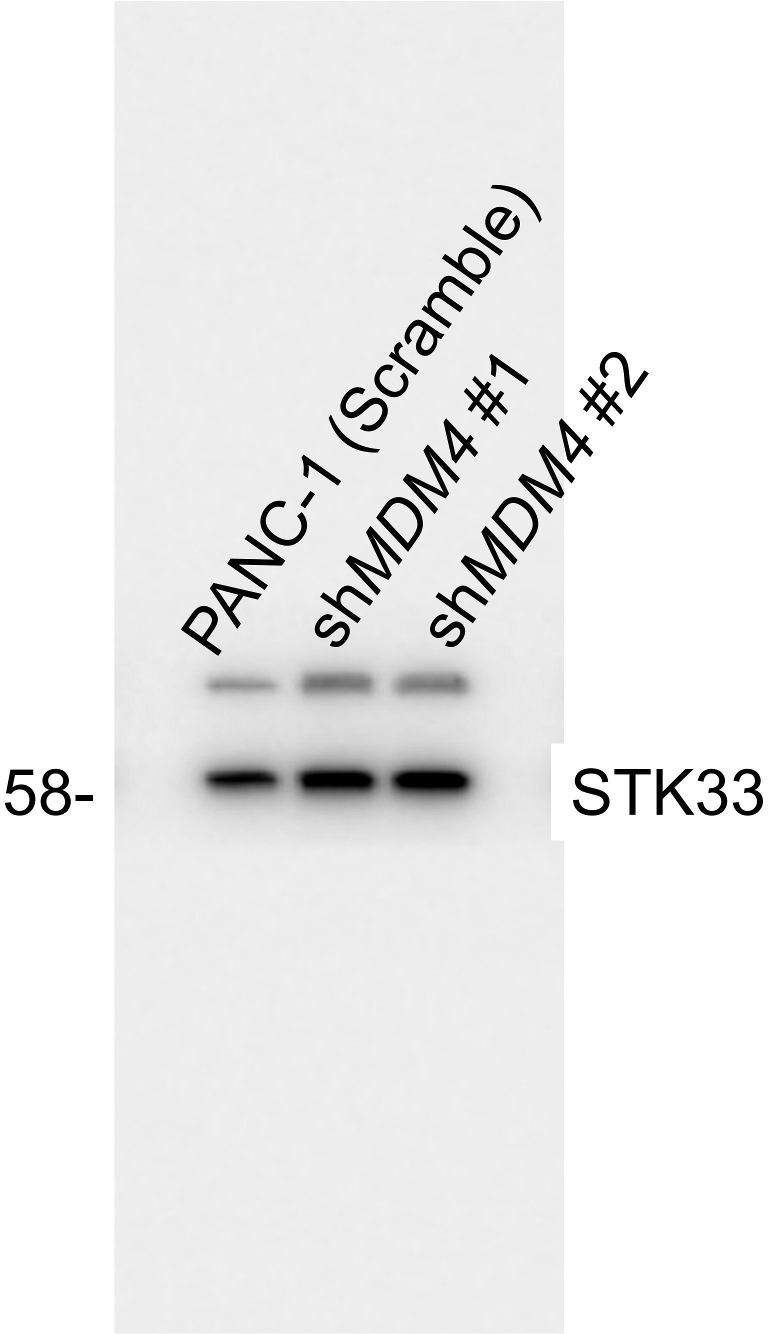
PANC-1 (Scramble)
shMDM4 #1
shMDM4 #2
58-
STK33

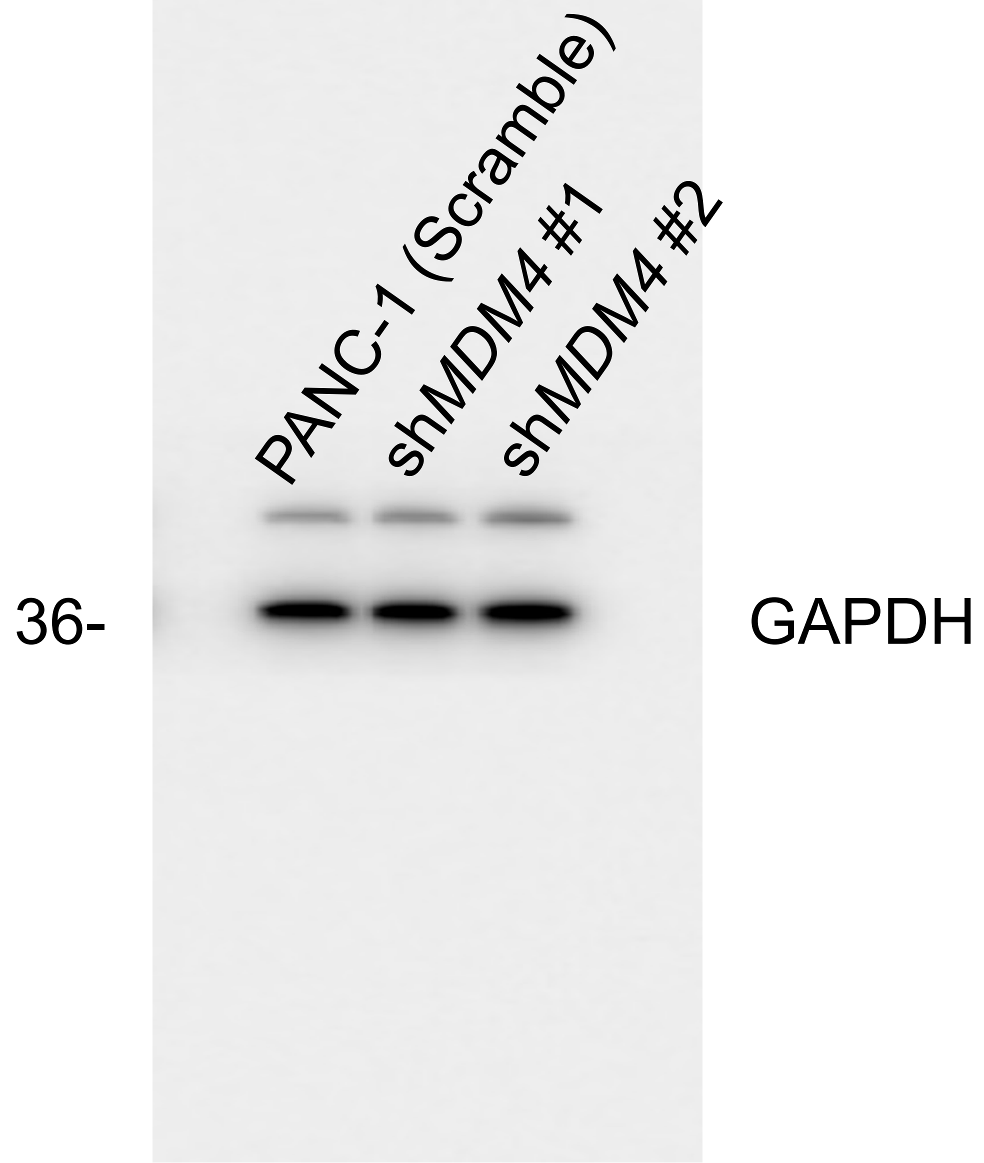
PANC-1 (Scramble)
shMDM4 #1
shMDM4 #2
36-
GAPDH

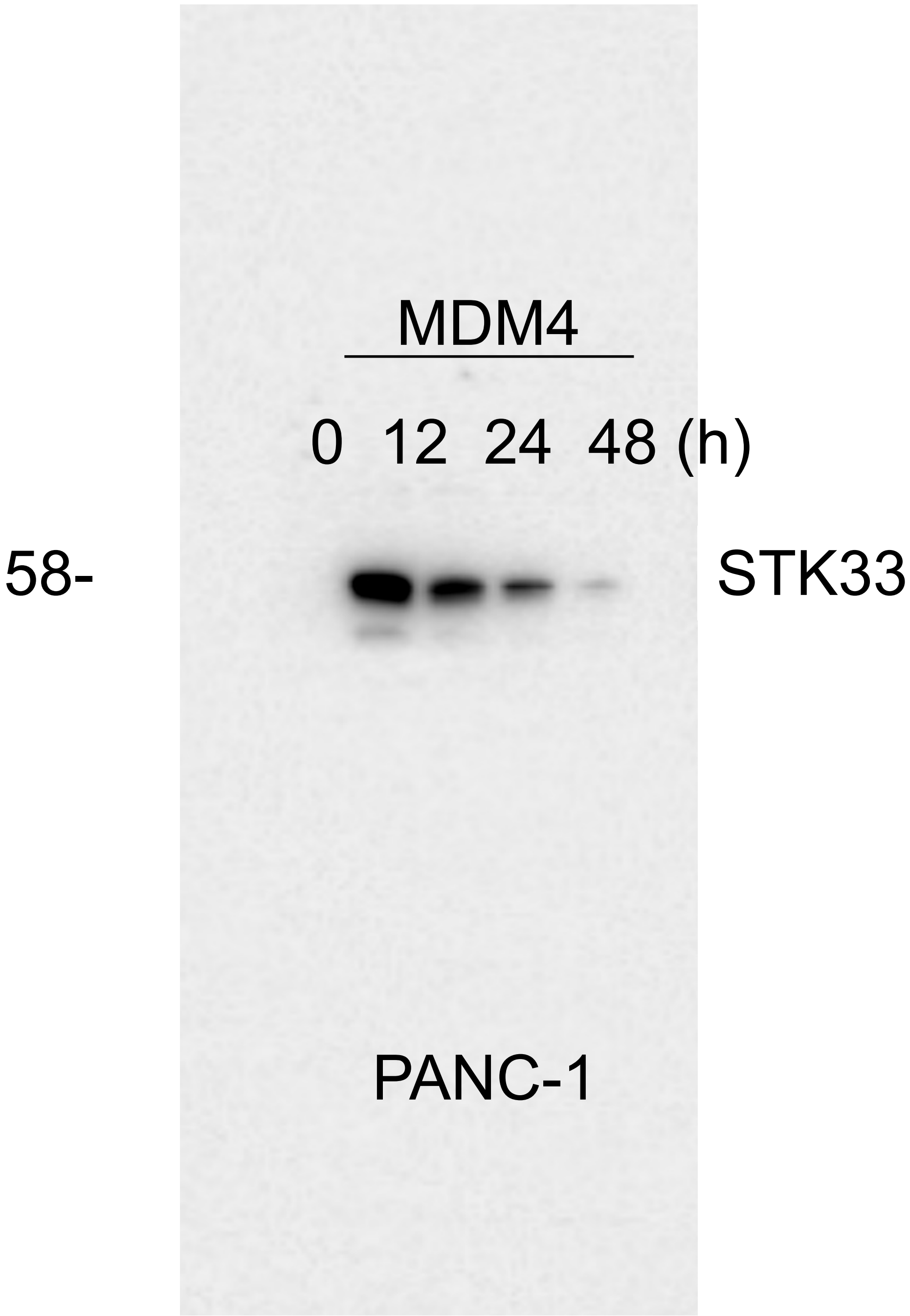
MDM4
0 12 24 48 (h)
58-
STK33
PANC-1

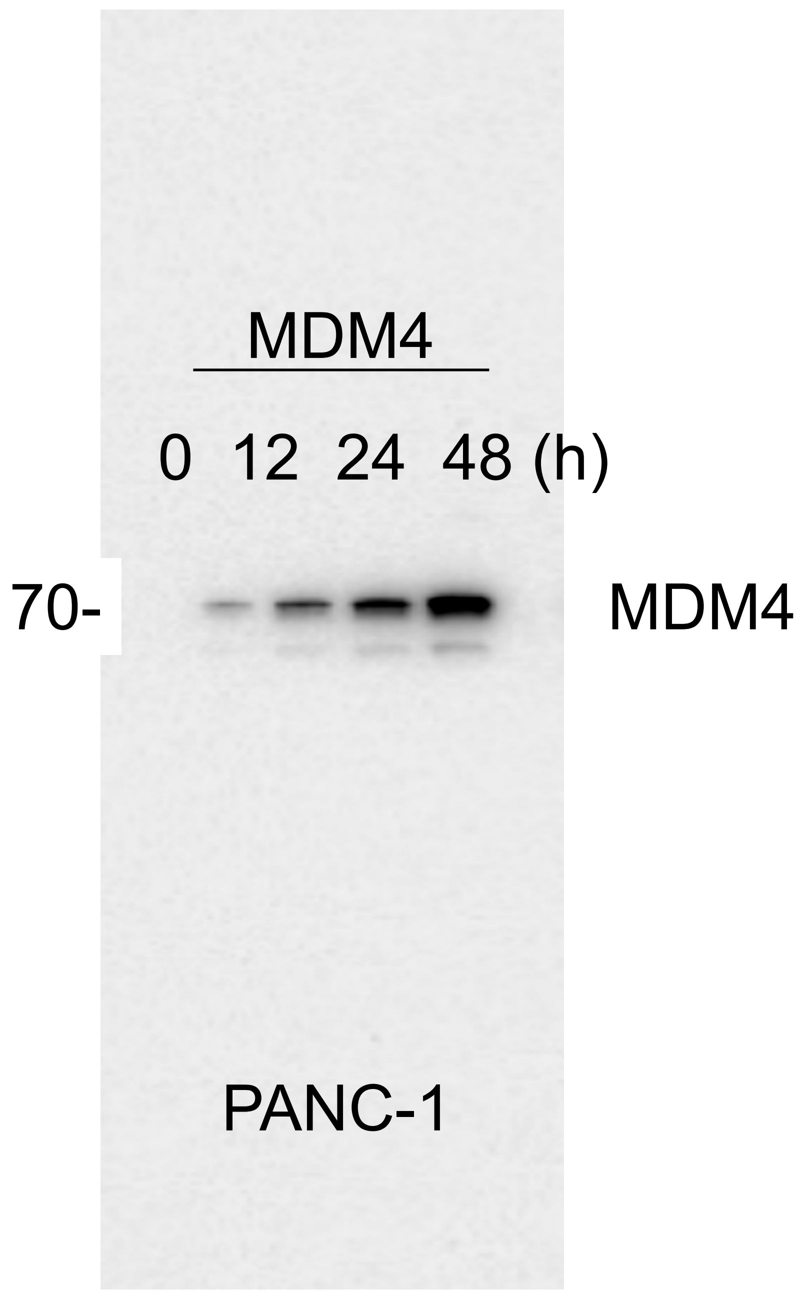
MDM4
0 12 24 48 (h)
70-
MDM4
PANC-1

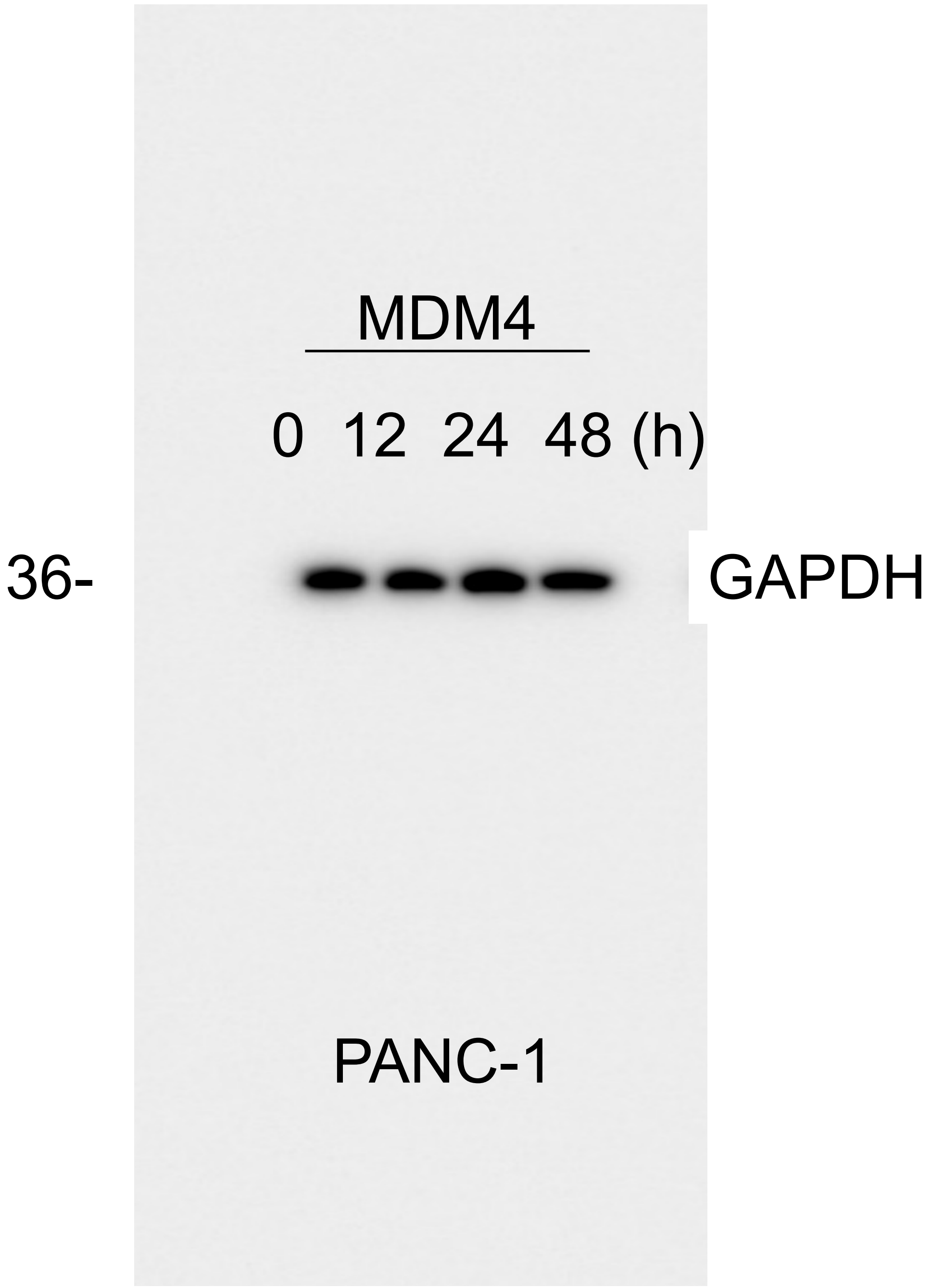

MDM4
0 12 24 48 (h)
36-
GAPDH
PANC-1

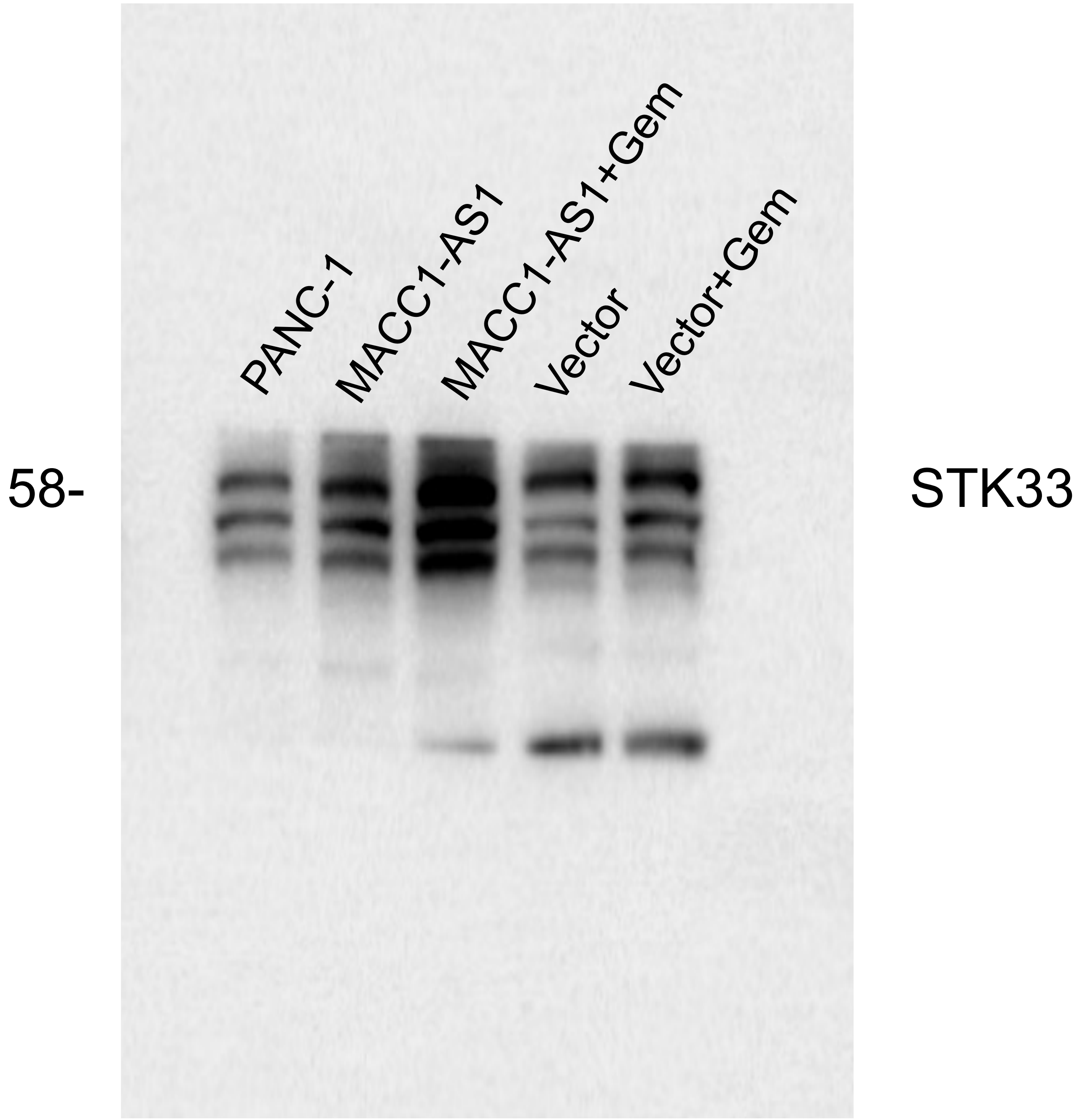
PANC-1
MACC1-AS1
MACC1-AS1+Gem
Vector
Vector+Gem
58-
STK33

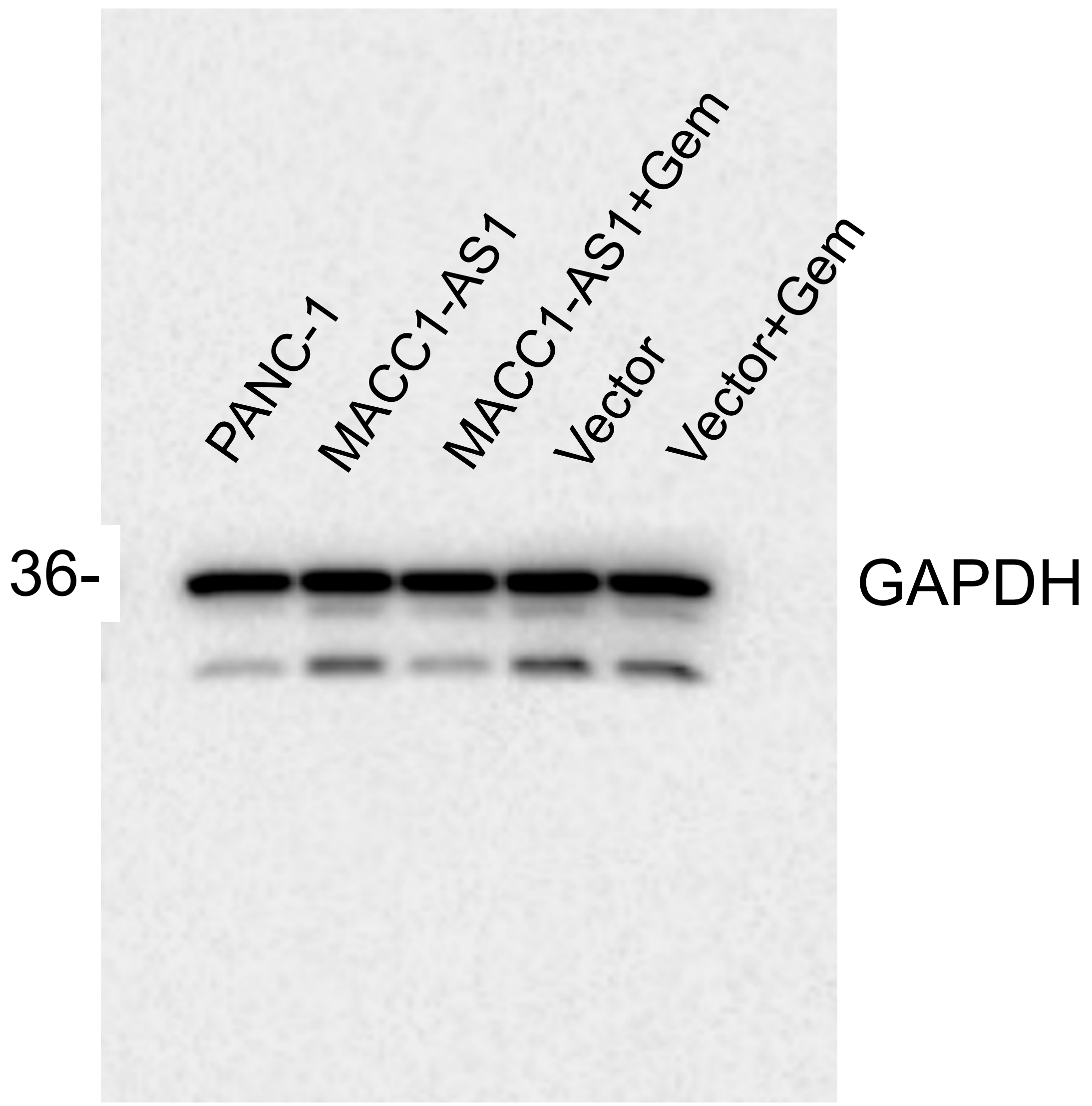
PANC-1
MACC1-AS1
MACC1-AS1+Gem
Vector
Vector+Gem
36-
GAPDH

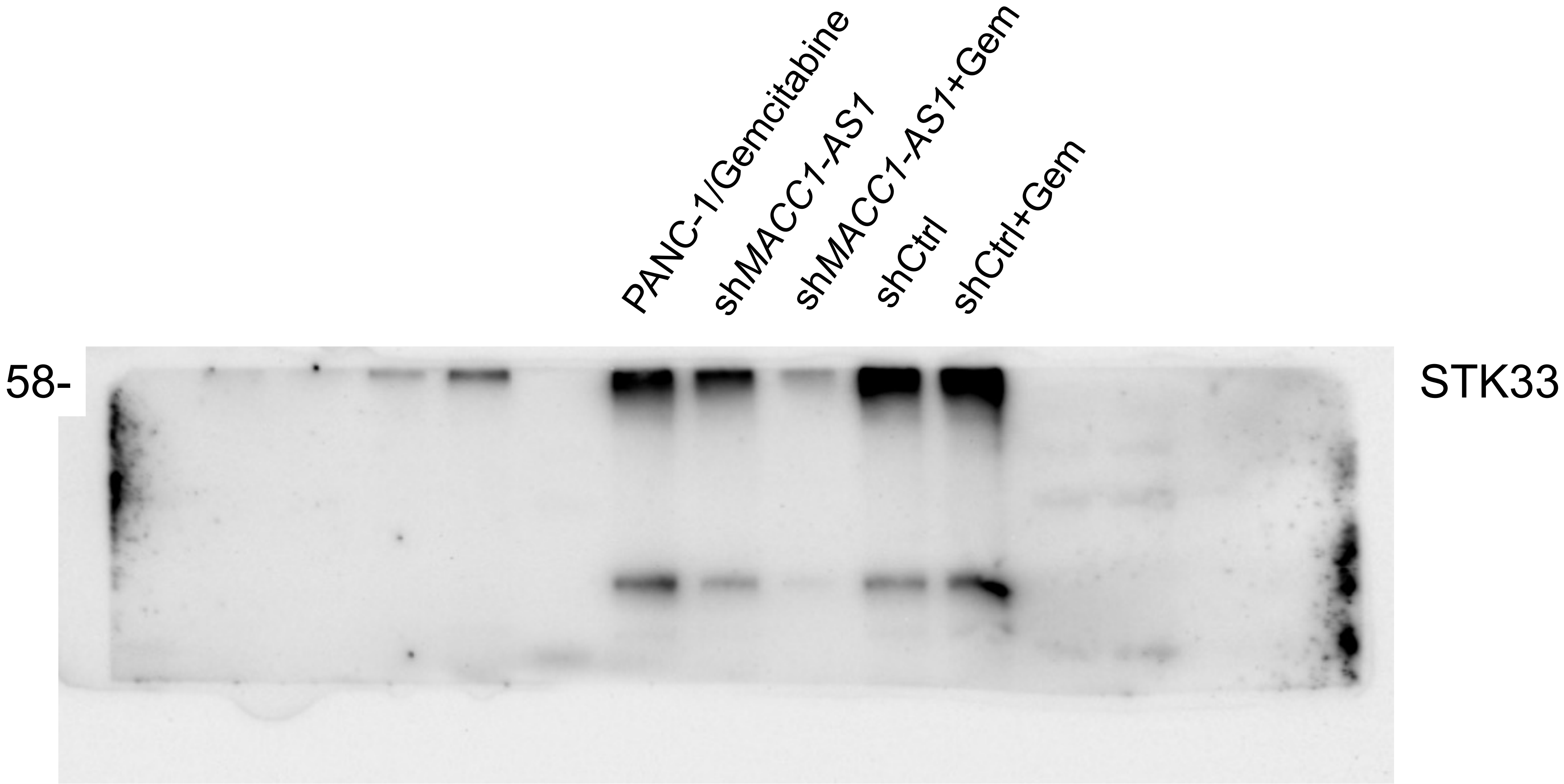
PANC-1/Gemcitabine
shMACC1-AS1
shMACC1-AS1+Gem
shCtrl
shCtrl+Gem
58-
STK33

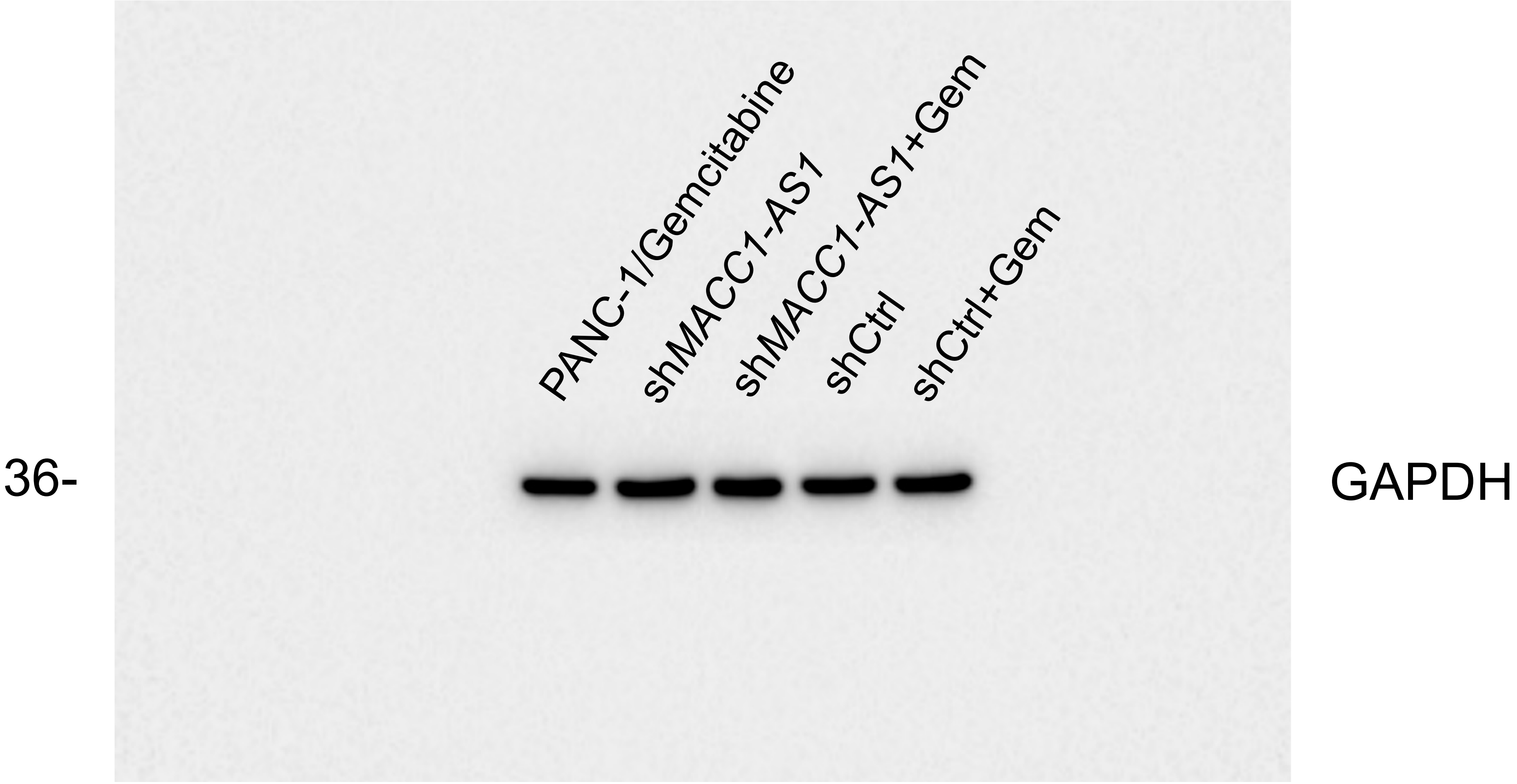
PANC-1/Gemcitabine
shMACC1-AS1
shMACC1-AS1+Gem
shCtrl
shCtrl+Gem
36-
GAPDH

Figure 4

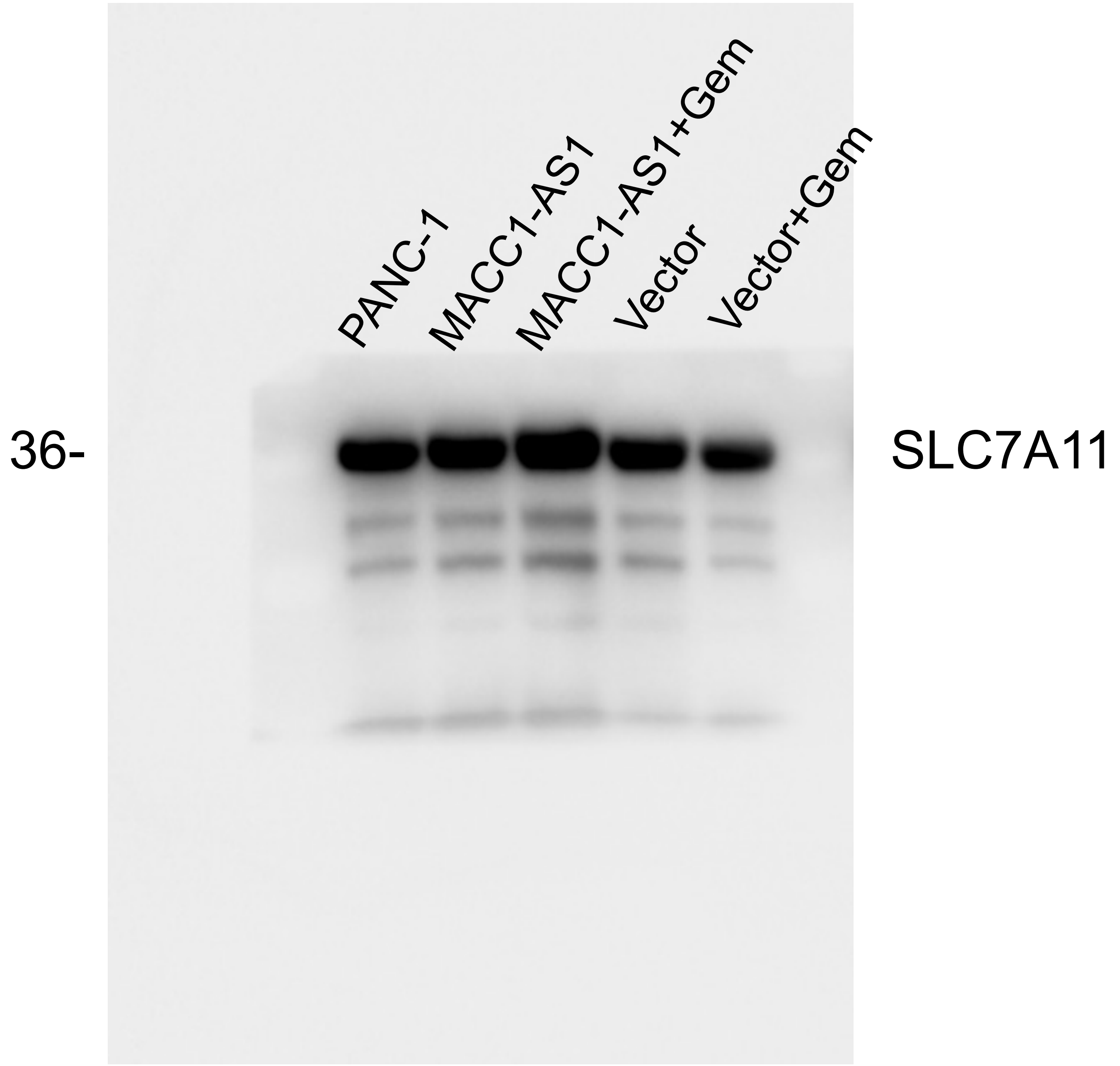

PANC-1
MACC1-AS1
MACC1-AS1+Gem
Vector
Vector+Gem
36-
SLC7A11

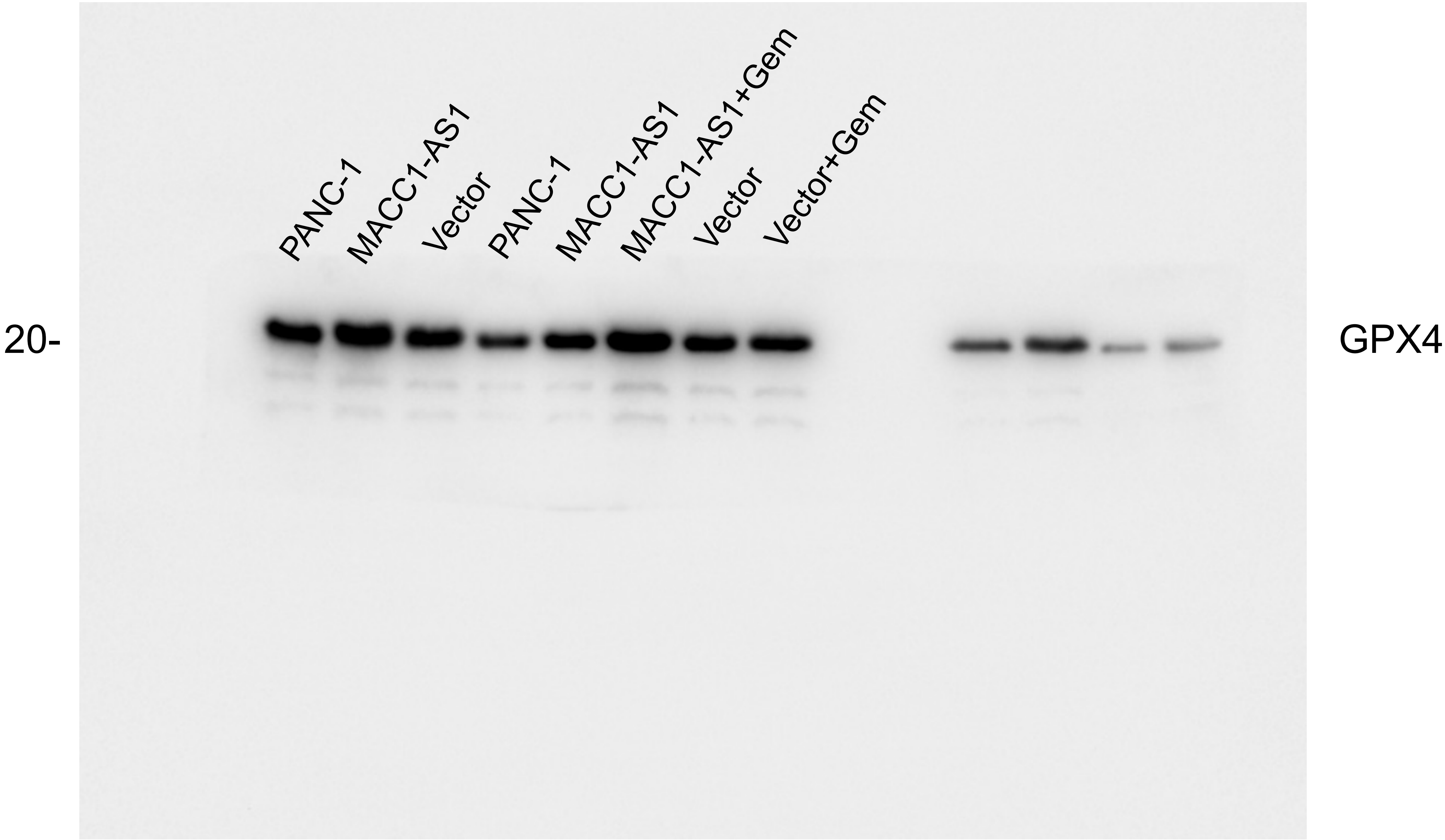

PANC-1
MACC1-AS1
Vector
PANC-1
MACC1-AS1
MACC1-AS1+Gem
Vector
Vector+Gem
20-
GPX4

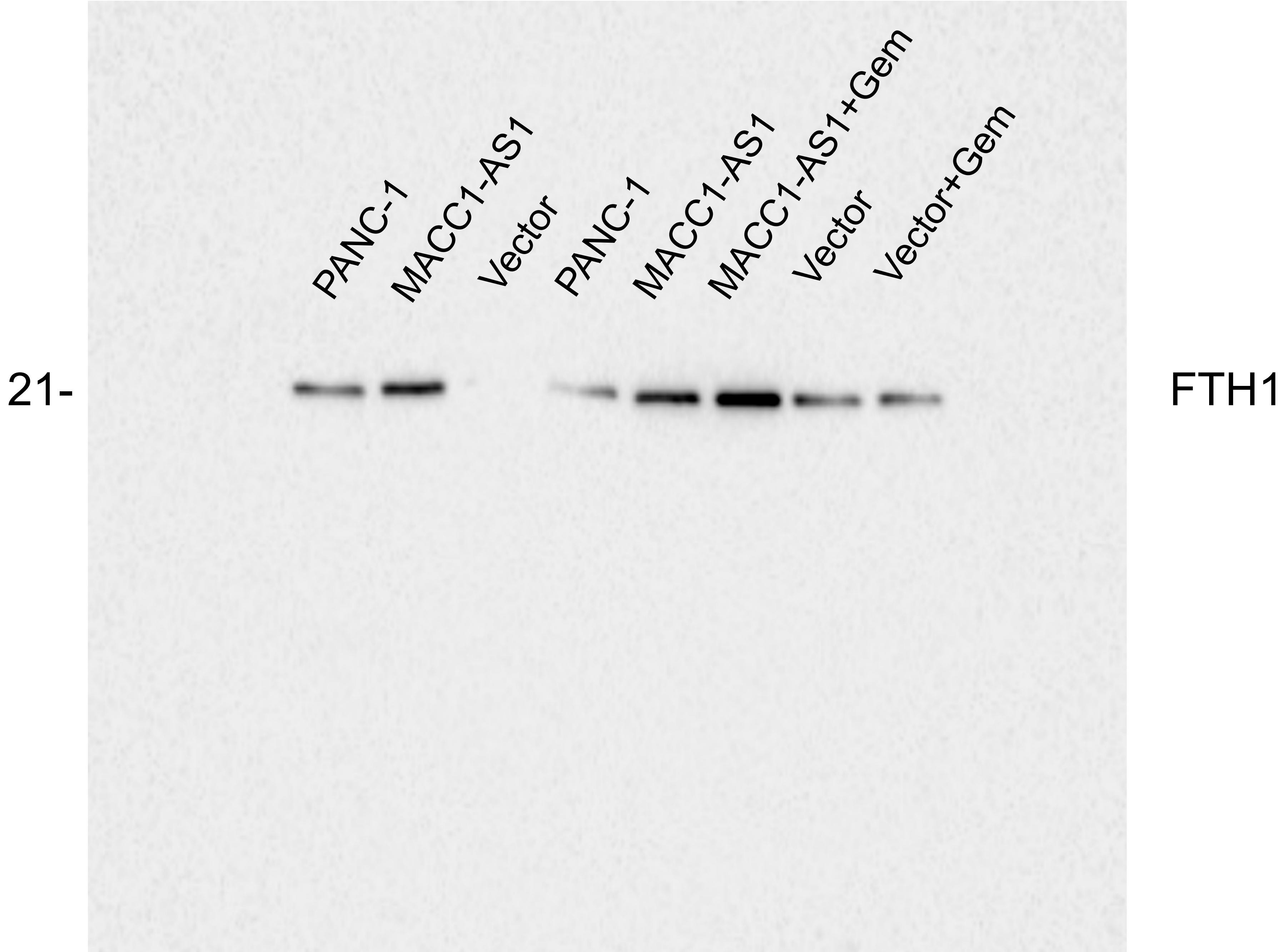

PANC-1
MACC1-AS1
Vector
PANC-1
MACC1-AS1
MACC1-AS1+Gem
Vector
Vector+Gem
21-
FTH1

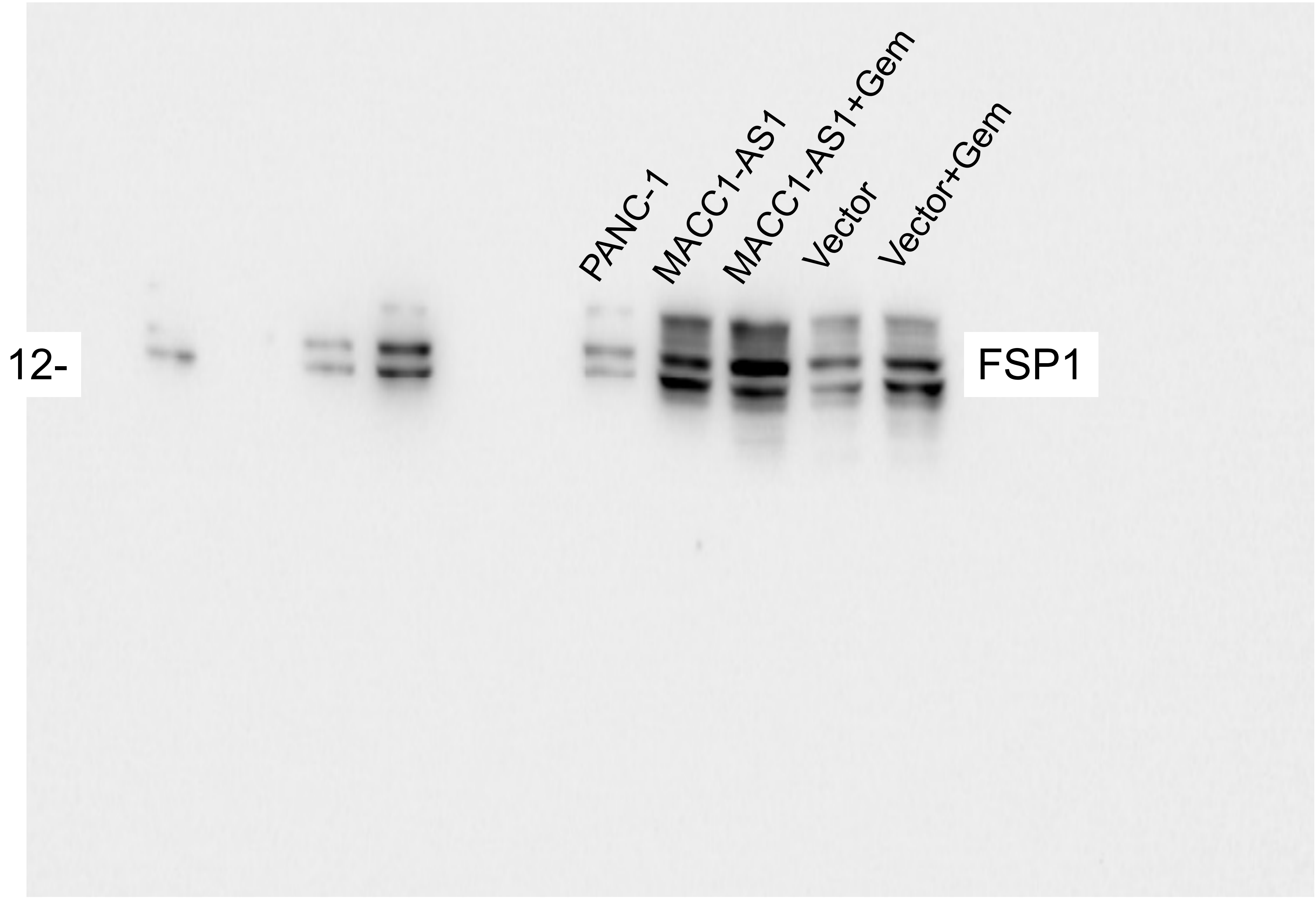
PANC-1
MACC1-AS1
MACC1-AS1+Gem
Vector
Vector+Gem
12-
FSP1

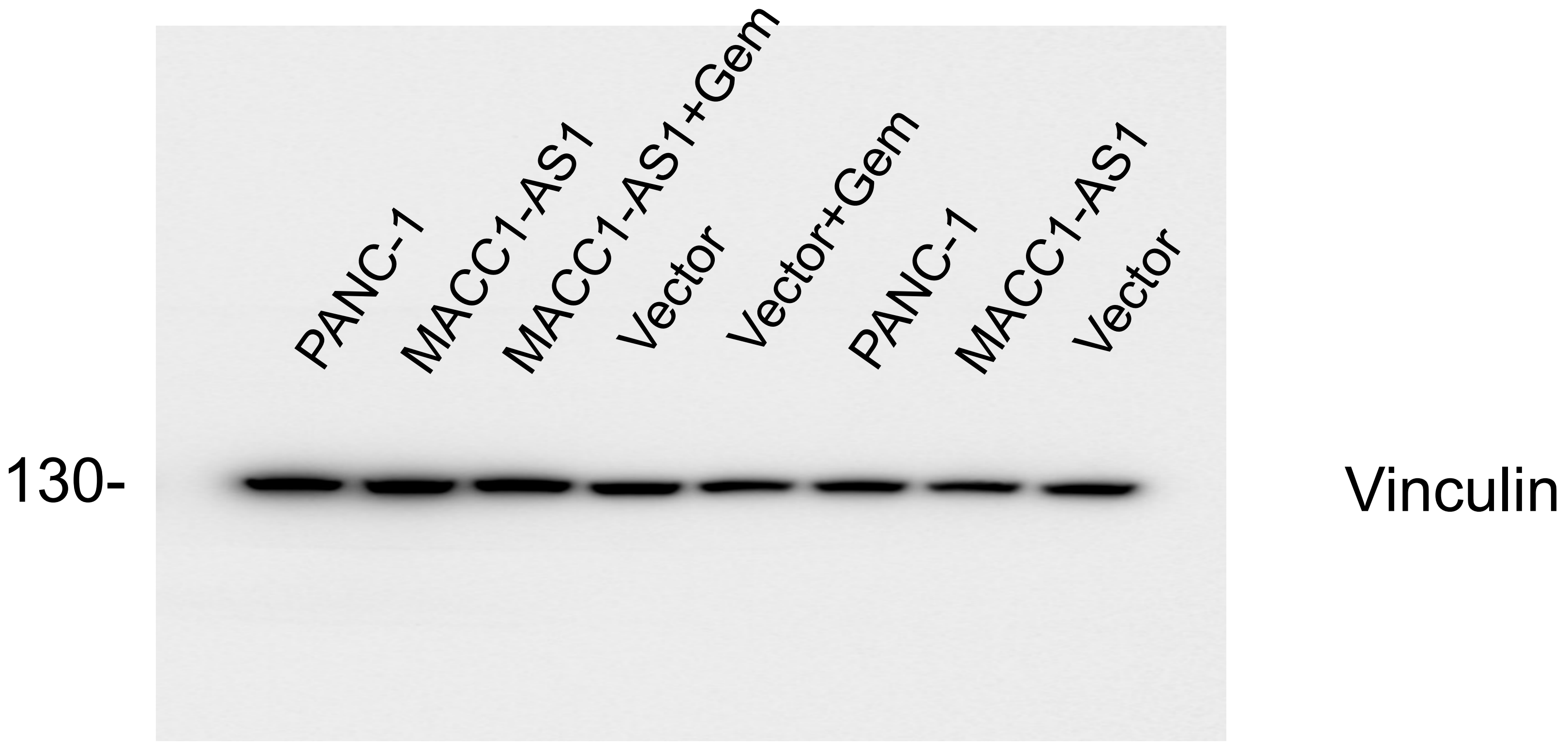
PANC-1
MACC1-AS1
MACC1-AS1+Gem
Vector
Vector+Gem
PANC-1
MACC1-AS1
Vector
130-
Vinculin

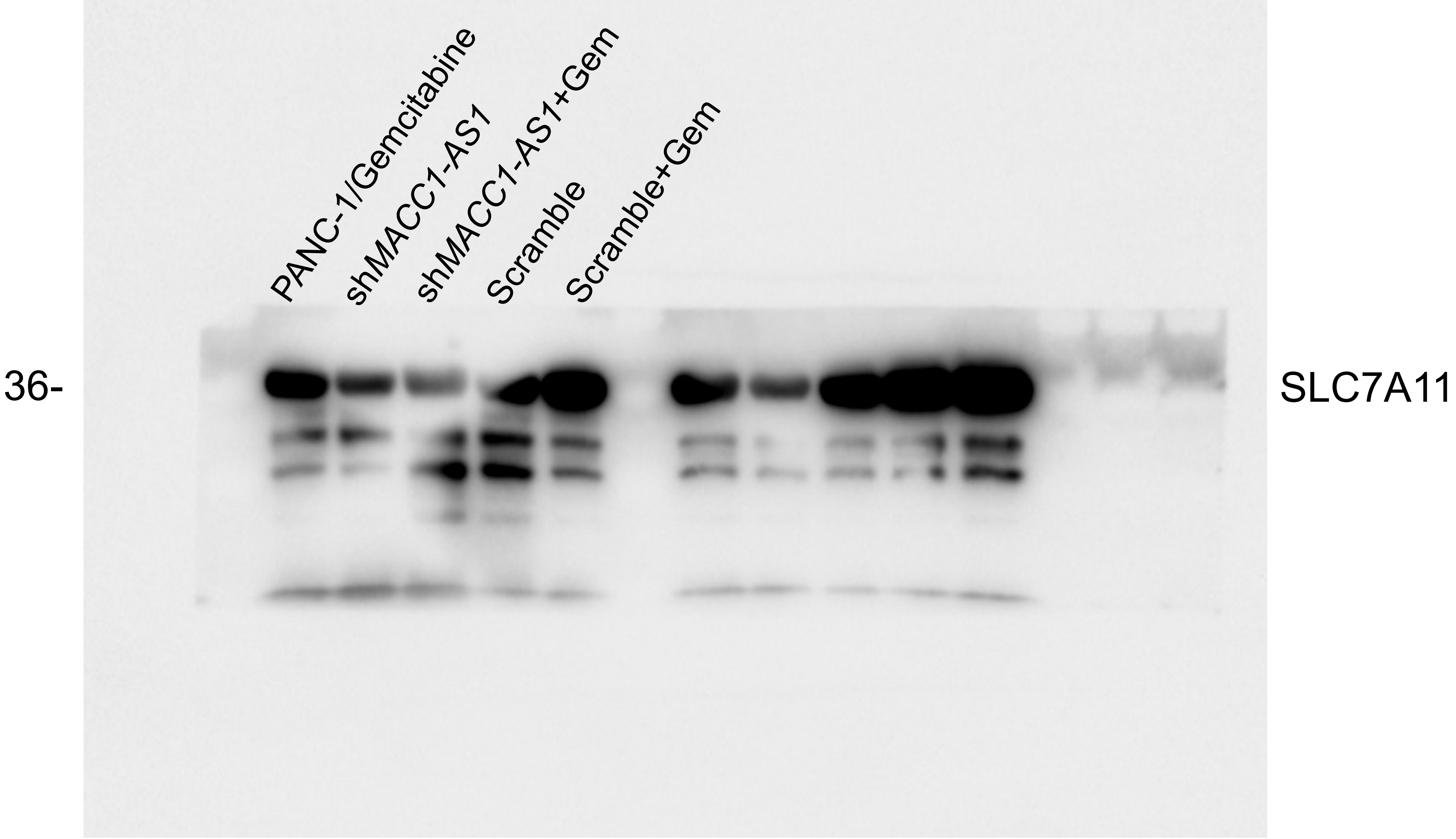

PANC-1/Gemcitabine
shMACC1-AS1
shMACC1-AS1+Gem
Scramble
Scramble+Gem
36-
SLC7A11

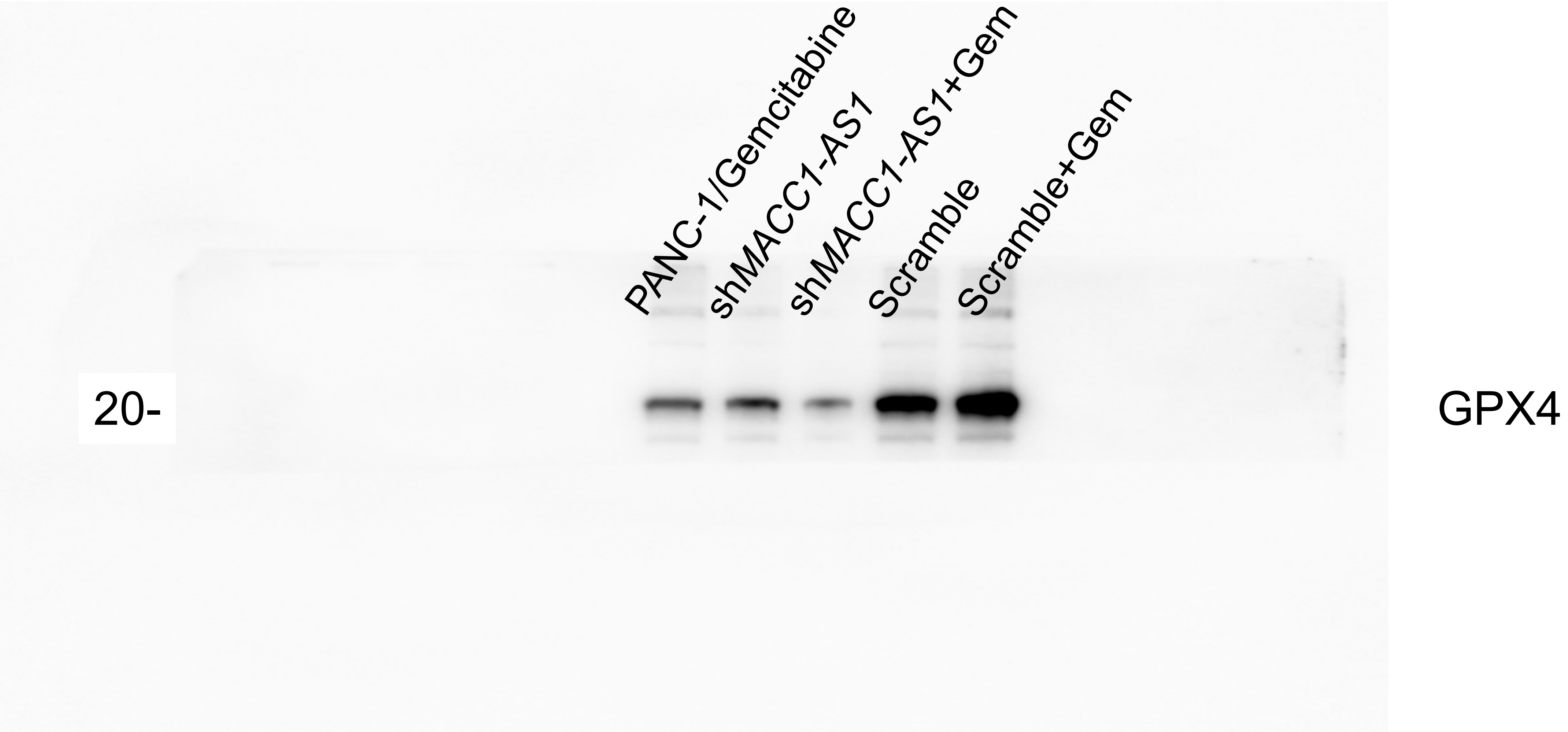
PANC-1/Gemcitabine
shMACC1-AS1
shMACC1-AS1+Gem
Scramble
Scramble+Gem
20-
GPX4

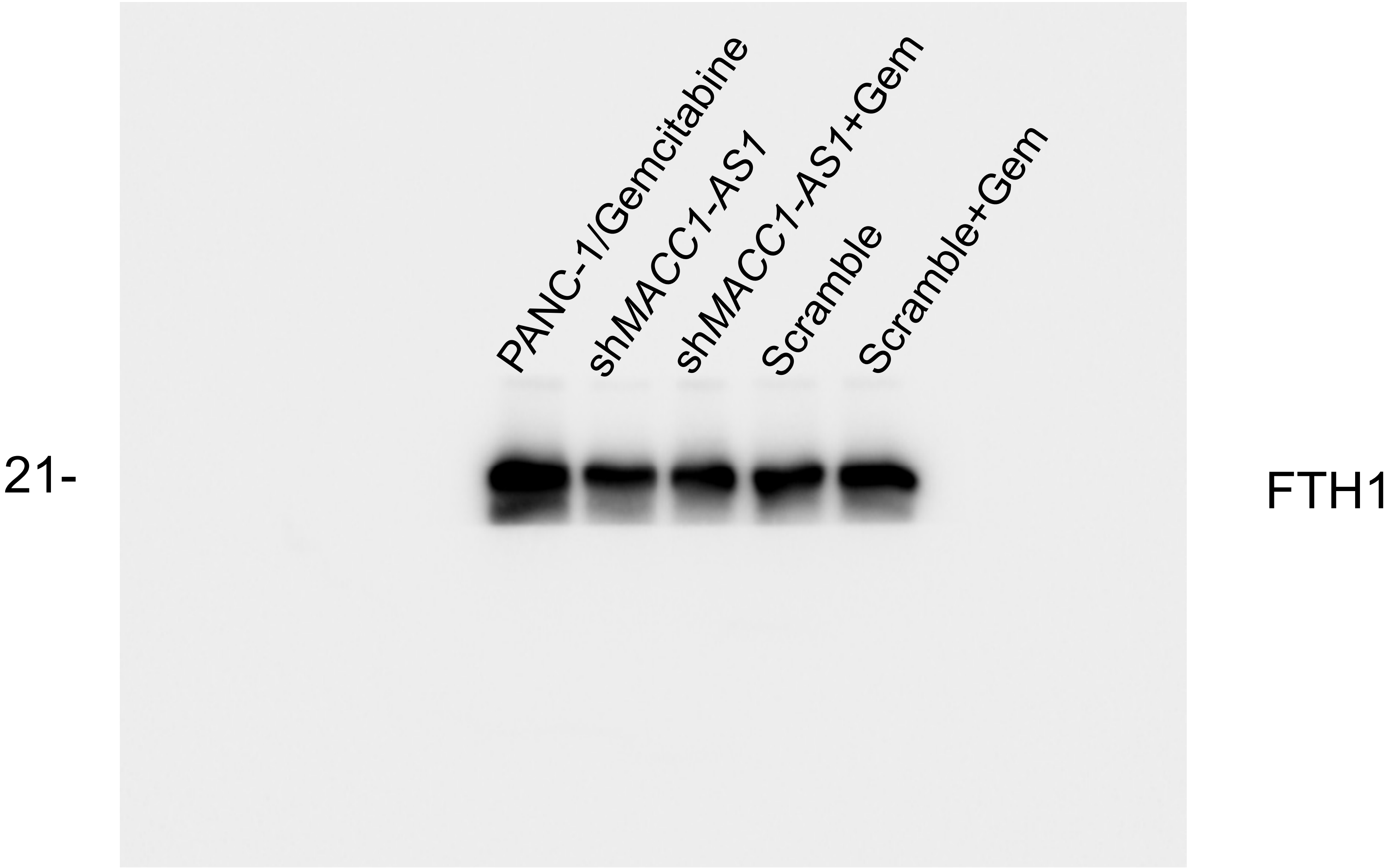
PANC-1/Gemcitabine
shMACC1-AS1
shMACC1-AS1+Gem
Scramble
Scramble+Gem
21-
FTH1

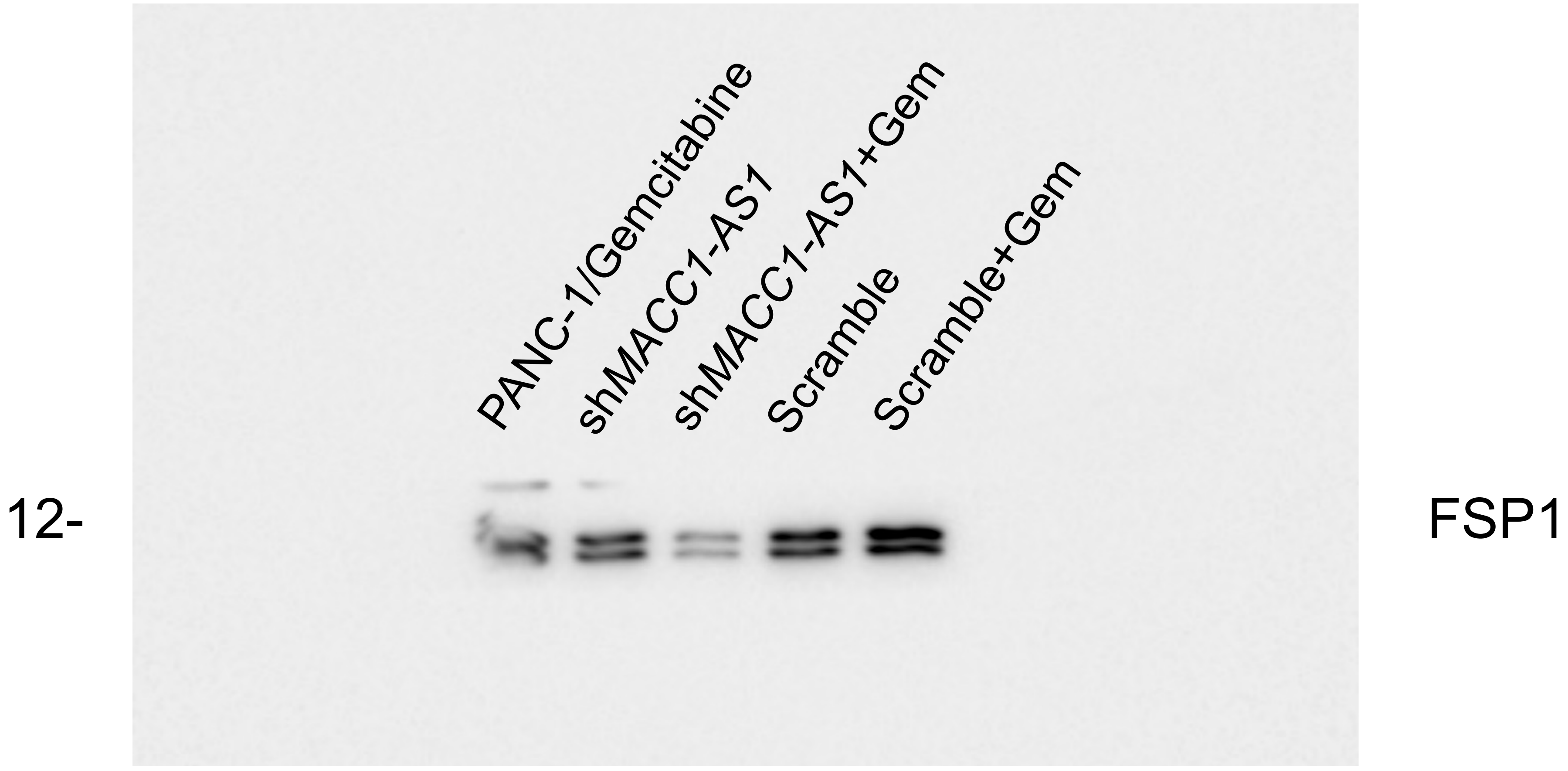
PANC-1/Gemcitabine
shMACC1-AS1
shMACC1-AS1+Gem
Scramble
Scramble+Gem
12-
FSP1

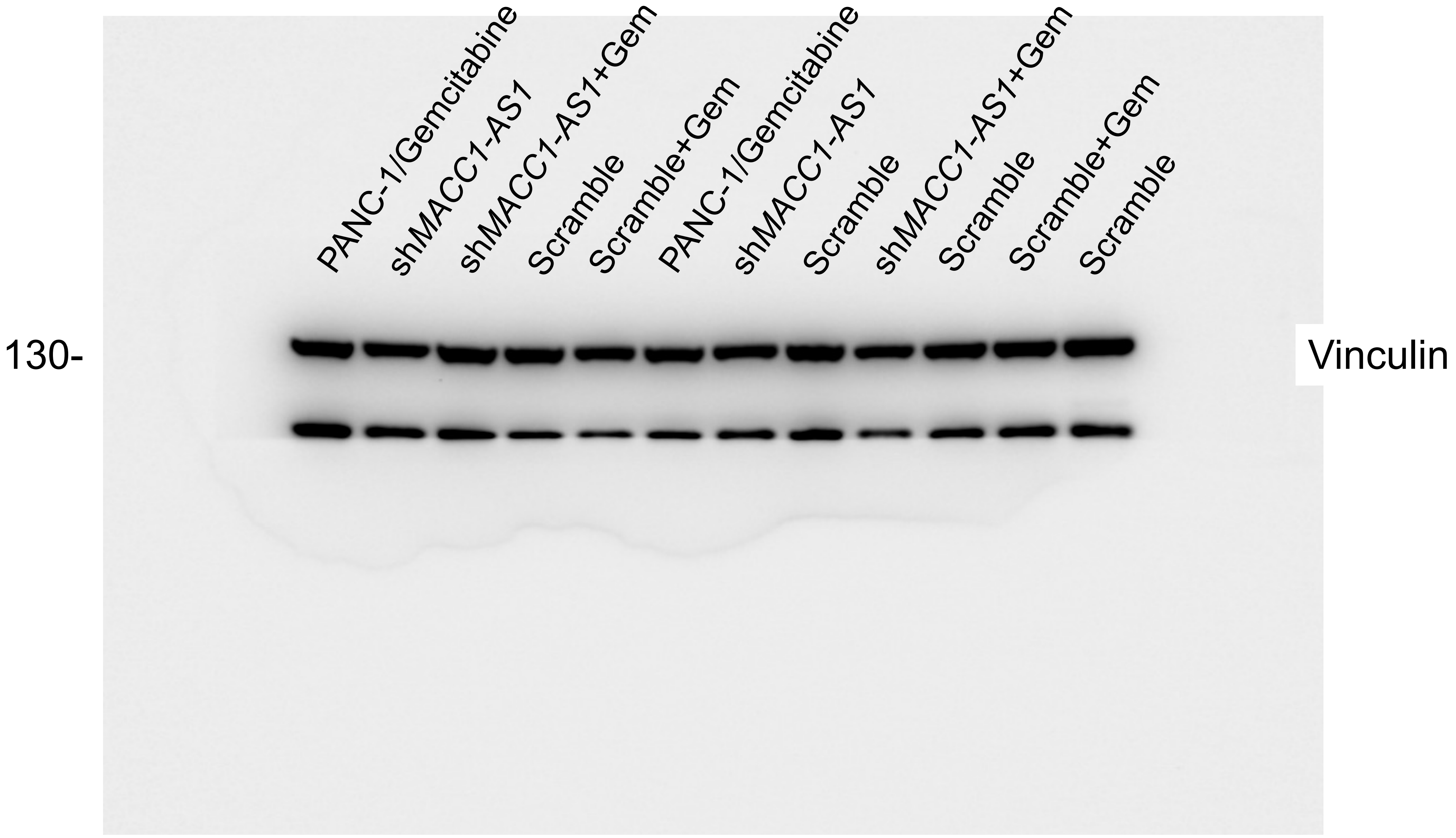

PANC-1/Gemcitabine
shMACC1-AS1
shMACC1-AS1+Gem
Scramble
Scramble+Gem
PANC-1/Gemcitabine
shMACC1-AS1
Scramble
shMACC1-AS1+Gem
Scramble
Scramble+Gem
Scramble
130-
Vinculin

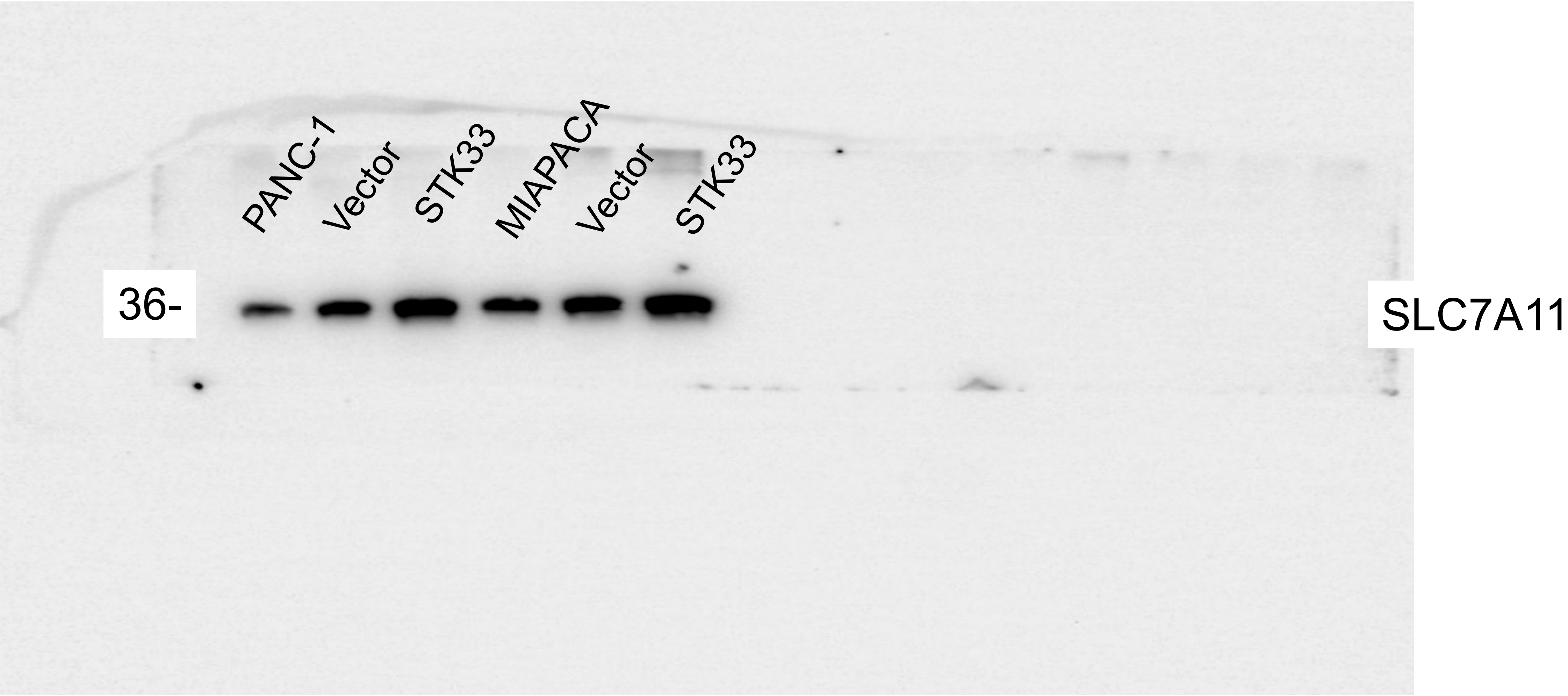
PANC-1
Vector
STK33
MIAPACA
Vector
STK33
36-
SLC7A11

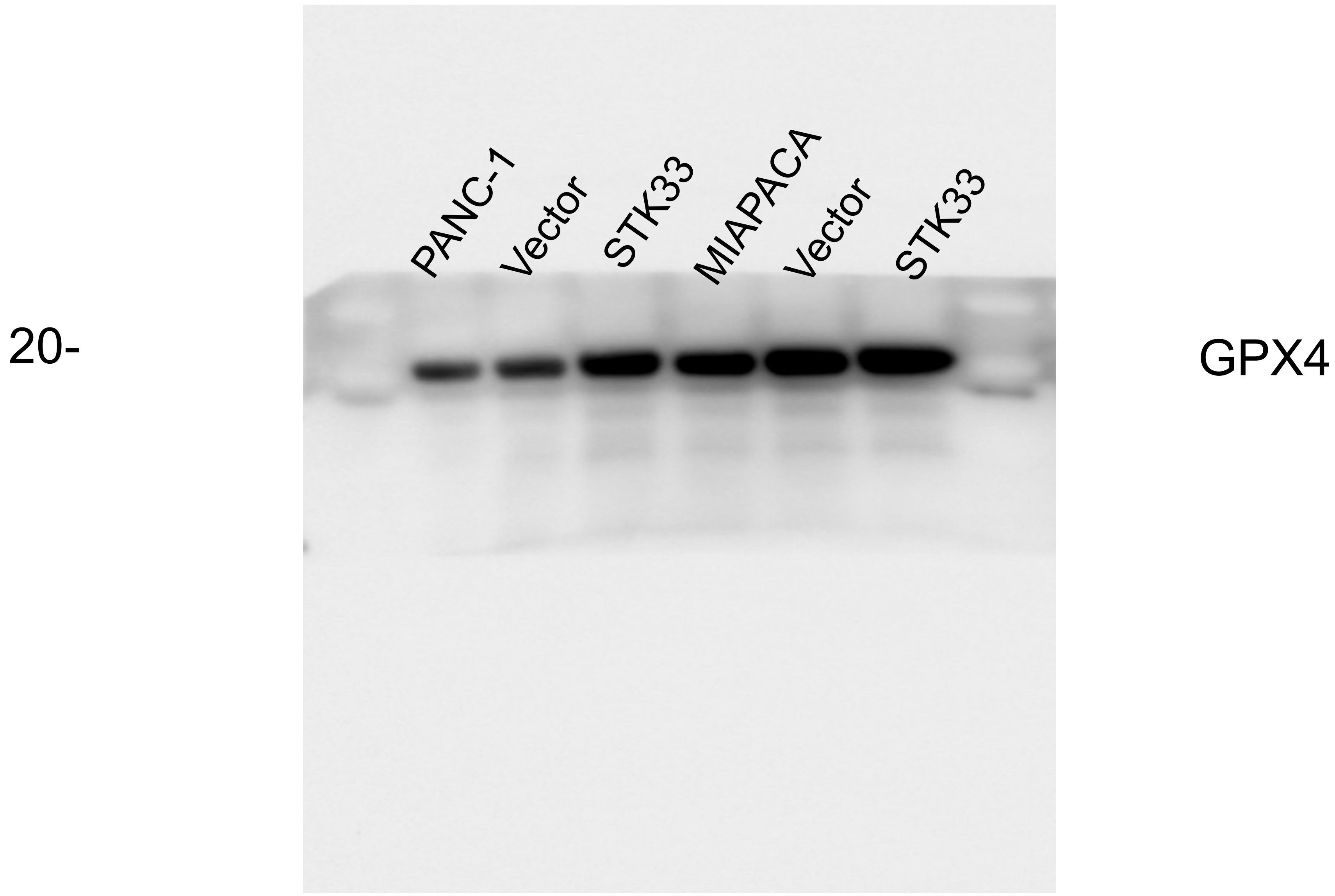
PANC-1
Vector
STK33
MIAPACA
Vector
STK33
20-
GPX4

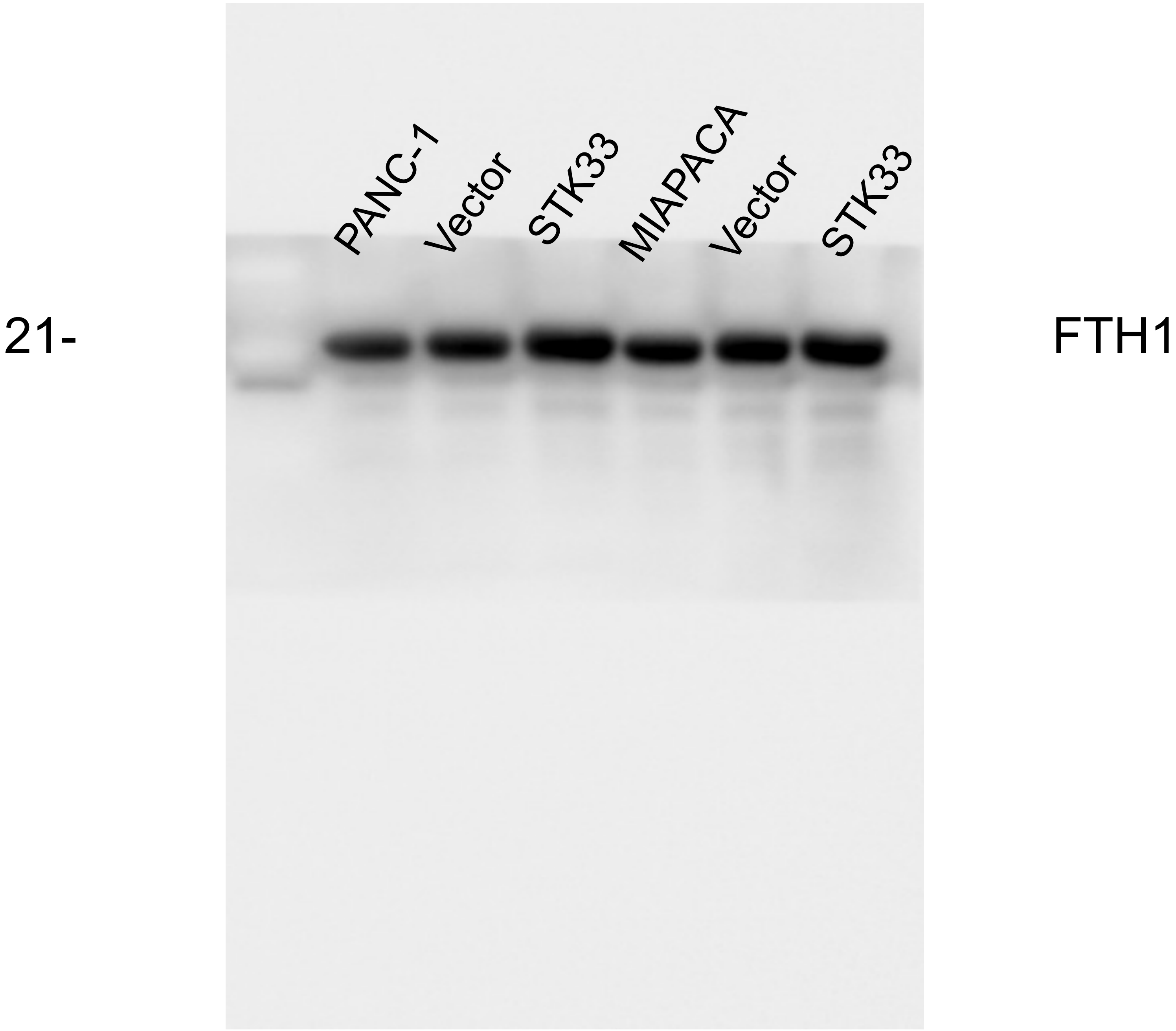
PANC-1
Vector
STK33
MIAPACA
Vector
STK33
21-
FTH1

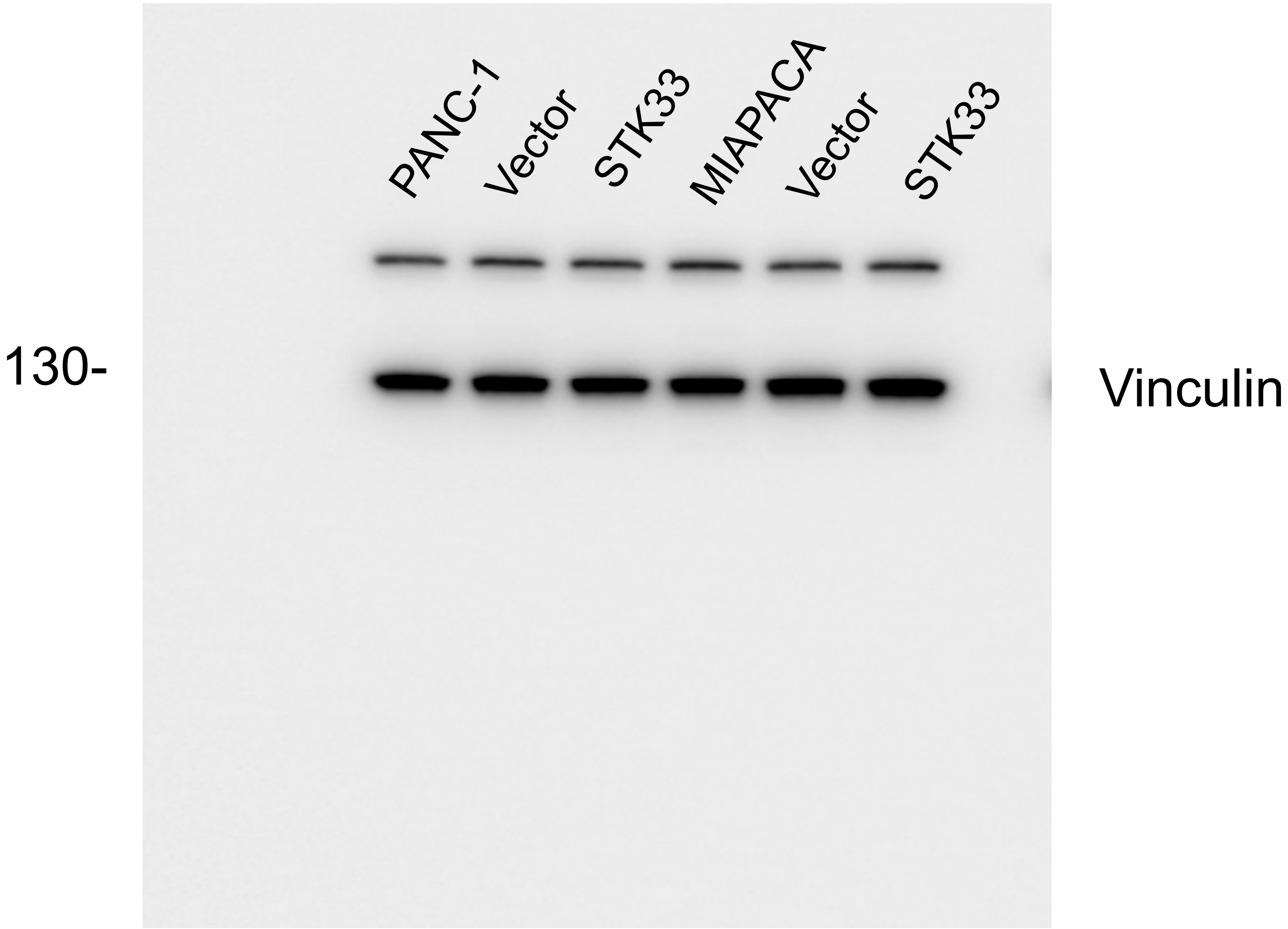
PANC-1
Vector
STK33
MIAPACA
Vector
STK33
130-
Vinculin

Figure 5

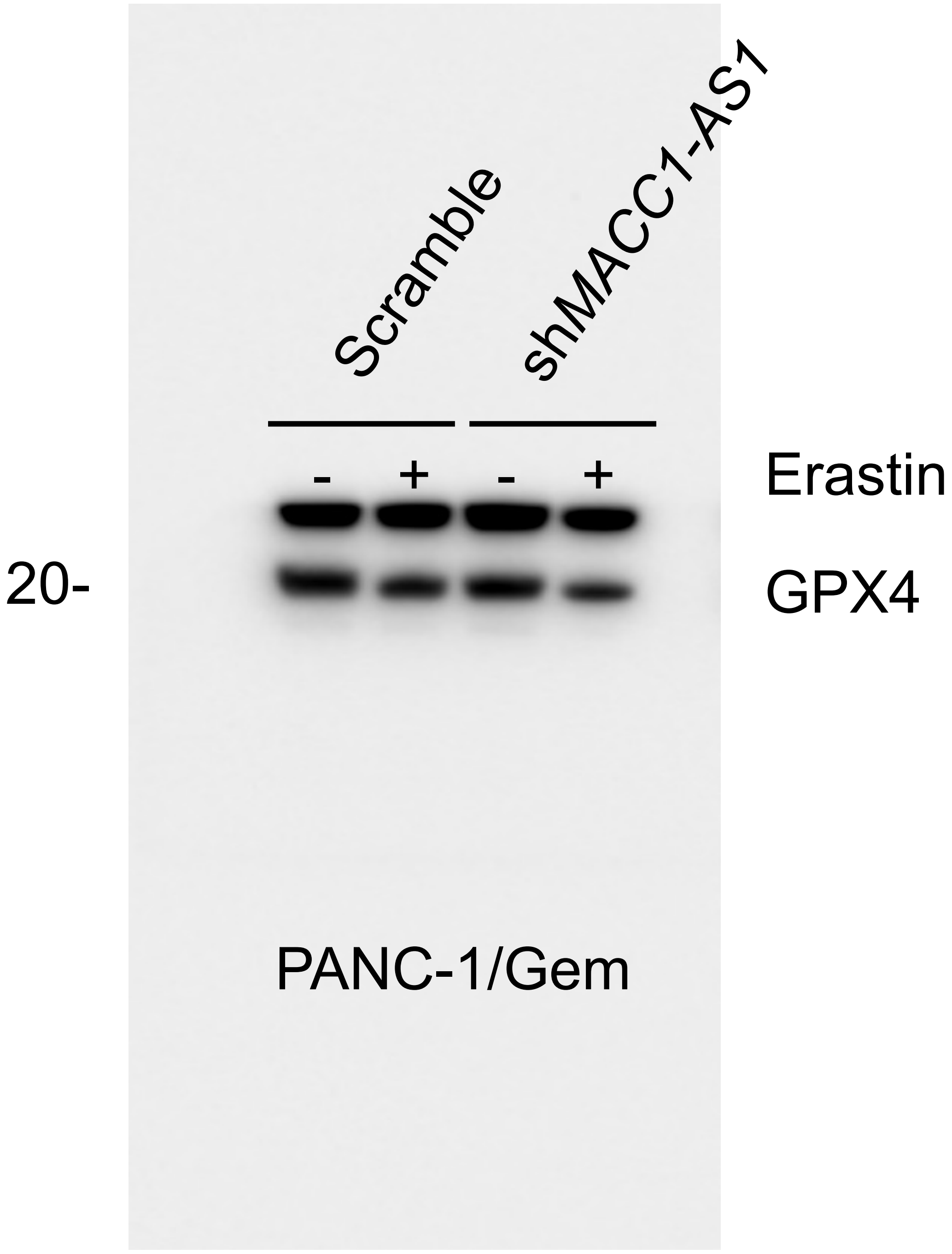
Scramble
shMACC1-AS1
- + - +
Erastin
20-
GPX4
PANC-1/Gem

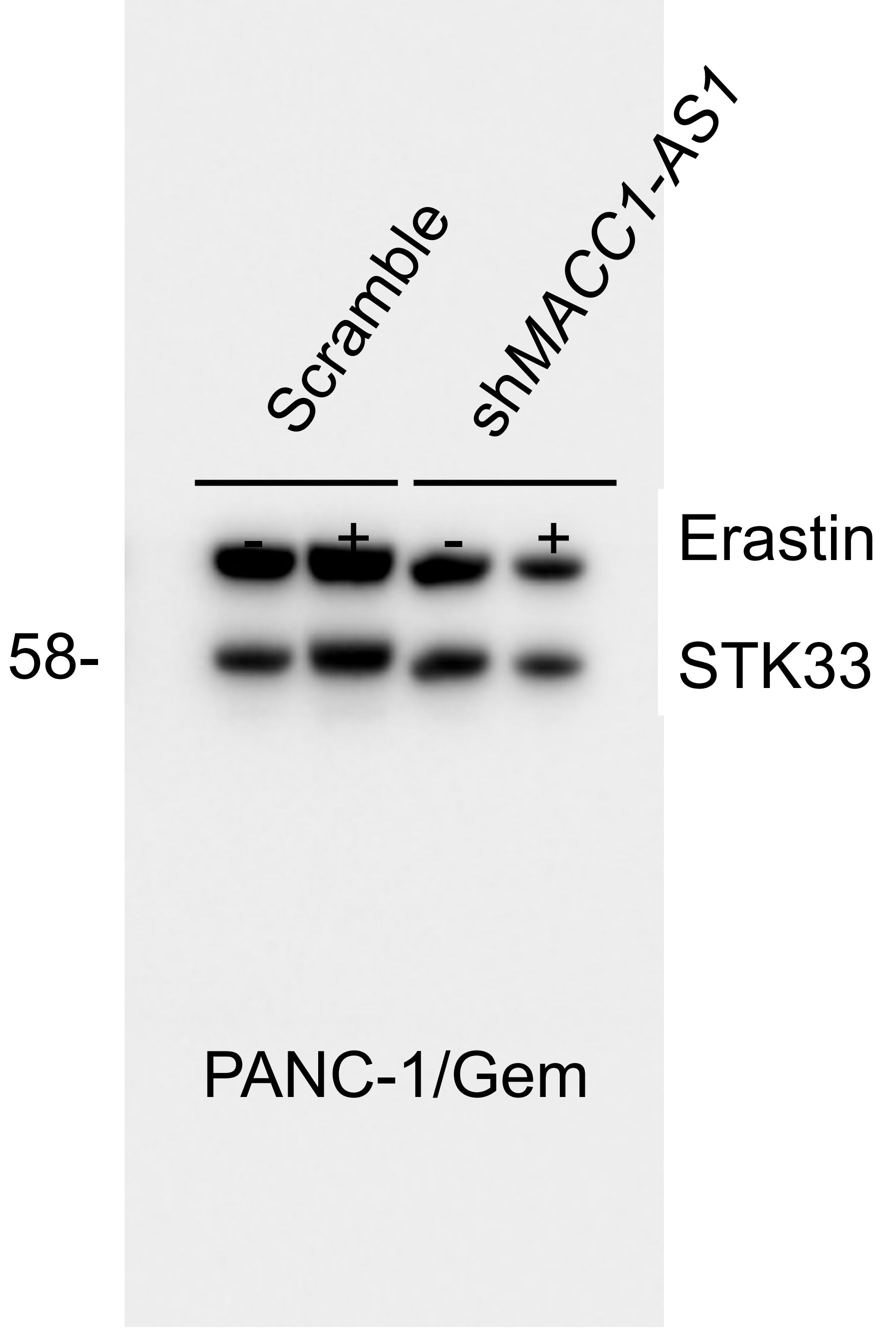
Scramble
shMACC1-AS1
- + - +
Erastin
58-
STK33
PANC-1/Gem

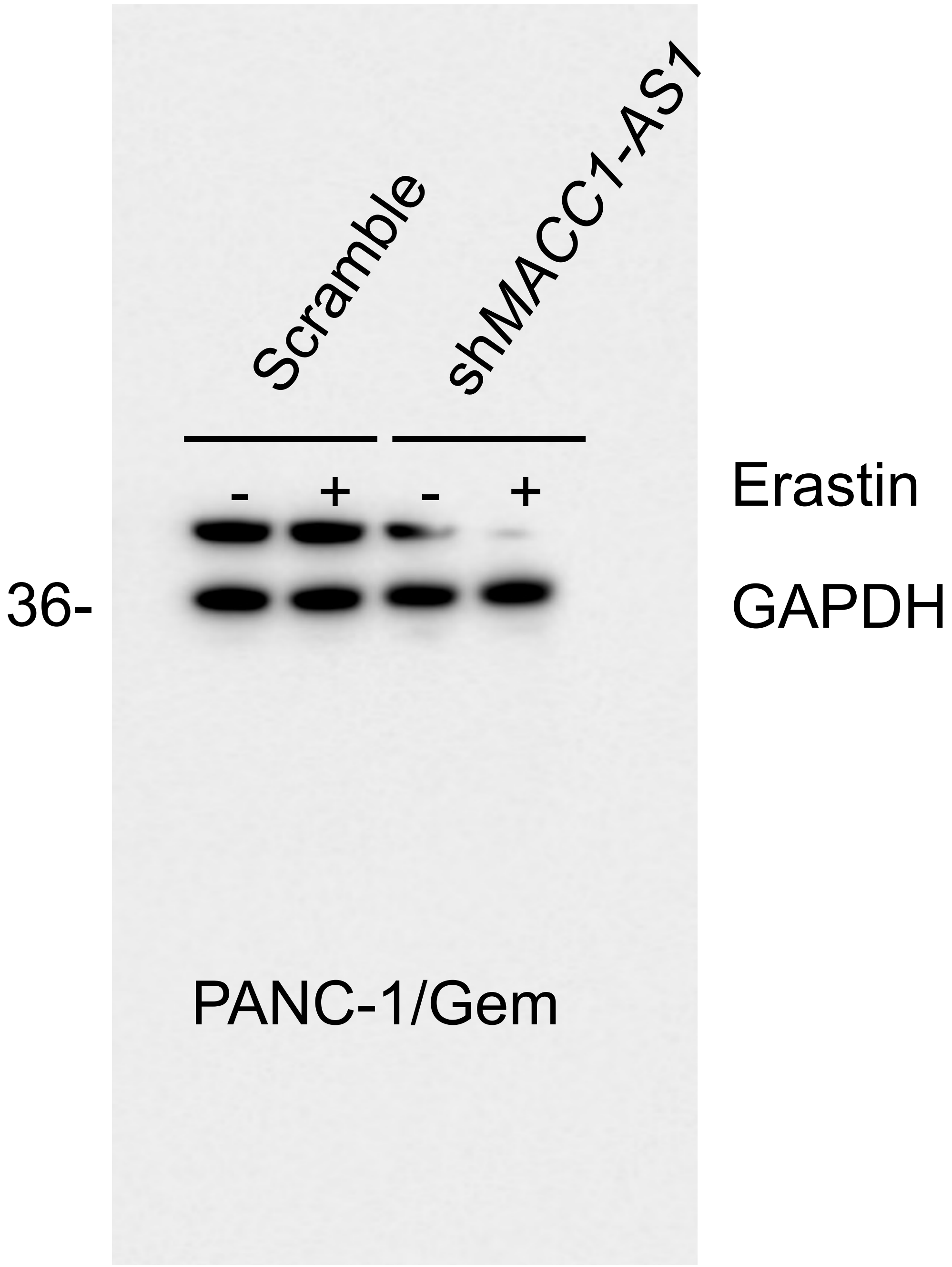
Scramble
shMACC1-AS1
- + - +
Erastin
36-
GAPDH
PANC-1/Gem

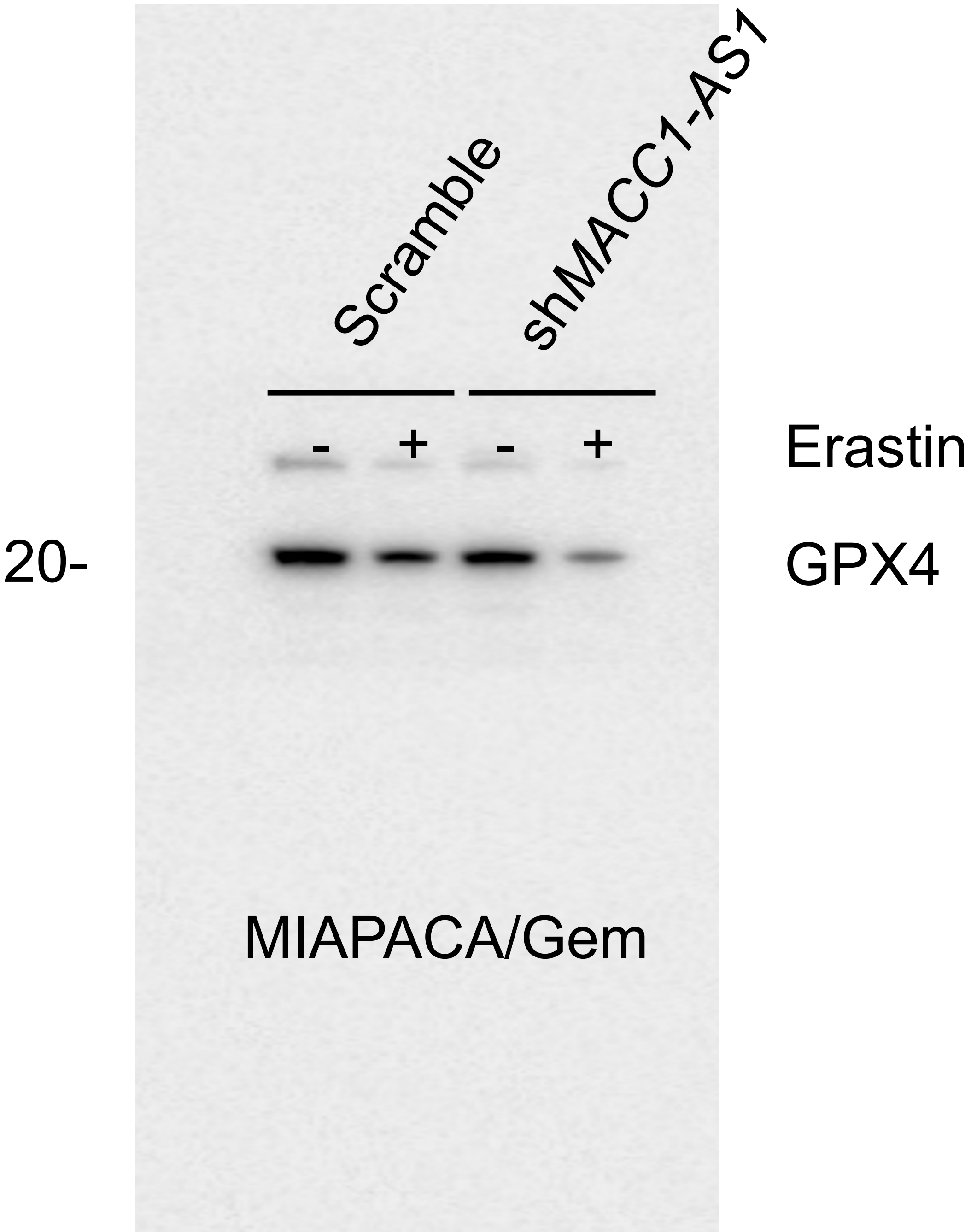
Scramble
shMACC1-AS1
- + - + Erastin
20-
GPX4
MIAPACA/Gem

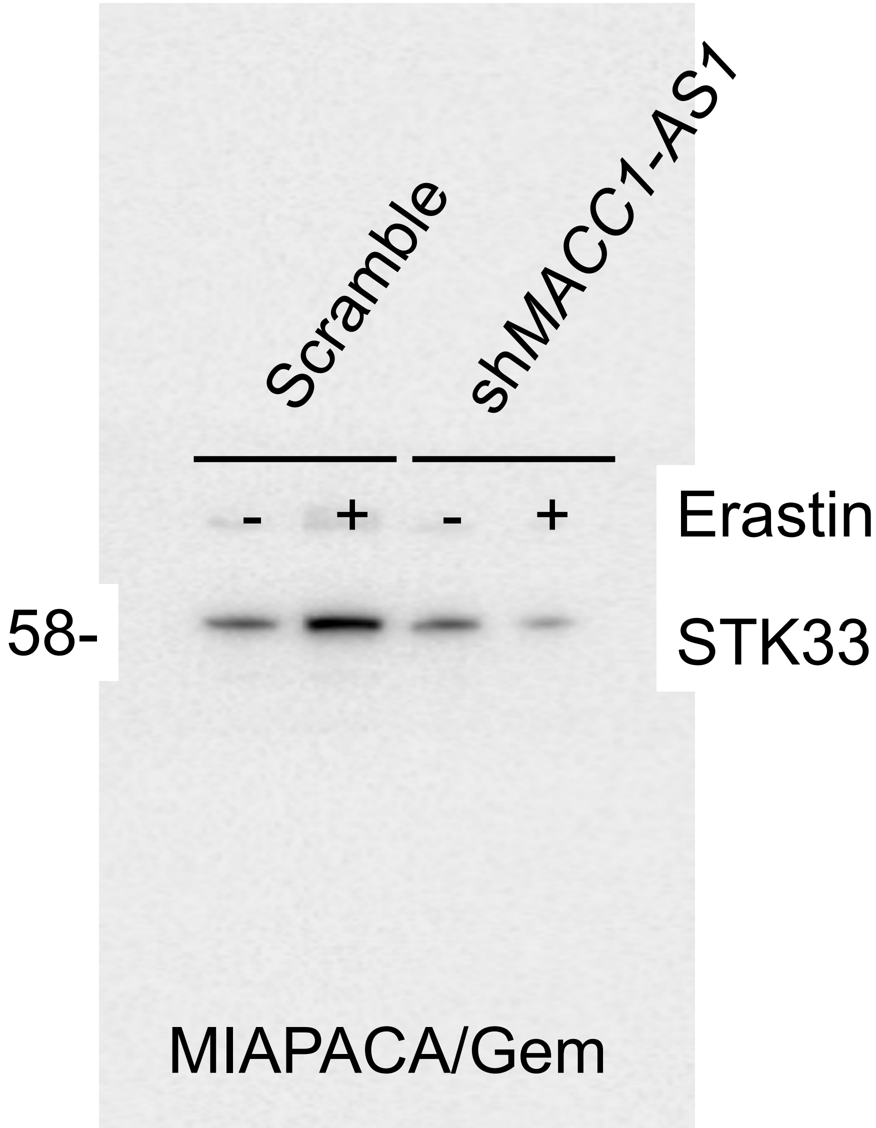

Scramble
shMACC1-AS1
- + - +
Erastin
58-
STK33
MIAPACA/Gem

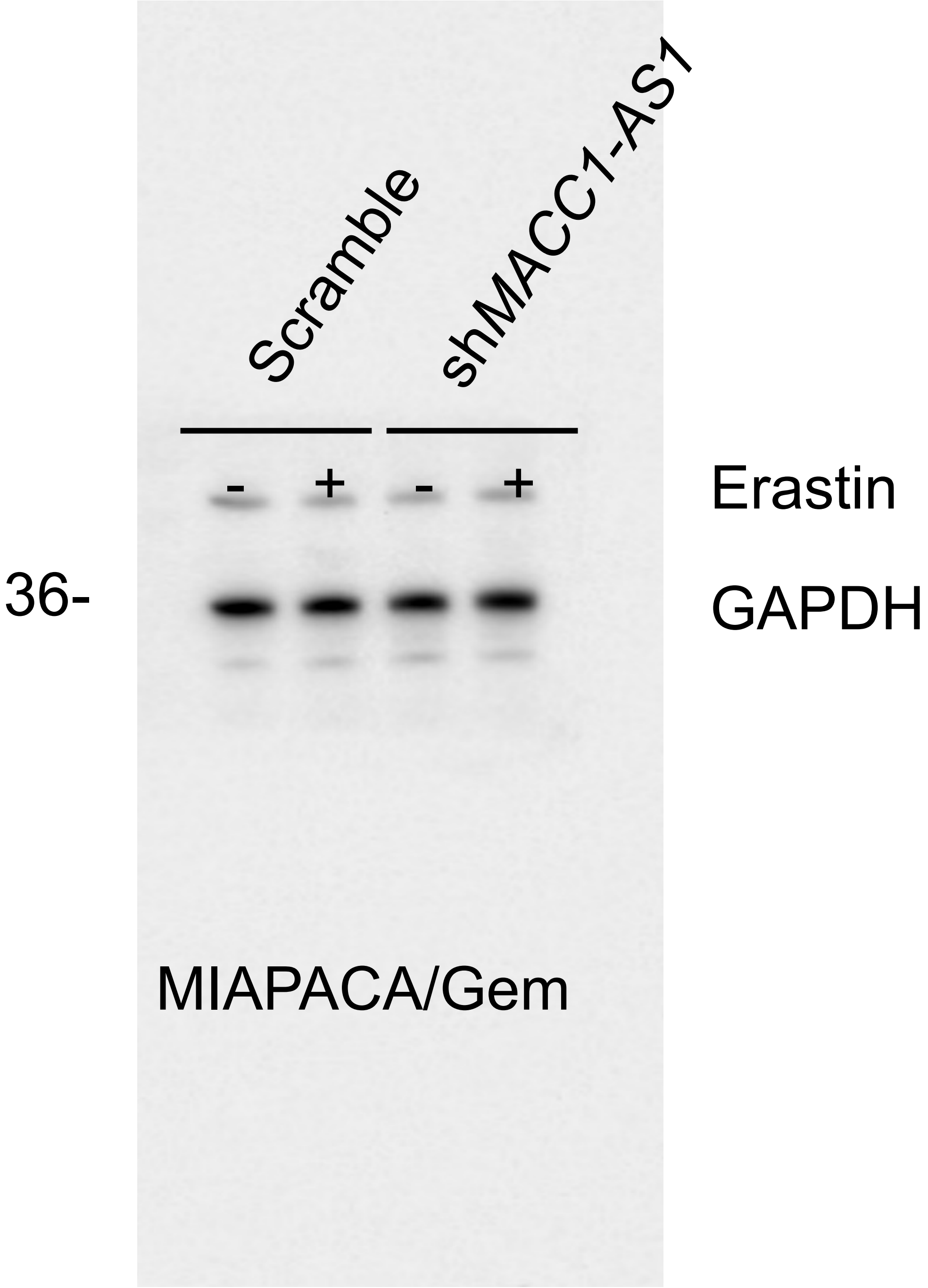
Scramble
shMACC1-AS1
- + - +
Erastin
36-
GAPDH
MIAPACA/Gem

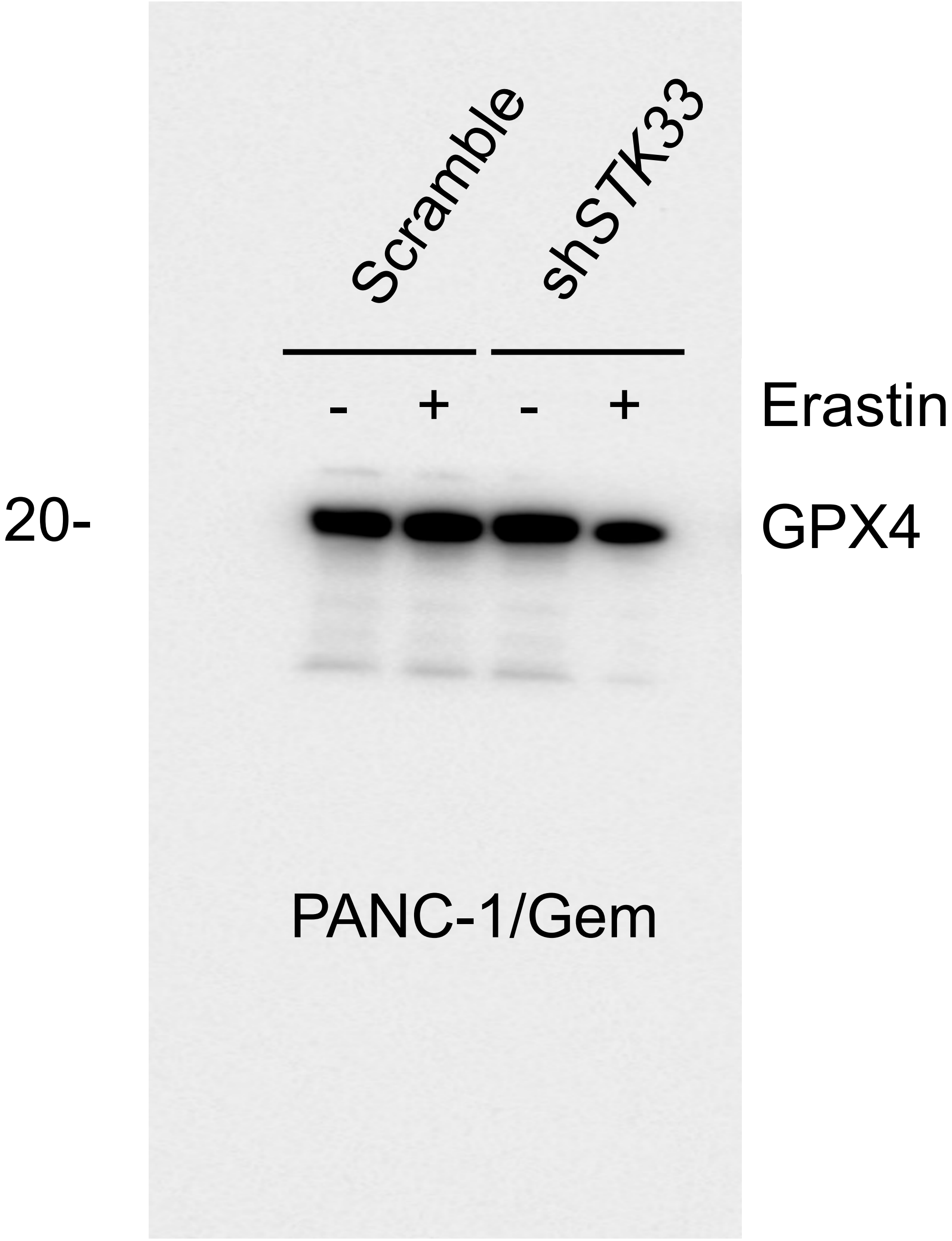

Scramble
shSTK33
- + - +
Erastin
20-
GPX4
PANC-1/Gem

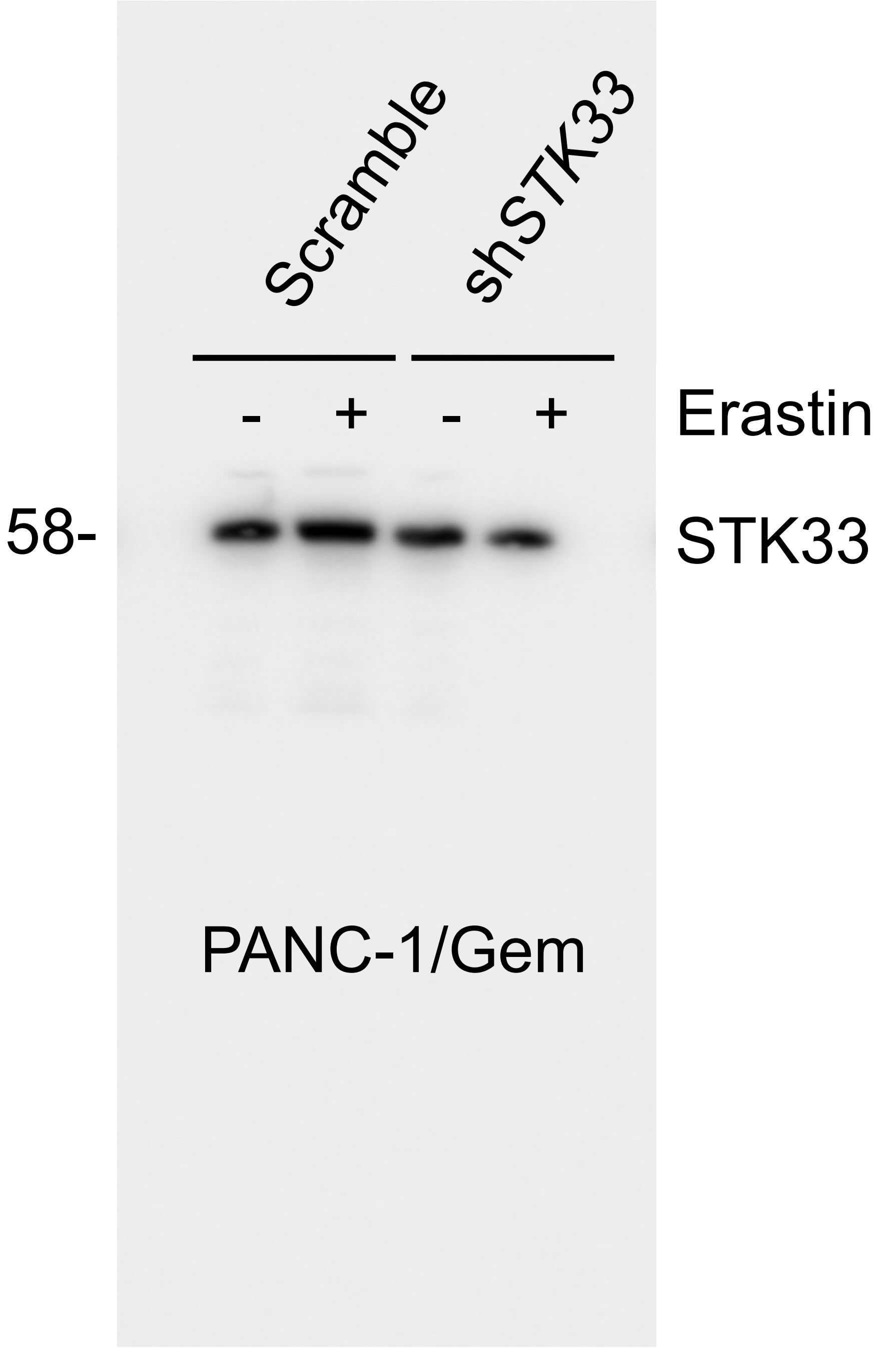

Scramble
shSTK33
- + - +
Erastin
58-
STK33
PANC-1/Gem

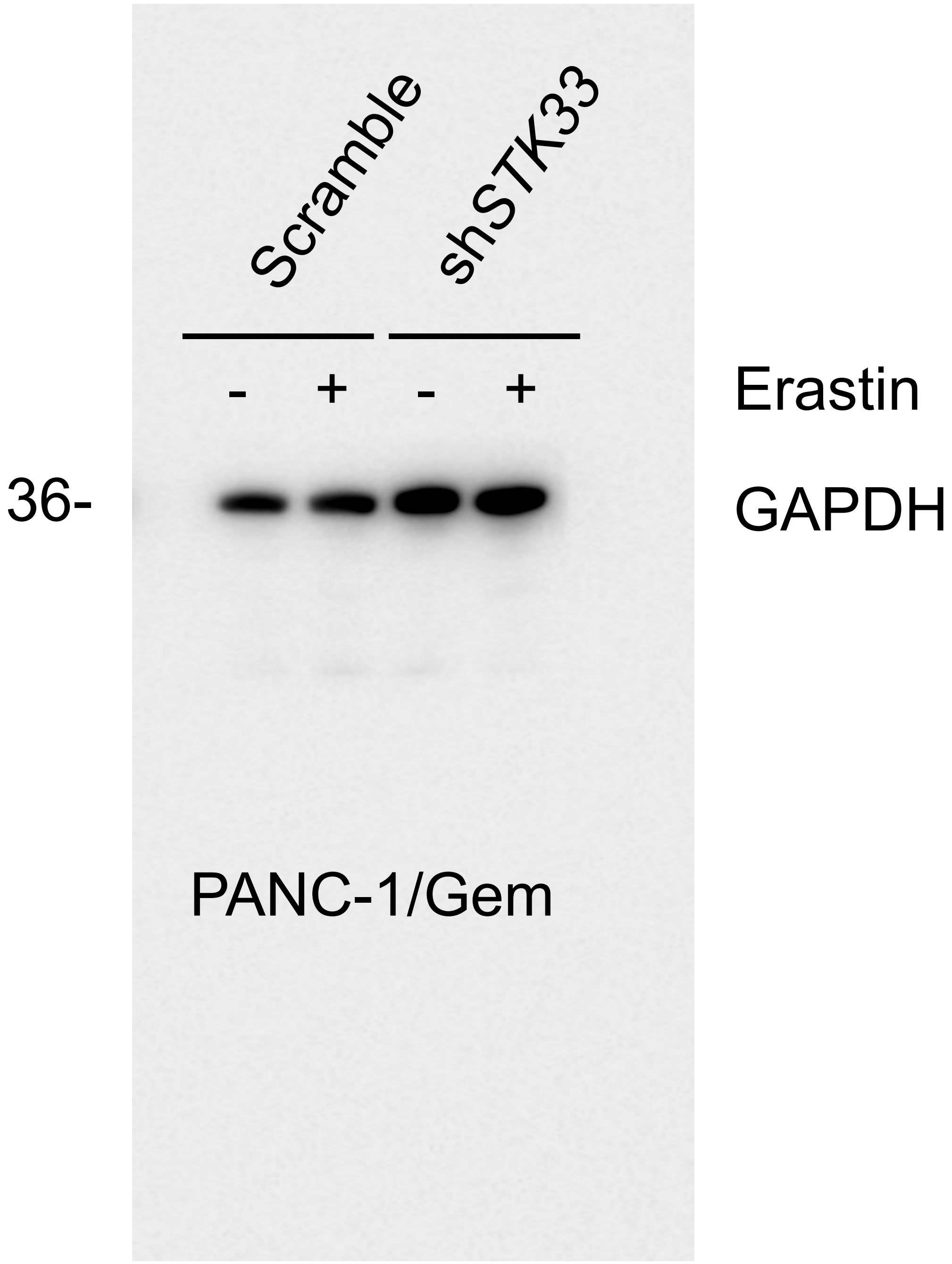

Scramble
shSTK33
- + - +
Erastin
36-
GAPDH
PANC-1/Gem

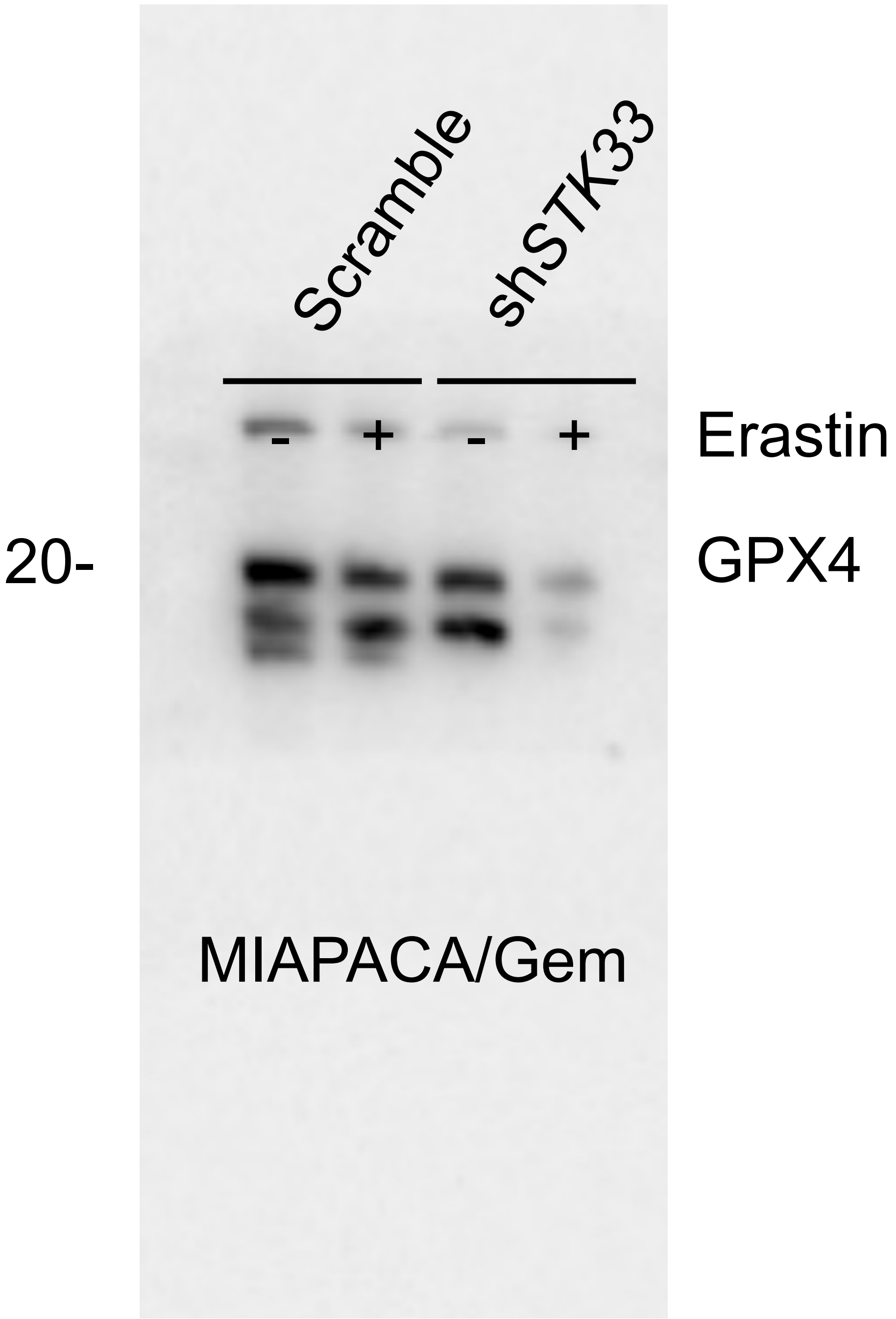
Scramble
shSTK33
-
+
-
+
Erastin
20-
GPX4
MIAPACA/Gem

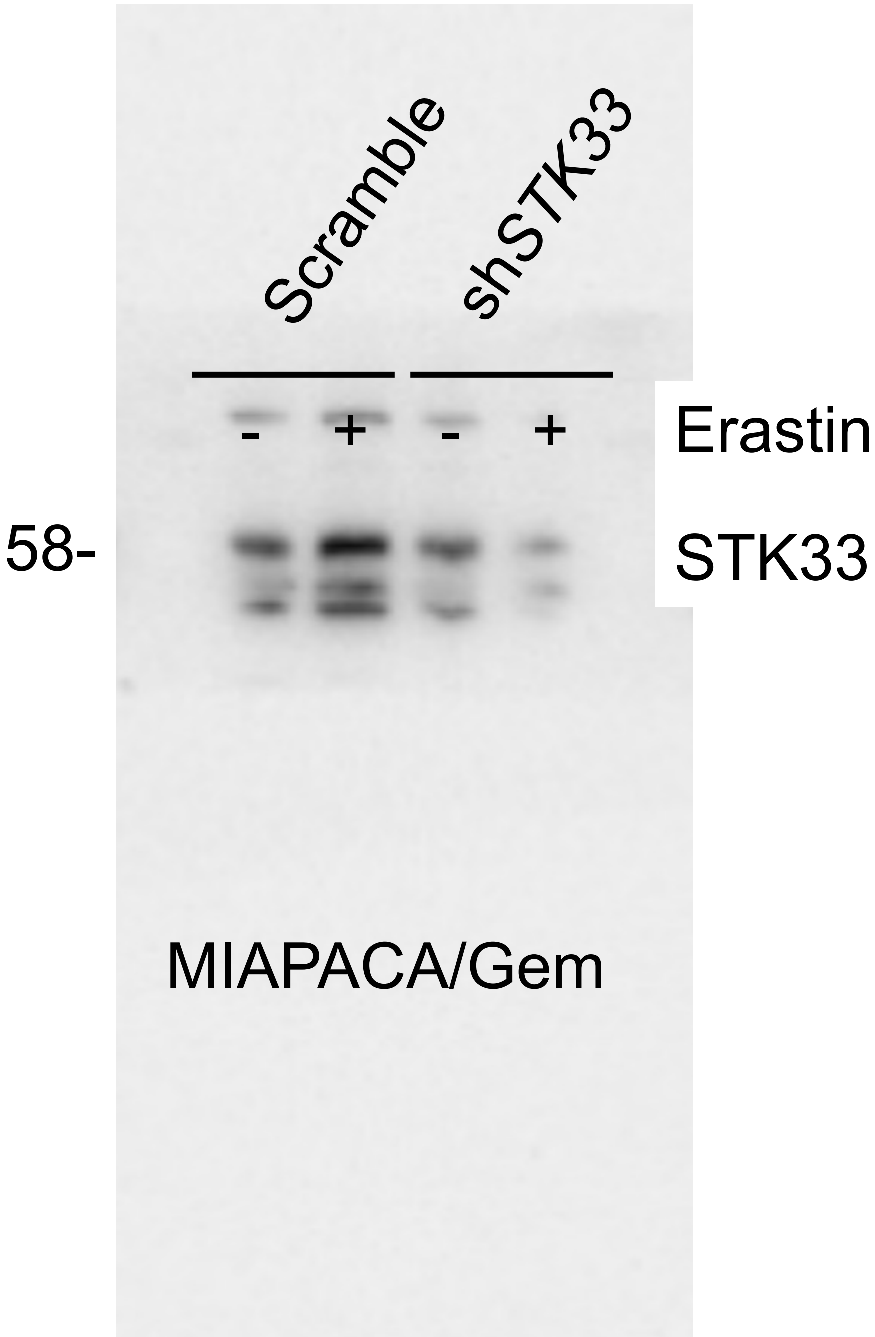
Scramble
shSTK33
- + - +
Erastin
58-
STK33
MIAPACA/Gem

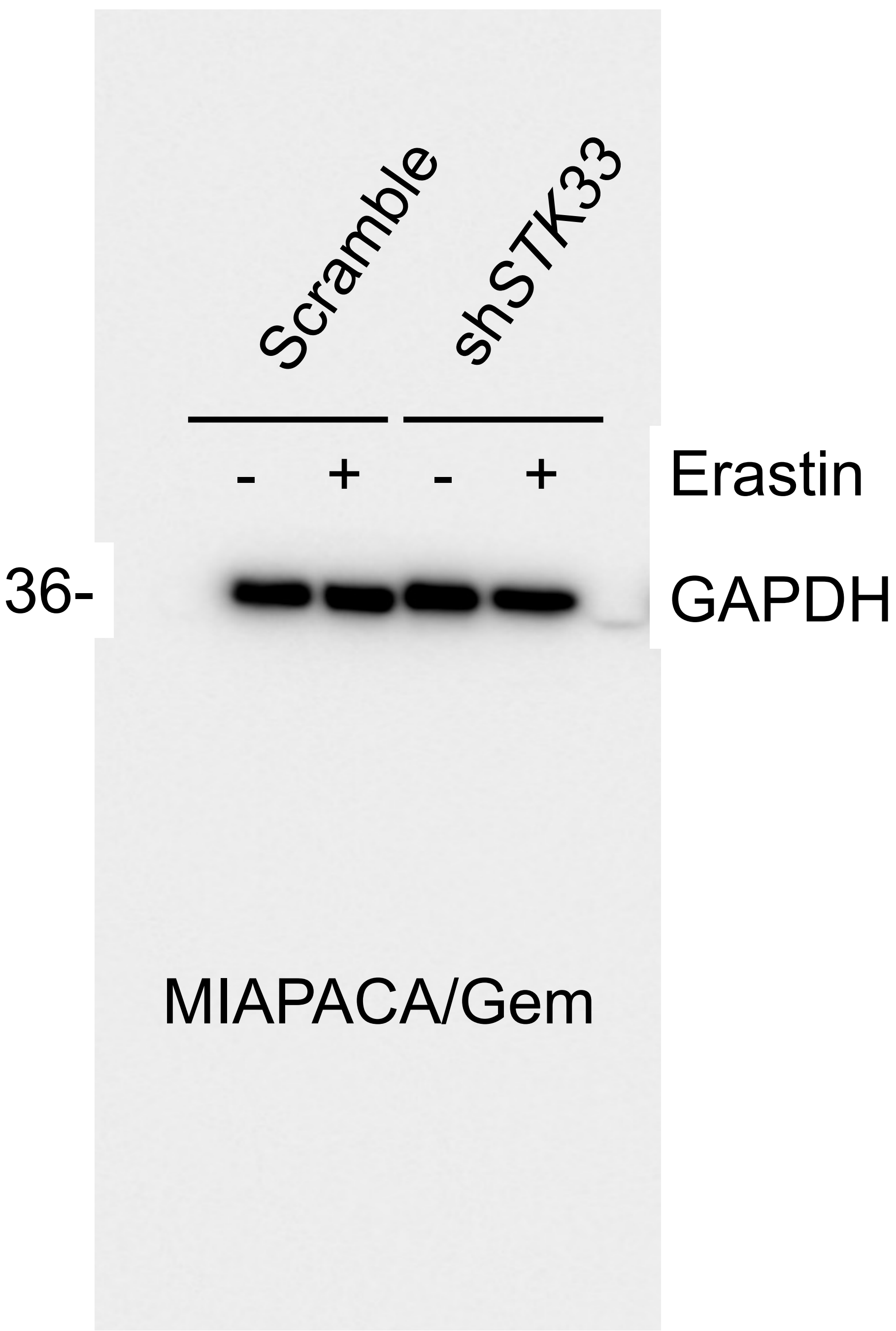
Scramble
shSTK33
- + - +
Erastin
36-
GAPDH
MIAPACA/Gem

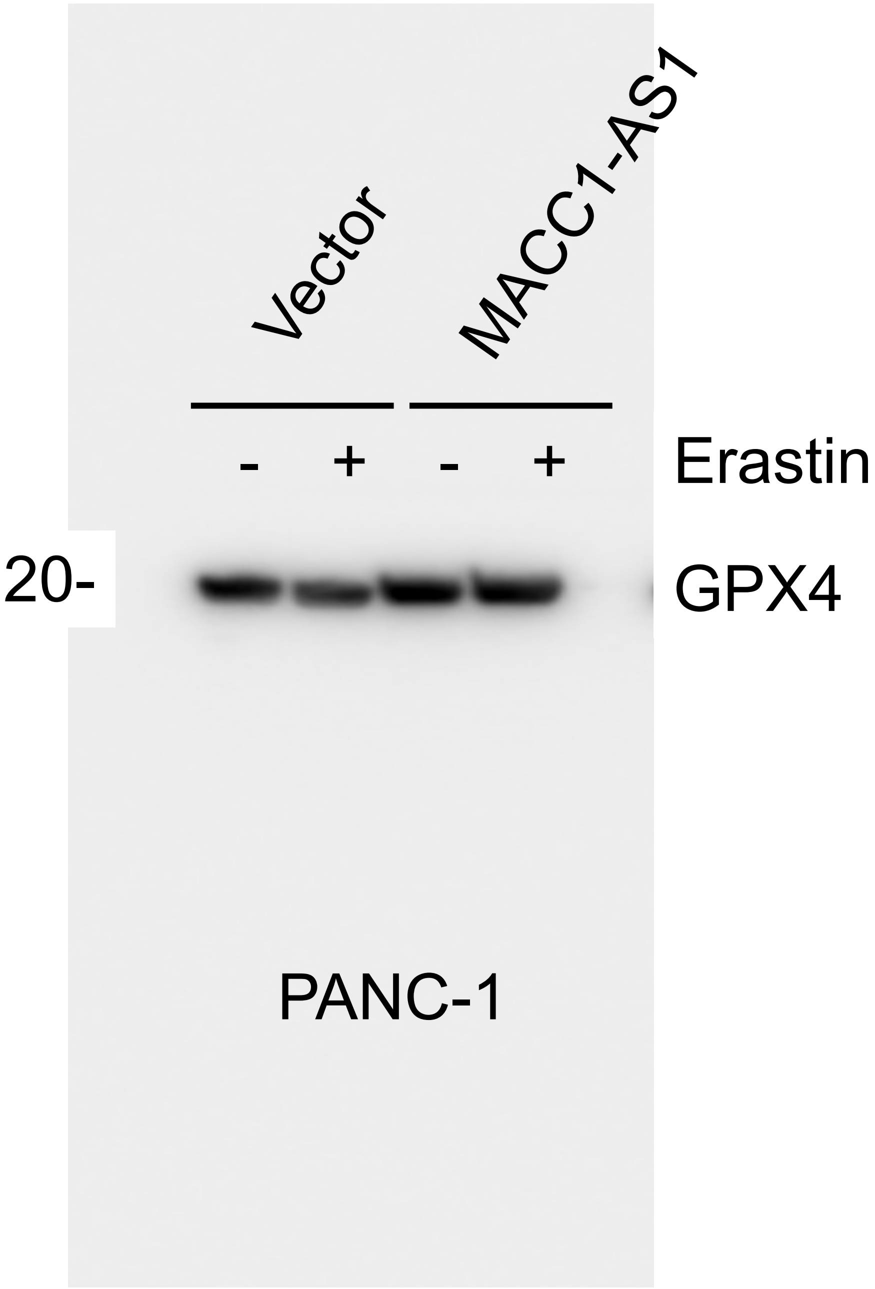
Vector
MACC1-AS1
- + - +
Erastin
20-
GPX4
PANC-1

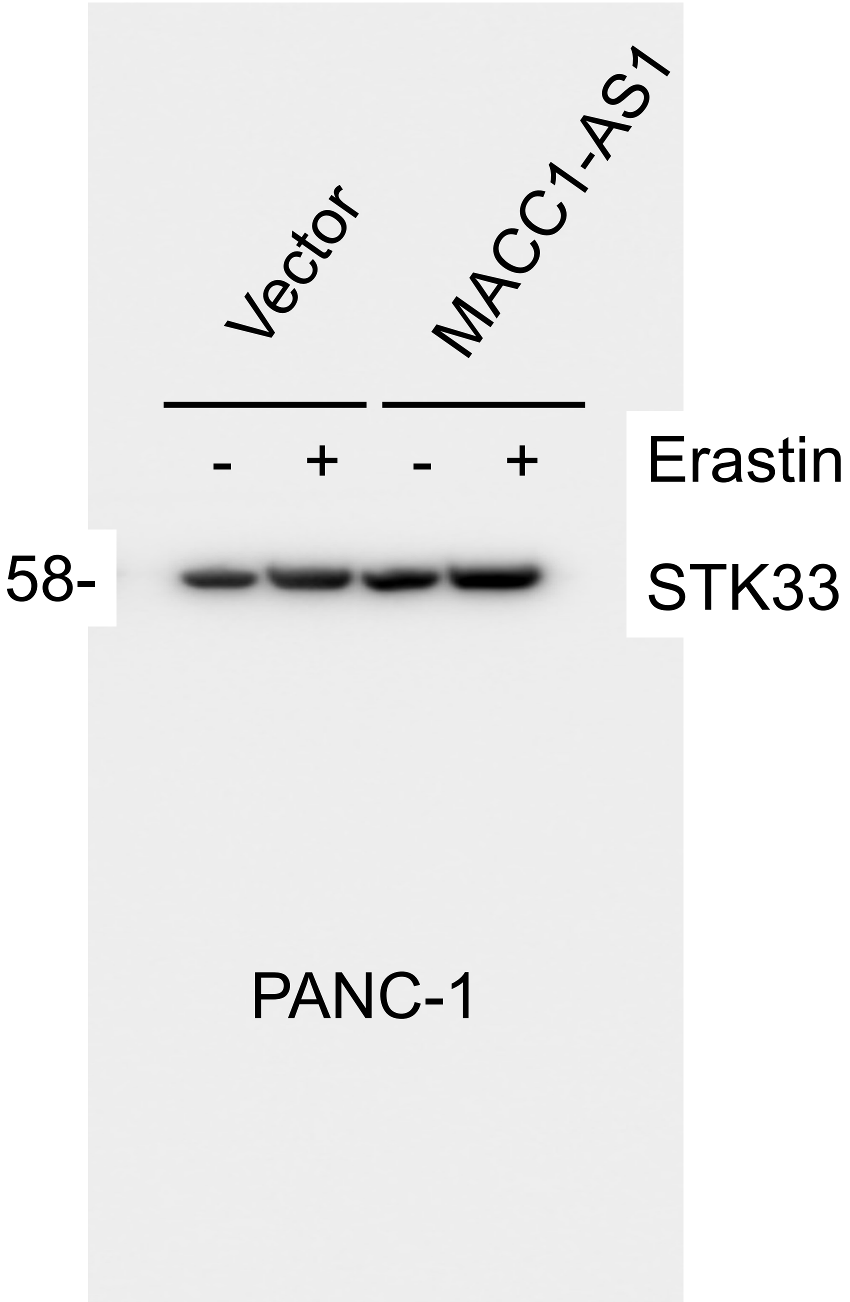
Vector
MACC1-AS1
- + - +
Erastin
58-
STK33
PANC-1

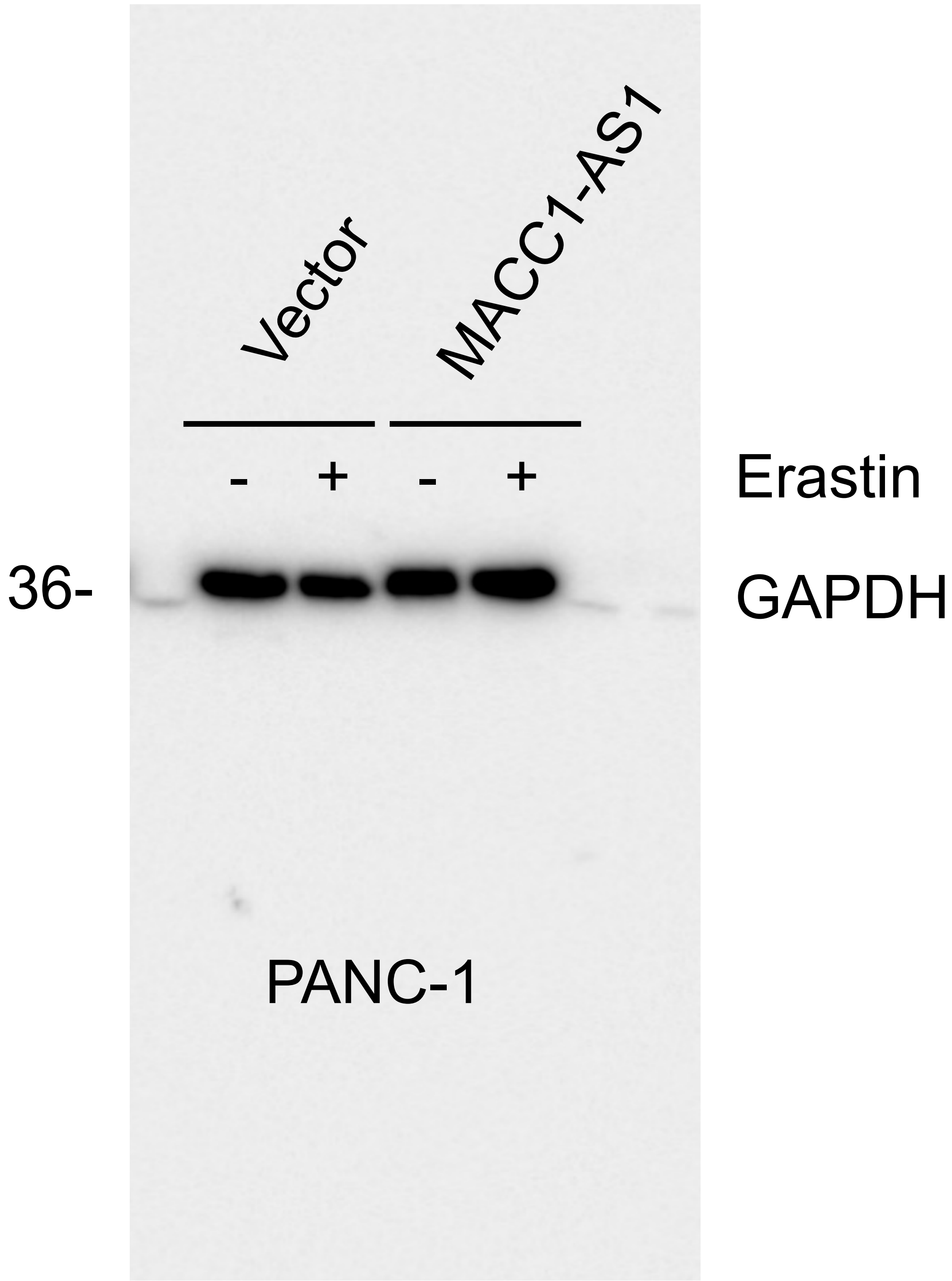
Vector
MACC1-AS1
- + - +
Erastin
36-
GAPDH
PANC-1

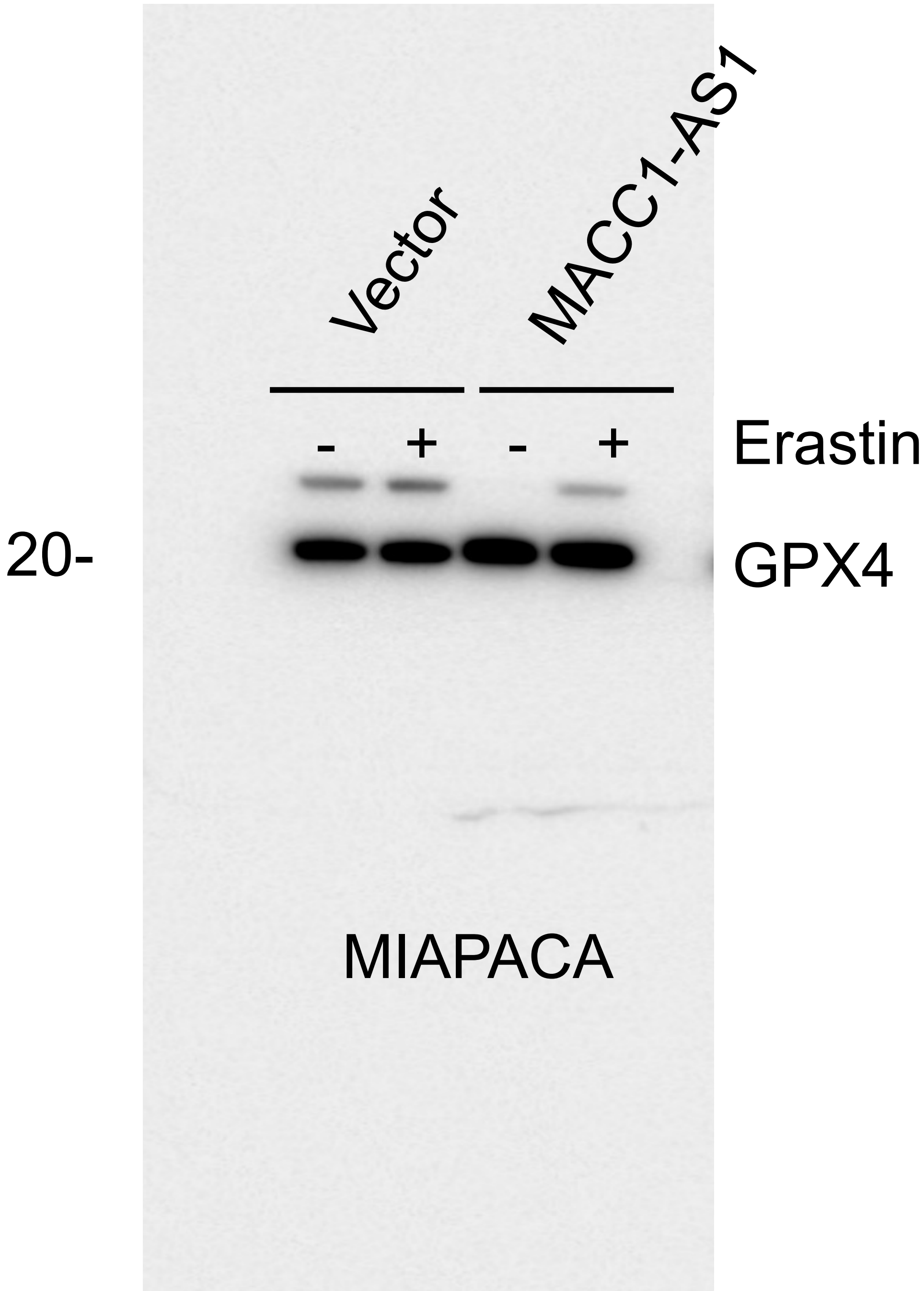
Vector
MACC1-AS1
- + - +
Erastin
20-
GPX4
MIAPACA

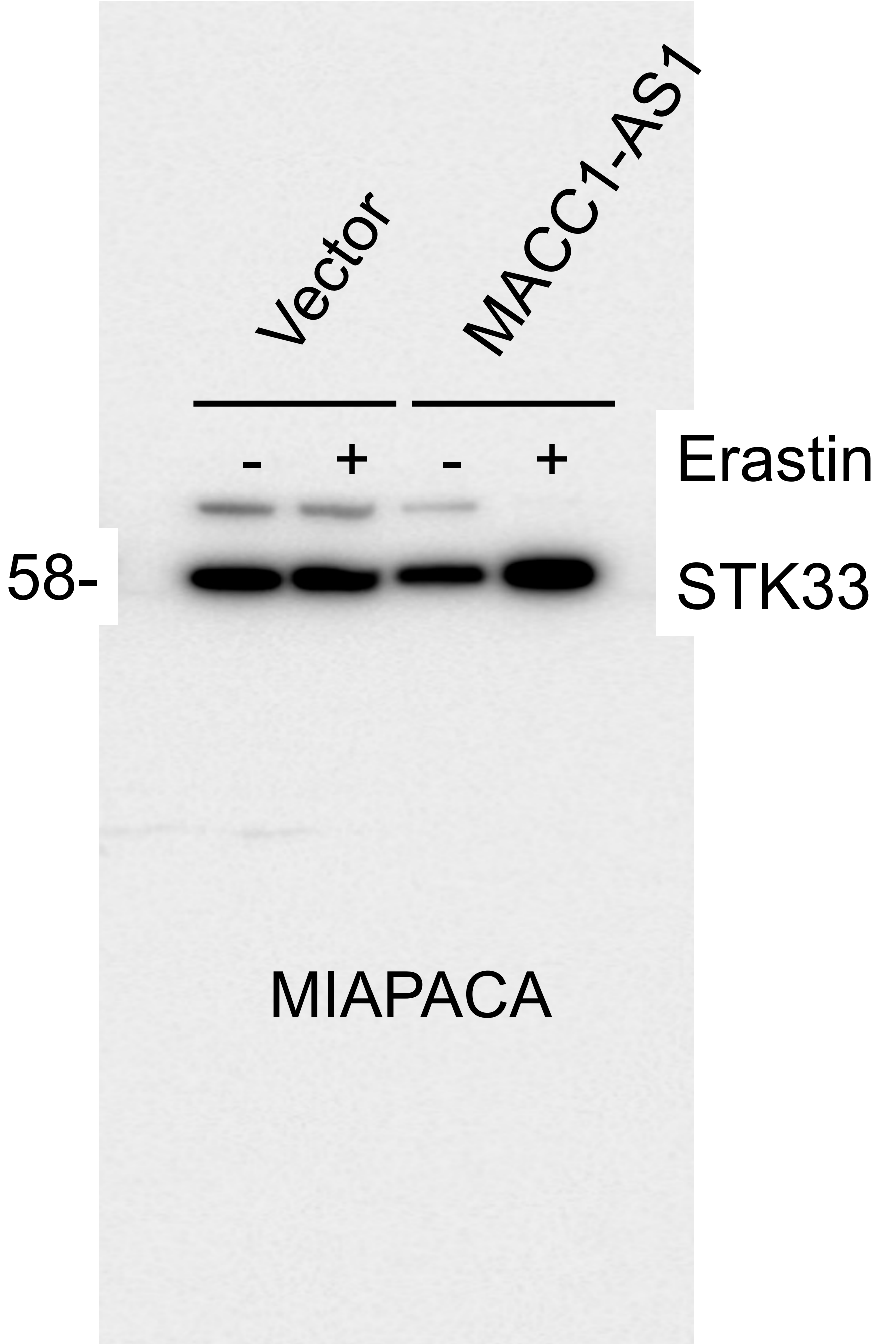
Vector
MACC1-AS1
- + - + Erastin
58-
STK33
MIAPACA

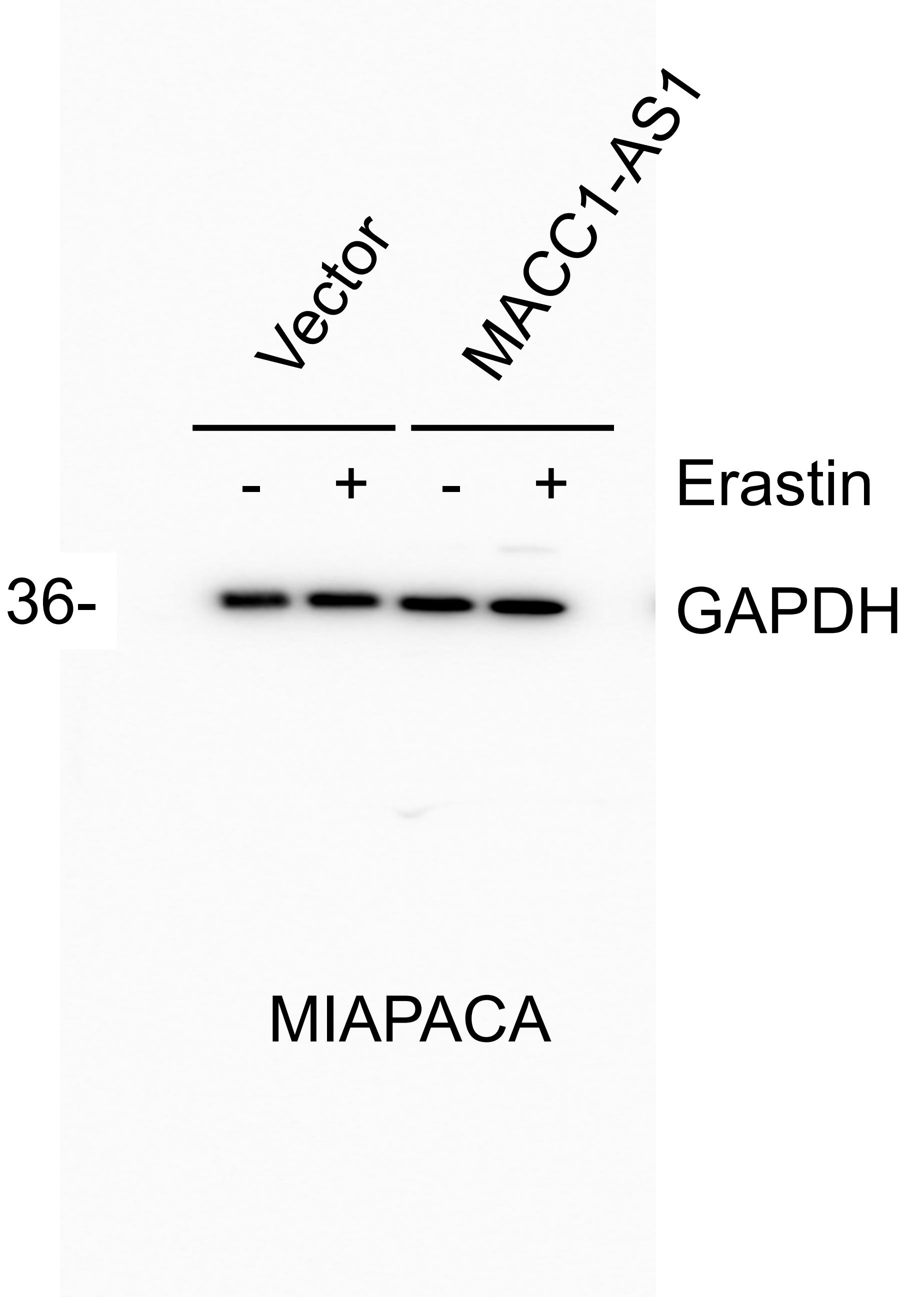

Vector
MACC1-AS1
- + - + Erastin
36-
GAPDH
MIAPACA

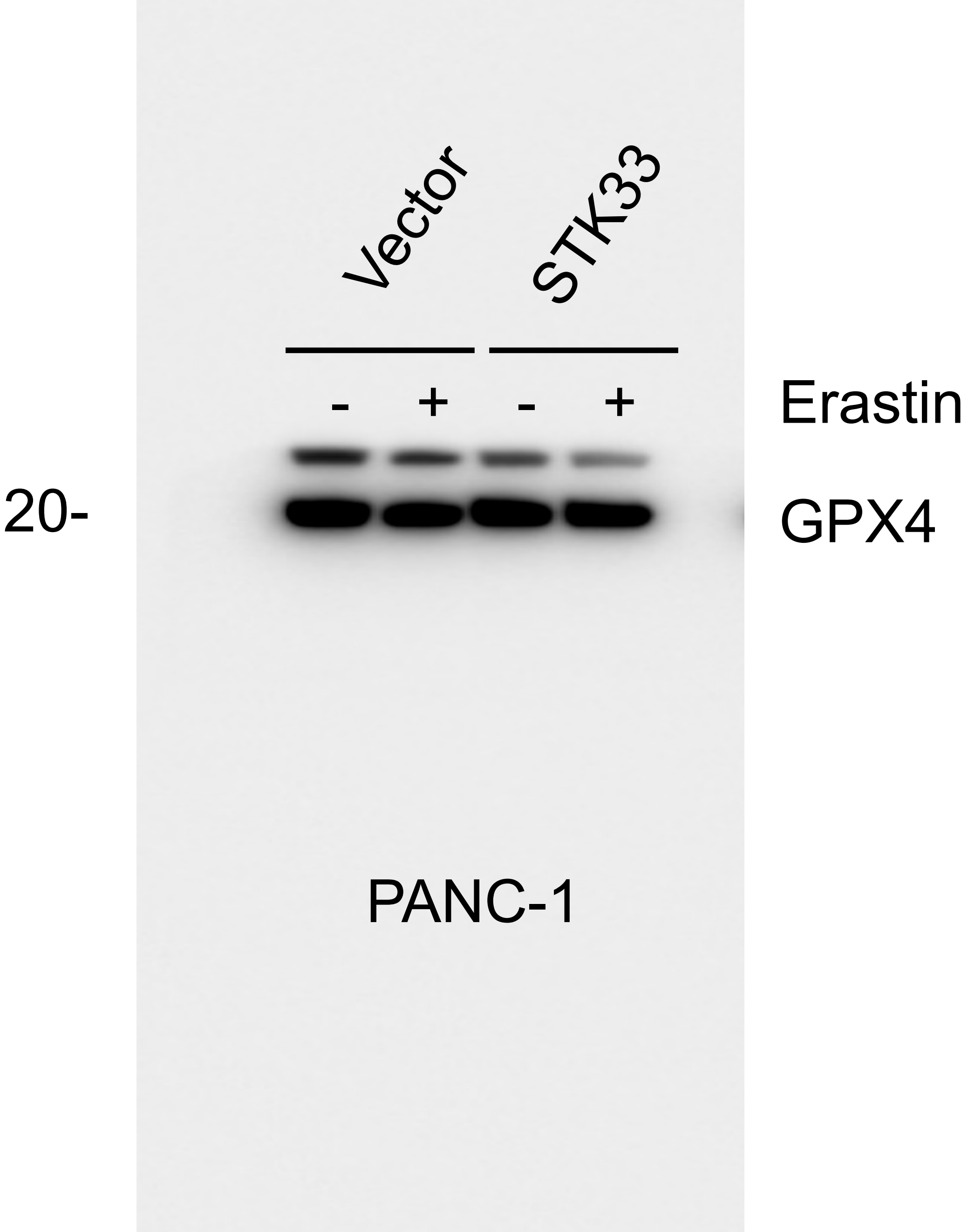
Vector
STK33
- + - +
Erastin
20-
GPX4
PANC-1

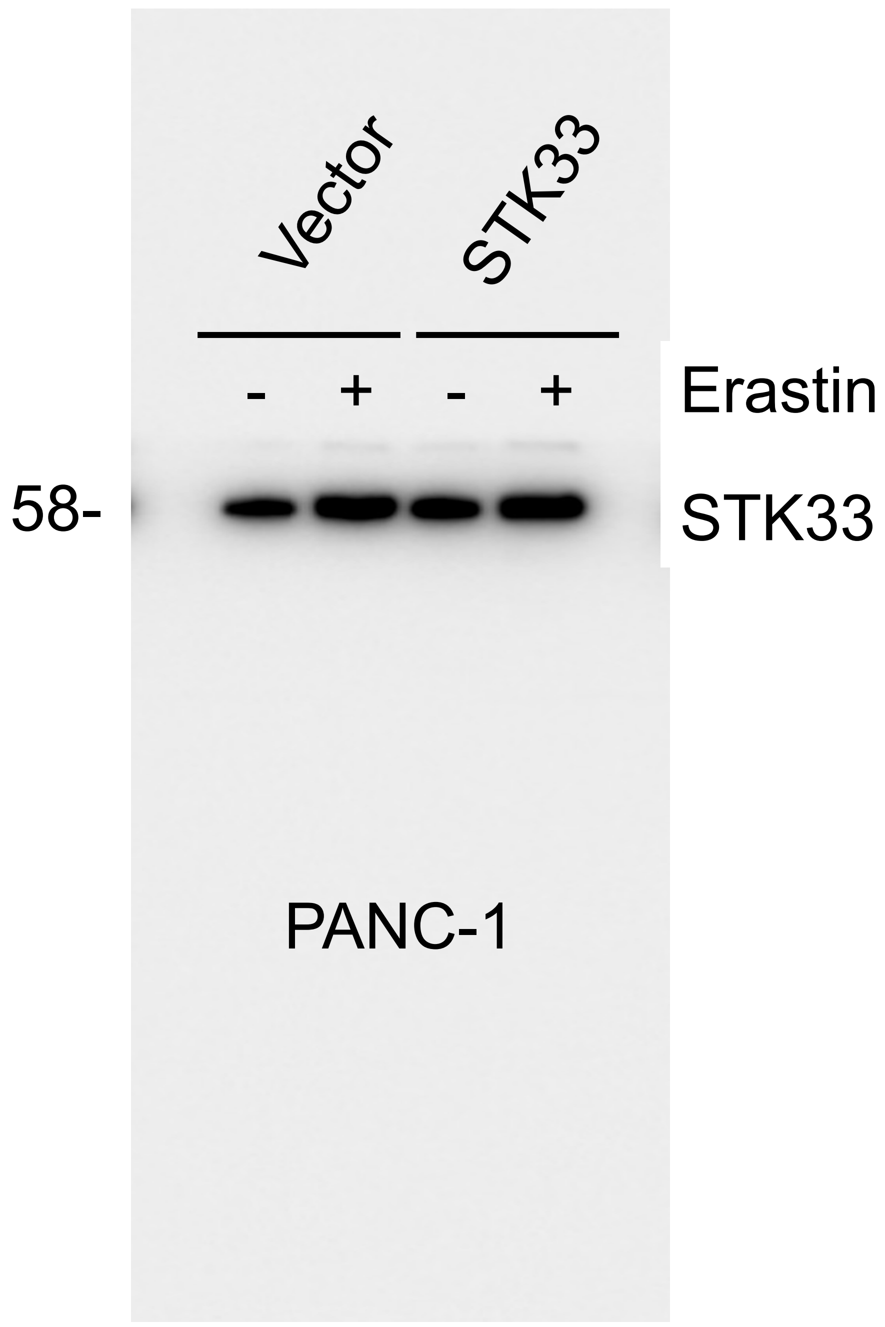
Vector
STK33
- + - +
Erastin
58-
STK33
PANC-1

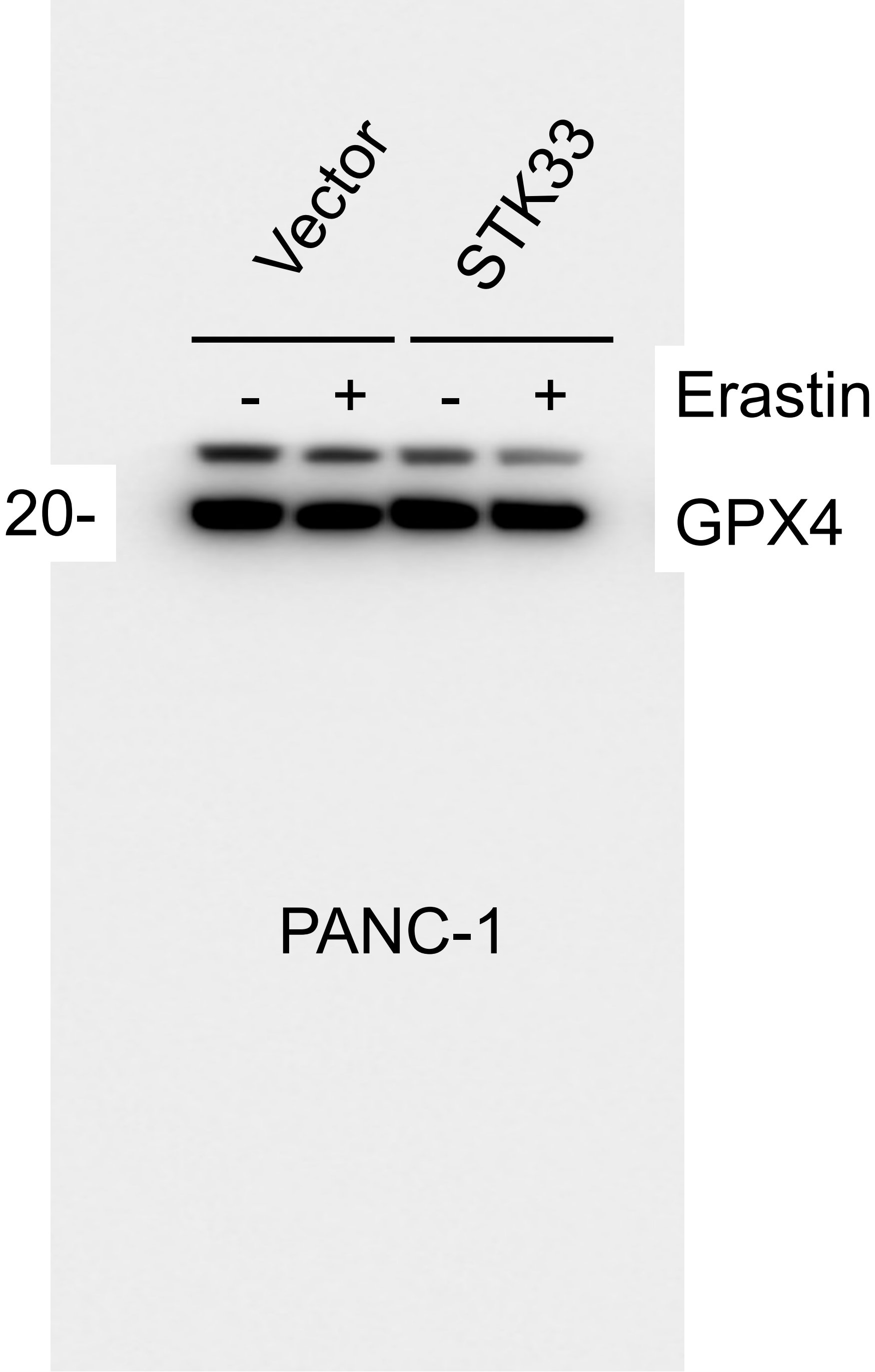

Vector
STK33
- + - +
Erastin
20-
GPX4
PANC-1

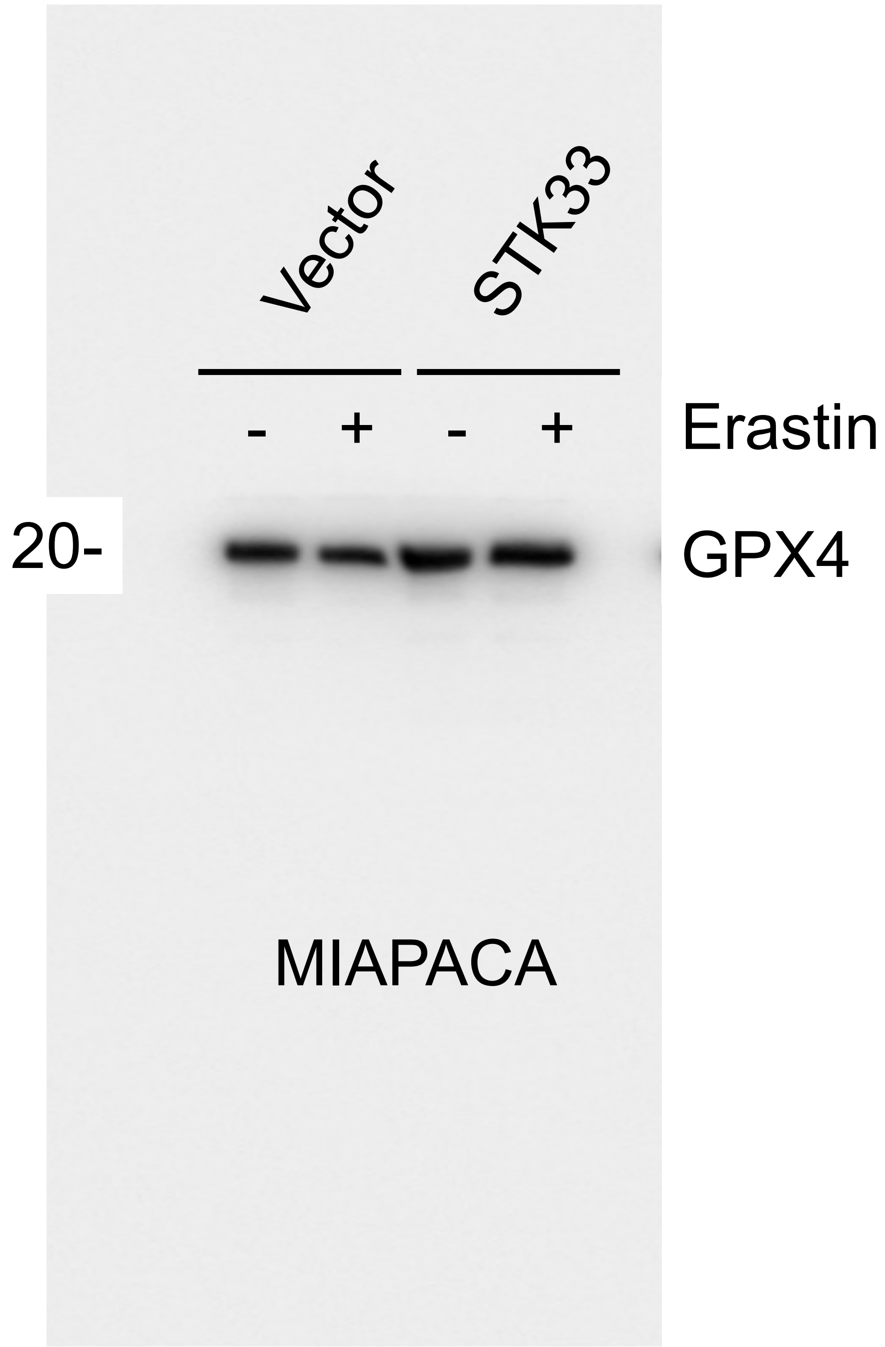
Vector
STK33
- + - +
Erastin
20-
GPX4
MIAPACA

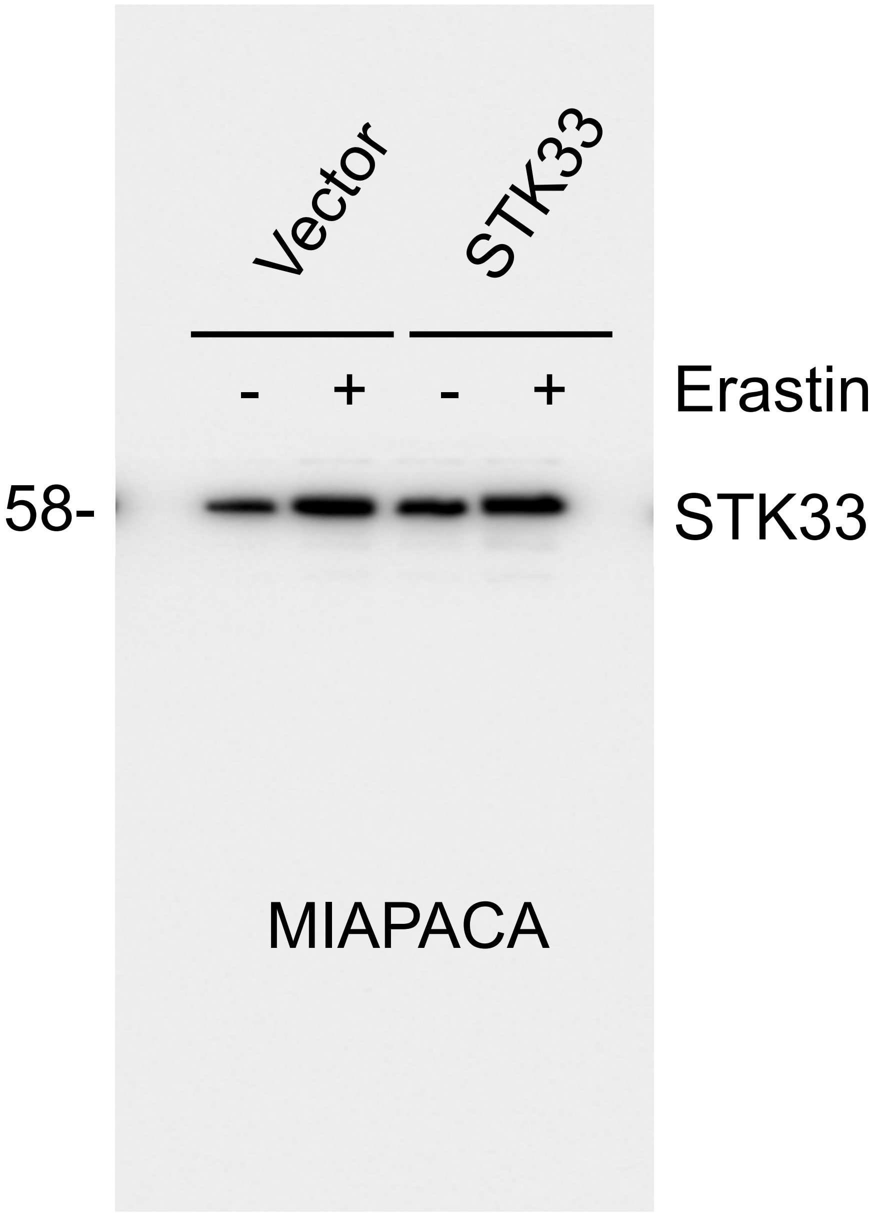
Vector
STK33
- + - +
Erastin
58-
STK33
MIAPACA

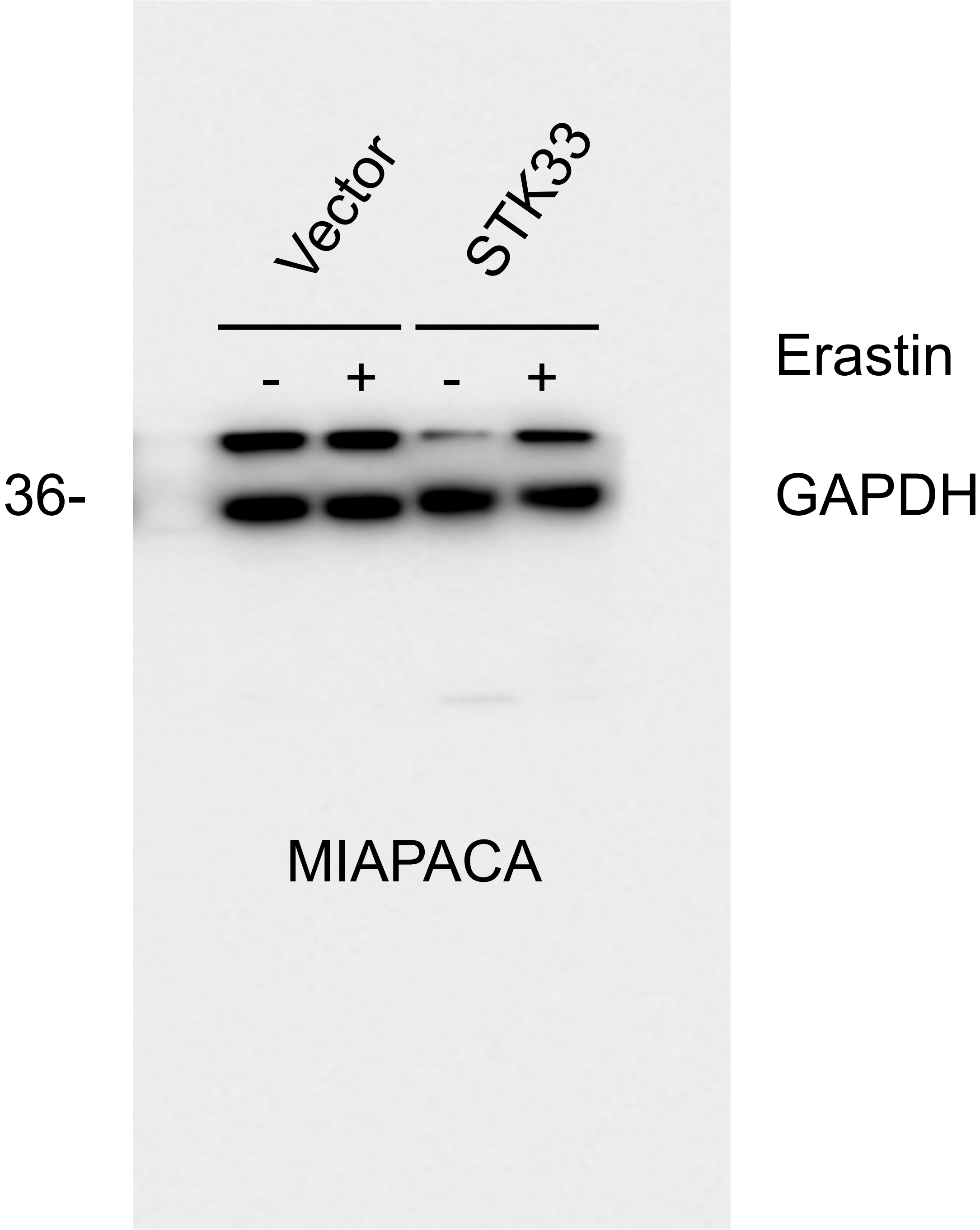

Vector
STK33
Erastin
- + - +
36-
GAPDH
MIAPACA

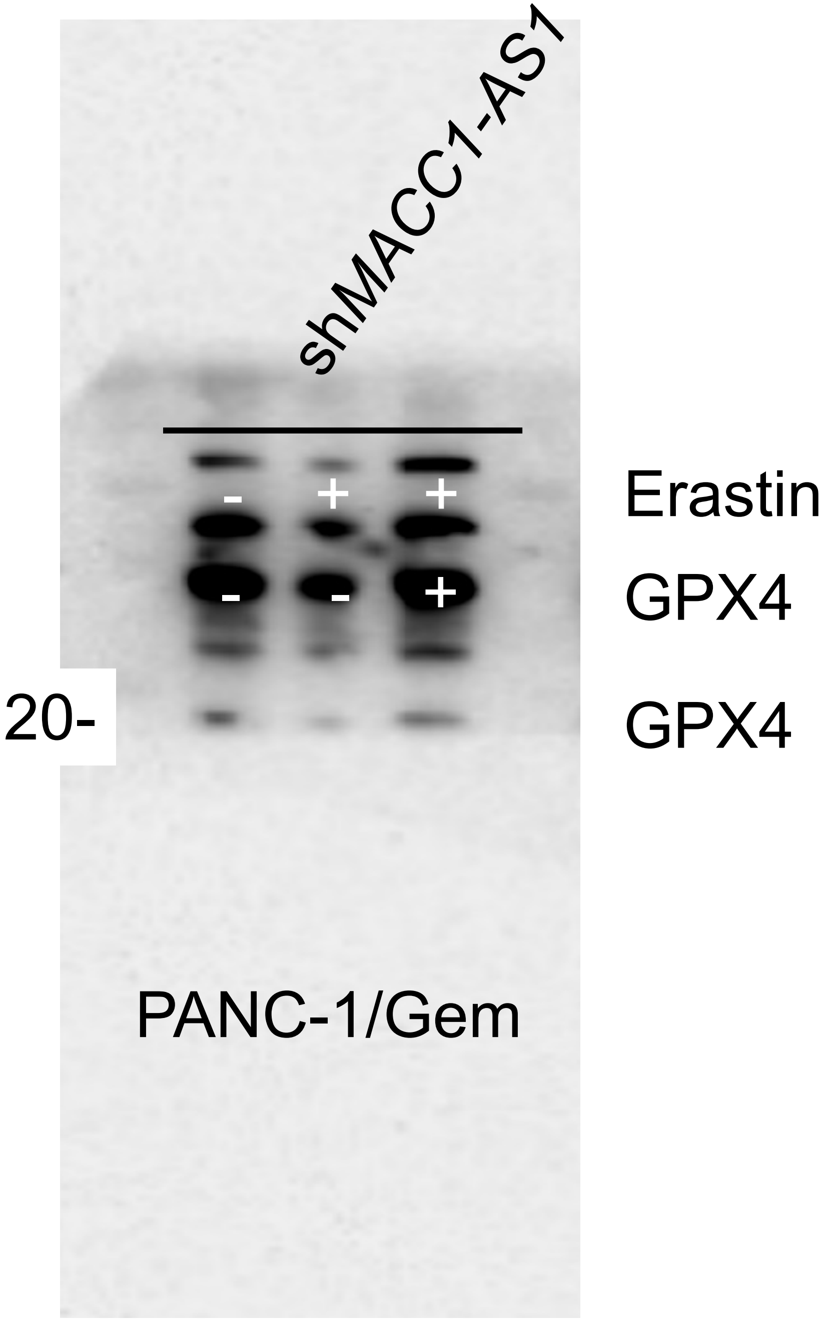
shMACC1-AS1
- - +
Erastin
- + +
GPX4
GPX4
20-
PANC-1/Gem

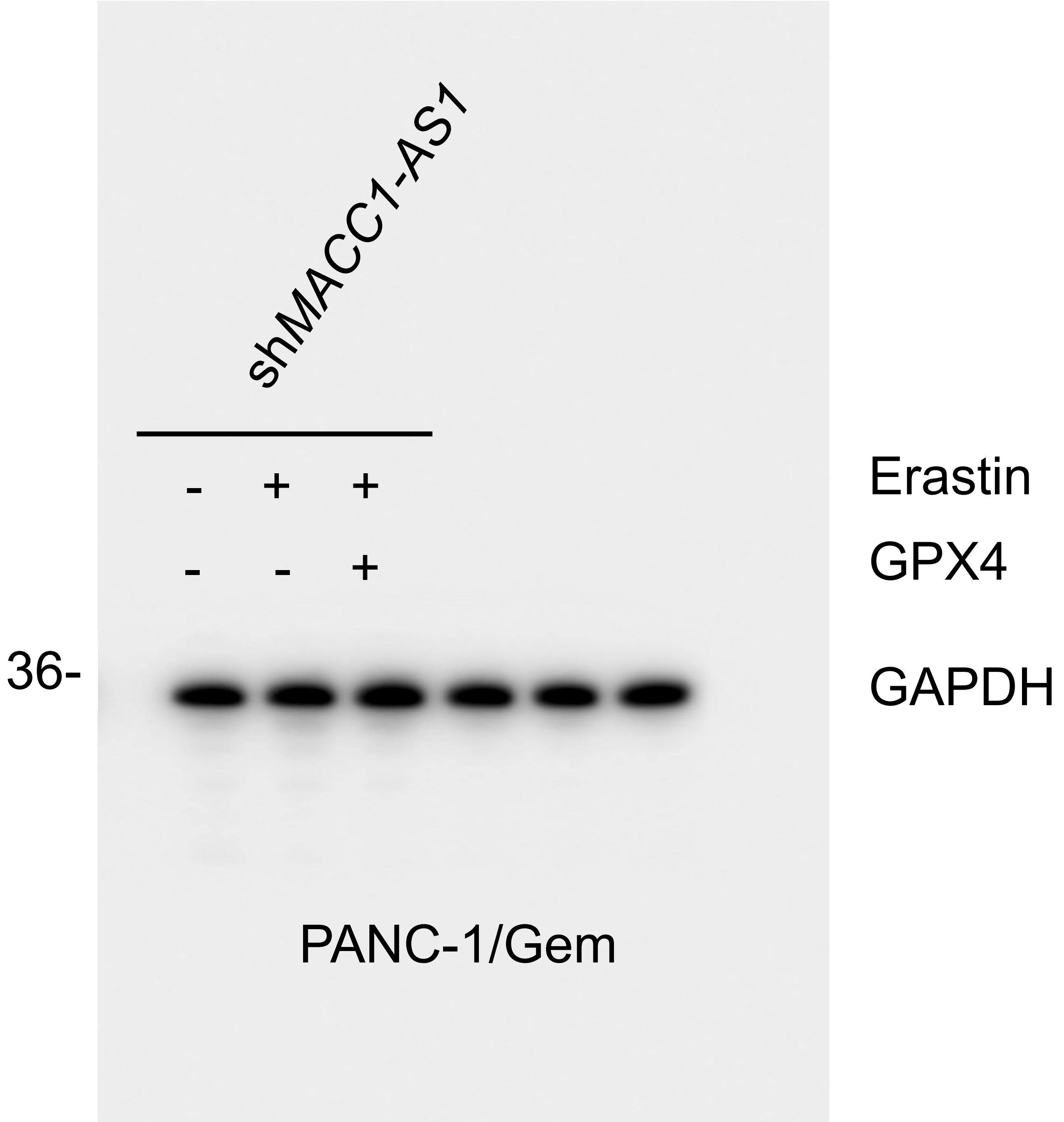
shMACC1-AS1
-
+
+
Erastin
-
-
+
GPX4
36-
GAPDH
PANC-1/Gem

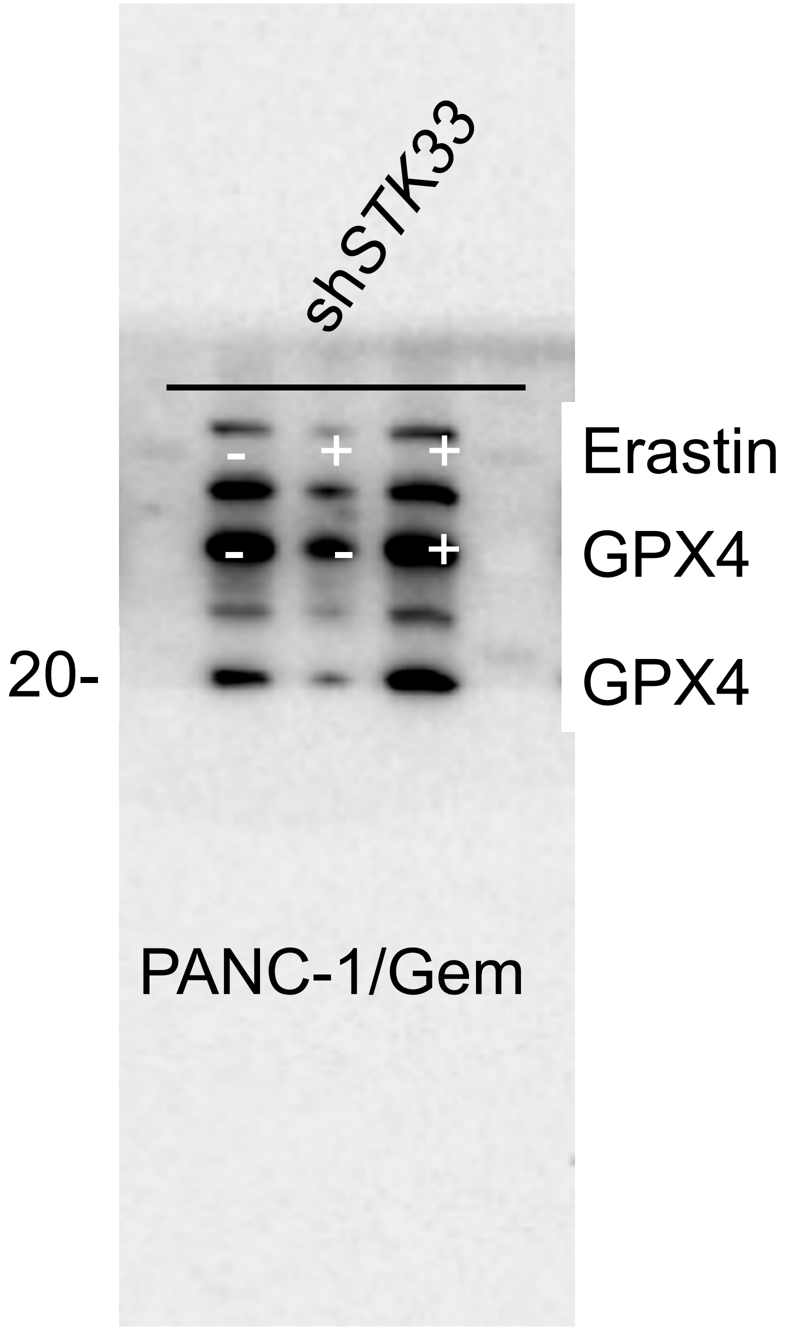

shSTK33
Erastin
- + +
GPX4
- - +
GPX4
20-
PANC-1/Gem

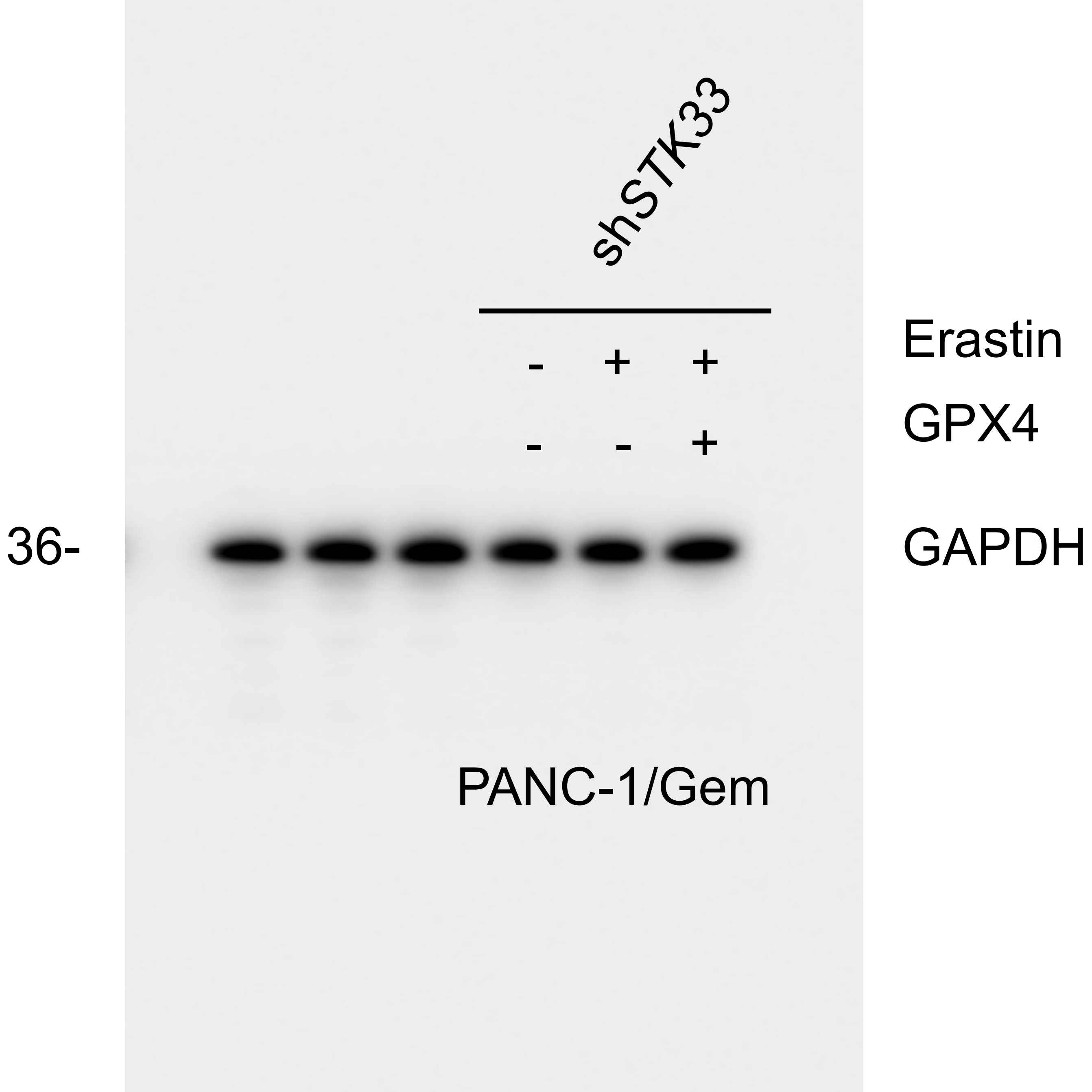
shSTK33
Erastin
- + +
GPX4
- - +
36-
GAPDH
PANC-1/Gem

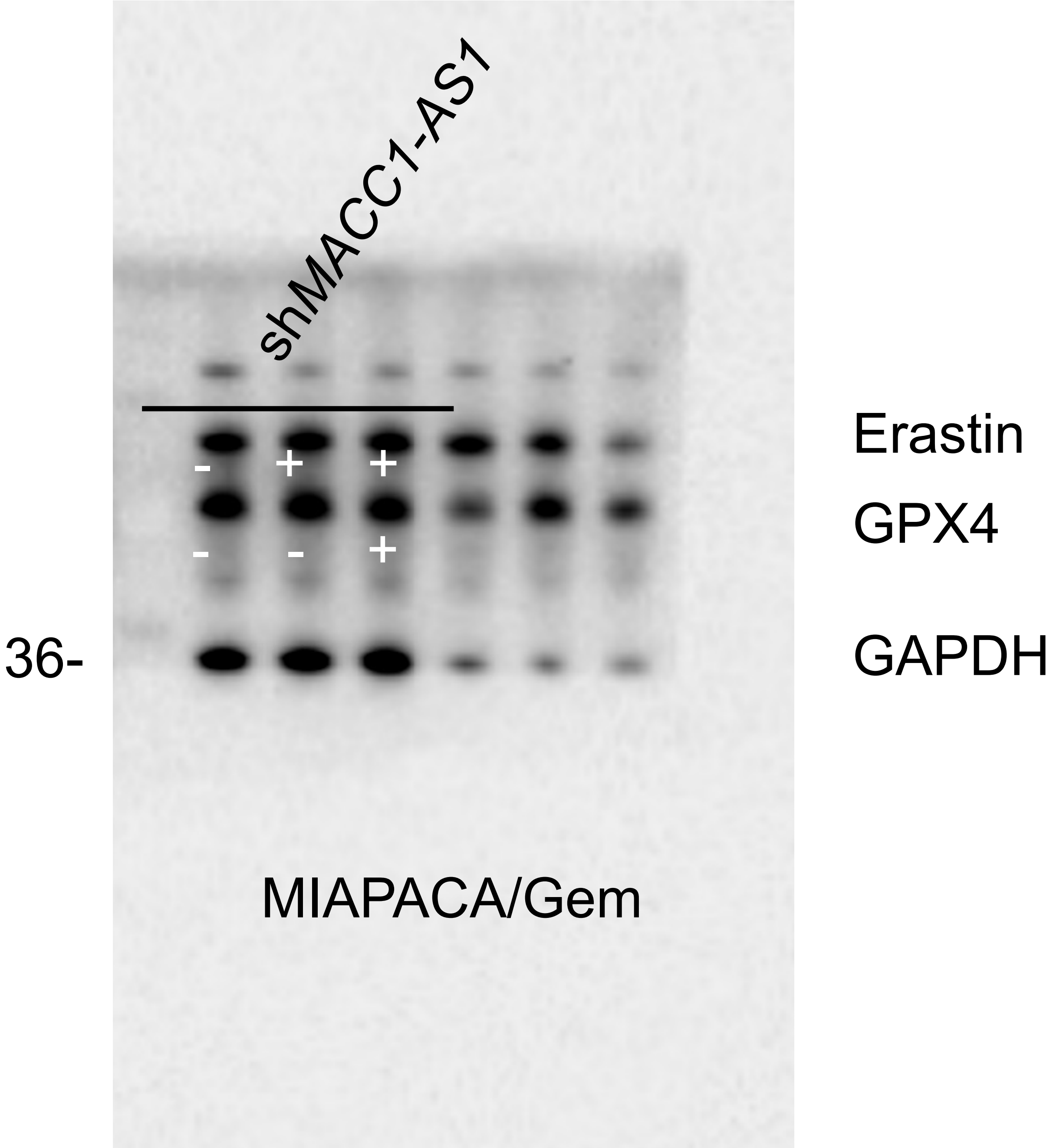
shMACC1-AS1
Erastin
- + +
GPX4
- - +
36-
GAPDH
MIAPACA/Gem

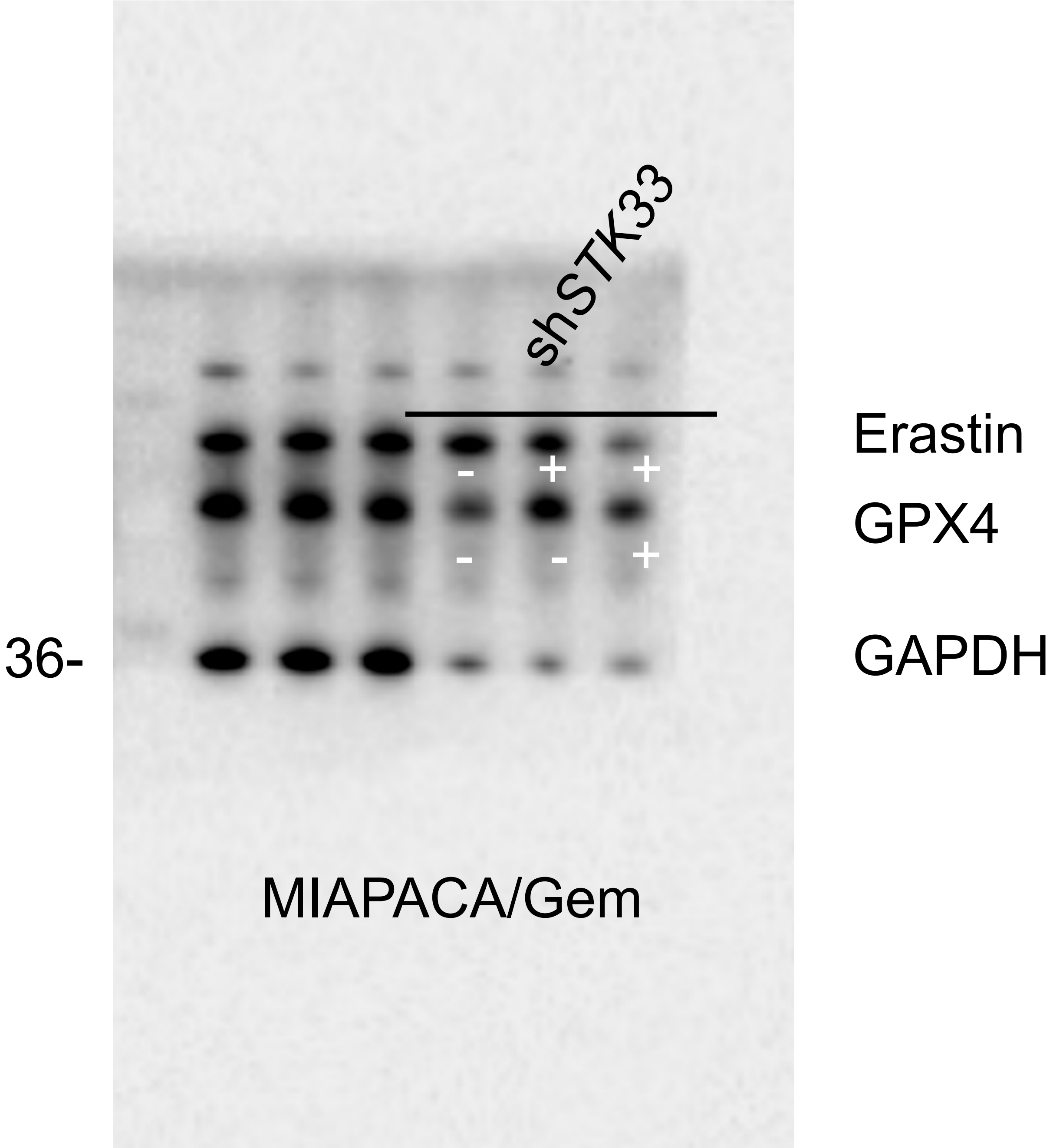

shSTK33
Erastin
- + +
GPX4
- - +
36-
GAPDH
MIAPACA/Gem

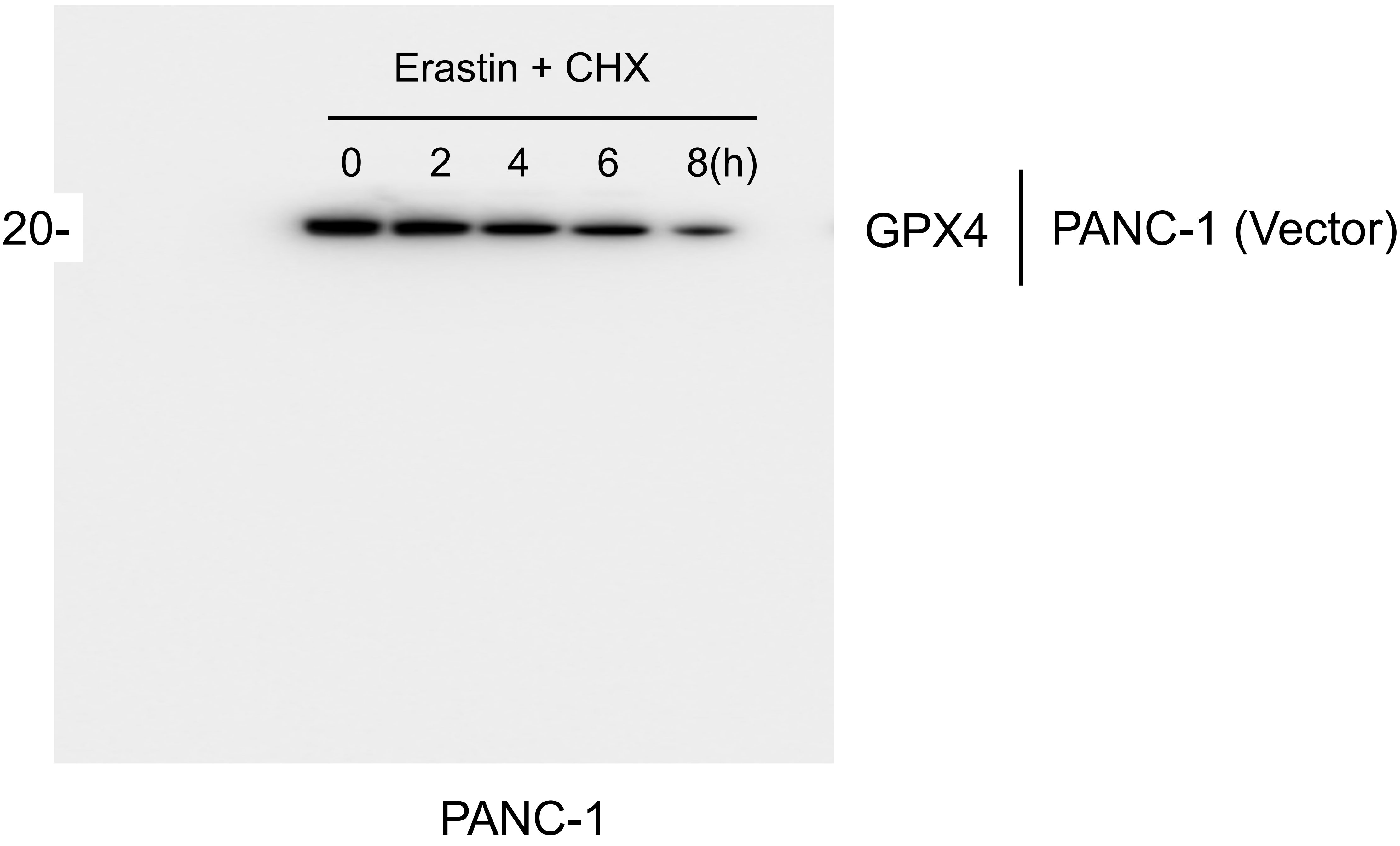
Erastin + CHX
0
2
4
6
8(h)
20-
GPX4
PANC-1 (Vector)
PANC-1

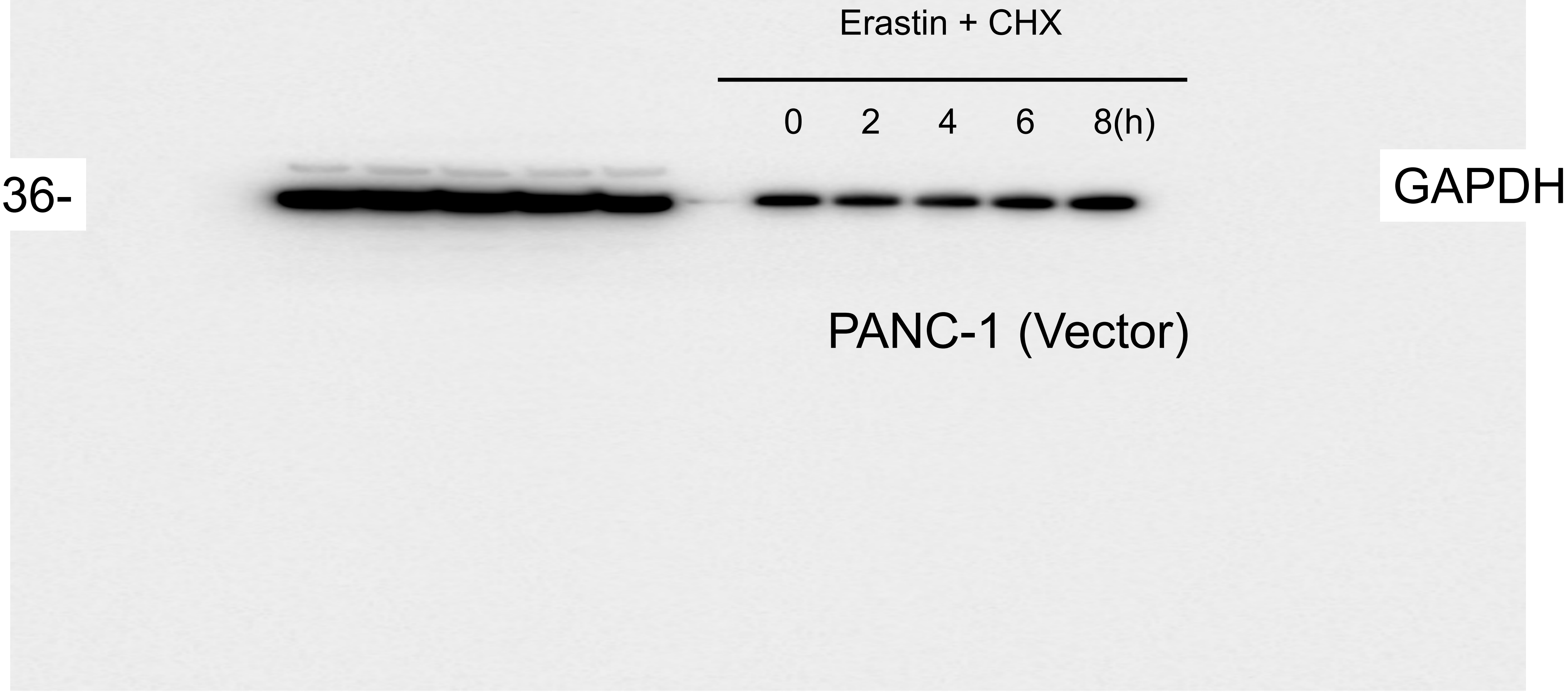
Erastin + CHX
0
2
4
6
8(h)
36-
GAPDH
PANC-1 (Vector)

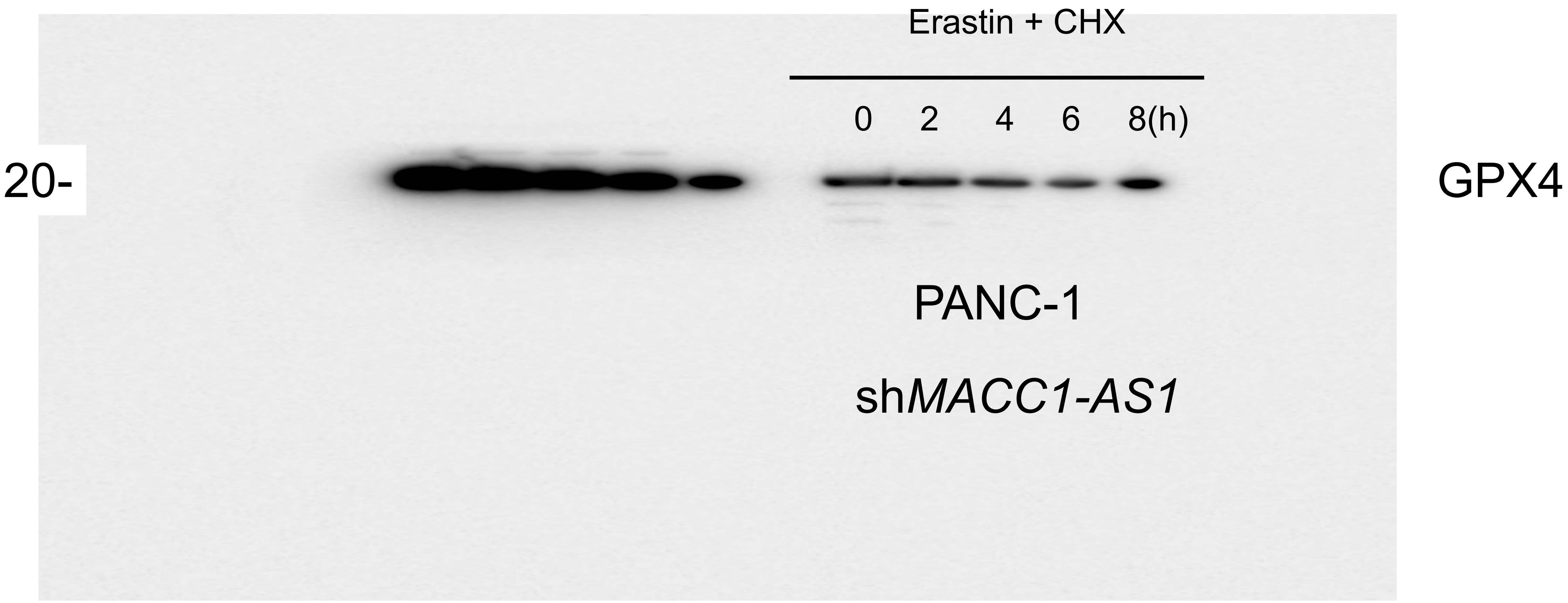
Erastin + CHX
0
2
4
6
8(h)
20-
GPX4
PANC-1
shMACC1-AS1

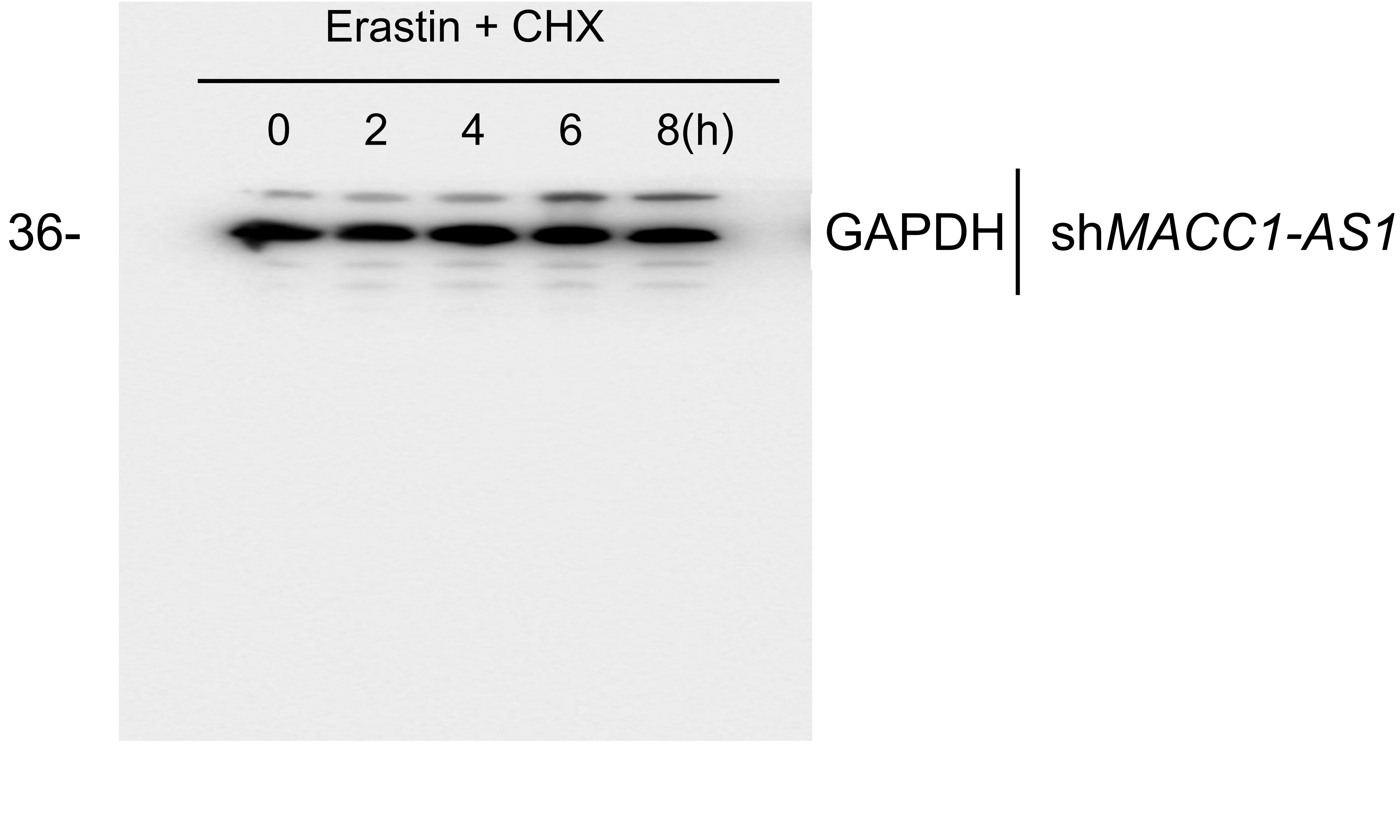

Erastin + CHX
0
2
4
6
8(h)
36-
GAPDH
sh*MACC1-AS1*
PANC-1

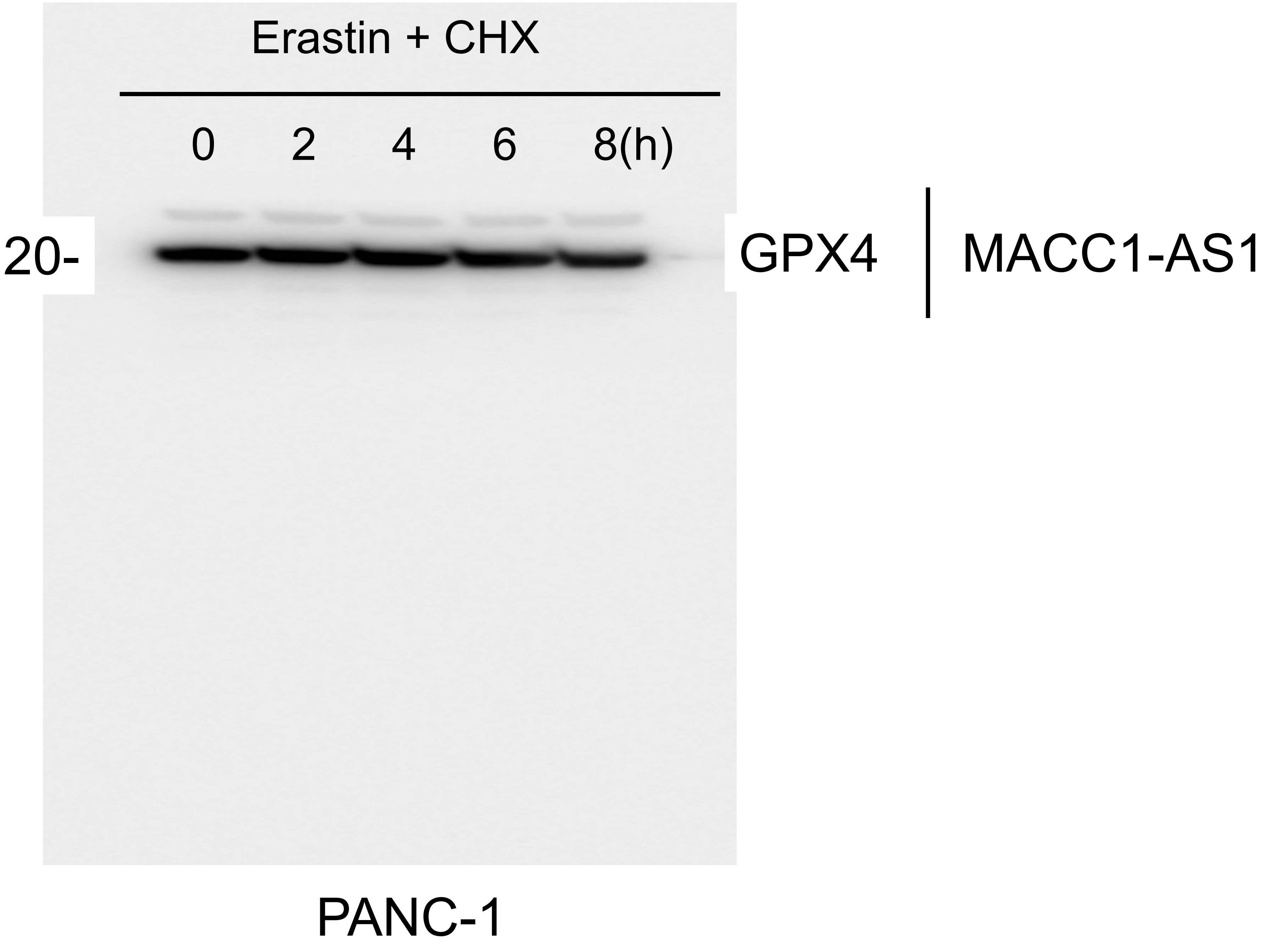
Erastin + CHX
0
2
4
6
8(h)
20-
GPX4
MACC1-AS1
PANC-1

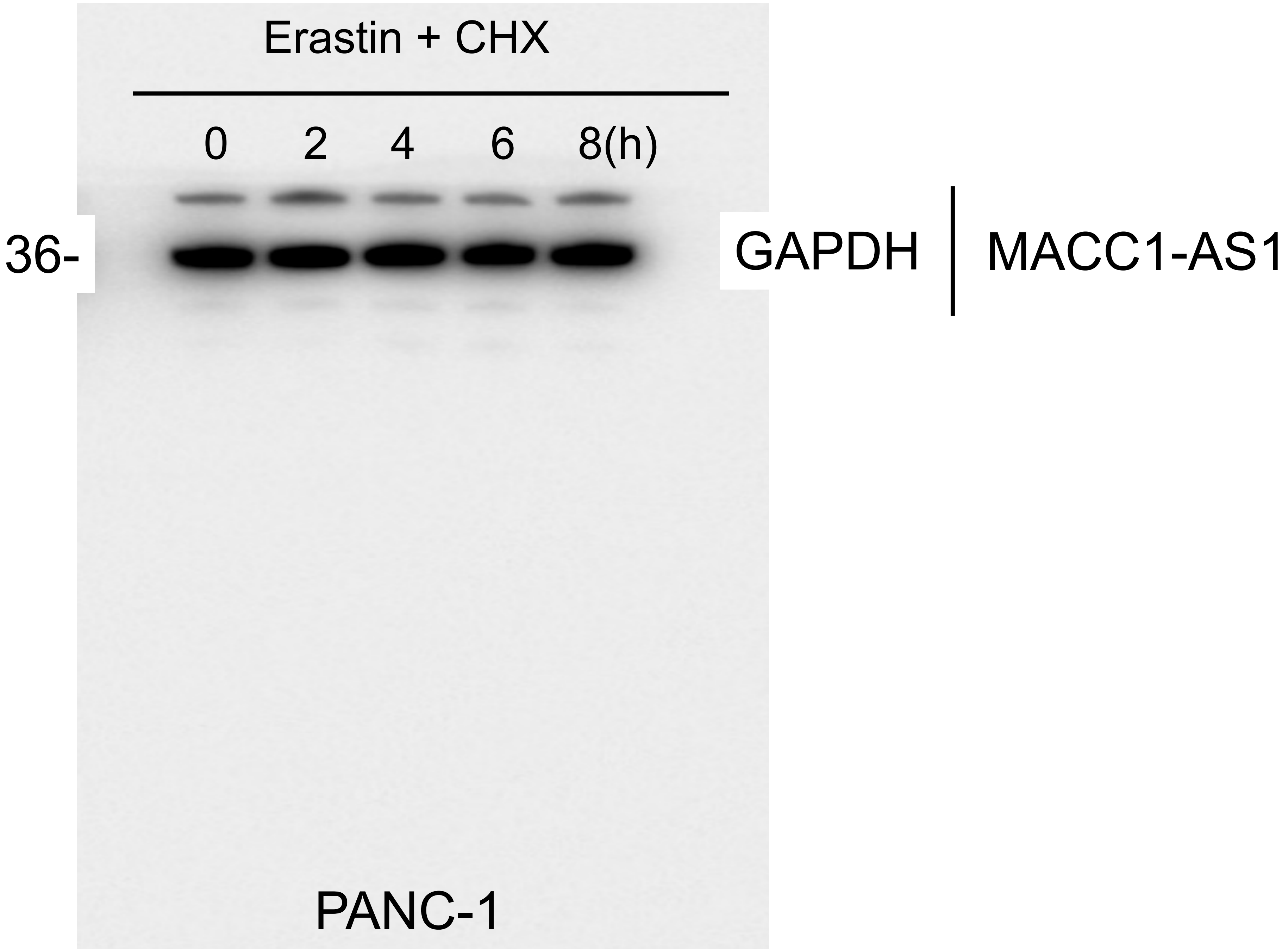
Erastin + CHX
0
2
4
6
8(h)
36-
GAPDH
MACC1-AS1
PANC-1

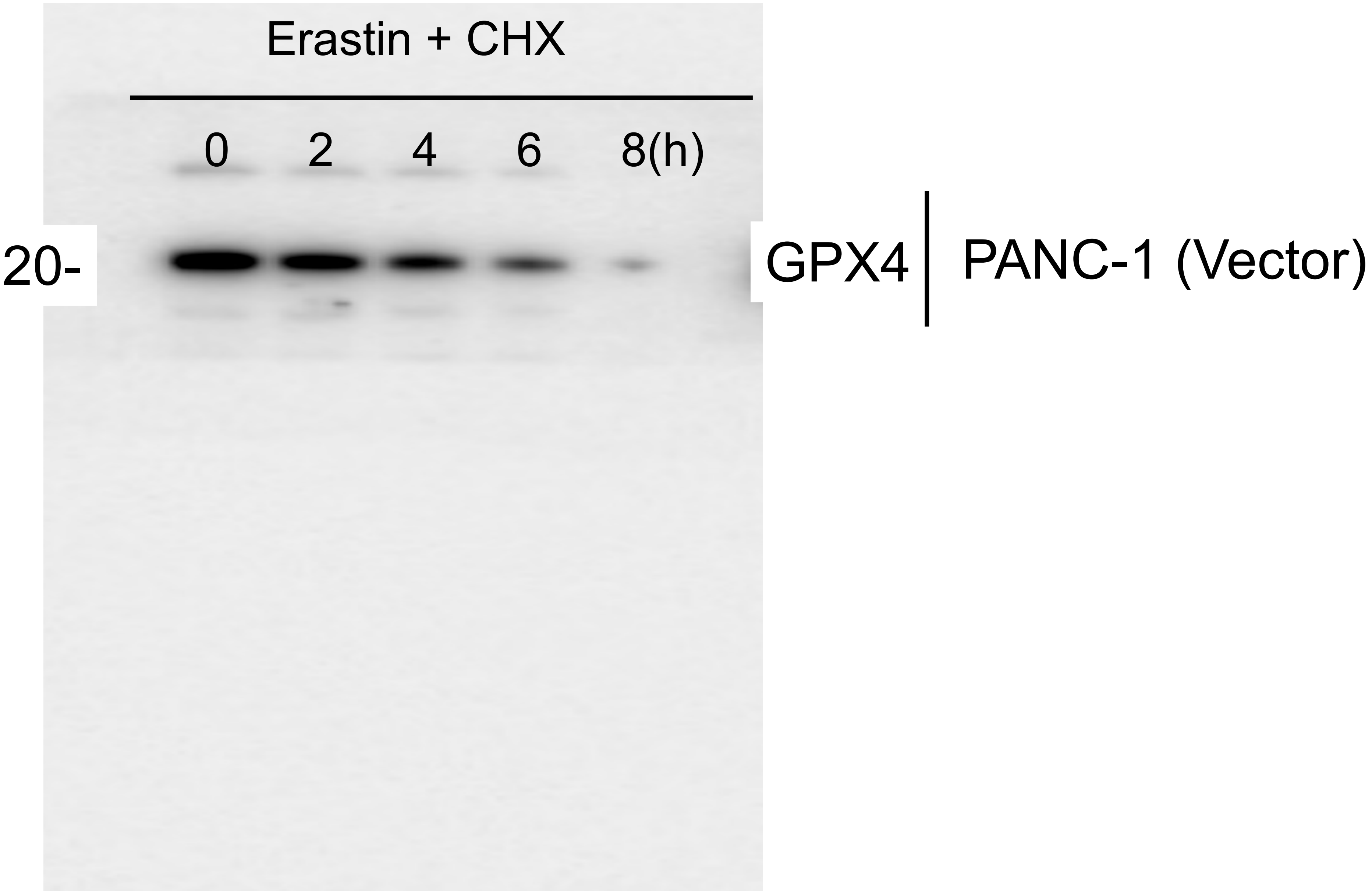


PANC-1

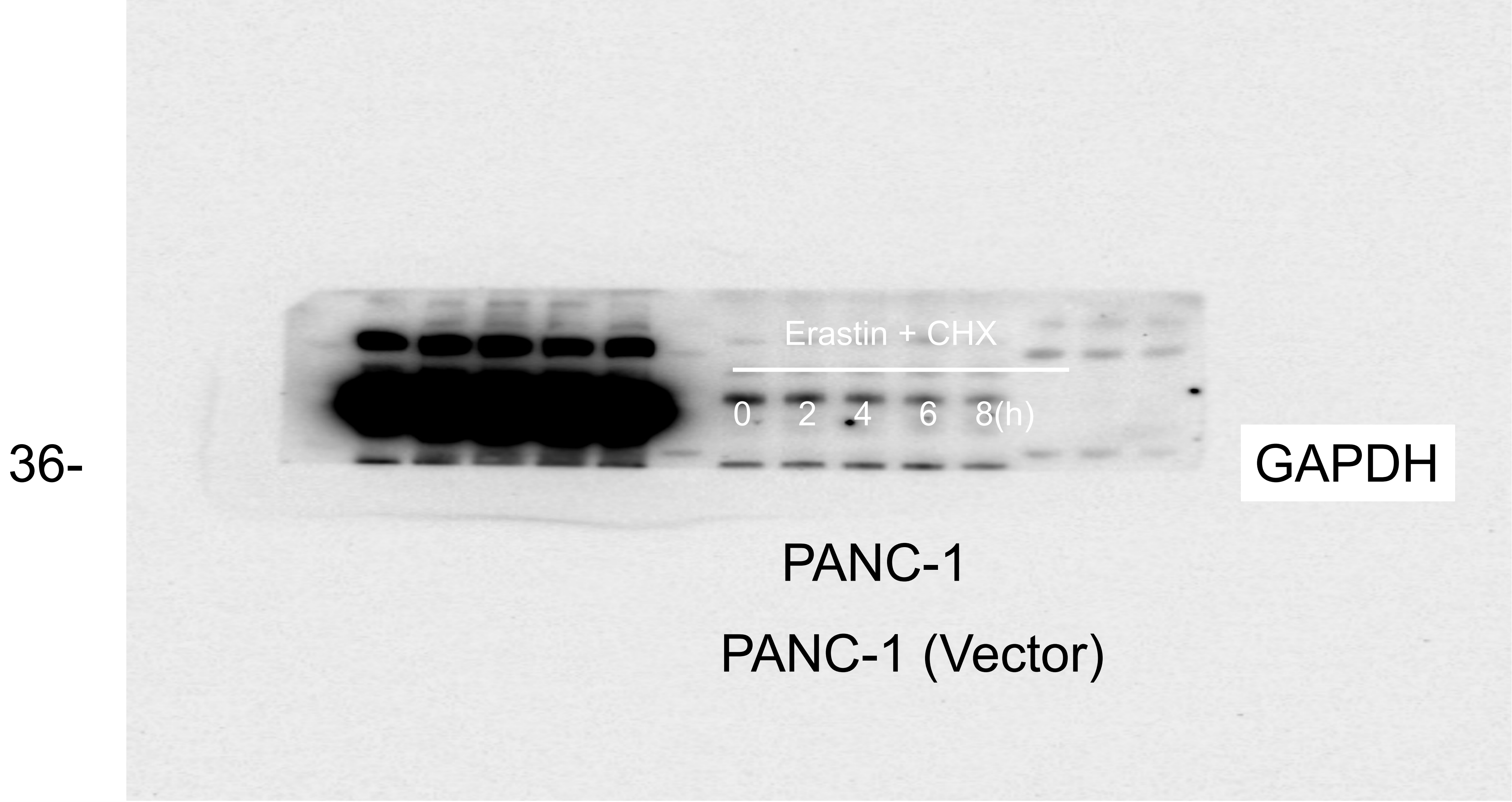
36-
Erastin + CHX
0 2 4 6 8(h)
GAPDH
PANC-1
PANC-1 (Vector)

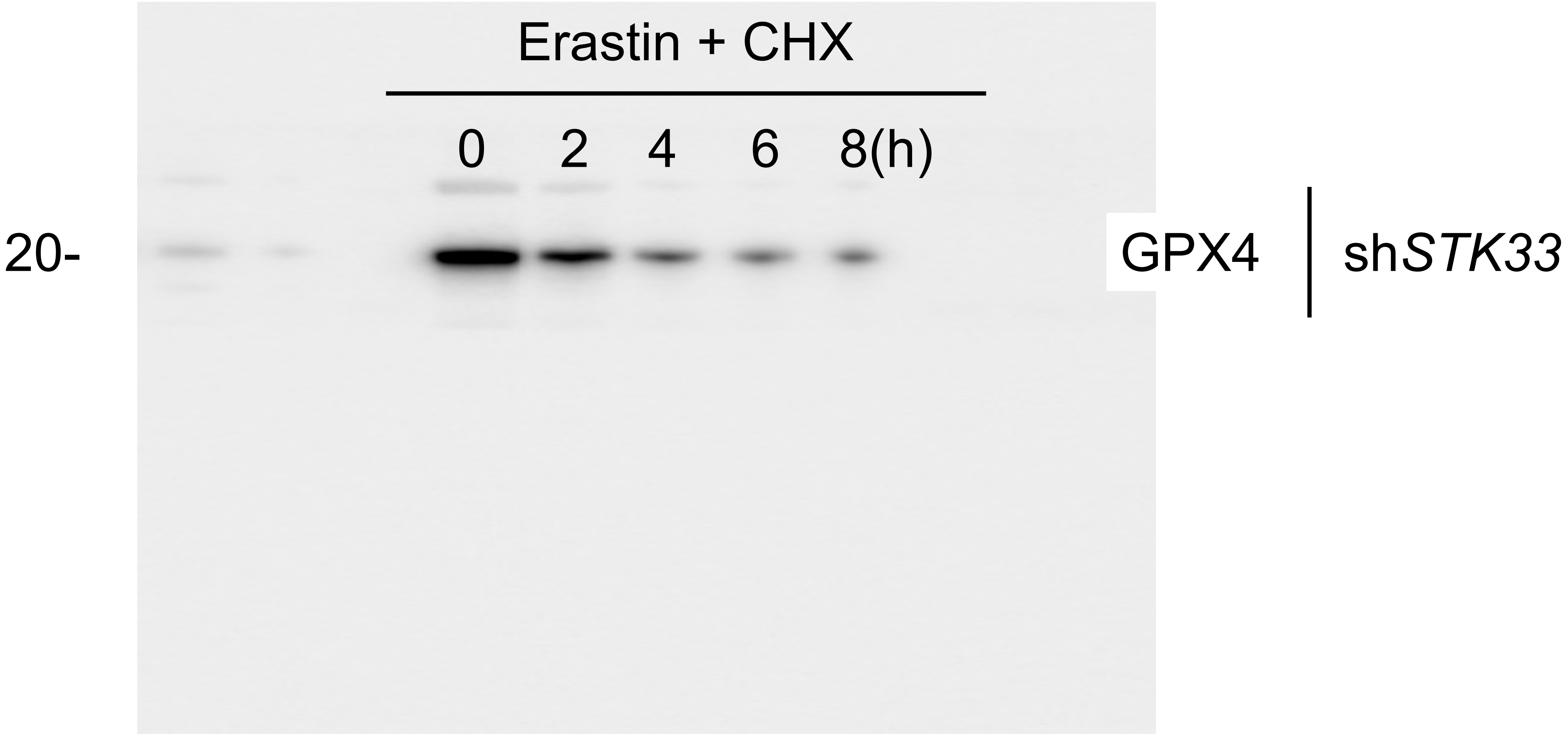


PANC-1

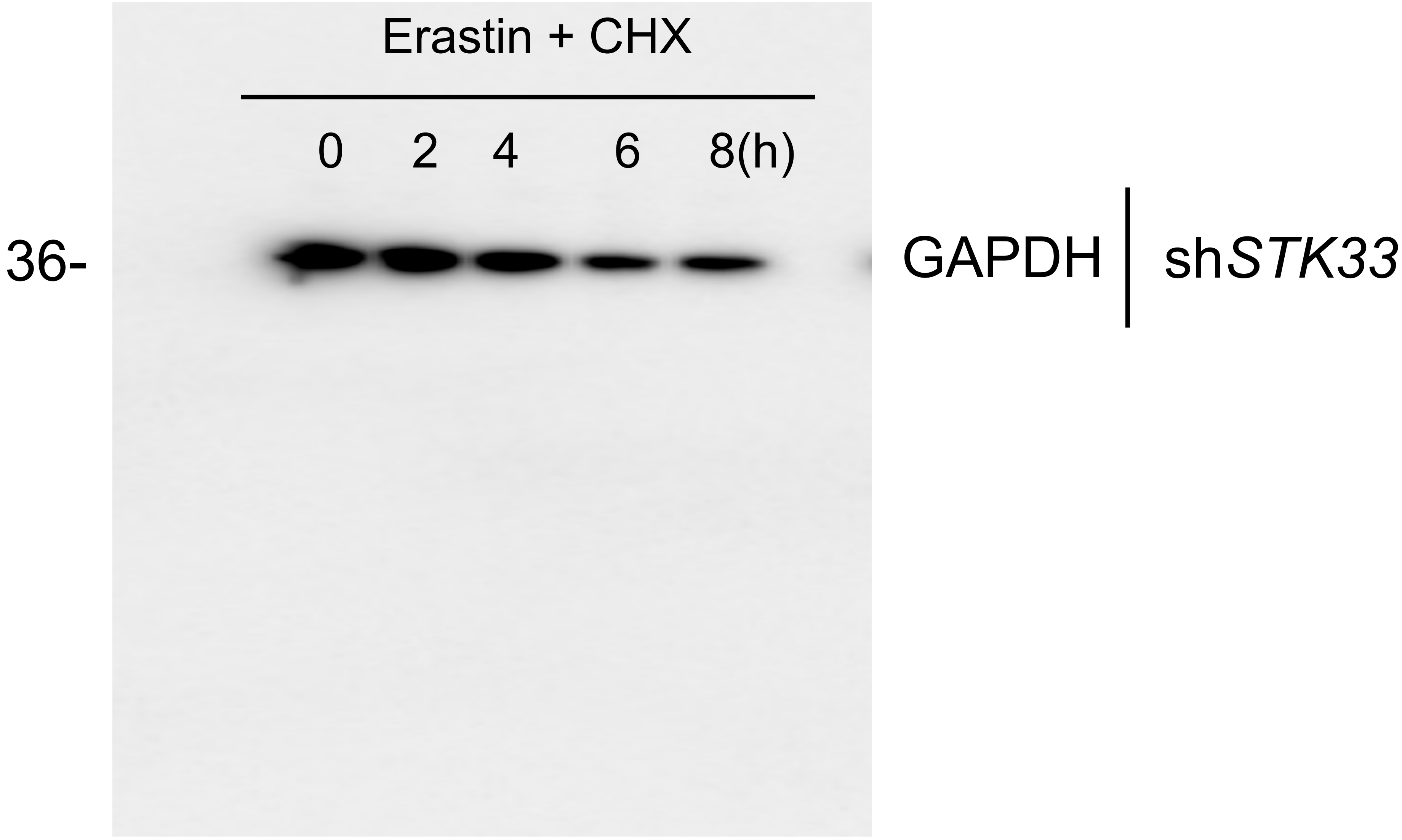


PANC-1

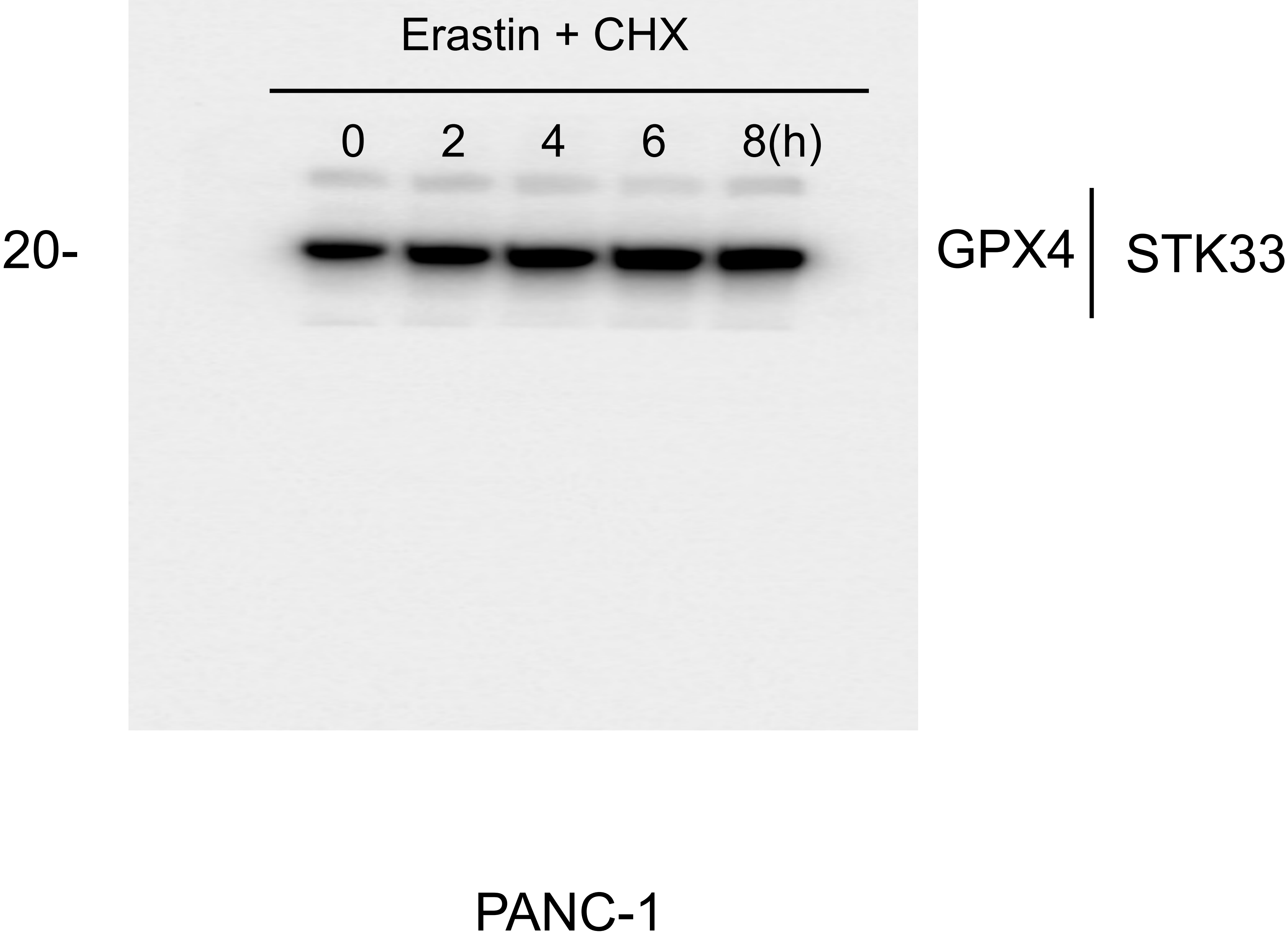
Erastin + CHX
0
2
4
6
8(h)
20-
GPX4
STK33
PANC-1

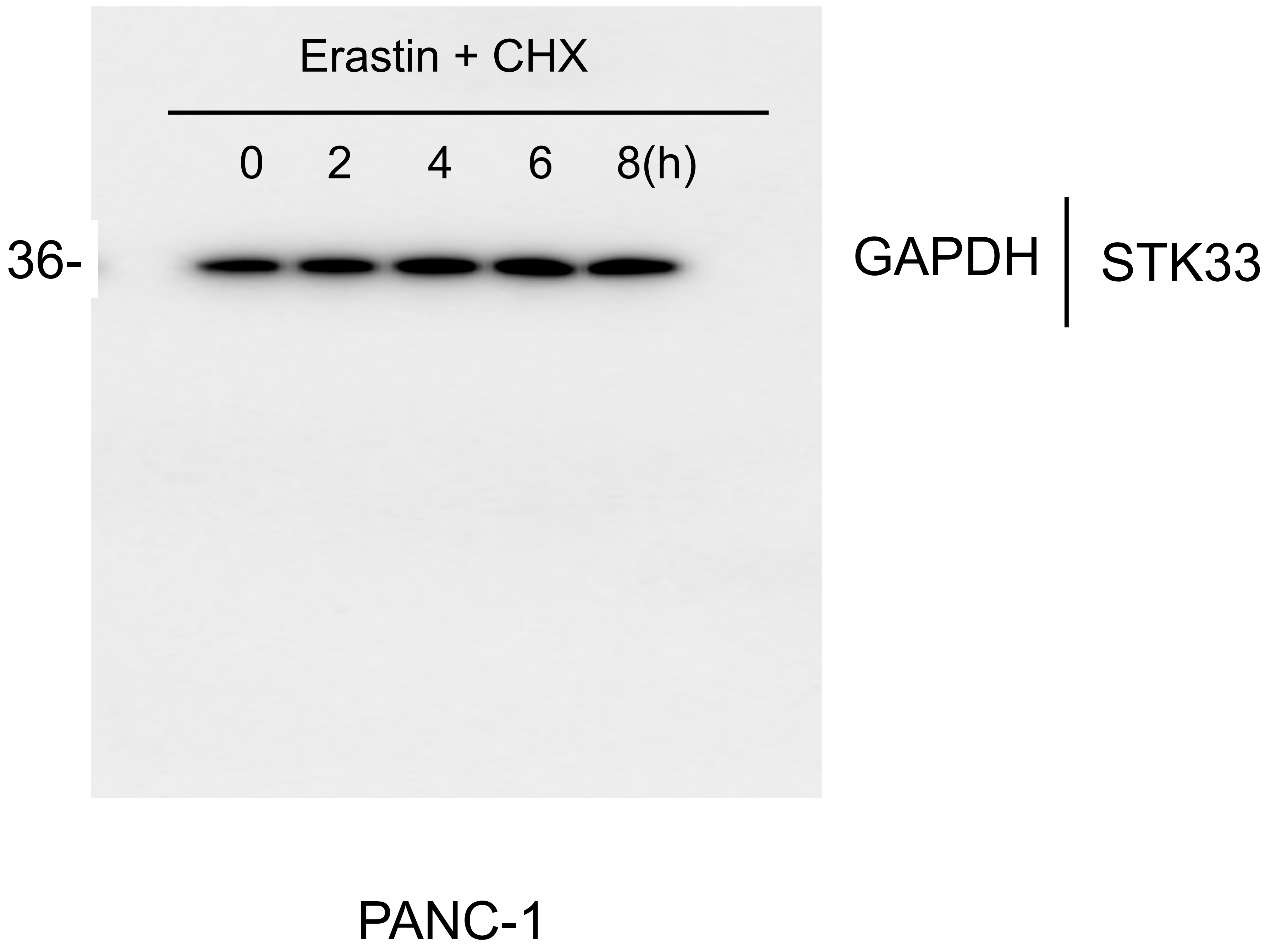
Erastin + CHX
0
2
4
6
8(h)
36-
GAPDH
STK33
PANC-1

Figure 6

22-

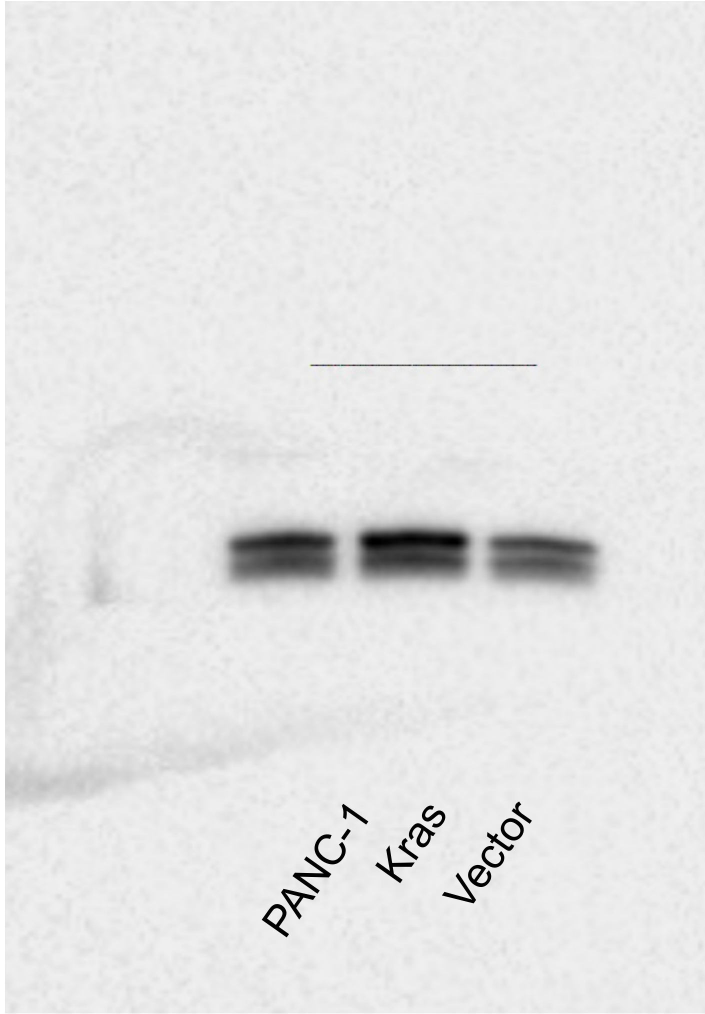


Kras

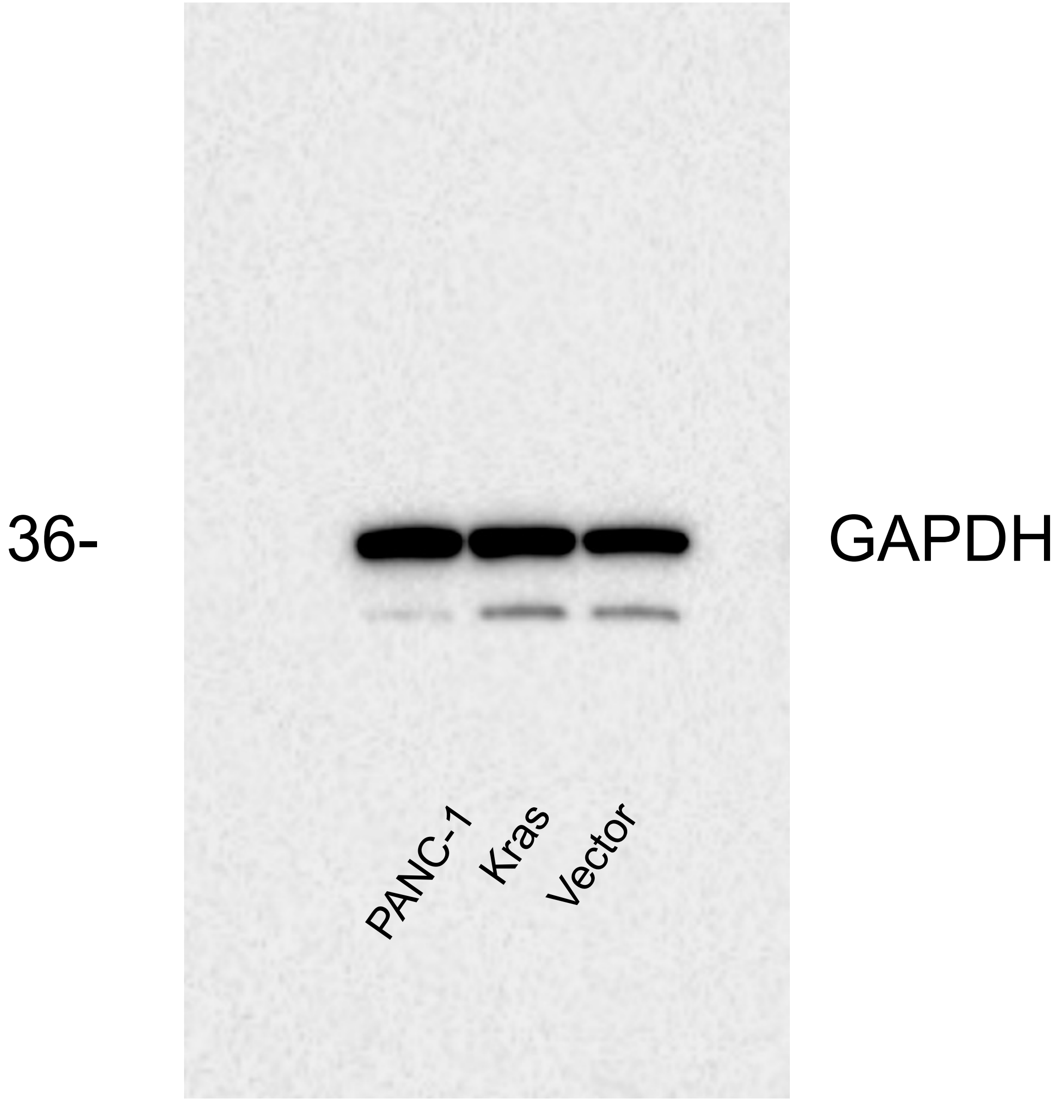
36-
GAPDH
PANC-1
Kras
Vector

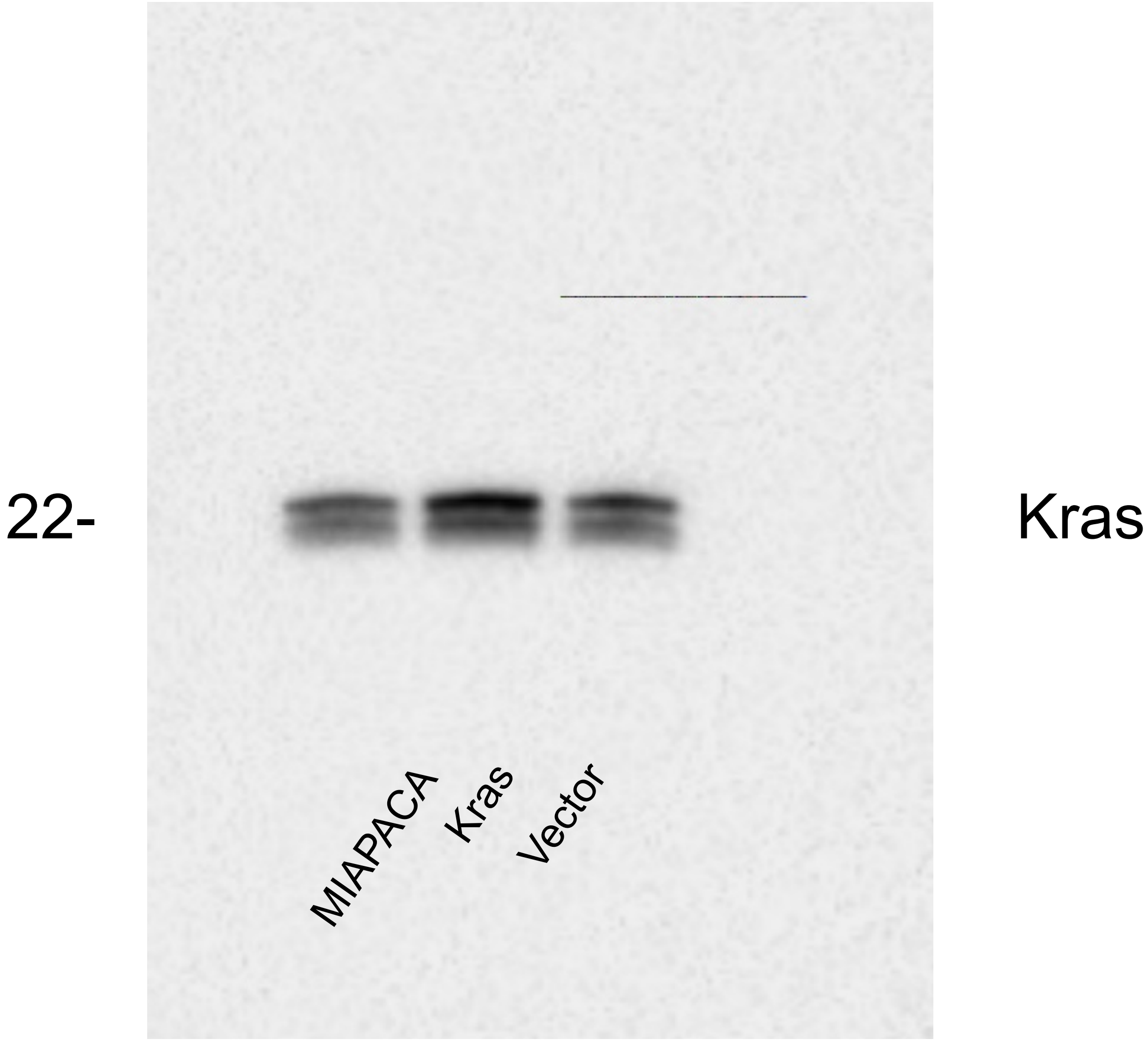
22-
Kras
MIAPACA
Kras
Vector

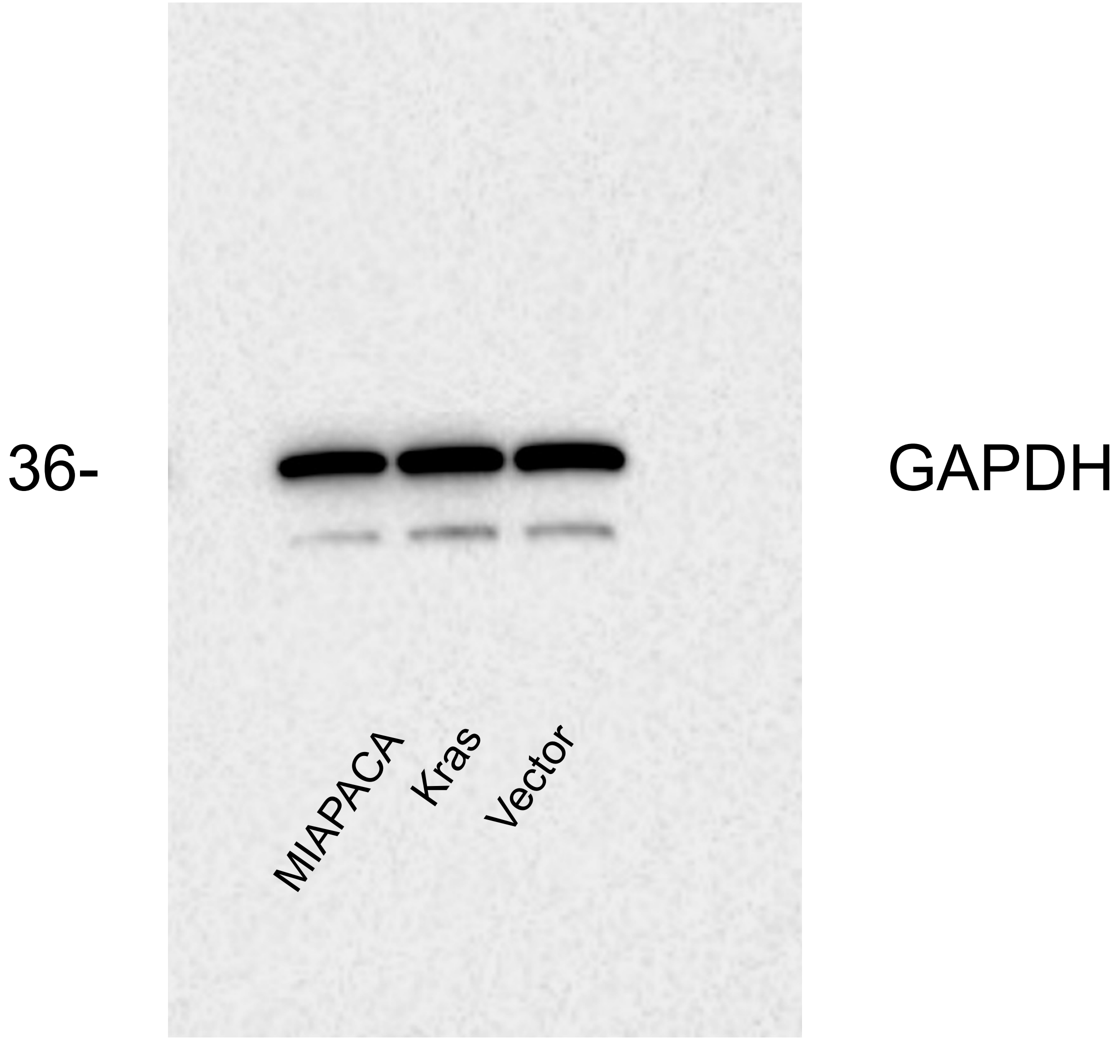
36-
GAPDH
MIAPACA
Kras
Vector

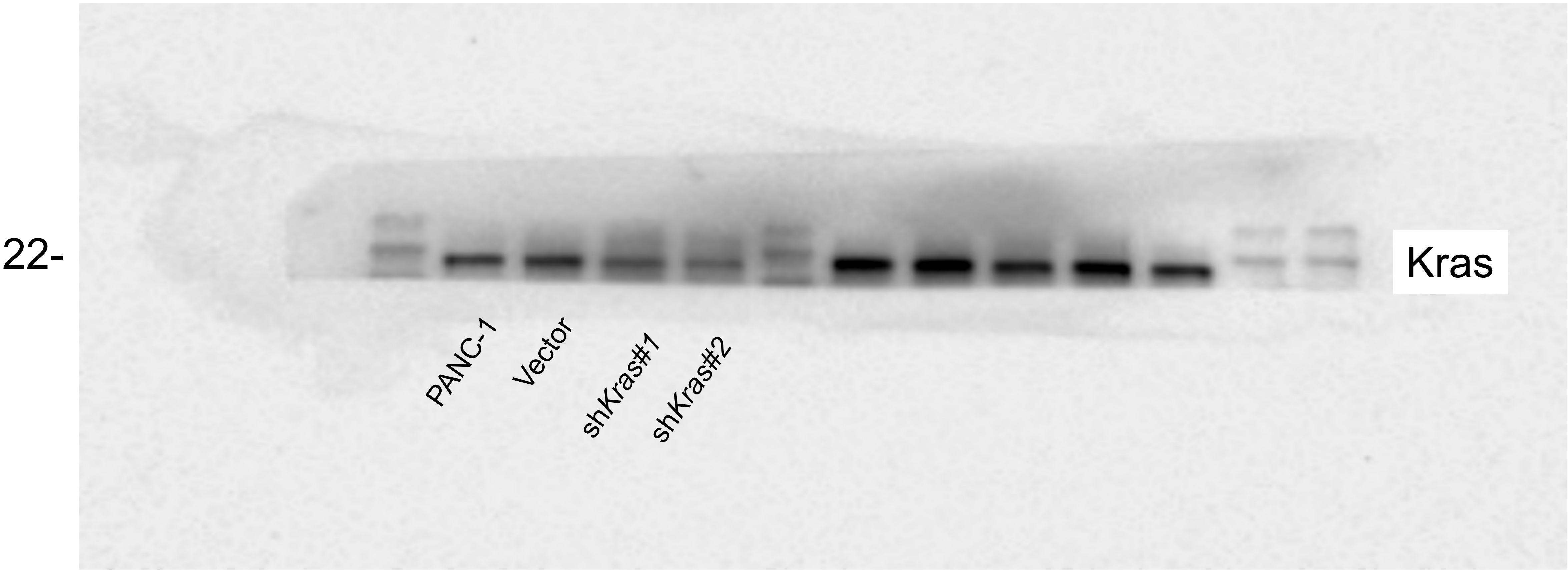
22-
PANC-1
Vector
shKras#1
shKras#2
Kras

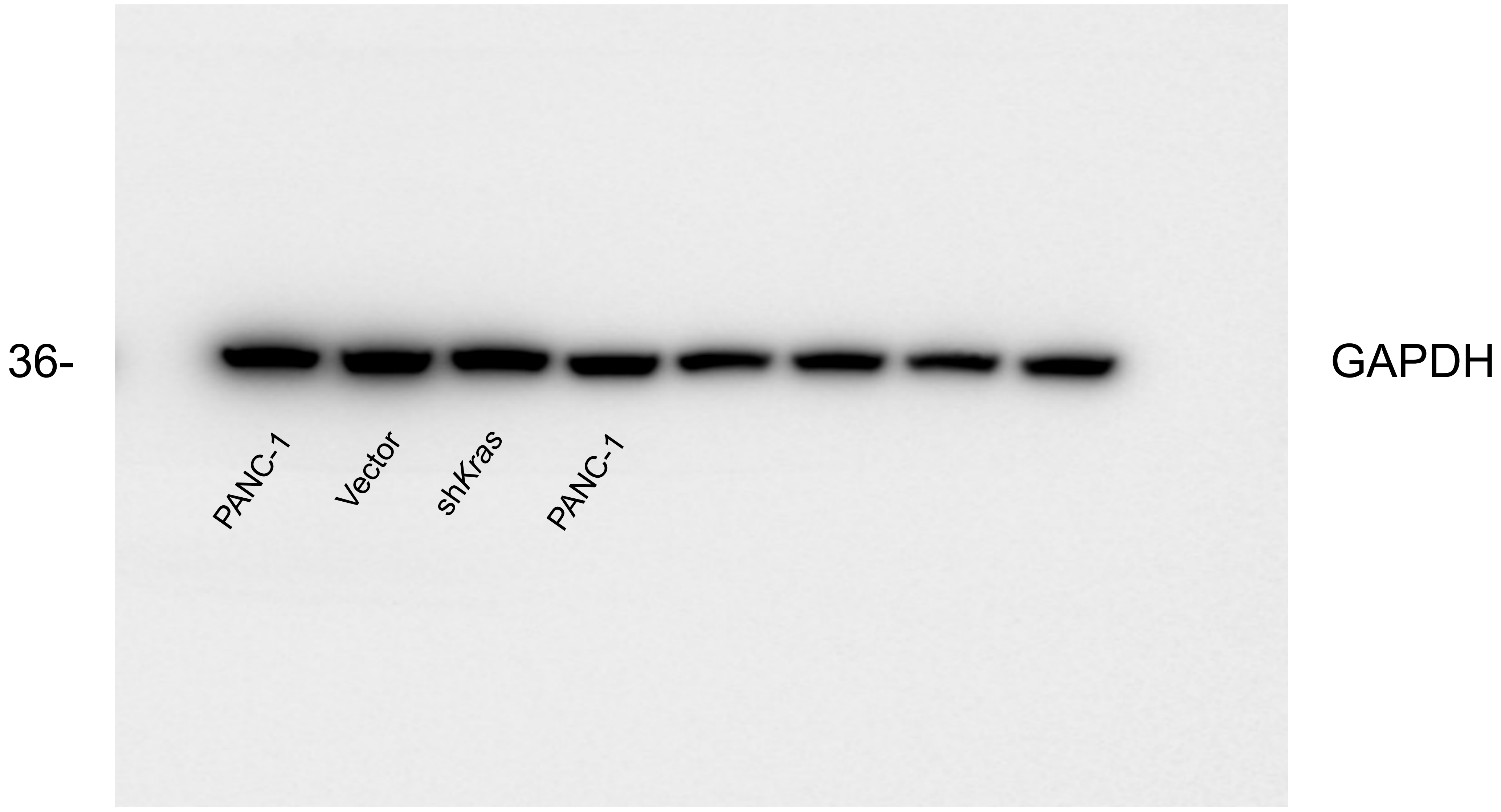
36-
GAPDH
PANC-1
Vector
shKras
PANC-1

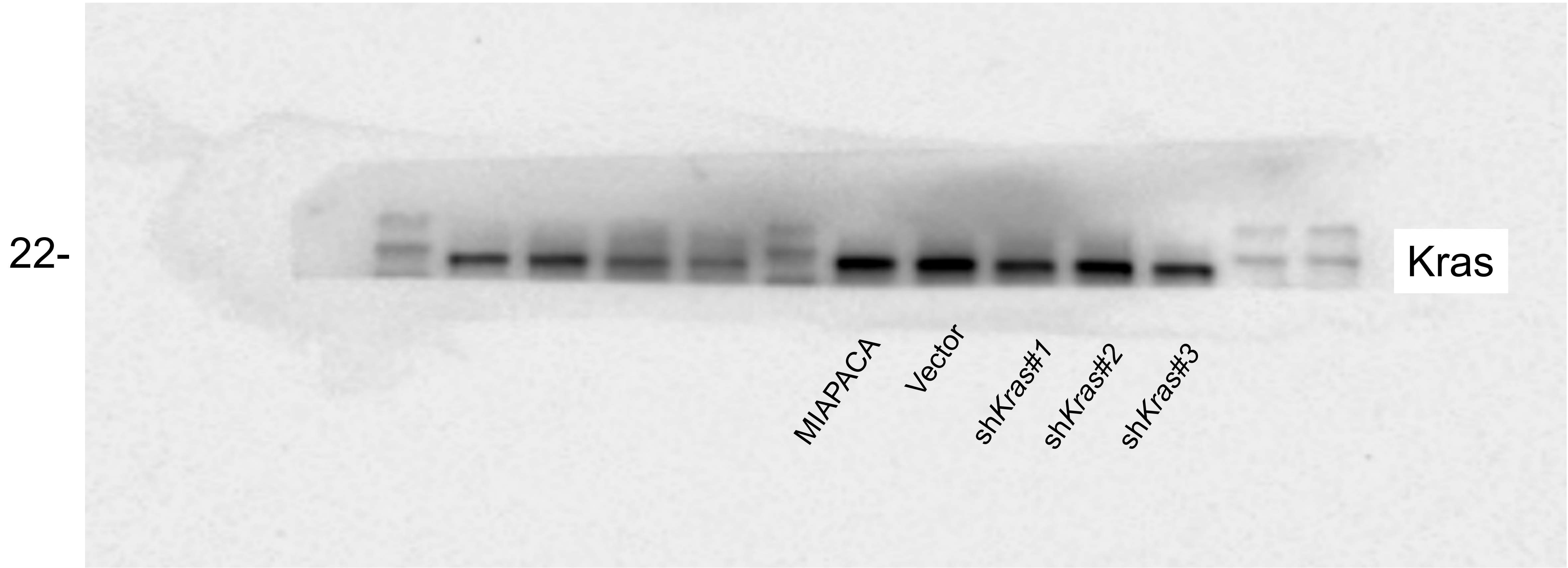
22-
Kras
MIAPACA
Vector
shKras#1
shKras#2
shKras#3

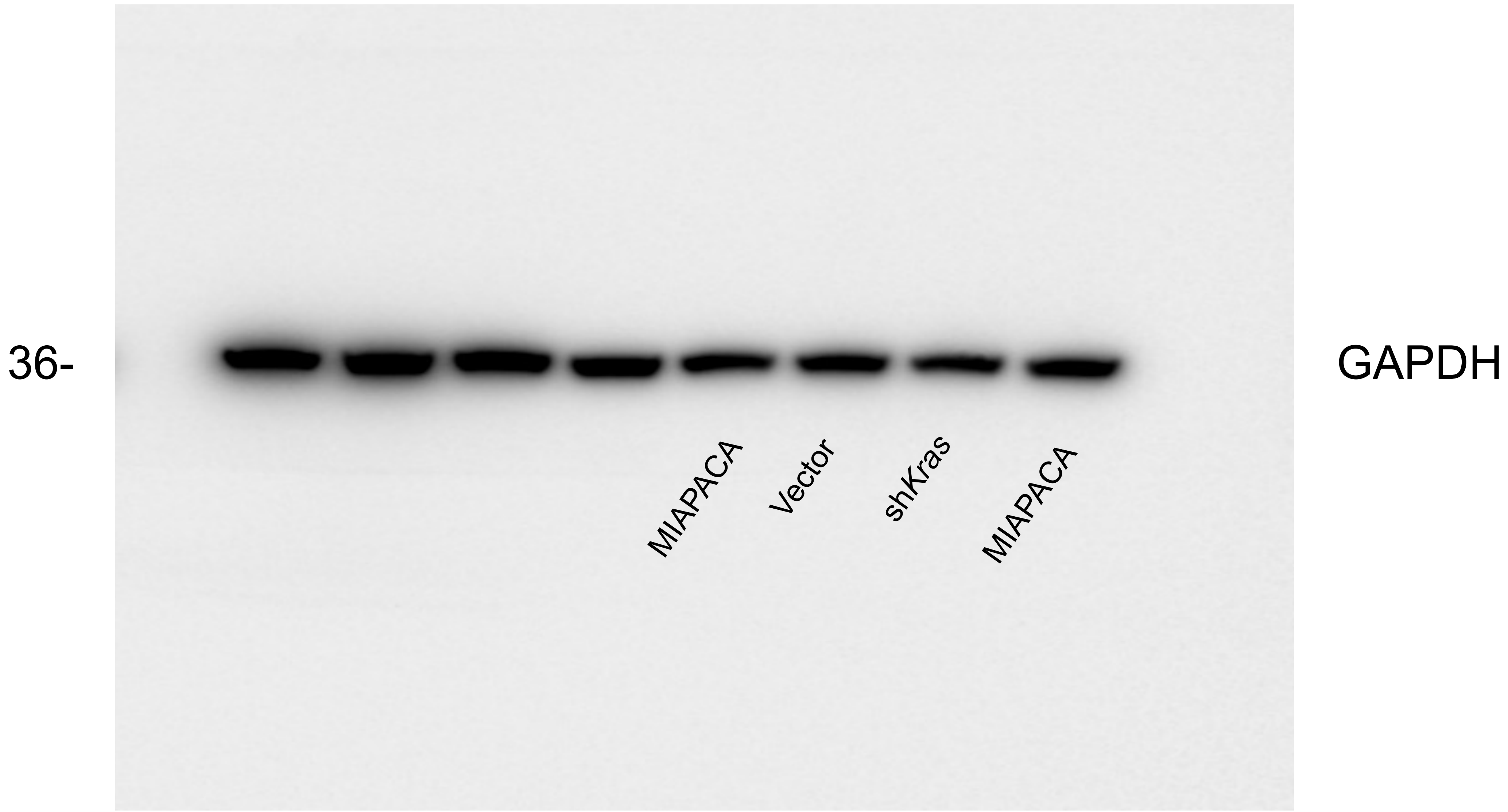

36-
GAPDH
MIAPACA
Vector
shKras
MIAPACA

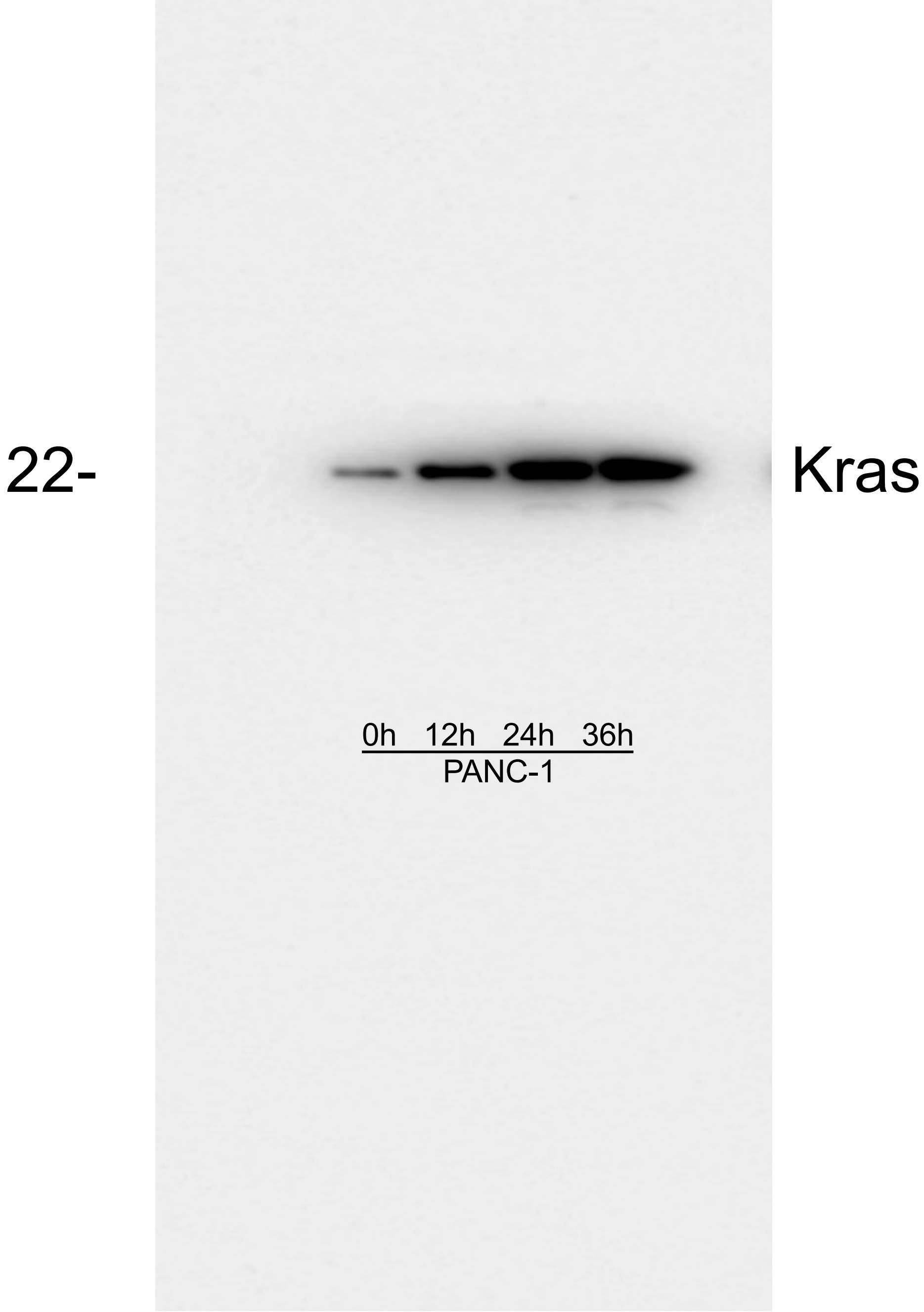

22-
Kras
0h 12h 24h 36h
PANC-1

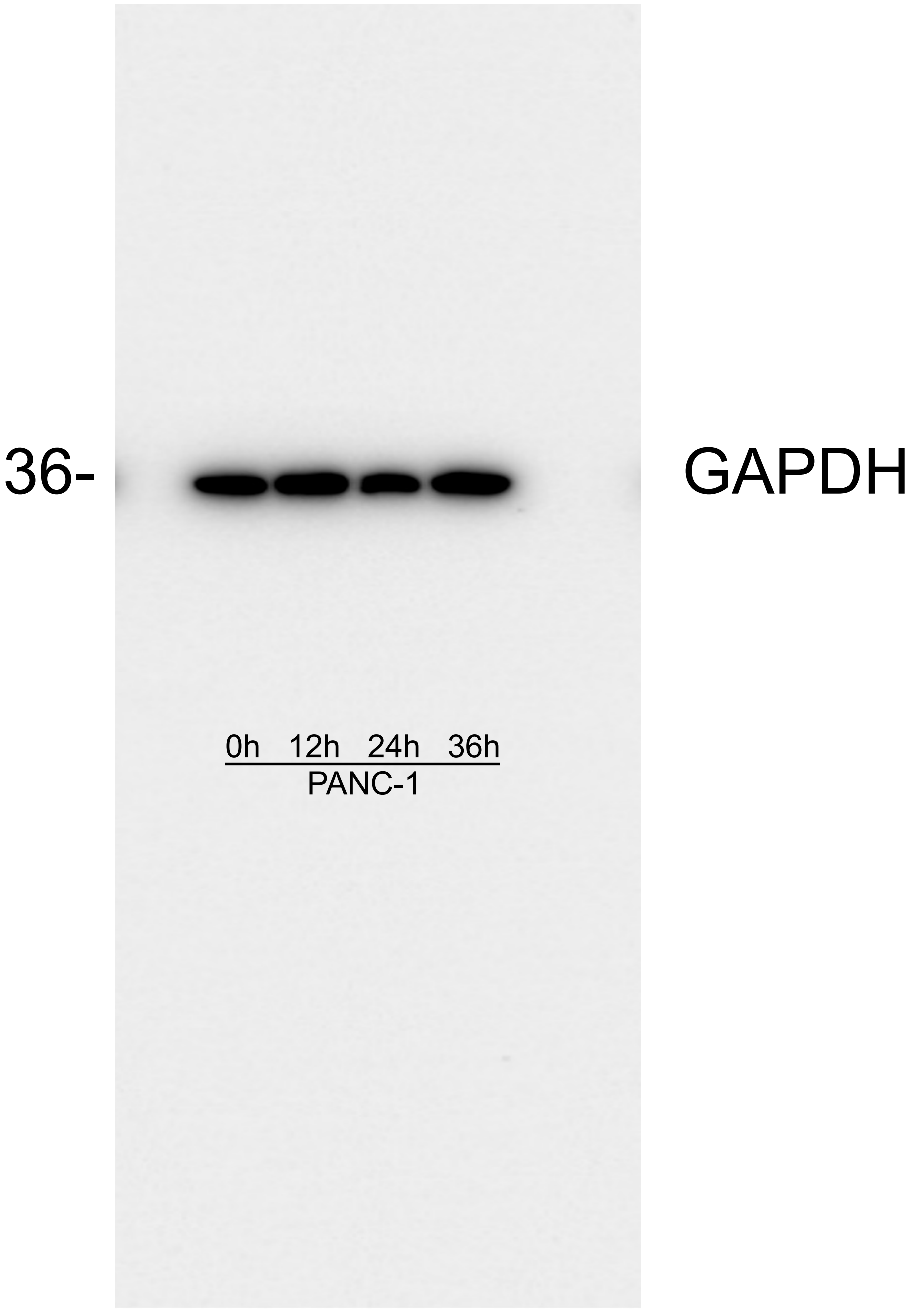
36-
GAPDH
0h 12h 24h 36h
PANC-1

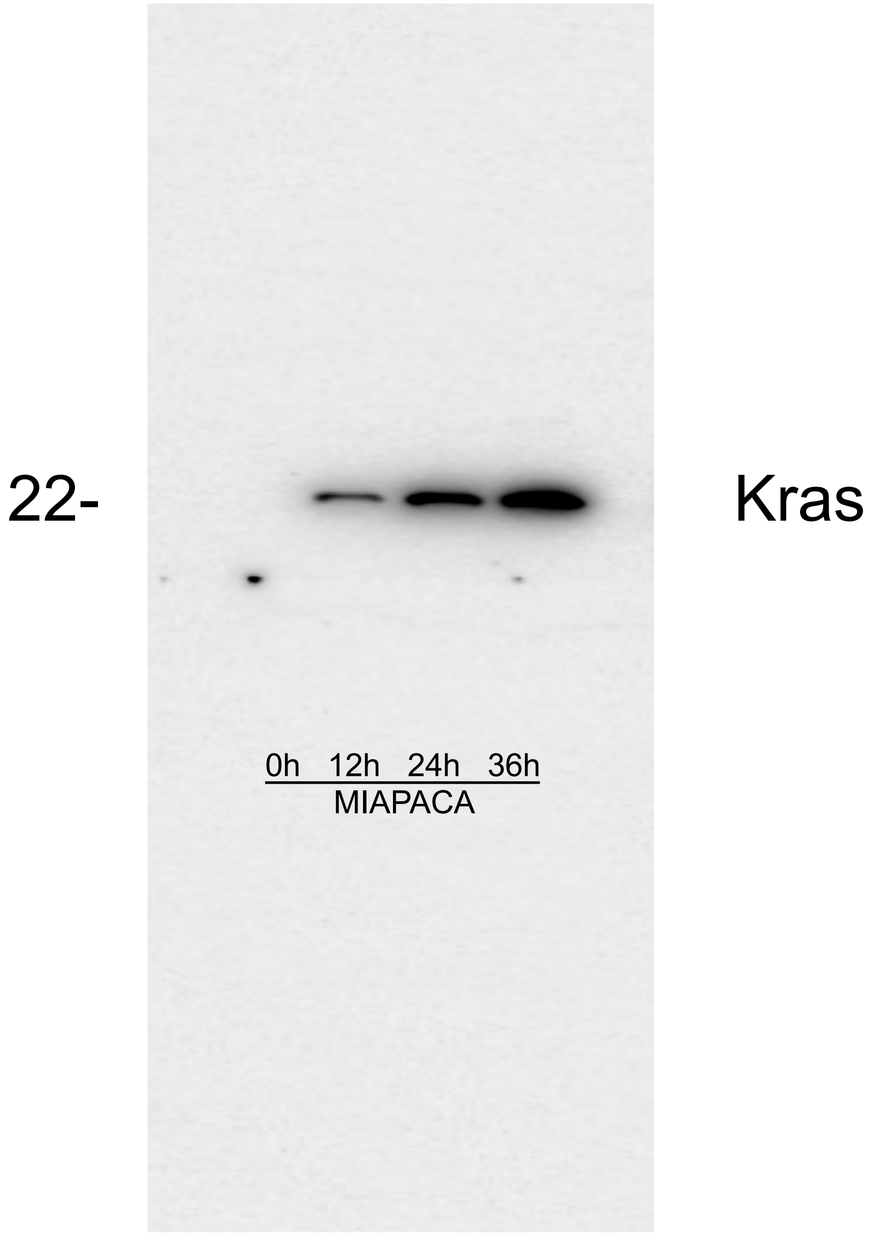

22-
Kras
0h 12h 24h 36h
MIAPACA

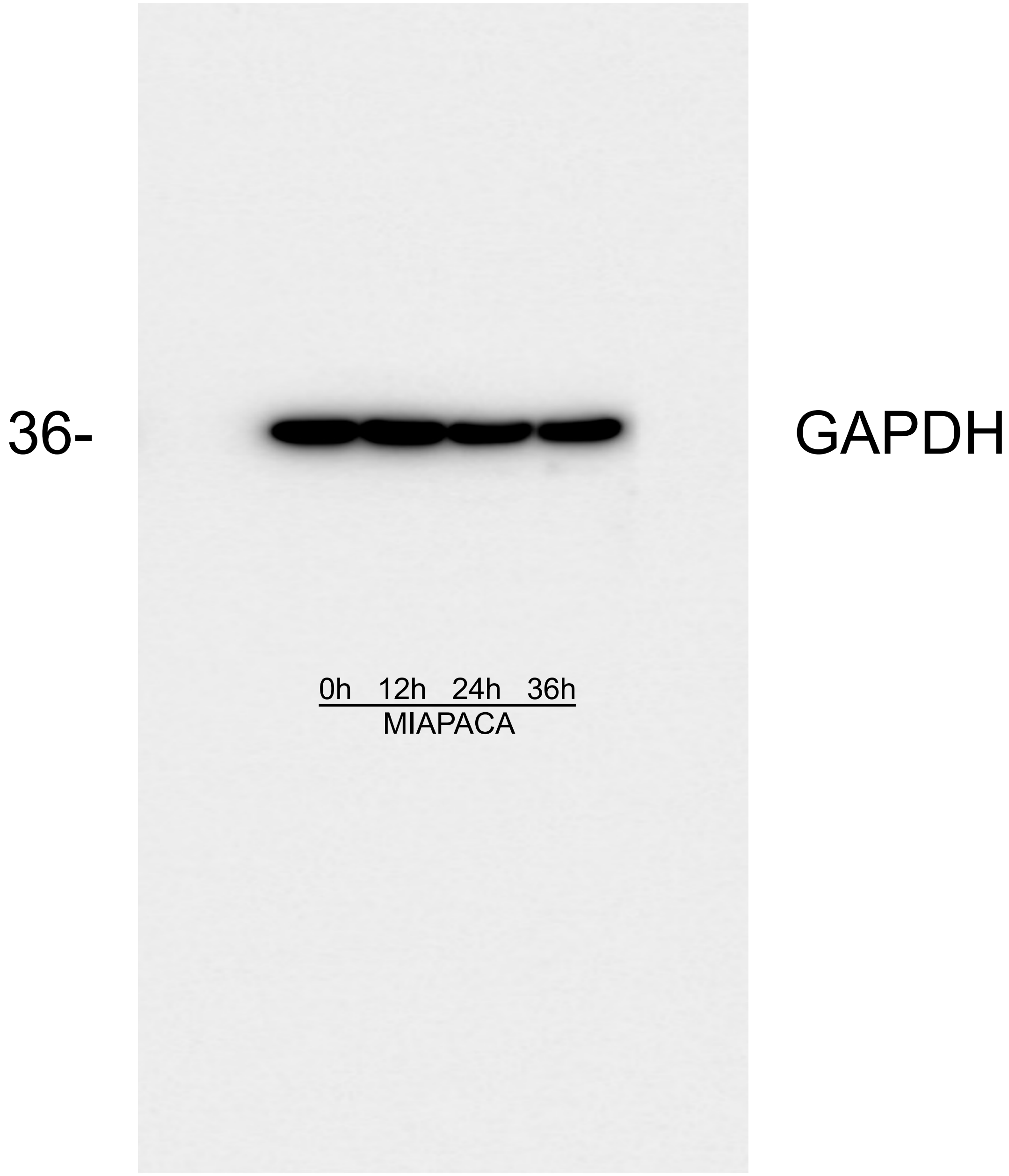
36-
GAPDH
0h 12h 24h 36h
MIAPACA
